# Supplementary material for: Classification of Environmental Strains from Order to Genus Levels Using Lipid and Protein MALDI-ToF Fingerprintings and Chemotaxonomic Network Analysis
Source: Microorganisms. 2022 Apr 17;10(4):831. doi: 10.3390/microorganisms10040831 (PMC9032901; doi:10.3390/microorganisms10040831)
Supplement: Supplementary file 1 [file microorganisms-10-00831-s001.zip › microorganisms-1595918-supplementary.pdf]

Supporting information

Classification of environmental strains from order to genus levels using lipid and protein MALDI-ToF fingerprintings and chemotaxonomic network analysis

Marceau Levasseur [1], Téo Hebra [1], Nicolas Elie[1], Vincent Guérineau[1], David Touboul [1,2]* and Véronique Eparvier [1]*

[1] Université Paris-Saclay, CNRS, Institut de Chimie des Substances Naturelles (ICSN), UPR 2301, Avenue de la Terrasse, 91 198 Gif-sur-Yvette, France; marceau.levasseur@cnrs.fr (M.L.); teo.hebra@cnrs.fr (T.H.); nicolas.elie@cnrs.fr (N.E.); vincent.guerineau@cnrs.fr (V.G.); david.touboul@cnrs.fr (D.T.) ; veronique.eparvier@cnrs.fr (V.E.)

[2] Université Paris-Saclay, CNRS, Laboratoire de Chimie Moléculaire (LCM), École Polytechnique, 91 128 Palaiseau, France; david.touboul@cnrs.fr (D.T.)

* Correspondence: david.touboul@cnrs.fr (D.T.); veronique.eparvier@cnrs.fr (V.E.); Tel.: +33-169-823-032 (D.T.); Tel.: +33-169-823-679 (V.E.)

TABLE OF CONTENTS

Table S1. Summary table of studied bacterial strains (RKI) 2

Table S2. Summary table of studied bacterial strains (BSNB)...21

Table S3. Summary table of studied fungal strains (BSNB)..26

Table S4. 16S of studied bacterial strains (BSNB) 34

Table S5. ITS of studied fungal strains (BSNB) 75

Figure S1. R script used: generation of a .mgf and .csv files 129

Table S6. Parameters used for constructing chemotaxonomic networks on MetGem.......131

TABLE S1. SUMMARY TABLE OF STUDIED BACTERIAL STRAINS (RKI)

**Names**	**Order**	**Genus**
Achromobacter_xylosoxidans_HST_107_01	Burkholderiales	*Achromobacter*
Achromobacter_xylosoxidans_HST_107_02	Burkholderiales	*Achromobacter*
Achromobacter_xylosoxidans_HST_115_01	Burkholderiales	*Achromobacter*
Achromobacter_xylosoxidans_HST_115_02	Burkholderiales	*Achromobacter*
Acinetobacter_baumannii_DSM_24110_01	Pseudomonadales	*Acinetobacter*
Acinetobacter_baumannii_DSM_24110_02	Pseudomonadales	*Acinetobacter*
Acinetobacter_baumannii_DSM_24110_03	Pseudomonadales	*Acinetobacter*
Acinetobacter_baumannii_DSM_24110_04	Pseudomonadales	*Acinetobacter*
Aeromonas_hydrophila_subsp_hydrophila_DSM_30187_01	Aeromonadales	*Aeromonas*
Aeromonas_hydrophila_subsp_hydrophila_DSM_30187_02	Aeromonadales	*Aeromonas*
Aeromonas_hydrophila_subsp_hydrophila_DSM_30187_03	Aeromonadales	*Aeromonas*
Aeromonas_hydrophila_subsp_hydrophila_DSM_30187_04	Aeromonadales	*Aeromonas*
Bacillus_alveayuensis_DSM_19092_01	Bacillales	*Bacillus*
Bacillus_alveayuensis_DSM_19092_02	Bacillales	*Bacillus*
Bacillus_alveayuensis_DSM_19092_03	Bacillales	*Bacillus*
Bacillus_alveayuensis_DSM_19092_04	Bacillales	*Bacillus*
Bacillus_aminovorans_DSM_4337_01	Bacillales	*Bacillus*
Bacillus_aminovorans_DSM_4337_02	Bacillales	*Bacillus*
Bacillus_aminovorans_DSM_4337_03	Bacillales	*Bacillus*
Bacillus_aminovorans_DSM_4337_04	Bacillales	*Bacillus*
Bacillus_amyloliquefaciens_ssp_amyloliquefaciens_DSM_7_01	Bacillales	*Bacillus*
Bacillus_amyloliquefaciens_ssp_amyloliquefaciens_DSM_7_02	Bacillales	*Bacillus*
Bacillus_amyloliquefaciens_ssp_amyloliquefaciens_DSM_7_03	Bacillales	*Bacillus*
Bacillus_amyloliquefaciens_ssp_amyloliquefaciens_DSM_7_04	Bacillales	*Bacillus*
Bacillus_anthracis_A1_01	Bacillales	*Bacillus*
Bacillus_anthracis_A1_02	Bacillales	*Bacillus*
Bacillus_anthracis_A1_03	Bacillales	*Bacillus*
Bacillus_anthracis_A1_04	Bacillales	*Bacillus*

Bacillus_atrophaeus_7264_01	Bacillales	*Bacillus*
Bacillus_atrophaeus_7264_02	Bacillales	*Bacillus*
Bacillus_atrophaeus_7264_03	Bacillales	*Bacillus*
Bacillus_atrophaeus_7264_04	Bacillales	*Bacillus*
Bacillus_bataviensis_DSM_15601_01	Bacillales	*Bacillus*
Bacillus_bataviensis_DSM_15601_02	Bacillales	*Bacillus*
Bacillus_bataviensis_DSM_15601_03	Bacillales	*Bacillus*
Bacillus_bataviensis_DSM_15601_04	Bacillales	*Bacillus*
Bacillus_clarkii_DSM_9725_01	Bacillales	*Bacillus*
Bacillus_clarkii_DSM_9725_02	Bacillales	*Bacillus*
Bacillus_clarkii_DSM_9725_03	Bacillales	*Bacillus*
Bacillus_clarkii_DSM_9725_04	Bacillales	*Bacillus*
Bacillus_clausii_DSM_8716_01	Bacillales	*Bacillus*
Bacillus_clausii_DSM_8716_02	Bacillales	*Bacillus*
Bacillus_clausii_DSM_8716_03	Bacillales	*Bacillus*
Bacillus_clausii_DSM_8716_04	Bacillales	*Bacillus*
Bacillus_coagulans_DSM_1_01	Bacillales	*Bacillus*
Bacillus_coagulans_DSM_1_02	Bacillales	*Bacillus*
Bacillus_coagulans_DSM_1_03	Bacillales	*Bacillus*
Bacillus_coagulans_DSM_1_04	Bacillales	*Bacillus*
Bacillus_cohnii_DSM_6350_01	Bacillales	*Bacillus*
Bacillus_cohnii_DSM_6350_02	Bacillales	*Bacillus*
Bacillus_cohnii_DSM_6350_03	Bacillales	*Bacillus*
Bacillus_cohnii_DSM_6350_04	Bacillales	*Bacillus*
Bacillus_cucumis_DSM_101566_01	Bacillales	*Bacillus*
Bacillus_cucumis_DSM_101566_02	Bacillales	*Bacillus*
Bacillus_cucumis_DSM_101566_03	Bacillales	*Bacillus*
Bacillus_cucumis_DSM_101566_04	Bacillales	*Bacillus*
Bacillus_cytotoxicus_DSM_22905_01	Bacillales	*Bacillus*
Bacillus_cytotoxicus_DSM_22905_02	Bacillales	*Bacillus*

Bacillus_cytotoxicus_DSM_22905_03	Bacillales	*Bacillus*
Bacillus_cytotoxicus_DSM_22905_04	Bacillales	*Bacillus*
Bacillus_ectoiniformans_DSM_28970_01	Bacillales	*Bacillus*
Bacillus_ectoiniformans_DSM_28970_02	Bacillales	*Bacillus*
Bacillus_ectoiniformans_DSM_28970_03	Bacillales	*Bacillus*
Bacillus_ectoiniformans_DSM_28970_04	Bacillales	*Bacillus*
Bacillus_encimensis_DSM_28241_01	Bacillales	*Bacillus*
Bacillus_encimensis_DSM_28241_02	Bacillales	*Bacillus*
Bacillus_encimensis_DSM_28241_03	Bacillales	*Bacillus*
Bacillus_encimensis_DSM_28241_04	Bacillales	*Bacillus*
Bacillus_firmus_DSM_12_01	Bacillales	*Bacillus*
Bacillus_firmus_DSM_12_02	Bacillales	*Bacillus*
Bacillus_flexus_DSM_1667_01	Bacillales	*Bacillus*
Bacillus_flexus_DSM_1667_02	Bacillales	*Bacillus*
Bacillus_flexus_DSM_1667_03	Bacillales	*Bacillus*
Bacillus_flexus_DSM_1667_04	Bacillales	*Bacillus*
Bacillus_halodurans_DSM_6940_01	Bacillales	*Bacillus*
Bacillus_halodurans_DSM_6940_02	Bacillales	*Bacillus*
Bacillus_halodurans_DSM_6940_03	Bacillales	*Bacillus*
Bacillus_halodurans_DSM_6940_04	Bacillales	*Bacillus*
Bacillus_halotolerans_18911_01	Bacillales	*Bacillus*
Bacillus_halotolerans_18911_02	Bacillales	*Bacillus*
Bacillus_halotolerans_18911_03	Bacillales	*Bacillus*
Bacillus_halotolerans_18911_04	Bacillales	*Bacillus*
Bacillus_horneckiae_23495_01	Bacillales	*Bacillus*
Bacillus_horneckiae_23495_02	Bacillales	*Bacillus*
Bacillus_horneckiae_23495_03	Bacillales	*Bacillus*
Bacillus_horneckiae_23495_04	Bacillales	*Bacillus*
Bacillus_infantis_DSM_19089_01	Bacillales	*Bacillus*
Bacillus_infantis_DSM_19089_02	Bacillales	*Bacillus*

Bacillus_infantis_DSM_19089_03	Bacillales	*Bacillus*
Bacillus_infantis_DSM_19089_04	Bacillales	*Bacillus*
Bacillus_lentus_DSM_5221_01	Bacillales	*Bacillus*
Bacillus_lentus_DSM_5221_02	Bacillales	*Bacillus*
Bacillus_lentus_DSM_5221_03	Bacillales	*Bacillus*
Bacillus_lentus_DSM_5221_04	Bacillales	*Bacillus*
Bacillus_licheniformis_DSM_13_01	Bacillales	*Bacillus*
Bacillus_licheniformis_DSM_13_02	Bacillales	*Bacillus*
Bacillus_megaterium_DSM_90_01	Bacillales	*Bacillus*
Bacillus_megaterium_DSM_90_02	Bacillales	*Bacillus*
Bacillus_mojavensis_DSM9206_01	Bacillales	*Bacillus*
Bacillus_mojavensis_DSM9206_02	Bacillales	*Bacillus*
Bacillus_mojavensis_DSM9206_03	Bacillales	*Bacillus*
Bacillus_mojavensis_DSM9206_04	Bacillales	*Bacillus*
Bacillus_muralis_DSM16288_01	Bacillales	*Bacillus*
Bacillus_muralis_DSM16288_02	Bacillales	*Bacillus*
Bacillus_muralis_DSM16288_03	Bacillales	*Bacillus*
Bacillus_muralis_DSM16288_04	Bacillales	*Bacillus*
Bacillus_mycoides_DSM_2048_01	Bacillales	*Bacillus*
Bacillus_mycoides_DSM_2048_02	Bacillales	*Bacillus*
Bacillus_mycoides_DSM_2048_03	Bacillales	*Bacillus*
Bacillus_pseudomycoides_DSM_12442_01	Bacillales	*Bacillus*
Bacillus_pseudomycoides_DSM_12442_02	Bacillales	*Bacillus*
Bacillus_pseudomycoides_DSM_12442_03	Bacillales	*Bacillus*
Bacillus_pseudomycoides_DSM_12442_04	Bacillales	*Bacillus*
Bacillus_psychrosaccharolyticus_DSM_6_01	Bacillales	*Bacillus*
Bacillus_psychrosaccharolyticus_DSM_6_02	Bacillales	*Bacillus*
Bacillus_psychrosaccharolyticus_DSM_6_03	Bacillales	*Bacillus*
Bacillus_psychrosaccharolyticus_DSM_6_04	Bacillales	*Bacillus*
Bacillus_pumilus_DSM_13835_01	Bacillales	*Bacillus*

Bacillus_pumilus_DSM_13835_02	Bacillales	*Bacillus*
Bacillus_salarius_DSM16461_01	Bacillales	*Bacillus*
Bacillus_salarius_DSM16461_02	Bacillales	*Bacillus*
Bacillus_salarius_DSM16461_03	Bacillales	*Bacillus*
Bacillus_salarius_DSM16461_04	Bacillales	*Bacillus*
Bacillus_siamensis_25261_01	Bacillales	*Bacillus*
Bacillus_siamensis_25261_02	Bacillales	*Bacillus*
Bacillus_siamensis_25261_03	Bacillales	*Bacillus*
Bacillus_siamensis_25261_04	Bacillales	*Bacillus*
Bacillus_simplex_DSM_1321_01	Bacillales	*Bacillus*
Bacillus_simplex_DSM_1321_02	Bacillales	*Bacillus*
Bacillus_simplex_DSM_1321_03	Bacillales	*Bacillus*
Bacillus_simplex_DSM_1321_04	Bacillales	*Bacillus*
Bacillus_sonorensis_DSM_13779_01	Bacillales	*Bacillus*
Bacillus_sonorensis_DSM_13779_02	Bacillales	*Bacillus*
Bacillus_sonorensis_DSM_13779_03	Bacillales	*Bacillus*
Bacillus_sonorensis_DSM_13779_04	Bacillales	*Bacillus*
Bacillus_sporothermodurans_DSM_10599_01	Bacillales	*Bacillus*
Bacillus_sporothermodurans_DSM_10599_02	Bacillales	*Bacillus*
Bacillus_sporothermodurans_DSM_10599_03	Bacillales	*Bacillus*
Bacillus_sporothermodurans_DSM_10599_04	Bacillales	*Bacillus*
Bacillus_subtilis_DSM_347_01	Bacillales	*Bacillus*
Bacillus_subtilis_DSM_347_02	Bacillales	*Bacillus*
Bacillus_subtilis_DSM_347_03	Bacillales	*Bacillus*
Bacillus_subtilis_DSM_347_04	Bacillales	*Bacillus*
Bacillus_thuringiensis_DSM_6073_01	Bacillales	*Bacillus*
Bacillus_thuringiensis_DSM_6073_02	Bacillales	*Bacillus*
Bacillus_thuringiensis_DSM_6073_03	Bacillales	*Bacillus*
Bacillus_thuringiensis_DSM_6073_04	Bacillales	*Bacillus*
Bacillus_vallismortis_DSM_11031_01	Bacillales	*Bacillus*

Bacillus_vallismortis_DSM_11031_02	Bacillales	*Bacillus*
Bacillus_vallismortis_DSM_11031_03	Bacillales	*Bacillus*
Bacillus_vallismortis_DSM_11031_04	Bacillales	*Bacillus*
Bacillus_weihenstephanensis_DSM_104119_01	Bacillales	*Bacillus*
Bacillus_weihenstephanensis_DSM_104119_02	Bacillales	*Bacillus*
Bacillus_weihenstephanensis_DSM_104119_03	Bacillales	*Bacillus*
Bacillus_weihenstephanensis_DSM_104119_04	Bacillales	*Bacillus*
Brucella_abortus_S19_delta_01	Rhizobiales	*Brucella*
Brucella_abortus_S19_delta_02	Rhizobiales	*Brucella*
Brucella_abortus_S19_delta_03	Rhizobiales	*Brucella*
Brucella_abortus_S19_delta_04	Rhizobiales	*Brucella*
Brucella_canis_A183_5_01	Rhizobiales	*Brucella*
Brucella_canis_A183_5_02	Rhizobiales	*Brucella*
Brucella_canis_A183_5_03	Rhizobiales	*Brucella*
Brucella_canis_A183_5_04	Rhizobiales	*Brucella*
Brucella_melitensis_A146_13_Ringversuch_A938_3_01	Rhizobiales	*Brucella*
Brucella_melitensis_A146_13_Ringversuch_A938_3_02	Rhizobiales	*Brucella*
Brucella_melitensis_A146_13_Ringversuch_A938_3_03	Rhizobiales	*Brucella*
Brucella_melitensis_A146_13_Ringversuch_A938_3_04	Rhizobiales	*Brucella*
Brucella_microti_CCM_4915_01	Rhizobiales	*Brucella*
Brucella_microti_CCM_4915_02	Rhizobiales	*Brucella*
Brucella_neotomae_A148_7_01	Rhizobiales	*Brucella*
Brucella_neotomae_A148_7_02	Rhizobiales	*Brucella*
Brucella_neotomae_A148_7_03	Rhizobiales	*Brucella*
Brucella_neotomae_A148_7_04	Rhizobiales	*Brucella*
Brucella_ovis_A138_7_01	Rhizobiales	*Brucella*
Brucella_ovis_A138_7_02	Rhizobiales	*Brucella*
Brucella_ovis_A138_7_03	Rhizobiales	*Brucella*
Brucella_ovis_A138_7_04	Rhizobiales	*Brucella*
Brucella_pinnipedialis_A148_8_01	Rhizobiales	*Brucella*

Brucella_pinnipedialis_A148_8_02	Rhizobiales	*Brucella*
Brucella_pinnipedialis_A148_8_03	Rhizobiales	*Brucella*
Brucella_pinnipedialis_A148_8_04	Rhizobiales	*Brucella*
Brucella_vulpis_DSM_101715_01	Rhizobiales	*Brucella*
Brucella_vulpis_DSM_101715_02	Rhizobiales	*Brucella*
Brucella_vulpis_DSM_101715_03	Rhizobiales	*Brucella*
Brucella_vulpis_DSM_101715_04	Rhizobiales	*Brucella*
Burkholderia_caledonica_LMG_19076_01	Burkholderiales	*Burkholderia*
Burkholderia_caledonica_LMG_19076_02	Burkholderiales	*Burkholderia*
Burkholderia_caledonica_LMG_19076_03	Burkholderiales	*Burkholderia*
Burkholderia_caribensis_LMG_18531_01	Burkholderiales	*Burkholderia*
Burkholderia_caribensis_LMG_18531_02	Burkholderiales	*Burkholderia*
Burkholderia_caribensis_LMG_18531_03	Burkholderiales	*Burkholderia*
Burkholderia_CC_ambifaria_DSM_16087_01	Burkholderiales	*Burkholderia*
Burkholderia_CC_ambifaria_DSM_16087_02	Burkholderiales	*Burkholderia*
Burkholderia_CC_ambifaria_DSM_16087_03	Burkholderiales	*Burkholderia*
Burkholderia_CC_ambifaria_DSM_16087_04	Burkholderiales	*Burkholderia*
Burkholderia_CC_anthina_DSM_16086_01	Burkholderiales	*Burkholderia*
Burkholderia_CC_anthina_DSM_16086_02	Burkholderiales	*Burkholderia*
Burkholderia_CC_anthina_DSM_16086_03	Burkholderiales	*Burkholderia*
Burkholderia_CC_anthina_DSM_16086_04	Burkholderiales	*Burkholderia*
Burkholderia_CC_arboris_DSM_23435_01	Burkholderiales	*Burkholderia*
Burkholderia_CC_arboris_DSM_23435_02	Burkholderiales	*Burkholderia*
Burkholderia_CC_arboris_DSM_23435_03	Burkholderiales	*Burkholderia*
Burkholderia_CC_arboris_DSM_23435_04	Burkholderiales	*Burkholderia*
Burkholderia_CC_cenocepacia_DSM_16553_01	Burkholderiales	*Burkholderia*
Burkholderia_CC_cenocepacia_DSM_16553_02	Burkholderiales	*Burkholderia*
Burkholderia_CC_cenocepacia_DSM_16553_03	Burkholderiales	*Burkholderia*
Burkholderia_CC_cenocepacia_DSM_16553_04	Burkholderiales	*Burkholderia*
Burkholderia_CC_cepacia_DSM_7288_01	Burkholderiales	*Burkholderia*

Burkholderia_CC_cepacia_DSM_7288_02	Burkholderiales	*Burkholderia*
Burkholderia_CC_cepacia_DSM_7288_03	Burkholderiales	*Burkholderia*
Burkholderia_CC_contaminans_DSM_22706 _01	Burkholderiales	*Burkholderia*
Burkholderia_CC_contaminans_DSM_22706 _02	Burkholderiales	*Burkholderia*
Burkholderia_CC_contaminans_DSM_22706 _03	Burkholderiales	*Burkholderia*
Burkholderia_CC_contaminans_DSM_22706 _04	Burkholderiales	*Burkholderia*
Burkholderia_CC_diffusa_DSM_23434_01	Burkholderiales	*Burkholderia*
Burkholderia_CC_diffusa_DSM_23434_02	Burkholderiales	*Burkholderia*
Burkholderia_CC_diffusa_DSM_23434_03	Burkholderiales	*Burkholderia*
Burkholderia_CC_diffusa_DSM_23434_04	Burkholderiales	*Burkholderia*
Burkholderia_CC_dolosa_DSM_16088_01	Burkholderiales	*Burkholderia*
Burkholderia_CC_dolosa_DSM_16088_02	Burkholderiales	*Burkholderia*
Burkholderia_CC_dolosa_DSM_16088_03	Burkholderiales	*Burkholderia*
Burkholderia_CC_dolosa_DSM_16088_04	Burkholderiales	*Burkholderia*
Burkholderia_CC_lata_DSM_23089_01	Burkholderiales	*Burkholderia*
Burkholderia_CC_lata_DSM_23089_02	Burkholderiales	*Burkholderia*
Burkholderia_CC_lata_DSM_23089_03	Burkholderiales	*Burkholderia*
Burkholderia_CC_lata_DSM_23089_04	Burkholderiales	*Burkholderia*
Burkholderia_CC_latens_DSM_23436_01	Burkholderiales	*Burkholderia*
Burkholderia_CC_latens_DSM_23436_02	Burkholderiales	*Burkholderia*
Burkholderia_CC_latens_DSM_23436_03	Burkholderiales	*Burkholderia*
Burkholderia_CC_latens_DSM_23436_04	Burkholderiales	*Burkholderia*
Burkholderia_CC_metallica_DSM_23519_01	Burkholderiales	*Burkholderia*
Burkholderia_CC_metallica_DSM_23519_02	Burkholderiales	*Burkholderia*
Burkholderia_CC_metallica_DSM_23519_03	Burkholderiales	*Burkholderia*
Burkholderia_CC_metallica_DSM_23519_04	Burkholderiales	*Burkholderia*
Burkholderia_CC_multivorans_ATCC_17616_01	Burkholderiales	*Burkholderia*
Burkholderia_CC_multivorans_ATCC_17616_02	Burkholderiales	*Burkholderia*
Burkholderia_CC_multivorans_ATCC_17616_03	Burkholderiales	*Burkholderia*
Burkholderia_CC_pseudomultivorans_LMG_16669_01	Burkholderiales	*Burkholderia*

Burkholderia_CC_pseudomultivorans_LMG_16669_02	Burkholderiales	*Burkholderia*
Burkholderia_CC_pseudomultivorans_LMG_16669_03	Burkholderiales	*Burkholderia*
Burkholderia_CC_pseudomultivorans_LMG_16669_04	Burkholderiales	*Burkholderia*
Burkholderia_CC_puraquae_CAMPA_565_HST_189_01	Burkholderiales	*Burkholderia*
Burkholderia_CC_puraquae_CAMPA_565_HST_189_02	Burkholderiales	*Burkholderia*
Burkholderia_CC_puraquae_CAMPA_565_HST_189_03	Burkholderiales	*Burkholderia*
Burkholderia_CC_puraquae_CAMPA_565_HST_189_04	Burkholderiales	*Burkholderia*
Burkholderia_CC_pyrrocinia_ATCC_15958_DSM_10685_01	Burkholderiales	*Burkholderia*
Burkholderia_CC_pyrrocinia_ATCC_15958_DSM_10685_02	Burkholderiales	*Burkholderia*
Burkholderia_CC_pyrrocinia_ATCC_15958_DSM_10685_03	Burkholderiales	*Burkholderia*
Burkholderia_CC_pyrrocinia_ATCC_15958_DSM_10685_04	Burkholderiales	*Burkholderia*
Burkholderia_CC_seminalis_DSM_23518_01	Burkholderiales	*Burkholderia*
Burkholderia_CC_seminalis_DSM_23518_02	Burkholderiales	*Burkholderia*
Burkholderia_CC_seminalis_DSM_23518_03	Burkholderiales	*Burkholderia*
Burkholderia_CC_seminalis_DSM_23518_04	Burkholderiales	*Burkholderia*
Burkholderia_CC_stabilis_DSM_16586_LMG_14294_01	Burkholderiales	*Burkholderia*
Burkholderia_CC_stabilis_DSM_16586_LMG_14294_02	Burkholderiales	*Burkholderia*
Burkholderia_CC_stabilis_DSM_16586_LMG_14294_03	Burkholderiales	*Burkholderia*
Burkholderia_CC_stabilis_DSM_16586_LMG_14294_04	Burkholderiales	*Burkholderia*
Burkholderia_CC_stagnalis_LMG_28156_01	Burkholderiales	*Burkholderia*
Burkholderia_CC_stagnalis_LMG_28156_02	Burkholderiales	*Burkholderia*
Burkholderia_CC_stagnalis_LMG_28156_03	Burkholderiales	*Burkholderia*
Burkholderia_CC_stagnalis_LMG_28156_04	Burkholderiales	*Burkholderia*
Burkholderia_CC_territorii_LMG_28158_01	Burkholderiales	*Burkholderia*
Burkholderia_CC_territorii_LMG_28158_02	Burkholderiales	*Burkholderia*
Burkholderia_CC_territorii_LMG_28158_03	Burkholderiales	*Burkholderia*
Burkholderia_CC_territorii_LMG_28158_04	Burkholderiales	*Burkholderia*
Burkholderia_CC_ubonensis_DSM_17311_01	Burkholderiales	*Burkholderia*
Burkholderia_CC_ubonensis_DSM_17311_02	Burkholderiales	*Burkholderia*
Burkholderia_CC_ubonensis_DSM_17311_03	Burkholderiales	*Burkholderia*

Burkholderia_CC_ubonensis_DSM_17311_04	Burkholderiales	*Burkholderia*
Burkholderia_CC_vietnamiensis_LMG_10929_01	Burkholderiales	*Burkholderia*
Burkholderia_CC_vietnamiensis_LMG_10929_02	Burkholderiales	*Burkholderia*
Burkholderia_CC_vietnamiensis_LMG_10929_03	Burkholderiales	*Burkholderia*
Burkholderia_CC_vietnamiensis_LMG_10929_04	Burkholderiales	*Burkholderia*
Burkholderia_gladioli_DSM_8361_01	Burkholderiales	*Burkholderia*
Burkholderia_gladioli_DSM_8361_02	Burkholderiales	*Burkholderia*
Burkholderia_gladioli_DSM_8361_03	Burkholderiales	*Burkholderia*
Burkholderia_gladioli_DSM_8361_04	Burkholderiales	*Burkholderia*
Burkholderia_glathei_LMG_14190_01	Burkholderiales	*Burkholderia*
Burkholderia_glathei_LMG_14190_02	Burkholderiales	*Burkholderia*
Burkholderia_glathei_LMG_14190_03	Burkholderiales	*Burkholderia*
Burkholderia_glumae_LMG_1277_01	Burkholderiales	*Burkholderia*
Burkholderia_glumae_LMG_1277_02	Burkholderiales	*Burkholderia*
Burkholderia_glumae_LMG_1277_03	Burkholderiales	*Burkholderia*
Burkholderia_hospita_LMG_20574_01	Burkholderiales	*Burkholderia*
Burkholderia_hospita_LMG_20574_02	Burkholderiales	*Burkholderia*
Burkholderia_hospita_LMG_20574_03	Burkholderiales	*Burkholderia*
Burkholderia_kururiensis_DSM_13464_01	Burkholderiales	*Burkholderia*
Burkholderia_kururiensis_DSM_13464_02	Burkholderiales	*Burkholderia*
Burkholderia_kururiensis_DSM_13464_03	Burkholderiales	*Burkholderia*
Burkholderia_kururiensis_DSM_13464_04	Burkholderiales	*Burkholderia*
Burkholderia_mallei_ATCC_23344_01	Burkholderiales	*Burkholderia*
Burkholderia_mallei_ATCC_23344_02	Burkholderiales	*Burkholderia*
Burkholderia_mallei_ATCC_23344_03	Burkholderiales	*Burkholderia*
Burkholderia_mallei_ATCC_23344_04	Burkholderiales	*Burkholderia*
Burkholderia_oklahomensis_DSM_21774_01	Burkholderiales	*Burkholderia*
Burkholderia_oklahomensis_DSM_21774_02	Burkholderiales	*Burkholderia*
Burkholderia_oklahomensis_DSM_21774_03	Burkholderiales	*Burkholderia*
Burkholderia_oklahomensis_DSM_21774_04	Burkholderiales	*Burkholderia*

Burkholderia_phymatum_DSM_17167_01	Burkholderiales	*Burkholderia*
Burkholderia_phymatum_DSM_17167_02	Burkholderiales	*Burkholderia*
Burkholderia_phymatum_DSM_17167_03	Burkholderiales	*Burkholderia*
Burkholderia_phytofirmans_DSM_17436_01	Burkholderiales	*Burkholderia*
Burkholderia_phytofirmans_DSM_17436_02	Burkholderiales	*Burkholderia*
Burkholderia_phytofirmans_DSM_17436_03	Burkholderiales	*Burkholderia*
Burkholderia_plantarii_LMG_10907_01	Burkholderiales	*Burkholderia*
Burkholderia_plantarii_LMG_10907_02	Burkholderiales	*Burkholderia*
Burkholderia_plantarii_LMG_10907_03	Burkholderiales	*Burkholderia*
Burkholderia_pseudomallei_CCUC_13790_01	Burkholderiales	*Burkholderia*
Burkholderia_pseudomallei_CCUC_13790_02	Burkholderiales	*Burkholderia*
Burkholderia_pseudomallei_CCUC_13790_03	Burkholderiales	*Burkholderia*
Burkholderia_thailandensis_DSM_13276_01	Burkholderiales	*Burkholderia*
Burkholderia_thailandensis_DSM_13276_02	Burkholderiales	*Burkholderia*
Burkholderia_thailandensis_DSM_13276_03	Burkholderiales	*Burkholderia*
Burkholderia_tuberum_LMG_21444_01	Burkholderiales	*Burkholderia*
Burkholderia_tuberum_LMG_21444_02	Burkholderiales	*Burkholderia*
Burkholderia_tuberum_LMG_21444_03	Burkholderiales	*Burkholderia*
Burkholderia_xenovorans_DSM_17367_01	Burkholderiales	*Burkholderia*
Burkholderia_xenovorans_DSM_17367_02	Burkholderiales	*Burkholderia*
Burkholderia_xenovorans_DSM_17367_03	Burkholderiales	*Burkholderia*
Campylobacter_jejuni_A669_01	Campylobacterales	*Campylobacter*
Campylobacter_jejuni_A669_02	Campylobacterales	*Campylobacter*
Campylobacter_jejuni_A669_03	Campylobacterales	*Campylobacter*
Campylobacter_jejuni_A669_04	Campylobacterales	*Campylobacter*
Citrobacter_amalonaticus_04_08695_01	Enterobacteriales	*Citrobacter*
Citrobacter_amalonaticus_04_08695_02	Enterobacteriales	*Citrobacter*
Citrobacter_diversus_ATCC_25408_01	Enterobacteriales	*Citrobacter*
Citrobacter_diversus_ATCC_25408_02	Enterobacteriales	*Citrobacter*
Citrobacter_freundii_DSM_30039_01	Enterobacteriales	*Citrobacter*

Citrobacter_freundii_DSM_30039_02	Enterobacteriales	*Citrobacter*
Citrobacter_freundii_DSM_30039_03	Enterobacteriales	*Citrobacter*
Citrobacter_freundii_DSM_30039_04	Enterobacteriales	*Citrobacter*
Corynebacterium_amycolatum_RRLK02_02_01	Actinomycetales	*Corynebacterium*
Corynebacterium_amycolatum_RRLK02_02_02	Actinomycetales	*Corynebacterium*
Corynebacterium_amycolatum_RRLK02_02_03	Actinomycetales	*Corynebacterium*
Corynebacterium_amycolatum_RRLK02_02_04	Actinomycetales	*Corynebacterium*
Edwardsiella_tarda_DSM_30052_01	Enterobacteriales	*Edwardsiella*
Edwardsiella_tarda_DSM_30052_02	Enterobacteriales	*Edwardsiella*
Enterobacter_aerogenes_DSM_30053_01	Enterobacteriales	*Enterobacter*
Enterobacter_aerogenes_DSM_30053_02	Enterobacteriales	*Enterobacter*
Enterobacter_cloacae_DSM_30054_01	Enterobacteriales	*Enterobacter*
Enterobacter_cloacae_DSM_30054_02	Enterobacteriales	*Enterobacter*
Enterobacter_gergoviae_ATCC_33426_01	Enterobacteriales	*Enterobacter*
Enterobacter_gergoviae_ATCC_33426_02	Enterobacteriales	*Enterobacter*
Enterobacter_sakazakii_04_01242_01	Enterobacteriales	*Enterobacter*
Enterobacter_sakazakii_04_01242_02	Enterobacteriales	*Enterobacter*
Enterococcus_faecalis_DSM_20371_01	Enterobacteriales	*Enterococcus*
Enterococcus_faecalis_DSM_20371_02	Enterobacteriales	*Enterococcus*
Enterococcus_faecalis_DSM_20371_03	Enterobacteriales	*Enterococcus*
Enterococcus_faecalis_DSM_20371_04	Enterobacteriales	*Enterococcus*
Escherichia_coli_DSM_3871_01	Enterobacteriales	*Escherichia*
Escherichia_coli_DSM_3871_02	Enterobacteriales	*Escherichia*
Escherichia_coli_DSM_3871_03	Enterobacteriales	*Escherichia*
Escherichia_coli_DSM_3871_04	Enterobacteriales	*Escherichia*
Francisella_guangzhouensis_DSM_102975_01	Thiotrichales	*Francisella*
Francisella_guangzhouensis_DSM_102975_02	Thiotrichales	*Francisella*
Francisella_guangzhouensis_DSM_102975_03	Thiotrichales	*Francisella*
Francisella_guangzhouensis_DSM_102975_04	Thiotrichales	*Francisella*
Francisella_hispaniensis_DSM_22475_01	Thiotrichales	*Francisella*

Francisella_hispaniensis_DSM_22475_02	Thiotrichales	*Francisella*
Francisella_hispaniensis_DSM_22475_03	Thiotrichales	*Francisella*
Francisella_hispaniensis_DSM_22475_04	Thiotrichales	*Francisella*
Francisella_naotunensis_ssp_orientalis_DSM_21254_01	Thiotrichales	*Francisella*
Francisella_naotunensis_ssp_orientalis_DSM_21254_02	Thiotrichales	*Francisella*
Francisella_naotunensis_ssp_orientalis_DSM_21254_03	Thiotrichales	*Francisella*
Francisella_naotunensis_ssp_orientalis_DSM_21254_04	Thiotrichales	*Francisella*
Francisella_tularensis_novicida_Ft26_01	Thiotrichales	*Francisella*
Francisella_tularensis_novicida_Ft26_02	Thiotrichales	*Francisella*
Francisella_tularensis_novicida_Ft26_03	Thiotrichales	*Francisella*
Francisella_tularensis_novicida_Ft26_04	Thiotrichales	*Francisella*
Francisella_tularensis_ssp_mediasiatica_Ft31_01	Thiotrichales	*Francisella*
Francisella_tularensis_ssp_mediasiatica_Ft31_02	Thiotrichales	*Francisella*
Francisella_tularensis_ssp_mediasiatica_Ft31_03	Thiotrichales	*Francisella*
Francisella_tularensis_ssp_mediasiatica_Ft31_04	Thiotrichales	*Francisella*
Francisella_tularensis_subsp_holarctica_A466_1_01	Thiotrichales	*Francisella*
Francisella_tularensis_subsp_holarctica_A466_1_02	Thiotrichales	*Francisella*
Francisella_tularensis_subsp_holarctica_A466_1_03	Thiotrichales	*Francisella*
Francisella_tularensis_subsp_holarctica_A466_1_04	Thiotrichales	*Francisella*
Inquilinus_limosus_DSM_16000_01	Rhodospirillales	*Inquilinus*
Inquilinus_limosus_DSM_16000_02	Rhodospirillales	*Inquilinus*
Inquilinus_limosus_DSM_16000_03	Rhodospirillales	*Inquilinus*
Inquilinus_limosus_DSM_16000_04	Rhodospirillales	*Inquilinus*
Klebsiella_oxytoca_ATCC_13182_01	Enterobacteriales	*Klebsiella*
Klebsiella_oxytoca_ATCC_13182_02	Enterobacteriales	*Klebsiella*
Klebsiella_pneumoniae_ssp_ozeanae_DSM_68_01	Enterobacteriales	*Klebsiella*
Klebsiella_pneumoniae_ssp_ozeanae_DSM_68_02	Enterobacteriales	*Klebsiella*
Kocuria_palustris_RRLK06_02_01	Actinomycetales	*Kocuria*
Kocuria_palustris_RRLK06_02_02	Actinomycetales	*Kocuria*
Kocuria_palustris_RRLK06_02_03	Actinomycetales	*Kocuria*

Kocuria_palustris_RRLK06_02_04	Actinomycetales	*Kocuria*
Lysinibacillus_fusiformis_B207_01	Bacillales	*Lysinibacillus*
Lysinibacillus_fusiformis_B207_02	Bacillales	*Lysinibacillus*
Lysinibacillus_fusiformis_B207_03	Bacillales	*Lysinibacillus*
Lysinibacillus_fusiformis_B207_04	Bacillales	*Lysinibacillus*
Lysinibacillus_sphaericus_DSM_396_01	Bacillales	*Lysinibacillus*
Lysinibacillus_sphaericus_DSM_396_02	Bacillales	*Lysinibacillus*
Micrococcus_luteus_RRLK07_01	Actinomycetales	*Micrococcus*
Micrococcus_luteus_RRLK07_02	Actinomycetales	*Micrococcus*
Micrococcus_luteus_RRLK07_03	Actinomycetales	*Micrococcus*
Micrococcus_luteus_RRLK07_04	Actinomycetales	*Micrococcus*
Ochrobactrum_anthropi_DSM_20150_01	Rhizobiales	*Ochrobactrum*
Ochrobactrum_anthropi_DSM_20150_02	Rhizobiales	*Ochrobactrum*
Ochrobactrum_anthropi_DSM_20150_03	Rhizobiales	*Ochrobactrum*
Ochrobactrum_anthropi_DSM_20150_04	Rhizobiales	*Ochrobactrum*
Oligella_urethralis_A691_2_01	Burkholderiales	*Oligella*
Oligella_urethralis_A691_2_02	Burkholderiales	*Oligella*
Paenibacillus_alvei_B210_01	Bacillales	*Paenibacillus*
Paenibacillus_alvei_B210_02	Bacillales	*Paenibacillus*
Paenibacillus_alvei_B210_03	Bacillales	*Paenibacillus*
Paenibacillus_alvei_B210_04	Bacillales	*Paenibacillus*
Paenibacillus_polymyxa_DSM_365_01	Bacillales	*Paenibacillus*
Paenibacillus_polymyxa_DSM_365_02	Bacillales	*Paenibacillus*
Paenibacillus_polymyxa_DSM_365_03	Bacillales	*Paenibacillus*
Paenibacillus_polymyxa_DSM_365_04	Bacillales	*Paenibacillus*
Pandoraea_apista_DSM_16535_01	Burkholderiales	*Pandoraea*
Pandoraea_apista_DSM_16535_02	Burkholderiales	*Pandoraea*
Pandoraea_apista_DSM_16535_03	Burkholderiales	*Pandoraea*
Pandoraea_apista_DSM_16535_04	Burkholderiales	*Pandoraea*
Pandoraea_pnomenusa_DSM_16536_01	Burkholderiales	*Pandoraea*

Pandoraea_pnomenusa_DSM_16536_02	Burkholderiales	*Pandoraea*
Pandoraea_pnomenusa_DSM_16536_03	Burkholderiales	*Pandoraea*
Pandoraea_pnomenusa_DSM_16536_04	Burkholderiales	*Pandoraea*
Pandoraea_pulmonicola_DSM_16583_01	Burkholderiales	*Pandoraea*
Pandoraea_pulmonicola_DSM_16583_02	Burkholderiales	*Pandoraea*
Pandoraea_pulmonicola_DSM_16583_03	Burkholderiales	*Pandoraea*
Pandoraea_pulmonicola_DSM_16583_04	Burkholderiales	*Pandoraea*
Pantoea_agglomerans_ATCC_27988_01	Enterobacteriales	*Pantoea*
Pantoea_agglomerans_ATCC_27988_02	Enterobacteriales	*Pantoea*
Paracoccus_yeei_RRLK05_01_01	Rhodobacterales	*Paracoccus*
Paracoccus_yeei_RRLK05_01_02	Rhodobacterales	*Paracoccus*
Paracoccus_yeei_RRLK05_01_03	Rhodobacterales	*Paracoccus*
Paracoccus_yeei_RRLK05_01_04	Rhodobacterales	*Paracoccus*
Proteus_inconstans_ATCC_25827_01	Enterobacteriales	*Proteus*
Proteus_inconstans_ATCC_25827_02	Enterobacteriales	*Proteus*
Proteus_mirabilis_SM_788_01	Enterobacteriales	*Proteus*
Proteus_mirabilis_SM_788_02	Enterobacteriales	*Proteus*
Proteus_morganii_DSM_30117_01	Enterobacteriales	*Proteus*
Proteus_morganii_DSM_30117_02	Enterobacteriales	*Proteus*
Proteus_vulgaris_ATCC_33420_01	Enterobacteriales	*Proteus*
Proteus_vulgaris_ATCC_33420_02	Enterobacteriales	*Proteus*
Pseudomonas_aeruginosa_ATCC_27853_01	Pseudomonadales	*Pseudomonas*
Pseudomonas_aeruginosa_ATCC_27853_02	Pseudomonadales	*Pseudomonas*
Pseudomonas_aeruginosa_ATCC_27853_03	Pseudomonadales	*Pseudomonas*
Pseudomonas_aeruginosa_ATCC_27853_04	Pseudomonadales	*Pseudomonas*
Pseudomonas_chlororaphis_ATCC_17809_01	Pseudomonadales	*Pseudomonas*
Pseudomonas_chlororaphis_ATCC_17809_02	Pseudomonadales	*Pseudomonas*
Pseudomonas_chlororaphis_ATCC_17809_03	Pseudomonadales	*Pseudomonas*
Pseudomonas_chlororaphis_ATCC_17809_04	Pseudomonadales	*Pseudomonas*
Ralstonia_insidiosa_DSM_17714_01	Burkholderiales	*Ralstonia*

Ralstonia_insidiosa_DSM_17714_02	Burkholderiales	*Ralstonia*
Ralstonia_insidiosa_DSM_17714_03	Burkholderiales	*Ralstonia*
Ralstonia_insidiosa_DSM_17714_04	Burkholderiales	*Ralstonia*
Ralstonia_mannitolilytica_DSM_17512_01	Burkholderiales	*Ralstonia*
Ralstonia_mannitolilytica_DSM_17512_02	Burkholderiales	*Ralstonia*
Ralstonia_mannitolilytica_DSM_17512_03	Burkholderiales	*Ralstonia*
Ralstonia_mannitolilytica_DSM_17512_04	Burkholderiales	*Ralstonia*
Ralstonia_pickettii_DSM_6297_01	Burkholderiales	*Ralstonia*
Ralstonia_pickettii_DSM_6297_02	Burkholderiales	*Ralstonia*
Ralstonia_pickettii_DSM_6297_03	Burkholderiales	*Ralstonia*
Ralstonia_pickettii_DSM_6297_04	Burkholderiales	*Ralstonia*
Salmonella_enterica_serovar_minnesota_SF_1111_01	Enterobacteriales	*Salmonella*
Salmonella_enterica_serovar_minnesota_SF_1111_02	Enterobacteriales	*Salmonella*
Salmonella_enteritidis_LT21_01	Enterobacteriales	*Salmonella*
Salmonella_enteritidis_LT21_02	Enterobacteriales	*Salmonella*
Salmonella_hadar_01	Enterobacteriales	*Salmonella*
Salmonella_hadar_02	Enterobacteriales	*Salmonella*
Salmonella_typhimurium_SH_9178_01	Enterobacteriales	*Salmonella*
Salmonella_typhimurium_SH_9178_02	Enterobacteriales	*Salmonella*
Serratia_grimesii_DSM_30063_01	Enterobacteriales	*Salmonella*
Serratia_grimesii_DSM_30063_02	Enterobacteriales	*Salmonella*
Serratia_marcescens_DSM_30121_01	Enterobacteriales	*Salmonella*
Serratia_marcescens_DSM_30121_02	Enterobacteriales	*Salmonella*
Shigella_boydii_01	Enterobacteriales	*Salmonella*
Shigella_boydii_02	Enterobacteriales	*Salmonella*
Shigella_flexneri_3A_01	Enterobacteriales	*Salmonella*
Shigella_flexneri_3A_02	Enterobacteriales	*Salmonella*
Staphylococcus_aureus_DSM_20231_01	Bacillales	*Staphylococcus*
Staphylococcus_aureus_DSM_20231_02	Bacillales	*Staphylococcus*
Staphylococcus_aureus_DSM_20231_03	Bacillales	*Staphylococcus*

Staphylococcus_aureus_DSM_20231_04	Bacillales	*Staphylococcus*
Staphylococcus_epidermidis_DSM_1798_01	Bacillales	*Staphylococcus*
Staphylococcus_epidermidis_DSM_1798_02	Bacillales	*Staphylococcus*
Staphylococcus_epidermidis_DSM_1798_03	Bacillales	*Staphylococcus*
Staphylococcus_epidermidis_DSM_1798_04	Bacillales	*Staphylococcus*
Staphylococcus_hominis_RRLK01_03_01	Bacillales	*Staphylococcus*
Staphylococcus_hominis_RRLK01_03_02	Bacillales	*Staphylococcus*
Staphylococcus_hominis_RRLK01_03_03	Bacillales	*Staphylococcus*
Staphylococcus_hominis_RRLK01_03_04	Bacillales	*Staphylococcus*
Stenotrophomonas_maltophilia_Sm_36_01	Xanthomonadales	*Stenotrophomonas*
Stenotrophomonas_maltophilia_Sm_36_02	Xanthomonadales	*Stenotrophomonas*
Stenotrophomonas_maltophilia_Sm_36_03	Xanthomonadales	*Stenotrophomonas*
Stenotrophomonas_rhizophila_RRLK03_01_01	Xanthomonadales	*Stenotrophomonas*
Stenotrophomonas_rhizophila_RRLK03_01_02	Xanthomonadales	*Stenotrophomonas*
Stenotrophomonas_rhizophila_RRLK03_01_03	Xanthomonadales	*Stenotrophomonas*
Stenotrophomonas_rhizophila_RRLK03_01_04	Xanthomonadales	*Stenotrophomonas*
Streptococcus_pyogenes_DSM_20565_01	Lactobacillales	*Streptococcus*
Streptococcus_pyogenes_DSM_20565_02	Lactobacillales	*Streptococcus*
Streptococcus_pyogenes_DSM_20565_03	Lactobacillales	*Streptococcus*
Streptococcus_pyogenes_DSM_20565_04	Lactobacillales	*Streptococcus*
Vibrio_alginolyticus_DSM_2171_01	Enterobacteriales	*Vibrio*
Vibrio_cholerae_A220_01	Enterobacteriales	*Vibrio*
Vibrio_cholerae_A220_02	Enterobacteriales	*Vibrio*
Vibrio_cholerae_A220_03	Enterobacteriales	*Vibrio*
Vibrio_cholerae_A220_04	Enterobacteriales	*Vibrio*
Vibrio_diazotrophicus_174_2_01	Enterobacteriales	*Vibrio*
Vibrio_mimicus_A177_1_01	Enterobacteriales	*Vibrio*
Vibrio_parahaemolyticus_177_2_01	Enterobacteriales	*Vibrio*
Vibrio_vulnificus_A177_3_02	Enterobacteriales	*Vibrio*
Xenophilus_sp_A395_01	Burkholderiales	*Xenophilus*

Xenophilus_sp_A395_02	Burkholderiales	*Xenophilus*
Yersinia_aldovae_DSM_18303_01	Enterobacteriales	*Yersinia*
Yersinia_aldovae_DSM_18303_02	Enterobacteriales	*Yersinia*
Yersinia_aldovae_DSM_18303_03	Enterobacteriales	*Yersinia*
Yersinia_aleksiciae_DSM_14987_01	Enterobacteriales	*Yersinia*
Yersinia_aleksiciae_DSM_14987_02	Enterobacteriales	*Yersinia*
Yersinia_aleksiciae_DSM_14987_03	Enterobacteriales	*Yersinia*
Yersinia_bercovieri_DSM_18528_01	Enterobacteriales	*Yersinia*
Yersinia_bercovieri_DSM_18528_02	Enterobacteriales	*Yersinia*
Yersinia_bercovieri_DSM_18528_03	Enterobacteriales	*Yersinia*
Yersinia_enterocolitica_DSM_9676_01	Enterobacteriales	*Yersinia*
Yersinia_enterocolitica_DSM_9676_02	Enterobacteriales	*Yersinia*
Yersinia_enterocolitica_DSM_9676_03	Enterobacteriales	*Yersinia*
Yersinia_entomophaga_DSM_22339_01	Enterobacteriales	*Yersinia*
Yersinia_entomophaga_DSM_22339_02	Enterobacteriales	*Yersinia*
Yersinia_entomophaga_DSM_22339_03	Enterobacteriales	*Yersinia*
Yersinia_entomophaga_DSM_22339_04	Enterobacteriales	*Yersinia*
Yersinia_frederiksenii_DSM_18490_01	Enterobacteriales	*Yersinia*
Yersinia_frederiksenii_DSM_18490_02	Enterobacteriales	*Yersinia*
Yersinia_frederiksenii_DSM_18490_03	Enterobacteriales	*Yersinia*
Yersinia_intermedia_DSM_18517_01	Enterobacteriales	*Yersinia*
Yersinia_intermedia_DSM_18517_02	Enterobacteriales	*Yersinia*
Yersinia_intermedia_DSM_18517_03	Enterobacteriales	*Yersinia*
Yersinia_kristensenii_DSM_18543_01	Enterobacteriales	*Yersinia*
Yersinia_kristensenii_DSM_18543_02	Enterobacteriales	*Yersinia*
Yersinia_kristensenii_DSM_18543_03	Enterobacteriales	*Yersinia*
Yersinia_massiliensis_DSM_21859_01	Enterobacteriales	*Yersinia*
Yersinia_massiliensis_DSM_21859_02	Enterobacteriales	*Yersinia*
Yersinia_massiliensis_DSM_21859_03	Enterobacteriales	*Yersinia*
Yersinia_massiliensis_DSM_21859_04	Enterobacteriales	*Yersinia*

Yersinia_mollaretii_DSM_18520_01	Enterobacteriales	*Yersinia*
Yersinia_mollaretii_DSM_18520_02	Enterobacteriales	*Yersinia*
Yersinia_mollaretii_DSM_18520_03	Enterobacteriales	*Yersinia*
Yersinia_nurmii_DSM_22296_01	Enterobacteriales	*Yersinia*
Yersinia_nurmii_DSM_22296_02	Enterobacteriales	*Yersinia*
Yersinia_nurmii_DSM_22296_03	Enterobacteriales	*Yersinia*
Yersinia_nurmii_DSM_22296_04	Enterobacteriales	*Yersinia*
Yersinia_pekkanenii_DSM_22769_01	Enterobacteriales	*Yersinia*
Yersinia_pekkanenii_DSM_22769_02	Enterobacteriales	*Yersinia*
Yersinia_pekkanenii_DSM_22769_03	Enterobacteriales	*Yersinia*
Yersinia_pekkanenii_DSM_22769_04	Enterobacteriales	*Yersinia*
Yersinia_pestis_10329_01	Enterobacteriales	*Yersinia*
Yersinia_pestis_10329_04	Enterobacteriales	*Yersinia*
Yersinia_pseudotuberculosis_DSM_8992_01	Enterobacteriales	*Yersinia*
Yersinia_pseudotuberculosis_DSM_8992_02	Enterobacteriales	*Yersinia*
Yersinia_pseudotuberculosis_DSM_8992_03	Enterobacteriales	*Yersinia*
Yersinia_rohdei_DSM_18270_01	Enterobacteriales	*Yersinia*
Yersinia_rohdei_DSM_18270_02	Enterobacteriales	*Yersinia*
Yersinia_rohdei_DSM_18270_03	Enterobacteriales	*Yersinia*
Yersinia_ruckeri_DSM_18506_01	Enterobacteriales	*Yersinia*
Yersinia_ruckeri_DSM_18506_02	Enterobacteriales	*Yersinia*
Yersinia_ruckeri_DSM_18506_03	Enterobacteriales	*Yersinia*
Yersinia_similis_DSM_18211_01	Enterobacteriales	*Yersinia*
Yersinia_similis_DSM_18211_02	Enterobacteriales	*Yersinia*
Yersinia_similis_DSM_18211_03	Enterobacteriales	*Yersinia*
Yersinia_similis_DSM_18211_04	Enterobacteriales	*Yersinia*
Yersinia_wautersii_DSM_27350_01	Enterobacteriales	*Yersinia*
Yersinia_wautersii_DSM_27350_02	Enterobacteriales	*Yersinia*
Yersinia_wautersii_DSM_27350_03	Enterobacteriales	*Yersinia*
Yersinia_wautersii_DSM_27350_04	Enterobacteriales	*Yersinia*

TABLE S2. SUMMARY TABLE OF STUDIED BACTERIAL STRAINS (BSNB)

**Names**	**ID**	**BSNB**	**Order**	**Genus**
Bacillus_cereus	GTC2112	BSNB_0016	Bacillales	*Bacillus*
Brevibacillus_sp	GCYPB01	BSNB_0034	Bacillales	*Brevibacillus*
Bacillus_sp	GTC2802	BSNB_0053	Bacillales	*Bacillus*
Serratia_sp	CN1	BSNB_0139	Enterobacteriales	*Serratia*
Serratia_marcescens	CN4	BSNB_0142	Enterobacteriales	*Serratia*
Burkholderia_seminalis	CN5A	BSNB_0143	Burkholderiales	*Burkholderia*
Pseudomonas_beteli	CN12	BSNB_0150	Pseudomonadales	*Pseudomonas*
Serratia_marcescens	CN14	BSNB_0152	Enterobacteriales	*Serratia*
Achromobacter_sp	CN15	BSNB_0153	Burkholderiales	*Achromobacter*
Burkholderia_sp	CN20	BSNB_0158	Burkholderiales	*Burkholderia*
Serratia_marcescens	CN27	BSNB_0165	Enterobacteriales	*Serratia*
Burkholderia_sp	CN28	BSNB_0166	Burkholderiales	*Burkholderia*
Burkholderia_pyrrocinia	CN32	BSNB_0170	Burkholderiales	*Burkholderia*
Burkholderia_sp	CN33	BSNB_0171	Burkholderiales	*Burkholderia*
Burkholderia_cepacia	CN34	BSNB_0172	Burkholderiales	*Burkholderia*
Burkholderia_sp	CN38	BSNB_0178	Burkholderiales	*Burkholderia*
Lactococcus_garvieae	CN39	BSNB_0179	Lactobacillales	*Lactococcus*
Lactococcus_garvieae	CN40	BSNB_0180	Lactobacillales	*Lactococcus*
Burkholderia_arboris	CN41	BSNB_0181	Burkholderiales	*Burkholderia*
Enterobacter_tabaci	CN42A	BSNB_0182	Enterobacteriales	*Enterobacter*
Enterobacter_tabaci	CN51	BSNB_0191	Enterobacteriales	*Enterobacter*
Enterobacter_tabaci	CN52	BSNB_0192	Enterobacteriales	*Enterobacter*
Pseudomonas_sp	CN54	BSNB_0194	Pseudomonadales	*Pseudomonas*
Kitasatospora_sp	CN61	BSNB_0202	Actinomycetales	*Kitasatospora*
Bacillus_cereus	CN89	BSNB_0230	Bacillales	*Bacillus*
Bacillus_cereus	CN91	BSNB_0232	Bacillales	*Bacillus*
Lysinibacillus_xylanilyticus	CN92	BSNB_0233	Bacillales	*Lysinibacillus*
Streptomyces_sp	CN93	BSNB_0234	Actinomycetales	*Streptomyces*

Bacillus_firmus	CN94	BSNB_0235	Bacillales	*Bacillus*
Bacillus_sp	CN95	BSNB_0236	Bacillales	*Bacillus*
Lysinibacillus_sp	CN98	BSNB_0239	Bacillales	*Lysinibacillus*
Enterobacter_tabaci	CN99	BSNB_0240	Enterobacteriales	*Enterobacter*
Serratia_marcescens	CN101	BSNB_0242	Enterobacteriales	*Serratia*
Lysinibacillus_xylanilyticus	CN104	BSNB_0245	Bacillales	*Lysinibacillus*
Burkholderia_sp	CN105	BSNB_0246	Burkholderiales	*Burkholderia*
Streptomyces_malaysiense	CN115	BSNB_0256	Actinomycetales	*Streptomyces*
Paraburkholderia_tropica	A1F1 2	BSNB_0493	Burkholderiales	*Paraburkholderia*
Klebsiella_michiganensis	A1F1 3	BSNB_0494	Enterobacteriales	*Klebsiella*
Enterobacter_asburiae	A2F_1 1.1	BSNB_0517	Enterobacteriales	*Enterobacter*
Burkholderia_tropica	A2F_1 3	BSNB_0519	Burkholderiales	*Burkholderia*
Burkholderia_sp	A2F_1 5.2	BSNB_0521	Burkholderiales	*Burkholderia*
Burkholderia_tropica	A2F11 4	BSNB_0547	Burkholderiales	*Burkholderia*
Bacillus_thuringiensis	A3F_1 2	BSNB_0561	Bacillales	*Bacillus*
*Paraburkholderia_tropica_*	A3F_1 3	BSNB_0562	Burkholderiales	*Paraburkholderia*
*Paraburkholderia_tropica_*	A3F_1 6	BSNB_0565	Burkholderiales	*Paraburkholderia*
Bacillus_subtilis	A3F1 1	BSNB_0566	Bacillales	*Bacillus*
Bacillus_subtilis	A3F1 8.2	BSNB_0567	Bacillales	*Bacillus*
Bacillus_subtilis	A3F1 3	BSNB_0568	Bacillales	*Bacillus*
Pantoea_dispersa	A3F1 4.1	BSNB_0569	Enterobacteriales	*Pantoea*
Burkholderia_tropica	A3F1 5	BSNB_0570	Burkholderiales	*Burkholderia*
Burkholderia_tropica	A3F1 7	BSNB_0572	Burkholderiales	*Burkholderia*
Bacillus_subtilis	A3F1 8.111	BSNB_0573	Bacillales	*Bacillus*
Bacillus_subtilis	A3F1 2	BSNB_0576	Bacillales	*Bacillus*
Klebsiella_variicola	A4F1 1	BSNB_0591	Enterobacteriales	*Klebsiella*
Klebsiella_variicola	A4F1 2	BSNB_0592	Enterobacteriales	*Klebsiella*
Burkholderia_tropica	A4F1 3	BSNB_0593	Burkholderiales	*Burkholderia*
Klebsiella_variicola	A4F1 4	BSNB_0594	Enterobacteriales	*Klebsiella*
Klebsiella_variicola	A4F1 5	BSNB_0595	Enterobacteriales	*Klebsiella*

Burkholderia_plantarii	A4F1 6	BSNB_0596	Burkholderiales	*Burkholderia*
Burkholderia_plantarii	A4F1 7	BSNB_0597	Burkholderiales	*Burkholderia*
Klebsiella_variicola	A4F1 9	BSNB_0598	Enterobacteriales	*Klebsiella*
Burkholderia_sp	A5F_1 2	BSNB_0616	Burkholderiales	*Burkholderia*
Burkholderia_arvi	A5F_1 4	BSNB_0617	Burkholderiales	*Burkholderia*
Bacillus_cereus	A5F_1 6	BSNB_0618	Bacillales	*Bacillus*
Burkholderia_sp	A5F_1 3	BSNB_0619	Burkholderiales	*Burkholderia*
Burkholderia_tropica	A5F1 8	BSNB_0626	Burkholderiales	*Burkholderia*
Burkholderia_tropica	A5F10 2	BSNB_0630	Burkholderiales	*Burkholderia*
Burkholderia_tropica	A5F10 4	BSNB_0632	Burkholderiales	*Burkholderia*
Paraburkholderia_guartelaensis	A5F10 7	BSNB_0635	Burkholderiales	*Paraburkholderia*
Burkholderia_sp	A5F10 8	BSNB_0636	Burkholderiales	*Burkholderia*
Burkholderia_tropica	A5F10 11	BSNB_0639	Burkholderiales	*Burkholderia*
Burkholderia_oxyphila	A5F10 12	BSNB_0640	Burkholderiales	*Burkholderia*
Paenibacillus_glucanolyticus	A6F_1 1	BSNB_0643	Bacillales	*Paenibacillus*
Bacillus_sp	A6F1 4	BSNB_0648	Bacillales	*Bacillus*
Burkholderia_tropica	A6F16 4	BSNB_0656	Burkholderiales	*Burkholderia*
Burkholderia_tropica	A6F16 6	BSNB_0658	Burkholderiales	*Burkholderia*
Bacillus_subtilis	A6F16 7.1	BSNB_0659	Bacillales	*Bacillus*
Burkholderia_tropica	A6F16 8	BSNB_0661	Burkholderiales	*Burkholderia*
Burkholderia_tropica	A6F16 10	BSNB_0663	Burkholderiales	*Burkholderia*
Enterobacter_amnigenus	A7F_1 1	BSNB_0669	Enterobacteriales	*Enterobacter*
Pantoea_stewartii	A7F_1 3	BSNB_0671	Enterobacteriales	*Pantoea*
Pantoea_stewartii	A7F_1 4	BSNB_0672	Enterobacteriales	*Pantoea*
Enterobacter_mori	A7F_1 7	BSNB_0675	Enterobacteriales	*Enterobacter*
Bacillus_subtilis	A7F1 5.1	BSNB_0683	Bacillales	*Bacillus*
Bacillus_subtilis	A7F1 8	BSNB_0685	Bacillales	*Bacillus*
Stenotrophomonas_maltophilia	A8F_1 1	BSNB_0704	Xanthomonadales	*Stenotrophomonas*
Enterobacter_mori	A8F_1 3	BSNB_0706	Enterobacteriales	*Enterobacter*
Enterobacter_mori	A8F_1 5.1	BSNB_0708	Enterobacteriales	*Enterobacter*

Enterobacter_mori	A8F_1 6.1	BSNB_0709	Enterobacteriales	*Enterobacter*
Bacillus_subtilis	A8F_1 8	BSNB_0712	Bacillales	*Bacillus*
Bacillus_subtilis	A8F1 1	BSNB_0716	Bacillales	*Bacillus*
Luteibacter_yeojuensis	A8F1 6.1	BSNB_0719	Xanthomonadales	*Luteibacter*
Luteibacter_yeojuensis	A8F1 7	BSNB_0721	Xanthomonadales	*Luteibacter*
Bacillus_subtilis	A8F1 8	BSNB_0722	Bacillales	*Bacillus*
Enterobacter_mori	A8F11 1	BSNB_0723	Enterobacteriales	*Enterobacter*
Enterobacter_mori	A8F11 3	BSNB_0725	Enterobacteriales	*Enterobacter*
Enterobacter_mori	A8F11 4	BSNB_0726	Enterobacteriales	*Enterobacter*
Enterobacter_mori	A8F11 5	BSNB_0727	Enterobacteriales	*Enterobacter*
Bacillus_subtilis	A8F11 8	BSNB_0730	Bacillales	*Bacillus*
Bacillus_subtilis	C1F9 10	BSNB_0762	Bacillales	*Bacillus*
Bacillus_subtilis	P1F1 1	BSNB_0803	Bacillales	*Bacillus*
Bacillus_subtilis	P1F1 2	BSNB_0804	Bacillales	*Bacillus*
Methylobacterium_mesophilicum	P1F1 3	BSNB_0805	Rhizobiales	*Methylobacterium*
Bacillus_subtilis	P1F5 8	BSNB_0816	Bacillales	*Bacillus*
Bacillus_subtilis	P1F5 9	BSNB_0817	Bacillales	*Bacillus*
Bacillus_subtilis	W2F_2 1	BSNB_0955	Bacillales	*Bacillus*
Bacillus_subtilis	W2F_2 3	BSNB_0956	Bacillales	*Bacillus*
Bacillus_subtilis	W2F_2 9	BSNB_0959	Bacillales	*Bacillus*
Acidisoma_sp	W3F_3 8	BSNB_0978	Rhodospirillales	*Acidosoma*
Burkholderia_tropica	AC A2 F11 9.1B	BSNB_1020	Burkholderiales	*Burkholderia*
Methylobacterium_populi	VECD13D	BSNB_1022	Rhizobiales	*Methylobacterium*
Curtobacterium_sp	VECD14A	BSNB_1023	Actinomycetales	*Curtobacterium*
Pantoea_dispersa	VECD14B	BSNB_1024	Enterobacteriales	*Pantoea*
Bacillus_megaterium	VECD14C	BSNB_1025	Bacillales	*Bacillus*
Methylobacterium_sp	VECD14G	BSNB_1026	Rhizobiales	*Methylobacterium*
Asaia_sp	VECD15A	BSNB_1027	Rhodospirillales	*Asaia*
Pseudomonas_sp	ST5C	BSNB_1030	Pseudomonadales	*Pseudomonas*

Arthrobacter_sp	ST8C	BSNB_1032	Actinomycetales	*Arthrobacter*
Pantoea_agglomerans	ST16C	BSNB_1034	Enterobacteriales	*Pantoea*
Methylobacterium_mesophilicum	LR_FP43	BSNB_1116	Rhizobiales	*Methylobacterium*
Methylobacterium_mesophilicum	LR_FP51	BSNB_1123	Rhizobiales	*Methylobacterium*
Methylobacterium_mesophilicum	LR_FP67	BSNB_1136	Rhizobiales	*Methylobacterium*
Methylobacterium_mesophilicum	LR_FP68	BSNB_1137	Rhizobiales	*Methylobacterium*
Methylobacterium_mesophilicum	LR_FP74	BSNB_1141	Rhizobiales	*Methylobacterium*
Rhodococcus_kroppenstedtii	LR_FP135	BSNB_1179	Actinomycetales	*Rhodococcus*
Rhodococcus_kroppenstedtii	LR_FP150	BSNB_1190	Actinomycetales	*Rhodococcus*
Microbacterium_testaceum	LR_FP151	BSNB_1191	Actinomycetales	*Microbacterium*
Microbacterium_testaceum	LR_FP152	BSNB_1192	Actinomycetales	*Microbacterium*
Bacillus_subtilis	LR_FP171	BSNB_1207	Bacillales	*Bacillus*
Bacillus_subtilis	LR_FP172	BSNB_1208	Bacillales	*Bacillus*
Bacillus_subtilis	LR_FP174	BSNB_1209	Bacillales	*Bacillus*
Bacillus_subtilis	LR_FP175	BSNB_1210	Bacillales	*Bacillus*
Rhodococcus_cerastii	LR_EL13	BSNB_1220	Actinomycetales	*Rhodococcus*
Bacillus_subtilis	LR_EL109	BSNB_1238	Bacillales	*Bacillus*
Rhodococcus_cerastii	LR_EL133	BSNB_1246	Actinomycetales	*Rhodococcus*
Klebsiella_michiganensis	A1F2A1	NO_BSNB	Enterobacteriales	*Klebsiella*
Klebsiella_michiganensis	A4F1_12	NO_BSNB	Enterobacteriales	*Klebsiella*
Methylobacterium_mesophilicum	LRFP_069	NO_BSNB	Rhizobiales	*Methylobacterium*

TABLE S3. SUMMARY TABLE OF STUDIED FUNGAL STRAINS (BSNB)

**Names**	**ID**	**BSNB**	**Order**	**Genus**
Epicoccum_nigrum	GTG01	BSNB_0001	Pleosporales	*Epicoccum*
Colletotrichum_gloeosporioides	GTC2001	BSNB_0002	Glomerellales	*Colletotrichum*
Pestalotiopsis_sp	GTC2102	BSNB_0006	Xylariales	*Pestalotiopsis*
Pestalotiopsis_sp	GTC2104	BSNB_0008	Xylariales	*Pestalotiopsis*
Chaetomium_globosum	GTC2114	BSNB_0018	Sordariales	*Chaetomium*
Diaporthe_phaseolorum	GTC2201	BSNB_0020	Diaporthales	*Diaporthe*
Xylaria_cubensis	GPP02	BSNB_0022	Xylariales	*Xylaria*
Mycoleptodiscus_sp	GTC2304	BSNB_0024	Magnaporthales	*Mycoleptodiscus*
Colletotrichum_siamense	GTC2303	BSNB_0025	Glomerellales	*Colletotrichum*
Mycoleptodiscus_sp	GTC2304	BSNB_0026	Magnaporthales	*Mycoleptodiscus*
Colletotrichum_gloeosporioides	GTC2401	BSNB_0029	Glomerellales	*Colletotrichum*
Colletotrichum_boninense	GTC2502	BSNB_0032	Glomerellales	*Colletotrichum*
Diaporthe_phaseolorum	GTC2503	BSNB_0033	Diaporthales	*Diaporthe*
Colletotrichum_boninense	GTC2601	BSNB_0035	Glomerellales	*Colletotrichum*
Xylaria_cubensis	GTC2604	BSNB_0038	Xylariales	*Xylaria*
Colletotrichum_gloeosporioides	GTC2605	BSNB_0039	Glomerellales	*Colletotrichum*
Xylaria_sp	GTC2702	BSNB_0041	Xylariales	*Xylaria*
Guignardia_mangiferae	GTC2703	BSNB_0042	Botryosphaeriales	*Guignardia*
Pestalotiopsis_sp	GTC2704	BSNB_0043	Xylariales	*Pestalotiopsis*
Diaporthe_sp	GTC2705	BSNB_0044	Diaporthales	*Diaporthe*
Diaporthe_sp	GTC2708	BSNB_0047	Diaporthales	*Diaporthe*
Xylariales_sp	GTC2709	BSNB_0048	Xylariales	*Xylaria*
Xylariales_sp	GTC2711	BSNB_0050	Xylariales	*Xylaria*
Penicillium_sp	GVL02	BSNB_0051	Eurotiales	*Penicillium*
Phomopsis_sp	GTC2801	BSNB_0052	Diaporthales	*Phomopsis*
Phomopsis_phyllanthicola	GTC2804	BSNB_0055	Diaporthales	*Phomopsis*
Xylaria_cubensis	GTC2805	BSNB_0056	Xylariales	*Xylaria*
Phomopsis_sp	GTC2806	BSNB_0057	Diaporthales	*Phomopsis*

Diaporthe_phaseolorum	GTC2808	BSNB_0059	Diaporthales	*Diaporthe*
Diaporthe_phaseolorum	GTC2809	BSNB_0060	Diaporthales	*Diaporthe*
Phomopsis_sp	GTC2812	BSNB_0063	Diaporthales	*Phomopsis*
Phomopsis_sp	GTC2820	BSNB_0071	Diaporthales	*Phomopsis*
Chaetomium_sp	GTC2901	BSNB_0074	Sordariales	*Chaetomium*
Xylaria_sp	GTC2902	BSNB_0075	Xylariales	*Xylaria*
Acremonium_sp	GTC2903	BSNB_0076	Hypocreales	*Acremonium*
Pestalotiopsis_microspora	GTC2904	BSNB_0077	Xylariales	*Pestalotiopsis*
Chaetomium_sp	GTC2905	BSNB_0078	Sordariales	*Chaetomium*
Colletotrichum_gloeosporioides	GTC3003	BSNB_0085	Glomerellales	*Colletotrichum*
Guignardia_mangiferae	GSS01	BSNB_0087	Botryosphaeriales	*Guignardia*
Xylaria_cubensis	GSS02	BSNB_0088	Xylariales	*Xylaria*
Xylaria_cubensis	GSS04	BSNB_0089	Xylariales	*Xylaria*
Xylaria_cubensis	GSS05	BSNB_0090	Xylariales	*Xylaria*
Xylaria_cubensis	GSS06	BSNB_0091	Xylariales	*Xylaria*
Colletotrichum_sp	GSS08	BSNB_0093	Glomerellales	*Colletotrichum*
Diaporthe_phaseolorum	GSS09	BSNB_0094	Diaporthales	*Diaporthe*
Diaporthe_sp	GSS11	BSNB_0096	Diaporthales	*Diaporthe*
Colletotrichum_sp	GSS13	BSNB_0098	Glomerellales	*Colletotrichum*
Diaporthe_sp	GSS15	BSNB_0100	Diaporthales	*Diaporthe*
Pestalotiopsis_sp	GCI01	BSNB_0102	Xylariales	*Pestalotiopsis*
Xylaria_cubensis	GCI02	BSNB_0103	Xylariales	*Xylaria*
Xylaria_cubensis	LD1.6	BSNB_0106	Xylariales	*Xylaria*
Muscodor_sp	LD1.5	BSNB_0107	Xylariales	*Muscodor*
Guignardia_mangiferae	LD2.13	BSNB_0115	Botryosphaeriales	*Guignardia*
Colletotrichum_gloeosporioides	LD3.4	BSNB_0118	Glomerellales	*Colletotrichum*
Xylaria_cubensis	LD3.6.2	BSNB_0121	Xylariales	*Xylaria*
Cystobasidium_minutum	LR01	BSNB_0122	Saccharomycetales	*Cystobasidium*
Xylaria_sp	LD4.11	BSNB_0123	Xylariales	*Xylaria*
Colletotrichum_sp	LD4.3.2	BSNB_0124	Glomerellales	*Colletotrichum*

Candida_etchellsii	LD5.7	BSNB_0125	Saccharomycetales	*Candida*
Colletotrichum_gloeosporioides	LD8.10	BSNB_0132	Glomerellales	*Colletotrichum*
Colletotrichum_gloeosporioides	LD8.6	BSNB_0133	Glomerellales	*Colletotrichum*
Guignardia_mangiferae	LD8.9	BSNB_0135	Botryosphaeriales	*Guignardia*
Rhizomucor_variabilis	CN16	BSNB_0154	Mucorales	*Rhizomucor*
Rhizomucor_variabilis	CN17	BSNB_0155	Mucorales	*Rhizomucor*
Penicillium_shearii	CN29	BSNB_0167	Eurotiales	*Penicillium*
Penicillium_simplicissimum	CN30	BSNB_0168	Eurotiales	*Penicillium*
Candida_tropicalis	CN36A	BSNB_0174	Saccharomycetales	*Candida*
Rhizomucor_variabilis	CN36B	BSNB_0175	Mucorales	*Rhizomucor*
Rhizomucor_variabilis	CN37B	BSNB_0177	Mucorales	*Rhizomucor*
Cladosporium_sp	CN56	BSNB_0196	Capnodiales	*Cladosporium*
Penicillium_sanguifluum	CN57	BSNB_0197	Eurotiales	*Penicillium*
Exophiala_xenobiotica	CN60bis	BSNB_0201	Chaetothyriales	*Exophiala*
Pseudallescheria_boydii	CN71	BSNB_0212	Microascales	*Pseudallescheria*
Scedosporium_boydii	CN75	BSNB_0216	Microascales	*Scedosporium*
Penicillium_citrinum	CN77	BSNB_0218	Eurotiales	*Penicillium*
Paecilomyces_formosus	CN79	BSNB_0220	Eurotiales	*Paecilomyces*
Scedosporium_boydii	CN81	BSNB_0222	Microascales	*Scedosporium*
Paecilomyces_formosus	CN83	BSNB_0224	Eurotiales	*Paecilomyces*
Pestalotiopsis_microspora	CN84	BSNB_0225	Xylariales	*Pestalotiopsis*
Pseudallescheria_boydii	CN85	BSNB_0226	Microascales	*Pseudallescheria*
Aspergillus_aculeatus	CN86	BSNB_0227	Eurotiales	*Aspergillus*
Penicillium_rubidurum	CN87	BSNB_0228	Eurotiales	*Penicillium*
Penicillium_araracuarense	CN102	BSNB_0243	Eurotiales	*Penicillium*
Penicillium_araracuarense	CN103	BSNB_0244	Eurotiales	*Penicillium*
Aspergillus_sp	CN109	BSNB_0250	Eurotiales	*Aspergillus*
Penicillium_sp	CN112	BSNB_0253	Eurotiales	*Penicillium*

Paecilomyces_formosus	CN122	BSNB_0263	Eurotiales	*Paecilomyces*
Rhizomucor_variabilis	CN125	BSNB_0266	Mucorales	*Rhizomucor*
Acrodictys_sp	A3F8 11.1	BSNB_0283	Hypocreales	*Acrodictys*
Ochroconis_sp	A5F_1 3	BSNB_0284	Venturiales	*Ochroconis*
Ochroconis_sp	A5F_1 5	BSNB_0285	Venturiales	*Ochroconis*
Sordariomycetes_sp	A5F1 13	BSNB_0287	Sordariales	*Sordariomycetes*
Paramicrothyrium_sp	A5F1 9	BSNB_0289	Microthyriales	*Paramicrothyrium*
Colletotrichium_gloeosporioides	A5F1 12	BSNB_0290	Glomerellales	*Colletotrichum*
Apioclypea_sp	A5F1 15	BSNB_0292	Xylariales	*Apioclypea*
Penicillium_sp	A5F1 10.1	BSNB_0293	Eurotiales	*Penicillium*
Chaetomium_sp	A6F1 9	BSNB_0295	Sordariales	*Chaetomium*
Chaetomium_sp	A6F1 12	BSNB_0296	Sordariales	*Chaetomium*
Penicillium_sp	A6F1 13	BSNB_0298	Eurotiales	*Penicillium*
Muscodor_sp	A6F1 19	BSNB_0299	Xylariales	*Muscodor*
Xylaria_sp	A6F1 8.2	BSNB_0300	Xylariales	*Xylaria*
Albonectria_rigidiuscula	A8F_1 9.1	BSNB_0301	Hypocreales	*Albonectria*
Fusarium_sp	A8F_1 9.2	BSNB_0302	Hypocreales	*Fusarium*
Fusarium_sp	A8F_1 13	BSNB_0303	Hypocreales	*Fusarium*
Sordariomycetes_sp	A8F1 2	BSNB_0304	Sordariales	*Sordariomycetes*
Chaetomium_sp	A8F1 5	BSNB_0305	Sordariales	*Chaetomium*
Oxydothis_sp	A1F12 12	BSNB_0516	Xylariales	*Oxydothis*
Diaporthe_sp	A2F_1 4.1	BSNB_0520	Diaporthales	*Diaporthe*
Stereum_hirsutum	A2F_1 7	BSNB_0523	Russulales	*Stereum*
Pseudopestalotiopsis_sp	A2F_1 10	BSNB_0526	Xylariales	*Pseudopestalotiopsis*
Endomelanconiopsis_endophytica	A2F1 1	BSNB_0528	Botryosphaeriales	*Endomelanconiopsis*
Colletotrichium_gloeosporioides	A2F1_2	BSNB_0529	Glomerellales	*Colletotrichum*
Colletotrichum_gloeosporioides	A2F1 3.1	BSNB_0530	Glomerellales	*Colletotrichum*
Stereum_hirsutum	A2F1 3.2	BSNB_0531	Russulales	*Stereum*
Stereum_hirsutum	A2F1 4.1	BSNB_0532	Russulales	*Stereum*
Nodulisporium_sp	A2F1 6	BSNB_0534	Xylariales	*Nodulisporium*

Colletotrichum_gloeosporioides	A2F1 8	BSNB_0536	Glomerellales	*Colletotrichum*
Colletotrichum_gloeosporioides	A2F1 9	BSNB_0537	Glomerellales	*Colletotrichum*
Colletotrichum_gloeosporioides	A2F1 10.22	BSNB_0538	Glomerellales	*Colletotrichum*
Nemania_sp	A2F1 11	BSNB_0539	Xylariales	*Nemania*
Colletotrichum_gloeosporioides	A2F1 12	BSNB_0540	Glomerellales	*Colletotrichum*
Diaporthe_sp	A2F1 13	BSNB_0541	Diaporthales	*Diaporthe*
Clonostachys_sp	A2F11 3	BSNB_0546	Hypocreales	*Clonostachys*
Stereum_hirsutum	A2F11 6.11	BSNB_0532	Russulales	*Stereum*
Colletotrichum_sp	A2F11 6.21	BSNB_0549	Glomerellales	*Colletotrichum*
Stereum_hirsutum	A2F11 7	BSNB_0550	Russulales	*Stereum*
Colletotrichum_gloeosporioides	A2F11_8	BSNB_0551	Glomerellales	*Colletotrichum*
Neopestalotiopsis_sp	A2F11 12.1	BSNB_0556	Xylariales	*Neopestalotiopsis*
Colletotrichum_vietnamense	A2F11 13	BSNB_0557	Glomerellales	*Colletotrichum*
Colletotrichum_gleoesporioides	A2F11 15	BSNB_0559	Glomerellales	*Colletotrichum*
Colletotrichum_gloeosporioides	A3F1 8.121	BSNB_0574	Glomerellales	*Colletotrichum*
Fusarium_concolor	A3F1 8.13	BSNB_0575	Hypocreales	*Fusarium*
Diaporthe_sp	A3F1 9	BSNB_0577	Diaporthales	*Diaporthe*
Diaporthe_sp	A3F1 10	BSNB_0578	Diaporthales	*Diaporthe*
Cladosporium_cladosporioides	A3F1 11	BSNB_0579	Capnodiales	*Cladosporium*
Colletotrichum_gloeosporioides	A3F8 1	BSNB_0580	Glomerellales	*Colletotrichum*
Fusarium_sp	A3F8 2	BSNB_0581	Hypocreales	*Fusarium*
Endomelanconiopsis_endophytica	A3F8 3	BSNB_0582	Botryosphaeriales	*Endomelanconiopsis*
Colletotrichum_sp	A3F8 4	BSNB_0583	Glomerellales	*Colletotrichum*
Endomelanconiopsis_endophytica	A3F8 6	BSNB_0584	Botryosphaeriales	*Endomelanconiopsis*
Xylaria_sp	A3F8 5.2	BSNB_0586	Xylariales	*Xylaria*
Endomelanconiopsis_sp	A3F8 8	BSNB_0587	Botryosphaeriales	*Endomelanconiopsis*
Endomelanconiopsis_endophytica	A3F8 9	BSNB_0588	Botryosphaeriales	*Endomelanconiopsis*
Cladosporium_sp	A3F8 10	BSNB_0589	Capnodiales	*Cladosporium*
Colletotrichum_gloeosporioides	A3F8 12	BSNB_0590	Glomerellales	*Colletotrichum*

Colletotrichum_fructicola	A5F_1 1	BSNB_0615	Glomerellales	*Colletotrichum*
Colletotrichum_gloeosporioides	A5F1 3	BSNB_0622	Glomerellales	*Colletotrichum*
Colletotrichum_gloeosporioides	A5F1 3	BSNB_0623	Glomerellales	*Colletotrichum*
Thanatephorus_cucumeris	A5F1 5	BSNB_0624	Cantharellales	*Thanatephorus*
Colletotrichium_gloeosporioides	A5F1 6.1	BSNB_0625	Glomerellales	*Colletotrichum*
Colletotrichum_sp	A5F1 11	BSNB_0627	Glomerellales	*Colletotrichum*
Colletotrichum_theobromicola	A5F1 17	BSNB_0628	Glomerellales	*Colletotrichum*
Colletotrichum_theobromicola	A5F10 9	BSNB_0637	Glomerellales	*Colletotrichum*
Cladosporium_cladosporioides	A5F10 10	BSNB_0638	Capnodiales	*Cladosporium*
Colletotrichum_gloeosporioides	A5F10 13	BSNB_0641	Glomerellales	*Colletotrichum*
Hypoxylon_investiens	A5F10 14.1	BSNB_0642	Xylariales	*Hypoxylon*
Diaporthe_sp	A6F_1 2	BSNB_0644	Diaporthales	*Diaporthe*
Colletotrichum_theobromicola	A6F_1 3	BSNB_0645	Glomerellales	*Colletotrichum*
Colletotrichum_gloeosporioides	A6F1 1	BSNB_0646	Glomerellales	*Colletotrichum*
Fusarium_sp	A6F1 2	BSNB_0647	Hypocreales	*Fusarium*
Colletotrichum_gloeosporioides	A6F1 5.1	BSNB_0649	Glomerellales	*Colletotrichum*
Colletotrichum_gloeosporioides	A6F1 7	BSNB_0650	Glomerellales	*Colletotrichum*
Fusarium_sp	A6F1 10	BSNB_0651	Hypocreales	*Fusarium*
Colletotrichum_theobromicola	A6F1 11	BSNB_0652	Glomerellales	*Colletotrichum*
Colletotrichum_gloeosporioides	A6F1 15.2	BSNB_0653	Glomerellales	*Colletotrichum*
Colletotrichum_gloeosporioides	A6F1 16.1	BSNB_0654	Glomerellales	*Colletotrichum*
Colletotrichum_gloeosporioides	A6F16 2	BSNB_0655	Glomerellales	*Colletotrichum*
Thanatephorus_cucumeris	A6F16 7.2	BSNB_0660	Cantharellales	*Thanatephorus*
Colletotrichum_gloeosporioides	A6F16 9.1	BSNB_0662	Glomerellales	*Colletotrichum*
Acutodesmus_obliquus	A6F16 7.2	BSNB_0664	Sphaeropleales	*Acutodesmus*
Nodulisporium_sp	A6F16 13	BSNB_0666	Xylariales	*Nodulisporium*
Cladosporium_herbarum	A6F16 15	BSNB_0668	Capnodiales	*Cladosporium*
Fusarium_lateritium	A7F_1 10	BSNB_0678	Hypocreales	*Fusarium*
Diaporthe_sp	A7F1 1	BSNB_0679	Diaporthales	*Diaporthe*

Endomelanconiopsis_endophytica	A7F1 2	BSNB_0680	Botryosphaeriales	*Endomelanconiopsis*
Endomelanconiopsis_endophytica	A7F1 3	BSNB_0681	Botryosphaeriales	*Endomelanconiopsis*
Colletotrichum_siamense	A7F1 4	BSNB_0682	Glomerellales	*Colletotrichum*
Endomelanconiopsis_endophytica	A7F1 6	BSNB_0684	Botryosphaeriales	*Endomelanconiopsis*
Fusarium_avenaceum	A7F1 9	BSNB_0686	Hypocreales	*Fusarium*
Diaporthe_sp	A7F1 10	BSNB_0687	Diaporthales	*Diaporthe*
Colletotrichum_gloeosporioides	A7F1 11	BSNB_0688	Glomerellales	*Colletotrichum*
Penicillium_verrucosum	A7F10 1	BSNB_0691	Eurotiales	*Penicillium*
Endomelanconiopsis_endophytica	A7F10 2	BSNB_0692	Botryosphaeriales	*Endomelanconiopsis*
Endomelanconiopsis_endophytica	A7F10 3	BSNB_0693	Botryosphaeriales	*Endomelanconiopsis*
Colletotrichum_theobromicola	A7F10 6.1	BSNB_0694	Glomerellales	*Colletotrichum*
Colletotrichum_theobromicola	A7F10 7.1	BSNB_0695	Glomerellales	*Colletotrichum*
Colletotrichum_gloeosporioides	A7F10 8	BSNB_0696	Glomerellales	*Colletotrichum*
Colletotrichum_theobromicola	A7F10 11	BSNB_0699	Glomerellales	*Colletotrichum*
Apioclypea_sp	A7F10 12	BSNB_0700	Xylariales	*Apioclypea*
Colletotrichum_gloeosporioides	A7F10 13.1	BSNB_0701	Glomerellales	*Colletotrichum*
Fusarium_lateritium	A7F10 13.2	BSNB_0702	Hypocreales	*Fusarium*
Colletotrichum_theobromicola	A7F10 14.2	BSNB_0703	Glomerellales	*Colletotrichum*
Fusarium_lateritium	A8F_1 4	BSNB_0707	Hypocreales	*Fusarium*
Harknessia_australiensis	A8F1 3	BSNB_0717	Xylariales	*Harknessia*
Xylaria_sp	A8F1 4	BSNB_0718	Xylariales	*Xylaria*
Oxydothis_sp	A8F1 6.2	BSNB_0720	Xylariales	*Oxydothis*
Endomelanconiopsis_endophytica	A8F11 9	BSNB_0731	Botryosphaeriales	*Endomelanconiopsis*
Akanthomyces_attenuatus	A8F11 10	BSNB_0732	Hypocreales	*Akanthomyces*
Endomelanconiopsis_endophytica	A8F11 11	BSNB_0733	Botryosphaeriales	*Endomelanconiopsis*
Colletotrichum_gloeosporioides	AC A2 F11 9.21	BSNB_1019	Glomerellales	*Colletotrichum*
Colletotrichum_theobromicola	VECD4B	BSNB_1021	Glomerellales	*Colletotrichum*
Pestalotiopsis_sp	VECD4A	BSNB_1036	Xylariales	*Pestalotiopsis*
Pestalotiopsis_sp	VECD4C	BSNB_1037	Xylariales	*Pestalotiopsis*

Beauveria_bassiana	VECD6B	BSNB_1038	Hypocreales	*Beauveria*
Trichoderma_sp	VECD6D	BSNB_1040	Hypocreales	*Trichoderma*
Beauveria_bassiana	VECD6E	BSNB_1041	Hypocreales	*Beauveria*
Beauveria_bassiana	VECD11A	BSNB_1042	Hypocreales	*Beauveria*
Mucor_sp	VECD11D	BSNB_1043	Mucorales	*Mucor*
Penicillium_sp	VECD11E	BSNB_1044	Eurotiales	*Penicillium*
Penicillium_sp	VECD11F	BSNB_1045	Eurotiales	*Penicillium*
Penicillium_simplicissimum	VECD11G	BSNB_1046	Eurotiales	*Penicillium*
Neosartorya_sp	VECD13B	BSNB_1048	Eurotiales	*Neosartorya*
Penicillium_oxalicum	VECD13C	BSNB_1049	Eurotiales	*Penicillium*
Beauveria_bassiana	VECD16B	BSNB_1053	Hypocreales	*Beauveria*
Pestalotiopsis_theae	VECD14E	BSNB_1054	Xylariales	*Pestalotiopsis*
Fusarium_sp	ST1C	BSNB_1055	Hypocreales	*Fusarium*
Lecanicillium_sp	ST4C	BSNB_1056	Hypocreales	*Lecanicillium*
Fusarium_sp	ST7C	BSNB_1057	Hypocreales	*Fusarium*
Fusarium_sp	ST9C	BSNB_1058	Hypocreales	*Fusarium*
Arthrinium_sp	ST10C	BSNB_1059	Sordariales	*Arthrinium*
Penicillium_sp	ST12C	BSNB_1061	Hypocreales	*Penicillium*
Mucor_hiemalis	ST15C	BSNB_1063	Mucorales	*Mucor*
Fusarium_sp	ST18C	BSNB_1065	Hypocreales	*Fusarium*
Penicillium_sp	ST21C	BSNB_1067	Eurotiales	*Penicillium*
Beauveria_sp	CLL14070	BSNB_1069	Hypocreales	*Beauveria*
Isaria_farinosa	CLL10038	BSNB_1250	Hypocreales	*Isaria*
Cordyceps_militaris	CLL10030	BSNB_1252	Hypocreales	*Cordyceps*
Ophiocordyceps_forquignonii	CLL11056	BSNB_1253	Hypocreales	*Ophiocordyceps*

TABLE S4. 16S OF STUDIED BACTERIAL STRAINS (BSNB)

>BSNB_0016_ Bacillus_cereus

CCCGCGTCGCATTAGCTAGTTGGTGAGGTAACGGCTCACCAAGGCAACGATGCGTAGCCGA
CCTGAGAGGGTGATCGGCCACACTGGGACTGAGACACGGCCCAGACTCCTACGGGAGGCA
GCAGTAGGGAATCTTCCGCAATGGACGAAAGTCTGACGGAGCAACGCCGCGTGAGTGATGA
AGGCTTTCGGGTCGTAAAACTCTGTTGTTAGGGAAGAACAAGTGCTAGTTGAATAAGCTGGC
ACCTTGACGGTACCTAACCAGAAAGCCACGGCTAACTACGTGCCAGCAGCCGCGGTAATAC
GTAGGTGGCAAGCGTTATCCGGAATTATTGGGCGTAAAGCGCGCGCAGGTGGTTTCTTAAGT
CTGATGTGAAAGCCCACGGCTCAACCGTGGAGGGTCATTGGAAACTGGGAGACTTGAGTGC
AGAAGAGGAAAGTGGAATTCCATGTGTAGCGGTGAAATGCGTAGAGATATGGAGGAACAC
CAGTGGCGAAGGCGACTTTCTGGTCTGTAACTGACACTGAGGCGCGAAAGCGTGGGGAGCA
AACAGGATTAGATACCCTGGTAGTCCACGCCGTAAACGATGAGTGCTAAGTGTTAGGGGGT
TTCCGCCCTTTAGTGCTGAAGTTAACGCATTAAGCACTCCGCC

>BSNB_0034_ Brevibacillus_sp

GGGCGGCTGCTAATACATGCAAGTCGAGCGAGTCTCTTCGGAGGCTAGCGGCGGACGGGTG
AGTAACACGTAGGCAACCTGCCTCTCAGACTGGGATAACATAGGGAAACTTATGCTAATAC
CGGATAGGTTTTTGGACCGCATGGTCCGAAAAGAAAAGATGGCTTCGGCTATCACTGGGAG
ATGGGCCTGCGGCGCATTAGCTAGTTGGTGGGGTAACGGCCTACCAAGGCGACGATGCGTA
GCCGACCTGAGAGGGTGACCGGCCACACTGGGACTGAGACACGGCCCAGACTCCTACGGG
AGGCAGCAGTAGGGAATTTTCCACAATGGACGAAAGTCTGATGGAGCAACGCCGCGTGAAC
GATGAAGGTCTTCGGATTGTAAAGTTCTGTTGTTAGGGACGAATAAGTACCGTTCGAATAGG
GCGGTACCTTGACGGTACCTGACGAGAAAGCCACGGCTAACTACGTGCCAGCAGCCGCGGT
AATACA

>BSNB_0053_ Bacillus_sp

GACAGTGGCGGGGTGCTTACACATGCAAGTCGAACGGAAAGGCCCTGCTTTTGTGGGGTGTT
CGTGTGGAGAAGGGGTGTGTAACACGTGTGTACCCTGCCCTTGTCTTTGGGATAACTTCGGG
AAACTGGGGCTAATACCGGATAAGAGCTCCTGCTGCGTGGTGGGGGTTGGAAAGTTTCTGCG
GGGGGGATGGACTCGCGGCTTATCTATTTGTTGGTGGGGTAGTGGCTTACCAAGGCTTTGAC
GGGTAGCCGGCCTGACAGGGTGGCCGACCACTTTGGGACTGAGATACAGCCCACACTCATA
CCGGAGGCAGCAGTGGGGAATATTTATAGATGGGCGGAAGCCTGATGCACTGCGCCGCGTG
CGGGATGACGGCCTTAAGGTTGTAAACCCCTTTCACCTGTGACTAAGCGTGAATGACGGGTA
TGGGTAAATAAGCACCGCCTAACTACATGCCATCAGACGCCCTAATATAACACCGTGCCAG
CAGCCGCGGTAATACAAAAAAAAAAAAAGGGGGGT

>BSNB_0139_ Serratia_sp

GCGGCGGCTTACACATGCAAGTCGAGCGGTAGCACAAGGGAGCTTGCTCYCTGGGTGACGA
GCGGCGGACGGGTGAGTAATGTCTGGGAAACTGCCTGATGGAGGGGGATAACTACTGGAAA
CGGTAGCTAATACCGCATAACGTCGCARGACCAAAGAGGGGGACCTTCGGGCCTCTTGCCA
TCAGATGTGCCCAGATGGGATTAGCTAGTAGGTGGGGTAATGGCTCACCTAGGCAACRATSC
CTAGMTGGTCTGAGAGGATGACCAGCCACACTGGAACTGAGACACGGTCCAKACTCCTACG
GGAGGCAGCAGTGGGGAATATTGCACAATGGGCGCAAGCCTGATGCAGCCRTGCCGCGTGT
GTGAAGAAGGCCTTCGGGTTGTAAAGCACTTTCAGCGAGRAGGAAGGTGGTGAACTTAATA
CGTTCATCAATTGACGTTACTCGCAAAAAAAGCACCGGCCAACTCCGTGCCAGCASCCGSGR
GTAATAMAGCGGCGGCTTACACATGCAAGTCGAGCGGTAGCACAAGGGAGCTTGCTCYCTG
GGTGACGAGCGGCGGACGGGTGAGTAATGTCTGGGAAACTGCCTGATGGAGGGGGATAACT
ACTGGAAACGGTAGCTAATACCGCATAACGTCGCARGACCAAAGAGGGGGACCTTCGGGCC
TCTTGCCATCAGATGTGCCCAGATGGGATTAGCTAGTAGGTGGGGTAATGGCTCACCTAGGC
AACRATSCCTAGMTGGTCTGAGAGGATGACCAGCCACACTGGAACTGAGACACGGTCCAKA

CTCCTACGGGAGGCAGCAGTGGGGAATATTGCACAATGGGCGCAAGCCTGATGCAGCCRTG
CCGCGTGTGTGAAGAAGGCCTTCGGGTTGTAAAGCACTTTCAGCGAGRAGGAAGGTGGTGA
ACTTAATACGTTCATCAATTGACGTTACTCGCAAAAAAAGCACCGGCCAACTCCGTGCCAGC
ASCCGSGRGTAATAMA

>BSNB_0140_ Serratia_marcescens

GCGGGAGGCCTAACACATGCAAGCCGAGCGGTAGAGATCTTTCGGGATCTTGAGAGCGGCG
TACGGGTGCGGAACACGTGTGCAACCTGCCTTTATCTGGGGGATAGCCTTTCGAAAGGAAG
ATTAATACCCCATAATATATCGACTGGCATCAGTTGATATTGAAAACTCCGGTGGATAGAGA
TGGGCACGCGCAAGATTAGATAGTTGGTGAGGTAACGGCTCACCAAGTCTGCGATCTTTAGG
GGGCCTGAGAGGGTGATCCCCCACACTGGTACTGAGACACGGACCAGACTCCTACGGGAGG
CAGCAGTGAGGAATATTGGACAATGGGTGAGAGCCTGATCCAGCCATCCCGCGTGAAGGAC
GACGGCCCTATGGGTTGTAAACTTCTTTTGTATAGGGATAAACCTAGATACGTGTATCTAGCT
GAAGGTACTATACGAATAAGCACCGGCTAACTCCGTGCCAGCAGCCGCGGTAATACGGAGG
GTGCAAGCGTTATCCGGATTTATTGGGTTTAAAGGGTCCGTAGGCGGATTTGTAAGTCAGTG
GTGAAATCTCACAGCTTAACTGTGAAACTGCCATTGATACTGCAAGTCTTGAGTGTTGTTGA
AGTAGCTGGAATAAGTAGTGTAGCGGTGAAATGCATAGATATTACTTAGAACACCAATTGC
GAAGGCAGGTTACTAAGCAACAACTGACGCTGATGGACGAAAGCGTGGGGAGCGAACAGG
ATTAGATACCCTGGTAGTCCACGCCGTAAACGATGCTAACTCGTTTTTGGCTTTCGGGTTCA
GAGACTAAGCGAAAGTGATAAGTTAG
>BSNB_0143_ Burkholderia_seminalis

TGCTCTTGGGTGACGAGTGGCGGACGGGTGAGTAATGTCTGGGGATCTGCCCGATAGAGGG
GGATAACCACTGGAAACGGTGGCTAATACCGCATAACGTCGCAAGACCAAAGAGGGGGAC
CTTCGGGCCTCTCACTATCGGATGAACCCAGATGGGATTAGCTAGTAGGCGGGGTAATGGCC
CACCTAGGCGACGATCCCTAGCTGGTCTGAGAGGATGACCAGCCACACTGGAACTGAGACA
CGGTCCAGACTCCTACGGGAGGCAGCAGTGGGGAATATTGCACAATGGGCGCAAGCCTGAT
GCAGCCATGCCGCGTGTATGAAGAAGGCCTTCGGGTTGTAAAGTACTTTCAGCGGGGAGGA
AGGCGACGGGGTTAATAACCCTGTCGATTGACGTTACCCGCAGAAGAAGCACCGGCTAACT
CCGTGCCAGCAGCCGCGGTAATACGGAGGGTGCAAGCGTTAATCGGAATTACTGGGCGTAA
AGCGCACGCAGGCGGTCTGTTAAGTCAGATGTGAAATCCCCGGGCTTAACCTGGGAACTGC
ATTTGAAACTGGCAGGCTTGAGTCTTGTAGAGGGGGGTAGAATTCCAGGTGTAGCGGTGAA
ATGCGTAGAGATCTGGAGGAATACCGGTGGCGAAGGCGGCCCCCTGGACAAAGACTGACG
CTCAGGTGCGAAAGCGTGGGGAGCAAACAGGATTAGATACCCTGGTAGTCCACGCCGTAAA
CGATGTCGACTTGGAGGTTGTTCCCTTGAGGAGTGGCTTCCGGAGCTAACGCGTTAAGT
>BSNB_0150_ Pseudomonas_beteli

GAGTGAACGCTGGCGGTAGGCCTAACACATGCAAGTCGAACGGCAGCACAGGAGAGCTTG
CTCTCTGGGTGGCGAGTGGCGGACGGGTGAGGAATACATCGGAATCTACTCTGTCGTGGGG
GATAACGTAGGGAAACTTACGCTAATACCGCATACGACCTACGGGTGAAAGCAGGGGATCT
TCGGACCTTGCGCGATTGAATGAGCCGATGTCGGATTAGCTAGTTGGCGGGGTAAAGGCCC
ACCAAGGCGACGATCCGTAGCTGGTCTGAGAGGATGATCAGCCACACTGGAACTGAGACAC
GGTCCAGACTCCTACGGGAGGCAGCAGTGGGGAATATTGGACAATGGGCGCAAGCCTGATC
CAGCCATACCGCGTGGGTGAAGAAGGCCTTCGGGTTGTAAAGCCCTTTTGTTGGGAAAGAA
ATCCAGCCGGCTAATACCTGGTTGGGATGACGGTACCCAAAGAATAAGCACCGGCTAACTT
CGTGCCAGCAGCCGCGGTAATACGAAGGGTGCAAGCGTTACTCGGAATTACTGGGCGTAAA
GCGTGCGTAGGTGGTTATTTAAGTCCGTTGTGAAAGCCCTGGGCTCAACCTGGGAACTGCAG
TGGATACTGGATGACTAGAATGTGGTAGAGGGTAGCGGAATTCCTGGTGTAGCAGTGAAAT
GCGTAGAGATCAGGAGGAACATCCATGGCGAAGGCAGCTACCTGGACCAACATTGACACT
GAGGCACGAAAGCGTGGGGAGCAAACAGGATTAGATACCCTGGTAGTCCACGCCCTAAAC

GATGCGAACTGGATGTTGGGTGCAATTTGGCACGCAGTATCGAAGCTAACGCGTTAAGTTCG
CCG
>BSNB_0152_ Serratia_marcescens

CGGCAGGCTTAACACATGCAAGTCGAGCGGTAGCACAAGGGAGCTTGCTCYCTGGGTGACG
AGCGGCGGACGGGTGAGTAATGTCTGGGAAACTGCCTGATGGAGGGGGATAACTACTGGAA
ACGGTAGCTAATACCGCATAACGTCGCAAGACCAAAGAGGGGGACCTTCGGGCCTCTTGCC
ATCAGATGTGCCCAGATGGGATTAGCTAGTAGGTGGGGTAATGGCTCACCTAGGCGACGAT
CCCTAGCTGGTCTGAGAGGATGACCAGCCACACTGGAACTGAGACACGGTCCAGACTCCTA
CGGGAGGCAGCAGTGGGGAATATTGCACAATGGGCGCAAGCCTGATGCAGCCATGCCGCGT
GTGTGAAGAAGGCCTTCGGGTTGTAAAGCACTTTCAGCGAGGAGGAAGGTGGTGAACTTAA
TACGTTCATCAATTGACGTTACTCGCAGAAGAAGCACCGGCTAACTCCGTGCCAGCAGCCG
CRRGGTAATACA

>BSNB_0153_ Achromobacter_sp

GCGGGWGCCTTACACATGCAAGTCGAACGGCAGCACGGACTTCGGTCTGGTGGCGAGTGGC
GAACGGGTGAGTAATGTATCGGAACGTGCCTAGTAGCGGGGGATAACTACGCGAAAGCGTA
GCTAATACCGCATACGCCCTACGGGGGAAAGCAGGGGATCGCAAGACCTTGCACTATTAGA
GCGGCCGATATCGGATTAGCTAGTTGGTGGGGTAACGGCTCACCAAGGCGACGATCCGTAG
CTGGTTTGAGAGGACGACCAGCCACACTGGGACTGAGACACGGCCCAGACTCCTACGGGAG
GCAGCAGTGGGGAATTTTGGACAATGGGGGAAACCCTGATCCAGCCATCCCGCGTGTGCGA
TGAAGGCCTTCGGGTTGTAAAGCACTTTTGGCAGGAAAGAAACGTCATGGGTTAATACCCC
GTGAAACTGACGGTACCTGCAGAATAAGCACCGGCTAACTACGTGCCAGCAGCCGCGGGTA
ATACA

>BSNB_0158_ Burkholderia_sp

GCCTTACACATGCAAGTCGAACGGCAGCACGGGTGCTTGCACCTGGTGGCGAGTGGCGAAC
GGGTGAGTAATACATCGGAACATGTCCTGTAGTGGGGGATAGCCCGGCGAAAGCCGGATTA
ATACCGCATACGATCTACGGATGAAAGCGGGGGACCTTCGGGCCTCGCGCTATAGGGTTGG
CCGATGGCTGATTAGCTAGTTGGTGGGGTAAAGGCCTACCAAGGCGACGATCAGTAGCTGG
TCTGAGAGGACGACCAGCCACACTGGGACTGAGACACGGCCCAGACTCCTACGGGAGGCA
GCAGTGGGGAATTTTGGACAATGGGCGAAAGCCTGATCCAGCAATGCCGCGTGTGTGAAGA
AGGCCTTCGGGTTGTAAAGCACTTTTGTCCGGAAAGAAATCCTTGGCCCTAATACGGTCGGG
GGATGACGGTACCGGAAGAATAAGCACCGGCTAACTACGTGCCAGCAGCCGCGGGTAAT
ACA

>BSNB_0165_ Serratia_marcescens

GCGGMGGCTTAACACATGCAAGTCGAGCGGTAGCACAAGRGAGCTTGCTCTCTGGGTGACG
AGCGGCGGACGGGTGAGTAATGTCTGGGAAACTGCCTGATGGAGGGGGATAACTACTGGAA
ACGGTAGCTAATACCGCATAACGTCGCAAGACCAAAGAGGGGGACCTTCGGGCCTCTTGCC
ATCAGATGTGCCCAGATGGGATTAGCTAGTAGGTGGGGTAATGGCTCACCTAGGCGACGAT
CCCTAGCTGGTCTGAGAGGATGACCAGCCACACTGGAACTGAGACACGGTCCAGACTCCTA
CGGGAGGCAGCAGTGGGGAATATTGCACAATGGGCGCAAGCCTGATGCAGCCATGCCGCGT
GTGTGAAGAAGGCCTTCGGGTTGTAAAGCACTTTCAGCGAGGAGGAAGGTGGTGARCTTAA
TACGYTCATCAATTGACGTTACTCGCAGAAGAAGCACCGGCTAACTCCGTGCCAGCAGCCGS
GGGGTAATACA

>BSNB_0166_ Burkholderia_sp

GGMMTGCCTTACACATGCAAGTCGAACGGCAGCACGGGTGCTTGCACCTGGTGGCGAGTGG
CGAACGGGTGAGTAATACATCGGAACATGTCCTGTAGTGGGGGATAGCCCGGCGAAAGCCG
GATTAATACCGCATACGATCTACGGATGAAAGCGGGGGACCTTCGGGCCTCGCGCTATAGG
GTTGGCCGATGGCTGATTAGCTAGTTGGTGGGGTAAAGGCCTACCAAGGCGACGATCAGTA
GCTGGTCTGAGAGGACGACCAGCCACACTGGGACTGAGACACGGCCCAGACTCCTACGGG
AGGCAGCAGTGGGGAATTTTGGACAATGGGCGAAAGCCTGATCCAGCAATGCCGCGTGTGT
GAAGAAGGCCTTCGGGTTGTAAAGCACTTTTGTCCGGAAAGAAATCCTTGGCCCTAATACGG
TCGGGGGATGACGGTACCGGAAGAATAAGCACCGGCTAACTACGTGCCAGCAGCCGCGNG
TAATACA

>BSNB_0170_ Burkholderia_pyrrocinia

CGGGTGCCTTACACATGCAAGTCGAACGGCAGCACGGGTGCTTGCACCTGGTGGCGAGTGG
CGAACGGGTGAGTAATACATCGGAACATGTCCTGTAGTGGGGGATAGCCCGGCGAARGCCG
GATTAATACCGCATACGATCTACGGATGAAAGCGGGGGACCTTCGGGCCTCGCGCTATAGG
GTTGGCCGATGGCTGATTAGCTAGTTGGTGGGGTAAAGGCCTACCAAGGCGACGATCAGTA
GCTGGTCTGAGAGGACGACCAGCCACACTGGGACTGAGACACGGCCCAGACTCCTACGGG
AGGCAGCAGTGGGGAATTTTGGACAATGGGCGAAAGCCTGATCCAGCAATGCCGCGTGTGT
GAAGAAGGCCTTCGGGTTGTAAAGCACTTTTGTCCGGAAAGAAATCCTTGGCTCTAATACAG
TCGGGGGATGACGGTACCGGAAGAATAAGCACCGGCAAACTACGTGCCAGCAGCCGCGGG
TAATACA

>BSNB_0171_ Burkholderia_sp

CGGCTGCCTTACACATGCAAGTCGAACGGCAGCACGGGTGCTTGCACCTGGTGGCGAGTGG
CGAACGGGTGAGTAATACATCKGAACATGTCCTGTAGTGGGGGATAGCCCGGCGAAGGCCG
GATTAATACCGCAYACGATCTACGGATGAAAGCGGGGGACCTTCGGGCCTCGCGCTATAGG
GTTGGCCGATGGCTGATTARCTAGTTGGTGGGGTAAAGGCCTACCAAGGCGACMATCARTA
GCTGGTCTGAGAGGACGACCAGCCACACTGGGACTGAGACACGGCCCAGACTCCTACSGGA
GGCAGCAGTGGGGAATTTTGGACAATGGGCGAAAGCCTGATCCAGCMATGCCKCGTGTGTG
AAGAAGGCCTTCGGGTTGTAAAGCACTTTTGTCCGGAAAGAAATCCTTGRCTCTAATACAGT
CGGGGGATGACGGTACCGGAAGAATAAGCACCGGCAAACTACGTGCCAGCAGCCGCSGSTA
ATACA

>BSNB_0172_ Burkholderia_cepacia

MTGCCTTACACATGCAGTCGACGGCAGCACGGGTGCTTGCACCTGGTGGCGAGTGGCGAAC
GGGTGAGTAATACATCGGAACATGTCCTGTAGTGGGGGATAGCCCGGCGAAAGCCGGATTA
ATACCGCATACGATCTACGGATGAAAGCGGGGGACCTTCGGGCCTCGCGCTATAGGGTTGG
CCGATGGCTGATTAGCTAGTTGGTGGGGTAAAGGCCTACCAAGGCGACGATCAGTAGCTGG
TCTGAGAGGACGACCAGCCACACTGGGACTGAGACACGGCCCAGACTCCTACGGGAGGCA
GCAGTGGGGAATTTTGGACAATGGGCGAAAGCCTGATCCAGCAATGCCGCGTGTGTGAAGA
AGGCCTTCGGGTTGTAAAGCACTTTTGTCCGGAAAGAAATCCTTGGCTCTAATACAGTCGGG
GGATGACGGTACCGGAAGAATAAGCACCGGCAAACTACGTGCCAGCAGCCGCGGGTAATA
CAA

>BSNB_0178_ Burkholderia_sp

GGCGGCTGCCTTACACATGCAAGTCGAACGGCAGCACGGGTGCTTGCACCTGGTGGCGAGT
GGCGAACGGGTGAGTAATACATCGGAACATGTCCTRTAGTGGGGGATAGCCCGGCGAAAGC
CGGATTAATACCGCATACGATCTACGGATGAAAGCGGGGGACCTTCGGGCCTCGCGCTATA
GGGTTGGCCGATGGCTGATTAGCTAGTTGGTGGGGTAAAGGCCTACCAAGGCGACGATCAG

TAGCTGGTCTGAGAGGACGACCAGCCACACTGGGACTGAGACACGGCCCAGACTCCTACGG
GAGGCAGCAGTGGGGAATTTTGGACAATGGGCGAAAGCCTGATCCAGCAATGCCGCGTGTG
TGAAGAAGGCCTTCGGGTTGTAAAGCACTTTTGTCCGGAAAGAAATCCTTGGCTCTAATACA
GTCGGGGGATGACGGTACCGGAAGAATAAGCACCGGCWAACTACGTGCCAGCAGCCGSRR
GTAATACA

>BSNB_0179_ Lactococcus_garvieae

CTCAGGACGAACGCTGGCGGCGTGCCTAATACATGCAAGTCGAGCGATGATTAAAGATAGC
TTGCTATTTTTATGAAGAGCGGCGAACGGGTGAGTAACGCGTGGGAAATCTGCCGAGTAGC
GGGGGACAACGTTTGGAAACGAACGCTAATACCGCATAACAATGAGAATCGCATGATTTTC
ATTTAAAAGAAGCAATTGCTTCACTACTTGATGATCCCGCGTTGTATTAGCTAGTTGGTAGTG
TAAAGGACTACCAAGGCGATGATACATAGCCGACCTGAGAGGGTGATCGGCCACACTGGG
ACTGAGACACGGCCCAGACTCCTACGGGAGGCAGCAGTAGGGAATCTTCGGCAATGGGGG
CAACCCTGACCGAGCAACGCCGCGTGAGTGAAGAAGGTTTTCGGATCGTAAAACTCTGTTGT
TAGAGAAGAACGTTAAGTAGAGTGGAAAATTACTTAAGTGACGGTATCTAACCAGAAAGGG
ACGGCTAACTACGTGCCAGCAGCCGCGGTAATACGTAGGTCCCAAGCGTTGTCCGGATTTAT
TGGGCGTAAAGCGAGCGCAGGTGGTTTCTTAAGTCTGATGTAAAAGGCAGTGGCTCAACCA
TTGTGTGCATTGGAAACTGGGAGACTTGAGTGCAGGAGAGGAGAGTGGAATTCCATGTGTA
GCGGTGAAATGCGTAGATATATGGAGGAACACCGGAGGCGAAAGCGGCTCTCTGGCCTGTA
ACTGACACTGAGGCTCGAAAGCGTGGGGAGCAAACAGGATTAGATACCCTGGTAGTCCACG
CCGTAAACGATGAGTGCTAGCTGTAGGGAGCTATAAGTTCTCTGTAGCGCAGCTAACGCATT
AAGCACTCCGCCT
>BSNB_0180_ Lactococcus_garvieae

TGGCGGGCTGCCTAATACATGCAAGTCGAGCGATGATTAAAGATAGCTTGCTATTTTTATGA
AGAGCGGCGAACGGGTGAGTAACGCGTGGGAAATCTGCCGAGTAGCGGGGGACAACGTTT
GGAAACGAACGCTAATACCGCATAACAATGAGAATCGCATGATTTTCATTTAAAAGAAGCA
ATTGCTTCACTACTTGATGATCCCGCGTTGTATTAGCTAGTTGGTAGTGTAAAGGACTACCAA
GGCGATGATACATAGCCGACCTGAGAGGGTGATCGGCCACACTGGGACTGAGACACGGCCC
AGACTCCTACGGGAGGCAGCAGTAGGGAATCTTCGGCAATGGGGGCAACCCTGACCGAGC
AACGCCGCGTGAGTGAAGAAGGTTTTCGGATCGTAAAACTCTGTTGTTAGAGAAGAACGTT
AAGTAGAGTGGAAAATTACTTAAGTGACGGTATCTAACCAGAAAGGGACGGCTAACTACGT
GCCAGCAGCCGGRAGGTAATACA

>BSNB_0181_ Burkholderia_arboris

CAGATTGAACGCTGGCGGCATGCCTTACACATGCAAGTCGAACGGCAGCACGGGTGCTTGC
ACCTGGTGGCGAGTGGCGAACGGGTGAGTAATACATCGGAACATGTCCTGTAGTGGGGGAT
AGCCCGGCGAAAGCCGGATTAATACCGCATACGATCTACGGATGAAAGCGGGGGACCTTCG
GGCCTCGCGCTATAGGGTTGGCCGATGGCTGATTAGCTAGTTGGTGGGGTAAAGGCCTACCA
AGGCGACGATCAGTAGCTGGTCTGAGAGGACGACCAGCCACACTGGGACTGAGACACGGC
CCAGACTCCTACGGGAGGCAGCAGTGGGGAATTTTGGACAATGGGCGAAAGCCTGATCCAG
CAATGCCGCGTGTGTGAAGAAGGCCTTCGGGTTGTAAAGCACTTTTGTCCGGAAAGAAATCC
TTGGCCCTAATACGGTCGGGGGATGACGGTACCGGAAGAATAAGCACCGGCTAACTACGTG
CCAGCAGCCGCGGTAATACGTAGGGTGCGAGCGTTAATCGGAATTACTGGGCGTAAAGCGT
GCGCAGGCGGTTTGCTAAGACCGATGTGAAATCCCCGGGCTCAACCTGGGAACTGCATTGG
TGACTGGCAGGCTAGAGTATGGCAGAGGGGGGTAGAATTCCACGTGTAGCAGTGAAATGCG
TAGAGATGTGGAGGAATACCGATGGCGAAGGCAGCCCCCTGGGCCAATACTGACGCTCATG
CACGAAAGCGTGGGGAGCAAACAGGATTAGATACCCTGGTAGTCCACGCCCTAAACGATGT
CAACTAGTTGTTGGGGATTCATTTCCTTAGTAACGTAGCTAACGCGTGAAGTTGACCGCC

>BSNB_0182_ Enterobacter_tabaci

CGAACGGCTGGCGGCGTGCCTAATACATGCAAGTTGAGCGCTGAAGGTTGGTACTTGTACCG
ACTGGATGAGCAGCGAACGGGTGAGTAACGCGTGGGAATCTGCCTTTGAGCGGGGGACA
ACATTTGGAAACGAATGCTAATACCGCATAAAAACTTTAAACACAAGTTTTAAGTTTGAAA
GATGCAATTGCATCACTCAAAGATGATCCCGCGTTGTATTAGCTAGTTGGTGAGGTAAAGGC
TCACCAAGGCGATGATACATAGCCGACCTGAGAGGGTGATCGGCCACATTGGGACTGAGAC
ACGGCCCAAACTCCTACGGGAGGCAGCAGTAGGGAATCTTCGGCAATGGACGAAAGTCTGA
CCGAGCAACGCCGCGTGAGTGAAGAAGGTTTTCGGATCGTAAAACTCTGTTGGTAGAGAAG
AACGTTGGTGAGAGTGGAAAGCTCATCAAGTGACGGTAACTACCCAGAAAGGGACGGCTA
ACTACGTGCCAGCAGCCGCGGTAATACGTAGGTCCCGAGCGTTGTCCGGATTTATTGGGCGT
AAAGCGAGCGCAGGTGGTTTATTAAGTCTGGTGTAAAAGGCAGTGGCTCAACCATTGTATGC
ATTGGAAACTGGTAGACTTGAGTGCAGGAGAGGAGAGTGGAATTCCATGTGTAGCGGTGAA
ATGCGTAGATATATGGAGGAACACCGGTGGCGAAAGCGGCTCTCTGGCCTGTAACTGACAC
TGAGGCTCGAAAGCGTGGGGAGCAAACAGGATTAGATACCTCTGGTAGTCCACGCCGTAAA
CGATGAGTGCTAGATGTAGGGAGCTATAAGTTCTCTGTATCGCAGCTAACGCAATAAGC
>BSNB_0191_ Enterobacter_tabaci

GGCGGCCTACACATGCAAGTCGAGCGGATGAGAGGAGCTTGCTCCTGGATTCAGCGGCGGA
CGGGTGAGTAATGCCTAGGAATCTGCCTGGTAGTGGGGGACAACGTTTCGAAAGGAACGCT
AATACCGCATACGTCCTACGGGAGAAAGCAGGGGACCTTCGGGCCTTGCGCTATCAGATGA
GCCTAGGTCGGATTAGCTAGTTGGTGAGGTAATGGCTCACCAAGGCGACGATCCGTAACTG
GTCTGAGAGGATGATCAGTCACACTGGAACTGAGACACGGTCCAGACTCCTACGGGAGGCA
GCAGTGGGGAATATTGGACAATGGGCGAAAGCCTGATCCAGCCATGCCGCGTGTGTGAAGA
AGGTCTTCGGATTGTAAAGCACTTTAAGTTGGGAGGAAGGGCAGTAAATTAATACTTTGCTG
TTTTGACGTTACCGACAGAATAAGCACCGGCTAACTCTGTGCCAGCAGCCGCNNGTAATAC
A

>BSNB_0192_ Enterobacter_tabaci

CGAGCGGCGGACGGGTGAGTAATGTCTGGGAAACTGCCTGATGGAGGGGGATAACTACTGG
AAACGGTAGCTAATACCGCATAACGTCGCAAGACCAAAGAGGGGGACCTTCGGGCCTCTTG
CCATCAGATGTGCCCAGATGGGATTAGCTAGTAGGTGGGGTAACGGCTCACCTAGGCGACG
ATCCCTAGCTGGTCTGAGAGGATGACCAGCCACACTGGAACTGAGACACGGTCCAGACTCC
TACGGGAGGCAGCAGTGGGGAATATTGCACAATGGGCGCAAGCCTGATGCAGCCATGCCGC
GTGTATGAAGAAGGCCTTCGGGTTGTAAAGTACTTTCAGCGGGGAGGAAGGTGTTGAGGTTA
ATAACCTCAGCAATTGACGTTACCCGCAGAAGAAGCACCGGCTAACTCCGTGCCAGCAGCC
GCGGTAATACGGAGGGTGCAAGCGTTAATCGGAATTACTGGGCGTAAAGCGCACGCAGGCG
GTCTGTCAAGTCGGATGTGAAATCCCCGGGCTCAACCTGGGAACTGCATTCGAAACTGGCA
GGCTAGAGTCTTGTAGAGGGGGGTAGAATTCCAGGTGTAGCGGTGAAATGCGTAGAGATCT
GGAGGAATACCGGTGGCGAAGGCGGCCCCCTGGACAAAGACTGACGCTCAGGTGCGAAAG
CGTGGGGAGCAAACAGGATTAGATACCCTGGTAGTCCACGCCGTAAACGATGTCGACTTGG
AGGTTGTGCCCTTGAGGCGTGGCTTCCGGAGCTAACGCGTTAAGTCGACC
>BSNB_0194_ Pseudomonas_sp

GMGGCCTAACACATGCAAGTCGAGCGGTAGAGAGAAGCTTGCTTCTCTTGAGAGCGGCGGA
CGGGTGAGTAATGCCTAGGAATCTGCCTGGTAGTGGGGGATAACGTTCGGAAACGAACGCT
AATACCGCATACGTCCTACGGGAGAAAGCAGGGGACCTTCGGGCCTTGCGCTATCAGATGA
GCCTAGGTCGGATTAGCTAGTTGGTGAGGTAATGGCTCACCAAGGCGACGATCCGTAACTG
GTCTGAGAGGATGATCAGTCACACTGGAACTGAGACACGGTCCAGACTCCTACGGGAGGCA
GCAGTGGGGAATATTGGACAATGGGCGAAAGCCTGATCCAGCCATGCCGCGTGTGTGAAGA
AGGTCTTCGGATTGTAAAGCACTTTAAGTTGGGAGGAAGGGCATTAACCTAATACGTTAGTG

TTTTGACGTTACCGACAGAATAAGCACCGGCTAACTCTGTGCCAGCAGCCGCGGTAATAC
A

>BSNB_0202_ Kitasatospora_sp

GGCGTGCTTACACATGCAAGTCGAACGGTGAAGCCCTTCGGGGTGGATCAGTGGCGAACGG
GTGAGTAACACGTGGGGAATCTGCCCTGAACTCTGGGACAAGCCTTGGAAACGAGGTCTAA
TACCGGATACGACCTTCTCCTGCATGGGGGTTGGTGGAAAGCTCCGGCGGTTCAGGATGATC
CCGCGGCCTATCAGCTTGTTGGTGGGGTAATGGCCTACCAAGGCGACGACGGGTAGCCGGC
CTGAGAGGGCGACCGGCCACACTGGGACTGAGACACGGCCCAGACTCCTACGGGAGGCAG
CAGTGGGGAATATTGCACAATGGGCGGAAGCCTGATGCAGCGACGCCGCGTGAGGGATGA
CGGCCTTCGGGTTGTAAACCTCTTTCAGCAGGGAAGAAGCGCAAGTGACGGTACCTGCAGA
AGAAGCACCGGCTAACTACGTGCCAGCAGCCGCGGGGTAATACA

>BSNB_0230_ Bacillus_cereus

TGGCGGCGTGCTAATACATGCAAGTCGAGCGAATGGATTGAGAGCTTGCTCTCAAGAAGTT
AGCGGCGGACGGGTGAGTAACACGTGGGTAACCTGCCCATAAGACTGGGATAACTCCGGG
AAACCGGGGCTAATACCGGATAACATTTTGAACYGCATGGTTCGAAATTGAAAGGCGGCTT
CGGCTGTCACTTATGGATGGACCCGCGTCGCATTAGCTAGTTGGTGAGGTAACGGCTCACCA
AGGCAACGATGCGTAGCCGACCTGAGAGGGTGATCGGCCACACTGGGACTGAGACACGGC
CCAGACTCCTACGGGAGGCAGCAGTAGGGAATCTTCCGCAATGGACGAAAGTCTGACGGAG
CAACGCCGCGTGAGTGATGAAGGCTTTCGGGTCGTAAAACTCTGTNGTTAGGGAAGAACAA
GTGCTAGTTGAATAAGCTGGCACCTTGACGGTACCTAACCAGAAAGCCACGGCTAACTACG
TGCCAGCAGCCGCRGTAATACA

>BSNB_0232_ Bacillus_cereus

TAGGCGGCTGCCTAATACATGCAAGTCGAGCGAATGGATTAAGAGCTTGCTCTTATGAAGTT
AGCGGCGGACGGGTGAGTAACACGTGGGTAACCTGCCCATAAGACTGGGATAACTCCGGG
AAACCGGGGCTAATACCGGATAACATTTTGAACCGCATGGTTCGAAATTGAAAGGCGGCTT
CGGCTGTCACTTATGGATGGACCCGCGTCGCATTAGCTAGTTGGTGAGGTAACGGCTCACCA
AGGCAACGATGCGTAGCCGACCTGAGAGGGTGATCGGCCACACTGGGACTGAGACACGGC
CCAGACTCCTACGGGAGGCAGCAGTAGGGAATCTTCCGCAATGGACGAAAGTCTGACGGAG
CAACGCCGCGTGAGTGATGAAGGCTTTCGGGTCGTAAAACTCTGTTGTTAGGGAAGAACAA
GTGCTAGTTGAATAAGCTGGCACCTTGACGGTACCTAACCAGAAAGCCACGGCTAACTACG
TGCCAGCAGCCGCGGTAATACA

>BSNB_0233_ Lysinibacillus_xylanilyticus

GTCGAGCGAACAGAGAAGGAGCTTGCTCCTTTGACGTTAGCGGCGGACGGGTGAGTAACAC
GTGGGCAACCTACCTTATAGTTTGGGATAACTCCGGGAAACCGGGGCTAATACCGAATAAT
CTATTTCACTTCATGGTGAAATACTGAAAGACGGCATCTCGCTGTCGCTATAAGATGGGCCC
GCGGCGCATTAGCTAGTTGGTGAGGTAACGGCTCACCAAGGCGACGATGCGTAGCCGACCT
GAGAGGGTGATCGGCCACACTGGGACTGAGACACGGCCCAGACTCCTACGGGAGGCAGCA
GTAGGGAATCTTCCACAATGGGCGAAAGCCTGATGGAGCAACGCCGCGTGAGTGAAGAAG
GTTTTCGGATCGTAAAACTCTGTTGTAAGGGAAGAACAAGTACAGTAGTAACTGGCTGTACC
TTGACGGTACCTTATTAGAAAGCCACGGCTAACTACGTGCCAGCAGCCGCGGTAATACGTA
GGTGGCAAGCGTTGTCCGGAATTATTGGGCGTAAAGCGCGCGCAGGCGGTCCTTTAAGTCTG
ATGTGAAAGCCCACGGCTCAACCGTGGAGGGTCATTGGAAACTGGGGGACTTGAGTGCAGA
AGAGGAAAGTGGAATTCCAAGTGTAGCGGTGAAATGCGTAGAGATTTGGAGGAACACCAG
TGGCGAAGGCGACTTTCTGGTCTGTAACTGACGCTGAGGCGCGAAAGCGTGGGGAGCAAAC

AGGATTAGATACCCTGGTAGTCCACGCCGTAAACGATGAGTGCTAAGTGTTAGGGGGTTTCC
GCCCCTTAGTGCTGCAGCTAACGCATTAAGCACTCCGC
>BSNB_0234_ Streptomyces_sp

TGGSGGGCTGCTTACACATGCAAGTCGAACGATGAAGCCCTTCGGGGTGGATTAGTGGCGA
ACGGGTGAGTAACACGTGGGCAATCTGCCCTGCACTCTGGGACAAGCCCTGGAAACGGGGT
CTAATACCGGATATGACCATCTTGGGCATCCTTGATGGTGTAAAGCTCCGGCGGTGCAGGAT
GAGCCCGCGGCCTATCAGCTTGTTGGTGAGGTAATGGCTCACCAAGGCGACGACGGGTAGC
CGGCCTGAGAGGGCGACCGGCCACACTGGGACTGAGACACGGCCCAGACTCCTACGGGAG
GCAGCAGTGGGGAATATTGCACAATGGGCGAAAGCCTGATGCAGCGACGCCGCGTGAGGG
ATGACGGCCTTCGGGTTGTAAACCTCTTTCAGCAGGGAAGAAGCGAAAGTGACGGTACCTG
CAGAAGAAGCGCCGGCTAACTACGTGCCAGCAGCCGCGGTAATACAA

>BSNB_0235_ Bacillus_firmus

GGCGGCGTGCCTAATACATGCAAGTCGAGCGGAYTKMTGGGAGCTTGCTCCCTGAAGTCAG
CGGCGGACGGGTGAGTAACACGTGGGCAACCTGCCTGTAAGACTGGGATAACTCCGGGAA
ACCGGGGCTAATACCGGATAATTCTTTCCCTCACATGAGGGAAAGCTGAAAGATGGTTTCGG
CTATCACTTACAGATGGGCCCGCGGCGCATTAGCTAGTTGGTGAGGTAACGGCTCACCAAG
GCGACGATGCGTAGCCGACCTGAGAGGGTGATCGGCCACACTGGGACTGAGACACGGCCC
AGACTCCTACGGGAGGCAGCAGTAGGGAATCTTCCGCAATGGACGAAAGTCTGACGGAGC
AACGCCGCGTGAGTGATGAAGGTTTTCGGATCGTAAAACTCTGTTGTTAGGGAAGAACAAG
TACCGGAGTAACTGCCGGTACCTTGACGGTACCTAACCAGAAAGCCACGGCTAACTACGTG
CCAGCAGCCGCGGTAATAC

>BSNB_0236_ Bacillus_sp

TGCGGCGTGCCTAATACATGCAAGTCGAGCGAATGGATTAAGAGCTTGCTCTTATGAAGTTA
GCGGCGGACGGGTGAGTAACACGTGGGTAACCTGCCCATAAGACTGGGATAACTCCGGGA
AACCGGGGCTAATACCGGATAACATTTTGAACYGCATGGTTCGAAATTGAAAGGCGGCTTC
GGCTGTCACTTATGGATGGACCCGCGTCGCATTAGCTAGTTGGTGAGGTAACGGCTCACCAA
GGCAACGATGCGTAGCCGACCTGAGAGGGTGATCGGCCACACTGGGACTGAGACACGGCC
CAGACTCCTACGGGAGGCAGCAGTAGGGAATCTTCCGCAATGGACGAAAGTCTGACGGAGC
AACGCCGCGTGAGTGATGAAGGCTTTCGGGTCGTAAAACTCTGTTGTTAGGGAAGAACAAG
TGCTAGTTGAATAAGCTGGCACCTTGACGGTACCTAACCAGAAAGCCACGGCTAACTACGT
GCCAGCAGCCGCGGTAATACA

>BSNB_0239_ Lysinibacillus_sp

GGCGGCGTGCCTAATACATGCAAGTCGAGCGAACAGAAAAGGAGCTTGCTCCTTTGACGTT
AGCGGCGGACGGGTGAGTAACACGTGGGCAACCTACCTTATAGTTTGGGATAACTCCGGGA
AACCGGGGCTAATACCGAATAATCTATTTCACTTCATGGTGAAATACTGAAAGACGGCATCT
CGCTGTCGCTATAAGATGGGCCCGCGGCGCATTAGCTAGTTGGTGAGGTAACGGCTCACCA
AGGCGACAATGCGTAGCCAACCTGAGAGGGTGATCGGCCACACTGGGACTGAGACACGGC
CCAGACTCCTACGGGAGGCAGCAGTAGGGAATCTTCCACAATGGGCGAAAGCCTGATGGAG
CAACGCCCCGTGAGTGAAGAAGGTTTTCGGATCGTAAAACTCTGTTGTAAGGGAAGAACAA
GTACAGTAGTAACTGGCTGTACCTTGACGGTACCTTATTAGAAAGCCACGGCTAACTACGTG
CCAGCAGCCGCGGKTAATACA

>BSNB_0240_ Enterobacter_tabaci

GTGCTAACAATGCAAGTCGAAGCGGTATYACATAGKCCTTGCTTCCCTGGGAGTMRAGGGG

GGGAGGGGTGACCATGTGTGGGCCCACCTTTAAGATAACGGAAGCTTCCGGAATGTTGTCM
TCATACCCGGTAAACATCTCAACCCCAAGGAGGGGGACCGGWAATTGGTTTGCCTTCCWAT
GATCCATGACCGGACTSAGARGAAGGTTGGGCACAGGGTCACTGAAATAACAAGCCTACCT
TAGWAWGAGGTCATCACCAAMCAATTTTGAACTATGACCSAGYCATTGATCCCSCTGGAGG
CAACAGGAGGGAATCTTTATRGTATCCCTCTGGGCTAAGGAGGATAATGACATTACCTCTTG
ATTAATCCCGGGTTTTCTRSTGCCAAAMTCCGSKATGAAGGGGGAAAACTTRTKACRTTCATC
ATTTGMCTCTACTCGCAGCCCAAKCACCAAGCCCACTCCGTGCCACCKGCCGCRRAACCCC
AG

>BSNB_0242_ Serratia_marcescens

GGCGGCTGCCTAATACATGCAAGTCGAGCGAACAGAAAAGGAGCTTGCTCCTTTGACGTTA
GCGGCGGACGGGTGAGTAACACGTGGGCAACCTACCTTATAGTTTGGGATAACTCCGGGAA
ACCGGGGCTAATACCGAATAATCTATTTCACTTCATGGTGRAATACTGAAAGACGGCWTCTS
GYTKTCGYTWTAAAAKGGGCCCGCGGCGCATTASCTAKTTGGTGAGGTAACGGCTCACCARG
GCAACAATCCKTASCCAACCTGARAGGGTGATCGGCCACMCTGGGACTGAAACMCGGCCC
AAACYCCWACGGGAGGCASCAKTAGGGAATCTYCCMCAATGGGCRAARSCCTGATGGASCA
MCSCCCCGGGAKTRAAAAAGGTTTTCGGATCGWAAAACTCTTTTGTARGGRAAAAACAAKT
ACRGTAKTAMCTGGYTKTACCTTGACGGTMCCTTATWAAAARSCCMCGGCWAAYTACTTGC
CASCACCGCGGGTAATACA

>BSNB_0245_ Lysinibacillus_xylanilyticus

ACTATCATCAGCGGAGGAGYATMTCAATAAGCGGAGGAKSTWTCRMKAWGCGGRGGAKYG
YCTAAAKATKAGRGGGARAGGWCTTTAGGATGTTAACCTAGCTAAAGTCAGGCTTAGGCCT
GGTATCCTAATTCATTATTTACCAAAAGAATTCAGAATTAATTATTGTAACATAAGCGTAAA
AAACTTATAAAACAACTTTTAACAACGGATCTCTTGGTTCTCGCATCGATGAARAACGTAGC
AAAGTGCGATAACTAGTGTGAATTGCATATTCAGTGAATCATCGAGTCTTTGAACGCATCTT
GCGCTCAATGGTATTCCATTGAGCACGCCTGTTTCAGTATCAACAACAACCCACATCCRCAA
TTTTGTTGTGAATGGAAATGAGAGTAATCGACGTTAAAATTGAACTCTTTAAAATTATTAGG
CCTGAACTATTGTTCTTTTASCCTGAACATTAATTTTAATATAAAGGAATGCTCTAGTTATTAA
RACTGTCTTGGGGGCCTCCCAAATAAATCATTTTTTAAACTTGATCTGAATCAGGTGGGATTA
CCCGCTGAACTTAAGCATATCAKMGACGWCGGGAGGAAT

>BSNB_0246_ Burkholderia_sp

TGGGCGGCATGCCTTACACATGCAAGTCGAACGGCAGCACGGGGGCAACCCTGGTGGCGAG
TGGCGAACGGGTGAGTAATACATCGGAACGTGTCCTGTAGTGGGGGATAGCCCGGCGAAAG
CCGGATTAATACCGCATACGATCTACGGAAGAAAGCGGGGGATCTTCGGACCTCGCGCTGC
AGGGGCGGCCGATGGCAGATTAGCTAGTTGGTGGGGTAAAGGCCTACCAAGGCGACGATCT
GTAGCTGGTCTGAGAGGACGACCAGCCACACTGGGACTGAGACACGGCCCAGACTCCTACG
GGAGGCAGCAGTGGGGAATTTTGGACAATGGGGGCAACCCTGATCCAGCAATGCCGCGTGT
GTGAAGAAGGCCTTCGGGTTGTAAAGCACTTTTGTCCGGAAAGAAAACTTACCCGCTAATAT
CGGGTGGGGATGACGGTACCGGAAGAATAAGCACCGGCTAACTACGTGCCAGCAGCCGCG
GGTAATACA

>BSNB_0256_ Streptomyces_malaysiense

ACGTACGCTGGCGGCGTGCTTAACACATGCAAGTCGAACGATGAAGCCCTTCGGGGTGGAT
TAGTGGCGAACGGGTGAGTAACACGTGGGCAATCTGCCCTGCACTCTGGGACAAGCCCTGG
AAACGGGGTCTAATACCGGATATGACCGTCTTGGGCATCCTTGACGGTGTAAAGCTCCGGCG
GTGCAGGATGAGCCCGCGGCCTATCAGCTTGTTGGTGAGGTAACGGCTCACCAAGGCGACG

ACGGGTAGCCGGCCTGAGAGGGCGACCGGCCACACTGGGACTGAGACACGGCCCAGACTC
CTACGGGAGGCAGCAGTGGGGAATATTGCACAATGGGCGAAAGCCTGATGCAGCGACGCC
GCGTGAGGGATGACGGCCTTCGGGTTGTAAACCTCTTTCAGCAGGGAAGAAGCGAAAGTGA
CGGTACCTGCAGAAGAAGCGCCGGCTAACTACGTGCCAGCAGCCGCGGTAATACGTAGGGC
GCAAGCGTTGTCCGGAATTATTGGGCGTAAAGAGCTCGTAGGCGGCTTGTCACGTCGGTTGT
GAAAGCCCGGGGCTTAACCCCGGGTCTGCAGTCGATACGGGCAGGCTAGAGTTCGGTAGGG
GAGATCGGAATTCCTGGTGTAGCGGTGAAATGCGCAGATATCAGGAGGAACACCGGTGGCG
AAGGCGGATCTCTGGGCCGATACTGACGCTGAGGAGCGAAAGCGTGGGGAGCGAACAGGA
TTAGATACCCTGGTAGTCCACGCCGTAAACGGTGGGCACTAGGTGTGGGCAACATTCCACGT
TGTCCGTGCCGCAGCTAACGCATTAAGTGCCCCGCCTG
>BSNB_0493_ Paraburkholderia_tropica

GAACGCTGGCGGCATGCCTTACACATGCAAGTCGAACGGCAGCACGGGTGCTTGCACCTGG
TGGCGAGTGGCGAACGGGTGAGTAATACATCGGAACGTGTCCTGTAGTGGGGGATAGCCCG
GCGAAAGCCGGATTAATACCGCATACGATCTGCGGATGAAAGCGGGGGATCTTCGGACCTC
GCGCTATAGGGGCGGCCGATGGCGGATTAGCTAGTTGGTGGGGTAAAGGCTCACCAAGGCG
ACGATCCGTAGCTGGTCTGAGAGGACGACCAGCCACACTGGGACTGAGACACGGCCCAGA
CTCCTACGGGAGGCAGCAGTGGGGAATTTTGGACAATGGGCGAAAGCCTGATCCAGCAATG
CCGCGTGTGTGAAGAAGGCCTTCGGGTTGTAAAGCACTTTTGTCCGGAAAGAAATCCTTGGT
CCTAATATGGTCGGGGGATGACGGTACCGGAAGAATAAGCACCGGCTAACTACGTGCCAGC
AGCCGCGGTAATACGTAGGGTGCAAGCGTTAATCGGAATTACTGGGCGTAAAGCGTGCGCA
GGCGGTGATGTAAGACCGATGTGAAATCCCCGGGCTCAACCTGGGAACTGCATTGGTGACT
GCATCGCTTGAGTATGGCAGAGGGGGGTAGAATTCCACGTGTAGCAGTGAAATGCGTAGAG
ATGTGGAGGAATACCGATGGCGAAGGCAGCCCCCTGGGTCAATACTGACGCTCATGCACGA
AAGCGTGGGGAGCAAACAGGATTAGATACCCTGGTAGTCCACGCCCTAAACGATGT
>BSNB_0494_ Klebsiella_michiganensis

TTCATGGCTCAGATTGAACGCTGGCGGCAGGCCTAACACATGCAAGTCGAGCGGTAGCACG
GGGAGCTTGCTCCTGGGTGACGAGCGGCGGACGGGTGAGTAATGTCTGGGGATCTGCCTGA
TGGAGGGGGATAACTACTGGAAACGGTAGCTAATACCGCATAACGTCGCAAGACCAAAGA
GGGGGACCTTCGGGCCTCTTGCCATCAGATGAACCCAGATGGGATTAGCTAGTAGGTGGGG
TAATGGCTCACCTAGGCGACGATCCCTAGCTGGTCTGAGAGGATGACCAGCCACACTGGAA
CTGAGACACGGTCCAGACTCCTACGGGAGGCAGCAGTGGGGAATATTGCACAATGGGCGCA
AGCCTGATGCAGCCATGCCGCGTGTATGAAGAAGGCCTTCGGGTTGTAAAGTACTTTCAGCG
AGGAGGAAGGCGTTAAGGTTAATAACCTTGGCGATTGACGTTACTCGCAGAAGAAGCACCG
GCTAACTCCGTGCCAGCAGCCGCGGTAATACGGAGGGTGCAAGCGTTAATCGGAATTACTG
GGCGTAAAGCGCACGCAGGCGGTTTGTTAAGTCGGATGTGAAATCCCCGGGCTCAACCTGG
GAACTGCATTCGAAACTGGCAAGCTTGAGTCTTGTAGAGGGGGGTAGAATTCCAGGTGTAG
CGGTGAAATGCGTAGAGATCTGGAGGAATACCGGTGGCGAAGGCGGCCCCCTGGACAAAG
ACTGACGCTCAGGTGCGAAAGCGTGGGGAGCAAACAGGATTAGATACCCTGGTAGTCCACG
CCGTAAACGATGTCGACTTGGAGGTTGTGCCCTTGAGGCGTGGCTTCCGGAGCTAACGCGTT
AAGTCGACCGCCTG
>BSNB_0517_ Enterobacter_asburiae

GGTCGACTTAACGCGTTAGCTCCGGAAGCCACGCCTCAAGGGCACAACCTCCAAGTCGACA
TCGTTTACGGCGTGGACTACCAGGGTATCTAATCCTGTTTGCTCCCCACGCTTTCGCACCTGA
GCGTCAGTCTTTGTCCAGGGGGCCGCCTTCGCCACCGGTATTCCTCCAGATCTCTACGCATTT
CACCGCTACACCTGGAATTCTACCCCCCTCTACAAGACTCTAGCCTGCCAGTTTCGAATGCA
GTTCCCAGGTTGAGCCCGGGGATTTCACATCCGACTTGACAGACCGCCTGCGTGCGCTTTAC
GCCCAGTAATTCCGATTAACGCTTGCACCCTCCGTATTACCGCGGCTGCTGGCACGGAGTTA
GCCGGTGCTTCTTCTGCGGGTAACGTCAATCGACAAGGTTATTAACCTTATCGCCTTCCTCCC

CGCTGAAAGTACTTTACAACCCGAAGGCCTTCTTCATACACGCGGCATGGCTGCATCAGGCT
TGCGCCCATTGTGCAATATTCCCCACTGCTGCCTCCCGTAGGAGTCTGGACCGTGTCTCAGTT
CCAGTGTGGCTGGTCATCCTCTCAGACCAGCTAGGGATCGTCGCCTAGGTGAGCCGTTACCC
CACCTACTAGCTAATCCCATCTGGGCACATCTGATGGCAAGAGGCCCGAAGGTCCCCCTCTT
TGGTCTTGCGACGTTATGCGGTATTAGCTACCGTTTCCAGTAGTTATCCCCCTCCATCAGGCA
GTTTCCCAGACATTACTCACCCGTCCGCCGCTCGTCACCCAGGAGCAAGC

>BSNB_0519_ Burkholderia_tropica

TGATTCATGGCTCAGATTGAACGCTGGCGGCATGCCTTACACATGCAAGTCGAACGGCAGC
ACGGGTGCTTGCACCTGGTGGCGAGTGGCGAACGGGTGAGTAATACATCGGAACGTGTCCT
GTAGTGGGGGATAGCCCGGCGAAAGCCGGATTAATACCGCATACGATCTACGGATGAAAGC
GGGGGATCTTCGGACCTCGCGCTATAGGGGCGGCCGATGGCGGATTAGCTAGTTGGTGAGG
TAAAGGCTCACCAAGGCGACGATCCGTAGCTGGTCTGAGAGGACGACCAGCCACACTGGG
ACTGAGACACGGCCCAGACTCCTACGGGAGGCAGCAGTGGGGAATTTTGGACAATGGGCG
AAAGCCTGATCCAGCAATGCCGCGTGTGTGAAGAAGGCCTTCGGGTTGTAAAGCACTTTTGT
CCGGAAAGAAATCCTTGATCCTAATATGGTCGGGGGATGACGGTACCGGAAGAATAAGCAC
CGGCTAACTACGTGCCAGCAGCCGCGGTAATACGTAGGGTGCAAGCGTTAATCGGAATTAC
TGGGCGTAAAGCGTGCGCAGGCGGTGATGTAAGACCGATGTGAAATCCCCGGGCTCAACCT
GGGAACTGCATTGGTGACTGCATCGCTTGAGTATGGCAGAGGGGGGTAGAATTCCACGTGT
AGCAGTGAAATGCGTAGAGATGTGGAGGAATACCGATGGCGAAGGCAGCCCCCTGGGTCA
ATACTGACGCTCATGCACGAAAGCGTGGGGAGCAAACAGGATTAGATACCCTGGTAGTCCA
CGCCCTAAACGATGTCAACTGGTTGTCGGGTCTTCATTGACTTGGTAACGTAGCTAACGCGT
GAAGTGACCGCCTGG

>BSNB_0521_ Burkholderia_sp

ACTTCACGCGTTAGCTACGTTACCAAGTCAATGAAGACCCGACAACCAGTTCGACATCGTTT
AGGGCGTGGACTACCAGGGTATCTAATCCTGTTTGCTCCCCACGCTTTCGTGCATGAGCGTC
AGTATTGACCCAGGGGGCTGCCTTCGCCATCGGTATTCCTCCACATCTCTACGCATTTCACTG
CTACACGTGGAATTCTACCCCCCTCTGCCATACTCAAGCCTTGCAGTCACCAATGCAGTTCC
CAGGTTAAGCCCGGGGATTTCACATCGGTCTTACAAAACCGCCTGCGCACGCTTTACGCCCA
GTAATTCCGATTAACGCTCGCACCCTACGTATTACCGCGGCTGCTGGCACGTAGTTAGCCGG
TGCTTATTCTTCCGGTACCGTCATCCCCGCGGGATATTATCCAACAGGATTTCTTTCCGGACA
AAAGTGCTTTACAACCCGAAGGCCTTCTTCACACACGCGGCATTGCTGGATCAGGCTTTCGC
CCATTGTCCAAAATTCCCCACTGCTGCCTCCCGTAGGAGTCTGGGCCGTGTCTCAGTCCCAGT
GTGGCTGGTCGTCCTCTCAGACCAGCTACGGATCGTCGCCTTGGTAGGCCTTTACCCCACCA
ACTAGCTAATCCGCCATCGGCCACCCCAATAGCGCGAGGTCTTGCGATCCCCCGCTTTCATC
CAAAGATCGTATGCGGTATTAATCCGGCTTTCGCCGGGCTATCCCCCACTACTGGACATGTT
CCGATGTATTACTCACCCGTTCGCCACTCGCCACCAGGTGCAAGCACCCGTGCTGCCGTTCG
ACTTGCATGTGTAAGGCAT

>BSNB_0547_ Burkholderia_tropica

ACACATGCAAGTCGAACGGCAGCACGGGTGCTTGCACCTGGTGGCGAGTGGCGAACGGGTG
AGTAATACATCGGAACGTGTCCTGTAGTGGGGGATAGCCCGGCGAAAGCCGGATTAATACC
GCATACGATCTACGGATGAAAGCGGGGGATCTTCGGACCTCGCGCTATAGGGGCGGCCGAT
GGCGGATTAGCTAGTTGGTGGGGTAAAGGCTCACCAAGGCGACGATCCGTAGCTGGTCTGA
GAGGACGACCAGCCACACTGGGACTGAGACACGGCCCAGACTCCTACGGGAGGCAGCAGT
GGGGAATTTTGGACAATGGGCGAAAGCCTGATCCAGCAATGCCGCGTGTGTGAAGAAGGCC
TTCGGGTTGTAAAGCACTTTTGTCCGGAAAGAAATCCTTGATCCTAATATGGTCGGGGGATG
ACGGTACCGGAAGAATAAGCACCGGCTAACTACGTGCCAGCAGCCGCGGTAATACGTAGG

GTGCAAGCGTTAATCGGAATTACTGGGCGTAAAGCGTGCGCAGGCGGTGATGTAAGACCGA
TGTGAAATCCCCGGGCTCAACCTGGGAACTGCATTGGTGACTGCATCGCTTGAGTATGGCAG
AGGGGGGTAGAATTCCACGTGTAGCAGTGAAATGCGTAGAGATGTGGAGGAATACCGATGG
CGAAGGCAGCCCCCTGGGTCAATACTGACGCTCATGCACGAAAGCGTGGGGAGCAAACAG
GATTAGATACCCTGGTAGTCCACGCCCTAAACGATGTCAACTGGTTGTCGGGTCTTCATTGA
CTTGGTAACGTAGCTAACGCGTGAAGTGACCGCC

>BSNB_0561_ Bacillus_thuringiensis

GTGCTTAATGCGTTAACTTCAGCACTAAAGGGCGGAAACCCTCTAACACTTAGCACTCATCG
TTTACGGCGTGGACTACCAGGGTATCTAATCCTGTTTGCTCCCCACGCTTTCGCGCCTCAGTG
TCAGTTACAGACCAGAAAGTCGCCTTCGCCACTGGTGTTCCTCCATATCTCTACGCATTTCAC
CGCTACACATGGAATTCCACTTTCCTCTTCTGCACTCAAGTCTCCCAGTTTCCAATGACCCTC
CACGGTTGAGCCGTGGGCTTTCACATCAGACTTAAGAAACCACCTGCGCGCGCTTTACGCCC
AATAATTCCGGATAACGCTTGCCACCTACGTATTACCGCGGCTGCTGGCACGTAGTTAGCCG
TGGCTTTCTGGTTAGGTACCGTCAAGGTGCCAGCTTATTCAACTAGCACTTGTTCTTCCCTAA
CAACAGAGTTTTACGACCCGAAAGCCTTCATCACTCACGCGGCGTTGCTCCGTCAGACTTTC
GTCCATTGCGGAAGATTCCCTACTGCTGCCTCCCGTAGGAGTCTGGGCCGTGTCTCAGTCCC
AGTGTGGCCGATCACCCTCTCAGGTCGGCTACGCATCGTTGCCTTGGTGAGCCGTTACCTCA
CCAACTAGCTAATGCGACGCGGGTCCATCCATAAGTGACAGCCGAAGCCGCCTTTCAATTTC
GAACCATGCAGTTCAAAATGTTATCCGGTATTAGCCCCGGTTTCCCGGAGTTATCCCAGTCTT
ATGGGCAGGTTACCCACGTGTTACTCACCCGTCCGCCGCTAACTTCATAAGAGCAAGCTCTT
AATCCATTCGCTCGACTTGCATGTATTAGGCACGC

>BSNB_0562_ Paraburkholderia_tropica

ACTTCACGCGTTAGCTACGTTACTAAGGAAATGAATCCCCAACAACCAGTTGACATCGTTTA
GGGCGTGGACTACCAGGGTATCTAATCCTGTTTGCTCCCCACGCTTTCGTGCATGAGCGTCA
GTATTGGCCCAGGGGGCTGCCTTCGCCATCGGTATTCCTCCACATCTCTACGCATTTCACTGC
TACACGTGGAATTCTACCCCCCTCTGCCATACTCCAGCGATGCAGTCACCAATGCAGTTCCC
AGGTTGAGCCCGGGGATTTCACATCGGTCTTACATCACCGCCTGCGCACGCTTTACGCCCAG
TAATTCCGATTAACGCTTGCACCCTACGTATTACCGCGGCTGCTGGCACGTAGTTAGCCGGT
GCTTATTCTTCCGGTACCGTCATCCCCGACGGATATTAGCCATCAGGATTTCTTTCCGGACAA
AAGTGCTTTACAACCCGAAGGCCTTCTTCACACACGCGGCATTGCTGGATCAGGGTTTCCCC
CATTGTCCAAAATTCCCCACTGCTGCCTCCCGTAGGAGTCTGGGCCGTGTCTCAGTCCCAGTG
TGGCTGGTCGTCCTCTCAGACCAGCTACGGATCGTCGCCTTGGTGGGCCTTTACCCCACCAA
CTAGCTAATCCGCCATCGGCCGCCCCTATAGCGCGAGGTCCGAAGATCCCCCGCTTTCATCC
GTAGATCGTATGCGGTATTAATCCGGCTTTCGCCGGGCTATCCCCCACTACAGGACACGTTC
CGATGTATTACTCACCCGTTCGCCACTCGCCACCAGGTGCAAGCACCCGTGCTGCCGTTCGA
CTTGCATGTGTAAGGCATGCCGCCAGCGTTCAATC

>BSNB_0565_ Paraburkholderia_tropica

CATGCCTTACACATGCAAGTACGAACGGCAGCACGGGTGCTTGCACCTGGTGGCGAGTGGC
GAACGGGTGAGTAATACATCGGAACGTGTCCTGTAGTGGGGGATAGCCCGGCGAAAGCCGG
ATTAATACCGCATACGATCTACGGATGAAAGCGGGGGATCTTCGGACCTCGCGCTATAGGG
GCGGCCGATGGCGGATTAGCTAGTTGGTGGGGTAAAGGCCCACCAAGGCGACGATCCGTAG
CTGGTCTGAGAGGACGACCAGCCACACTGGGACTGAGACACGGCCCAGACTCCTACGGGA
GGCAGCAGTGGGGAATTTTGGACAATGGGGGAAACCCTGATCCAGCAATGCCGCGTGTGTG
AAGAAGGCCTTCGGGTTGTAAAGCACTTTTGTCCGGAAAGAAATCCTGATGGCTAATATCCG
TCGGGGATGACGGTACCGGAAGAATAAGCACCGGCTAACTACGTGCCAGCAGCCGCGGTA
ATACGTAGGGTGCAAGCGTTAATCGGAATTACTGGGCGTAAAGCGTGCGCAGGCGGTGATG

TAAGACCGATGTGAAATCCCCGGGCTCAACCTGGGAACTGCATTGGTGACTGCATCGCTGG
AGTATGGCAGAGGGGGGTAGAATTCCACGTGTAGCAGTGAAATGCGTAGAGATGTGGAGG
AATACCGATGGCGAAGGCAGCCCCCTGGGCCAATACTGACGCTCATGCACGAAAGCGTGGG
GAGCAAACAGGATTAGATACCCTGGTAGTCCACGCCCTAAACGATGTCAAGGGGTTGTTGG
GGATTCATTTCCTTAGTAACGTAGCTAACGCGTGA

>BSNB_0566_ Bacillus_subtilis

CGGAGTGCTTAATGCGTTAGCTGCAGCACTAAGGGGCGGAAACCCCCTAACACTTAGCACT
CATCGTTTACGGCGTGGACTACCAGGGTATCTAATCCTGTTCGCTCCCCACGCTTTCGCTCCT
CAGCGTCAGTTACAGACCAGAGAGTCGCCTTCGCCACTGGTGTTCCTCCACATCTCTACGCA
TTTCACCGCTACACGTGGAATTCCACTCTCCTCTTCTGCACTCAAGTTCCCCAGTTTCCAATG
ACCCTCCCCGGTTGAGCCGGGGGCTTTCACATCAGACTTAAGAAACCGCCTGCGAGCCCTTT
ACGCCCAATAATTCCGGACAACGCTTGCCACCTACGTATTACCGCGGCTGCTGGCACGTAGT
TAGCCGTGGCTTTCTGGTTAGGTACCGTCAAGGTACCGCCCTATTCGAACGGTACTTGTTCTT
CCCTAACAACAGAGCTTTACGATCCGAAAACCTTCATCACTCACGCGGCGTTGCTCCGTCAG
ACTTTCGTCCATTGCGGAAGATTCCCTACTGCTGCCTCCCGTAGGAGTCTGGGCCGTGTCTCA
GTCCCAGTGTGGCCGATCACCCTCTCAGGTCGGCTACGCATCGTTGCCTTGGTGAGCCGTTAC
CTCACCAACTAGCTAATGCGCCGCGGGTCCATCTGTAAGTGGTAGCCGAAGCCACCTTTTAT
GTTTGAACCATGCGGTTCAAACAACCATCCGGTATTAGCCCCGGTTTCCCGGAGTTATCCCA
GTCTTACAGGCAGGTTACCCACGTGTTACTCACCCGTCCGCCGCTAACATCAGGGAGCAAGC
TCCCATCTGTCCGCTCGACTTGCATGTATTAGGCACGCCGCCAGCGTTCGTC

>BSNB_0567_ Bacillus_subtilis

CGGAGTGCTTAATGCGTTAGCTGCAGCACTAAGGGGCGGAAACCCCCTAACACTTAGCACT
CATCGTTTACGGCGTGGACTACCAGGGTATCTAATCCTGTTCGCTCCCCACGCTTTCGCTCCT
CAGCGTCAGTTACAGACCAGAGAGTCGCCTTCGCCACTGGTGTTCCTCCACATCTCTACGCA
TTTCACCGCTACACGTGGAATTCCACTCTCCTCTTCTGCACTCAAGTTCCCCAGTTTCCAATG
ACCCTCCCCGGTTGAGCCGGGGGCTTTCACATCAGACTTAAGAAACCGCCTGCGAGCCCTTT
ACGCCCAATAATTCCGGACAACGCTTGCCACCTACGTATTACCGCGGCTGCTGGCACGTAGT
TAGCCGTGGCTTTCTGGTTAGGTACCGTCAAGGTACCGCCCTATTCGAACGGTACTTGTTCTT
CCCTAACAACAGAGCTTTACGATCCGAAAACCTTCATCACTCACGCGGCGTTGCTCCGTCAG
ACTTTCGTCCATTGCGGAAGATTCCCTACTGCTGCCTCCCGTAGGAGTCTGGGCCGTGTCTCA
GTCCCAGTGTGGCCGATCACCCTCTCAGGTCGGCTACGCATCGTTGCCTTGGTGAGCCGTTAC
CTCACCAACTAGCTAATGCGCCGCGGGTCCATCTGTAAGTGGTAGCCGAAGCCACCTTTTAT
GTTTGAACCATGCGGTTCAAACAACCATCCGGTATTAGCCCCGGTTTCCCGGAGTTATCCCA
GTCTTACAGGCAGGTTACCCACGTGTTACTCACCCGTCCGCCGCTAACATCAGGGAGCAAGC
TCCCATCTGTCCGCTCGACTTGCATGTATTAGGCACGCCGCCAGCGTTCGTC

>BSNB_0568_ Bacillus_subtilis

CGGAGTGCTTAATGCGTTAGCTGCAGCACTAAGGGGCGGAAACCCCCTAACACTTAGCACT
CATCGTTTACGGCGTGGACTACCAGGGTATCTAATCCTGTTCGCTCCCCACGCTTTCGCTCCT
CAGCGTCAGTTACAGACCAGAGAGTCGCCTTCGCCACTGGTGTTCCTCCACATCTCTACGCA
TTTCACCGCTACACGTGGAATTCCACTCTCCTCTTCTGCACTCAAGTTCCCCAGTTTCCAATG
ACCCTCCCCGGTTGAGCCGGGGGCTTTCACATCAGACTTAAGAAACCGCCTGCGAGCCCTTT
ACGCCCAATAATTCCGGACAACGCTTGCCACCTACGTATTACCGCGGCTGCTGGCACGTAGT
TAGCCGTGGCTTTCTGGTTAGGTACCGTCAAGGTACCGCCCTATTCGAACGGTACTTGTTCTT
CCCTAACAACAGAGCTTTACGATCCGAAAACCTTCATCACTCACGCGGCGTTGCTCCGTCAG
ACTTTCGTCCATTGCGGAAGATTCCCTACTGCTGCCTCCCGTAGGAGTCTGGGCCGTGTCTCA
GTCCCAGTGTGGCCGATCACCCTCTCAGGTCGGCTACGCATCGTTGCCTTGGTGAGCCGTTAC

CTCACCAACTAGCTAATGCGCCGCGGGTCCATCTGTAAGTGGTAGCCGAAGCCACCTTTTAT
GTTTGAACCATGCGGTTCAAACAACCATCCGGTATTAGCCCCGGTTTCCCGGAGTTATCCCA
GTCTTACAGGCAGGTTACCCACGTGTTACTCACCCGTCCGCCGCTAACATCAGGGAGCAAGC
TCCCATCTGTCCGCTCGACTTGCATGTATTAGGCACGCCGCCAGCGTTCGTC

>BSNB_0569_ Pantoea_dispersa

GGTCGACTTAACGCGTTAGCTCCGGAAGCCACGCCTCAAGGGCACAACCTCCAAGTCGACA
TCGTTTACGGCGTGGACTACCAGGGTATCTAATCCTGTTTGCTCCCCACGCTTTCGCACCTGA
GCGTCAGTCTTCGTCCAGGGGGCCGCCTTCGCCACCGGTATTCCTCCAGATCTCTACGCATTT
CACCGCTACACCTGGAATTCTACCCCCCTCTACGAGACTCAAGCCTGCCAGTTTCAAATGCA
GTTCCCAGGTTAAGCCCGGGGATTTCACATCTGACTTAACAGACCGCCTGCGTGCGCTTTAC
GCCCAGTAATTCCGATTAACGCTTGCACCCTCCGTATTACCGCGGCTGCTGGCACGGAGTTA
GCCGGTGCTTCTTCTGCGGGTAACGTCAATCGGCAAGGTTATTAACCTCACCGCCTTCCTCCC
CGCTGAAAGTACTTTACAACCCGAAGGCCTTCTTCATACACGCGGCATGGCTGCATCAGGCT
TGCGCCCATTGTGCAATATTCCCCACTGCTGCCTCCCGTAGGAGTCTGGACCGTGTCTCAGTT
CCAGTGTGGCTGGTCATCCTCTCAGACCAGCTAGGGATCGTCGCCTAGGTGAGCCATTACCC
CACCTACTAGCTAATCCCATCTGGGCACATCCGATGGTGTGAGGCCCGAAGGTCCCCCACTT
TGGTCTTGCGACGTTATGCGGTATTAGCTACCGTTTCCAGTAGTTATCCCCCTCCATCGGGCA
GTTTCCCAGACATTACTCACCCGTCCGCCACTCGCCACCCAAAGAGCAAGCTCTTCTGTGCT
GCCGTTCGACTTGCATGTGTTAGGCCTG

>BSNB_0570_ Burkholderia_tropica

GGCTCAGATTGAACGCTGGCGGCATGCCTTACACATGCAAGTCGAACGGCAGCACGGGTGC
TTGCACCTGGTGGCGAGTGGCGAACGGGTGAGTAATACATCGGAACGTGTCCTGTAGTGGG
GGATAGCCCGGCGAAAGCCGGATTAATACCGCATACGATCTAGGGATGAAAGCGGGGGAT
CTTCGGACCTCGCGCTATAGGGGCGGCCGATGGCGGATTAGCTAGTTGGTGGGGTAAAGGCT
CACCAAGGCGACGATCCGTAGCTGGTCTGAGAGGACGACCAGCCACACTGGGACTGAGAC
ACGGCCCAGACTCCTACGGGAGGCAGCAGTGGGGAATTTTGGACAATGGGCGAAAGCCTG
ATCCAGCAATGCCGCGTGTGTGAAGAAGGCCTTCGGGTTGTAAAGCACTTTTGTCCGGAAAG
AAATCCTTGATCCTAATATGGTCGGGGGATGACGGTACCGGAAGAATAAGCACCGGCTAAC
TACGTGCCAGCAGCCGCGGTAATACGTAGGGTGCAAGCGTTAATCGGAATTACTGGGCGTA
AAGCGTGCGCAGGCGGTGATGTAAGACCGATGTGAAATCCCCGGGCTCAACCTGGGAACTG
CATTGGTGACTGCATCGCTTGAGTATGGCAGAGGGGGGTAGAATTCCACGTGTAGCAGTGA
AATGCGTAGAGATGTGGAGGAATACCGATGGCGAAGGCAGCCCCCTGGGTCAATACTGACG
CTCATGCACGAAAGCGTGGGGAGCAAACAGGATTAGATACCCTGGTAGTCCACGCCCTAAA
CGATGTCAACTGGTTGTCGGGTCTTCATTGACTTGGTAACGTAGCTAACGCGTGAAGTGACC
GCCTG

>BSNB_0572_ Burkholderia_tropica

ACTTCACGCGTTAGCTACGTTACCAAGTCAATGAAGACCCGACAACCAGTTGACATCGTTTA
GGGCGTGGACTACCAGGGTATCTAATCCTGTTTGCTCCCCACGCTTTCGTGCATGAGCGTCA
GTATTGACCCAGGGGGCTGCCTTCGCCATCGGTATTCCTCCACATCTCTACGCATTTCACTGC
TACACGTGGAATTCTACCCCCCTCTGCCATACTCAAGCGATGCAGTCACCAATGCAGTTCCC
AGGTTGAGCCCGGGGATTTCACATCGGTCTTACATCACCGCCTGCGCACGCTTTACGCCCAG
TAATTCCGATTAACGCTTGCACCCTACGTATTACCGCGGCTGCTGGCACGTAGTTAGCCGGT
GCTTATTCTTCCGGTACCGTCATCCCCCGACCATATTAGGATCAAGGATTTCTTTCCGGACAA
AAGTGCTTTACAACCCGAAGGCCTTCTTCACACACGCGGCATTGCTGGATCAGGCTTTCGCC
CATTGTCCAAAATTCCCCACTGCTGCCTCCCGTAGGAGTCTGGGCCGTGTCTCAGTCCCAGTG
TGGCTGGTCGTCCTCTCAGACCAGCTACGGATCGTCGCCTTGGTGAGCCTTTACCCCACCAA
CTAGCTAATCCGCCATCGGCCGCCCCTATAGCGCGAGGTCCGAAGATCCCCCGCTTTCATCC

GTAGATCGTATGCGGTATTAATCCGGCTTTCGCCGGGCTATCCCCCACTACAGGACACGTTC
CGATGTATTACTCACCCGTTCGCCACTCGCCACCAGGTGCAAGCACCCGTGCTGCCGTTCGA
CTTGCATGTGT

>BSNB_0573_ Bacillus_subtilis

CGGAGTGCTTAATGCGTTAGCTGCAGCACTAAGGGGCGGAAACCCCCTAACACTTAGCACT
CATCGTTTACGGCGTGGACTACCAGGGTATCTAATCCTGTTCGCTCCCCACGCTTTCGCTCCT
CAGCGTCAGTTACAGACCAGAGAGTCGCCTTCGCCACTGGTGTTCCTCCACATCTCTACGCA
TTTCACCGCTACACGTGGAATTCCACTCTCCTCTTCTGCACTCAAGTTCCCCAGTTTCCAATG
ACCCTCCCCGGTTGAGCCGGGGGCTTTCACATCAGACTTAAGAAACCGCCTGCGAGCCCTTT
ACGCCCAATAATTCCGGACAACGCTTGCCACCTACGTATTACCGCGGCTGCTGGCACGTAGT
TAGCCGTGGCTTTCTGGTTAGGTACCGTCAAGGTACCGCCCTATTCGAACGGTACTTGTTCTT
CCCTAACAACAGAGCTTTACGATCCGAAAACCTTCATCACTCACGCGGCGTTGCTCCGTCAG
ACTTTCGTCCATTGCGGAAGATTCCCTACTGCTGCCTCCCGTAGGAGTCTGGGCCGTGTCTCA
GTCCCAGTGTGGCCGATCACCCTCTCAGGTCGGCTACGCATCGTTGCCTTGGTGAGCCGTTAC
CTCACCAACTAGCTAATGCGCCGCGGGTCCATCTGTAAGTGGTAGCCGAAGCCACCTTTTAT
GTTTGAACCATGCGGTTCAAACAACCATCCGGTATTAGCCCCGGTTTCCCGGAGTTATCCCA
GTCTTACAGGCAGGTTACCCACGTGTTACTCACCCGTCCGCCGCTAACATCAGGGAGCAAGC
TCCCATCTGTCCGCTCGACTTGCATGTATTAGGCACGCCGCCAGCGTTCGTC

>BSNB_0576_ Bacillus_subtilis

CGGAGTGCTTAATGCGTTAGCTGCAGCACTAAGGGGCGGAAACCCCCTAACACTTAGCACT
CATCGTTTACGGCGTGGACTACCAGGGTATCTAATCCTGTTCGCTCCCCACGCTTTCGCTCCT
CAGCGTCAGTTACAGACCAGAGAGTCGCCTTCGCCACTGGTGTTCCTCCACATCTCTACGCA
TTTCACCGCTACACGTGGAATTCCACTCTCCTCTTCTGCACTCAAGTTCCCCAGTTTCCAATG
ACCCTCCCCGGTTGAGCCGGGGGCTTTCACATCAGACTTAAGAAACCGCCTGCGAGCCCTTT
ACGCCCAATAATTCCGGACAACGCTTGCCACCTACGTATTACCGCGGCTGCTGGCACGTAGT
TAGCCGTGGCTTTCTGGTTAGGTACCGTCAAGGTACCGCCCTATTCGAACGGTACTTGTTCTT
CCCTAACAACAGAGCTTTACGATCCGAAAACCTTCATCACTCACGCGGCGTTGCTCCGTCAG
ACTTTCGTCCATTGCGGAAGATTCCCTACTGCTGCCTCCCGTAGGAGTCTGGGCCGTGTCTCA
GTCCCAGTGTGGCCGATCACCCTCTCAGGTCGGCTACGCATCGTTGCCTTGGTGAGCCGTTAC
CTCACCAACTAGCTAATGCGCCGCGGGTCCATCTGTAAGTGGTAGCCGAAGCCACCTTTTAT
GTTTGAACCATGCGGTTCAAACAACCATCCGGTATTAGCCCCGGTTTCCCGGAGTTATCCCA
GTCTTACAGGCAGGTTACCCACGTGTTACTCACCCGTCCGCCGCTAACATCAGGGAGCAAGC
TCCCATCTGTCCGCTCGACTTGCATGTATTAGGCACGCCGCCAGCGTTCGTC

>BSNB_0591_ Klebsiella_variicola

GATTCATGGCTCAGATTGAACGCTGGCGGCAGGCCTAACACATGCAAGTCGAGCGGTAGCA
CAGAGAGCTTGCTCTCGGGTGACGAGCGGCGGACGGGTGAGTAATGTCTGGGAAACTGCCT
GATGGAGGGGGATAACTACTGGAAACGGTAGCTAATACCGCATAACGTCGCAAGACCAAA
GTGGGGGACCTTCGGGCCTCATGCCATCAGATGTGCCCAGATGGGATTAGCTGGTAGGTGGG
GTAACGGCTCACCTAGGCGACGATCCCTAGCTGGTCTGAGAGGATGACCAGCCACACTGGA
ACTGAGACACGGTCCAGACTCCTACGGGAGGCAGCAGTGGGGAATATTGCACAATGGGCGC
AAGCCTGATGCAGCCATGCCGCGTGTGTGAAGAAGGCCTTCGGGTTGTAAAGCACTTTCAGC
GGGGAGGAAGGCGATAAGGTTAATAACCTCATCGATTGACGTTACCCGCAGAAGAAGCACC
GGCTAACTCCGTGCCAGCAGCCGCGGTAATACGGAGGGTGCAAGCGTTAATCGGAATTACT
GGGCGTAAAGCGCACGCAGGCGGTCTGTCAAGTCGGATGTGAAATCCCCGGGCTCAACCTG
GGAACTGCATTCGAAACTGGCAGGCTAGAGTCTTGTAGAGGGGGGTAGAATTCCAGGTGTA
GCGGTGAAATGCGTAGAGATCTGGAGGAATACCGGTGGCGAAGGCGGCCCCCTGGACAAA

GACTGACGCTCAGGTGCGAAAGCGTGGGGAGCAAACAGGATTAGATACCCTGGTAGTCCAC
GCTGTAAACGATGTCGATTTGGAGGTTGTGCCCTTGAGGCGTGGCTTCCGGAGCTAACGCGT
TAAATCGACCGC
>BSNB_0592_ Klebsiella_variicola

GATTCATGGCTCAGATTGAACGCTGGCGGCAGGCCTAACACATGCAAGTCGAGCGGTAGCA
CAGAGAGCTTGCTCTCGGGTGACGAGCGGCGGACGGGTGAGTAATGTCTGGGAAACTGCCT
GATGGAGGGGGATAACTACTGGAAACGGTAGCTAATACCGCATAACGTCGCAAGACCAAA
GTGGGGGACCTTCGGGCCTCATGCCATCAGATGTGCCCAGATGGGATTAGCTGGTAGGTGGG
GTAACGGCTCACCTAGGCGACGATCCCTAGCTGGTCTGAGAGGATGACCAGCCACACTGGA
ACTGAGACACGGTCCAGACTCCTACGGGAGGCAGCAGTGGGGAATATTGCACAATGGGCGC
AAGCCTGATGCAGCCATGCCGCGTGTGTGAAGAAGGCCTTCGGGTTGTAAAGCACTTTCAGC
GGGGAGGAAGGCGATAAGGTTAATAACCTCATCGATTGACGTTACCCGCAGAAGAAGCACC
GGCTAACTCCGTGCCAGCAGCCGCGGTAATACGGAGGGTGCAAGCGTTAATCGGAATTACT
GGGCGTAAAGCGCACGCAGGCGGTCTGTCAAGTCGGATGTGAAATCCCCGGGCTCAACCTG
GGAACTGCATTCGAAACTGGCAGGCTAGAGTCTTGTAGAGGGGGGTAGAATTCCAGGTGTA
GCGGTGAAATGCGTAGAGATCTGGAGGAATACCGGTGGCGAAGGCGGCCCCCTGGACAAA
GACTGACGCTCAGGTGCGAAAGCGTGGGGAGCAAACAGGATTAGATACCCTGGTAGTCCAC
GCTGTAAACGATGTCGATTTGGAGGTTGTGCCCTTGAGGCGTGGCTTCCGGAGCTAACGCGT
TAAATCGACCGC
>BSNB_0593_ Burkholderia_tropica

TCATGGCTCAGATTGAACGCTGGCGGCATGCCTTACACATGCAAGTCGAACGGCAGCACGG
GTGCTTGCACCTGGTGGCGAGTGGCGAACGGGTGAGTAATACATCGGAACGTGTCCTGTAGT
GGGGGATAGCCCGGCGAAAGCCGGATTAATACCGCATACGATCTACGGATGAAAGCGGGG
GATCTTCGGACCTCGCGCTATAGGGGCGGCCGATGGCGGATTAGCTAGTTGGTGAGGTAAA
GGCTCACCAAGGCGACGATCCGTAGCTGGTCTGAGAGGACGACCAGCCACACTGGGACTGA
GACACGGCCCAGACTCCTACGGGAGGCAGCAGTGGGGAATTTTGGACAATGGGCGAAAGC
CTGATCCAGCAATGCCGCGTGTGTGAAGAAGGCCTTCGGGTTGTAAAGCACTTTTGTCCGGA
AAGAAATCCTTGATCCTAATATGGTCGGGGGATGACGGTACCGGAAGAATAAGCACCGGCT
AACTACGTGCCAGCAGCCGCGGTAATACGTAGGGTGCAAGCGTTAATCGGAATTACTGGGC
GTAAAGCGTGCGCAGGCGGTGATGTAAGACCGATGTGAAATCCCCGGGCTCAACCTGGGAA
CTGCATTGGTGACTGCATCGCTTGAGTATGGCAGAGGGGGGTAGAATTCCACGTGTAGCAGT
GAAATGCGTAGAGATGTGGAGGAATACCGATGGCGAAGGCAGCCCCCTGGGTCAATACTG
ACGCTCATGCACGAAAGCGTGGGGAGCAAACAGGATTAGATACCCTGGTAGTCCACGCCCT
AAACGATGTCAACTGGTTGTCGGGTCTTCATTGACTTGGTAACGTAGCTAACGCGTGAAGTG
ACCGCC

>BSNB_0594_ Klebsiella_variicola

GATTCATGGCTCAGATTGAACGCTGGCGGCAGGCCTAACACATGCAAGTCGAGCGGTAGCA
CAGAGAGCTTGCTCTCGGGTGACGAGCGGCGGACGGGTGAGTAATGTCTGGGAAACTGCCT
GATGGAGGGGGATAACTACTGGAAACGGTAGCTAATACCGCATAACGTCGCAAGACCAAA
GTGGGGGACCTTCGGGCCTCATGCCATCAGATGTGCCCAGATGGGATTAGCTGGTAGGTGGG
GTAACGGCTCACCTAGGCGACGATCCCTAGCTGGTCTGAGAGGATGACCAGCCACACTGGA
ACTGAGACACGGTCCAGACTCCTACGGGAGGCAGCAGTGGGGAATATTGCACAATGGGCGC
AAGCCTGATGCAGCCATGCCGCGTGTGTGAAGAAGGCCTTCGGGTTGTAAAGCACTTTCAGC
GGGGAGGAAGGCGATAAGGTTAATAACCTCATCGATTGACGTTACCCGCAGAAGAAGCACC
GGCTAACTCCGTGCCAGCAGCCGCGGTAATACGGAGGGTGCAAGCGTTAATCGGAATTACT
GGGCGTAAAGCGCACGCAGGCGGTCTGTCAAGTCGGATGTGAAATCCCCGGGCTCAACCTG
GGAACTGCATTCGAAACTGGCAGGCTAGAGTCTTGTAGAGGGGGGTAGAATTCCAGGTGTA
GCGGTGAAATGCGTAGAGATCTGGAGGAATACCGGTGGCGAAGGCGGCCCCCTGGACAAA

GACTGACGCTCAGGTGCGAAAGCGTGGGGAGCAAACAGGATTAGATACCCTGGTAGTCCAC
GCTGTAAACGATGTCGATTTGGAGGTTGTGCCCTTGAGGCGTGGCTTCCGGAGCTAACGCGT
TAAATCGACCGC
>BSNB_0595_ Klebsiella_variicola

GATTCATGGCTCAGATTGAACGCTGGCGGCAGGCCTAACACATGCAAGTCGAGCGGTAGCA
CAGAGAGCTTGCTCTCGGGTGACGAGCGGCGGACGGGTGAGTAATGTCTGGGAAACTGCCT
GATGGAGGGGGATAACTACTGGAAACGGTAGCTAATACCGCATAACGTCGCAAGACCAAA
GTGGGGGACCTTCGGGCCTCATGCCATCAGATGTGCCCAGATGGGATTAGCTGGTAGGTGGG
GTAACGGCTCACCTAGGCGACGATCCCTAGCTGGTCTGAGAGGATGACCAGCCACACTGGA
ACTGAGACACGGTCCAGACTCCTACGGGAGGCAGCAGTGGGGAATATTGCACAATGGGCGC
AAGCCTGATGCAGCCATGCCGCGTGTGTGAAGAAGGCCTTCGGGTTGTAAAGCACTTTCAGC
GGGGAGGAAGGCGATAAGGTTAATAACCTCATCGATTGACGTTACCCGCAGAAGAAGCACC
GGCTAACTCCGTGCCAGCAGCCGCGGTAATACGGAGGGTGCAAGCGTTAATCGGAATTACT
GGGCGTAAAGCGCACGCAGGCGGTCTGTCAAGTCGGATGTGAAATCCCCGGGCTCAACCTG
GGAACTGCATTCGAAACTGGCAGGCTAGAGTCTTGTAGAGGGGGGTAGAATTCCAGGTGTA
GCGGTGAAATGCGTAGAGATCTGGAGGAATACCGGTGGCGAAGGCGGCCCCCTGGACAAA
GACTGACGCTCAGGTGCGAAAGCGTGGGGAGCAAACAGGATTAGATACCCTGGTAGTCCAC
GCTGTAAACGATGTCGATTTGGAGGTTGTGCCCTTGAGGCGTGGCTTCCGGAGCTAACGCGT
TAAATCGACCGC
>BSNB_0596_ Burkholderia_plantarii

ATTGAACGCTGGCGGCATGCCTTACACATGCAAGTCGAACGGCAGCACGGACTTCGGTCTG
GTGGCGAGTGGCGAACGGGTGAGTAATACATCGGAACATGTCCTGTAGTGGGGGATAGCCC
GGCGAAAGCCGGATTAATACCGCATACGATCTACGGATGAAAGCGGGGGATCTTCGGACCT
CGCGCTATAGGGTTGGCCGATGGCTGATTAGCTAGTTGGTAGGGTAAAAGCCTACCAAGGC
GACGATCAGTAGCTGGTCTGAGAGGACGACCAGCCACACTGGGACTGAGACACGGCCCAG
ACTCCTACGGGAGGCAGCAGTGGGGAATTTTGGACAATGGGCGAAAGCCTGATCCAGCAAT
GCCGCGTGTGTGAAGAAGGCCTTCGGGTTGTAAAGCACTTTTGTCCGGAAAGAAATCCTGAG
GGCTAATATCCTTCGGGGATGACGGTACCGGAAGAATAAGCACCGGCTAACTACGTGCCAG
CAGCCGCGGTAATACGTAGGGTGCGAGCGTTAATCGGAATTACTGGGCGTAAAGCGTGCGC
AGGCGGTTTGTTAAGACCGATGTGAAATCCCCGGGCTCAACCTGGGAACTGCATTGGTGACT
GGCAAGCTAGAGTATGGCAGAGGGGGGTAGAATTCCACGTGTAGCAGTGAAATGCGTAGA
GATGTGGAGGAATACCGATGGCGAAGGCAGCCCCCTGGGCCAATACTGACGCTCATGCACG
AAAGCGTGGGGAGCAAACAGGATTAGATACCCTGGTAGTCCACGCCCTAAACGATGTCAAC
TAGTTGTTGGGGATTCATTTCCTTAGTAACGTAGCTAACGCGTGAAGTGACCGC
>BSNB_0597_ Burkholderia_plantarii

ATTGAACGCTGGCGGCATGCCTTACACATGCAAGTCGAACGGCAGCACGGACTTCGGTCTG
GTGGCGAGTGGCGAACGGGTGAGTAATACATCGGAACATGTCCTGTAGTGGGGGATAGCCC
GGCGAAAGCCGGATTAATACCGCATACGATCTACGGATGAAAGCGGGGGATCTTCGGACCT
CGCGCTATAGGGTTGGCCGATGGCTGATTAGCTAGTTGGTAGGGTAAAAGCCTACCAAGGC
GACGATCAGTAGCTGGTCTGAGAGGACGACCAGCCACACTGGGACTGAGACACGGCCCAG
ACTCCTACGGGAGGCAGCAGTGGGGAATTTTGGACAATGGGCGAAAGCCTGATCCAGCAAT
GCCGCGTGTGTGAAGAAGGCCTTCGGGTTGTAAAGCACTTTTGTCCGGAAAGAAATCCTGAG
GGCTAATATCCTTCGGGGATGACGGTACCGGAAGAATAAGCACCGGCTAACTACGTGCCAG
CAGCCGCGGTAATACGTAGGGTGCGAGCGTTAATCGGAATTACTGGGCGTAAAGCGTGCGC
AGGCGGTTTGTTAAGACCGATGTGAAATCCCCGGGCTCAACCTGGGAACTGCATTGGTGACT
GGCAAGCTAGAGTATGGCAGAGGGGGGTAGAATTCCACGTGTAGCAGTGAAATGCGTAGA
GATGTGGAGGAATACCGATGGCGAAGGCAGCCCCCTGGGCCAATACTGACGCTCATGCACG

AAAGCGTGGGGAGCAAACAGGATTAGATACCCTGGTAGTCCACGCCCTAAACGATGTCAAC
TAGTTGTTGGGGATTCATTTCCTTAGTAACGTAGCTAACGCGTGAAGTGACCGC
>BSNB_0598_ Klebsiella_variicola

GATTCATGGCTCAGATTGAACGCTGGCGGCAGGCCTAACACATGCAAGTCGAGCGGTAGCA
CAGAGAGCTTGCTCTCGGGTGACGAGCGGCGGACGGGTGAGTAATGTCTGGGAAACTGCCT
GATGGAGGGGGATAACTACTGGAAACGGTAGCTAATACCGCATAACGTCGCAAGACCAAA
GTGGGGGACCTTCGGGCCTCATGCCATCAGATGTGCCCAGATGGGATTAGCTGGTAGGTGGG
GTAACGGCTCACCTAGGCGACGATCCCTAGCTGGTCTGAGAGGATGACCAGCCACACTGGA
ACTGAGACACGGTCCAGACTCCTACGGGAGGCAGCAGTGGGGAATATTGCACAATGGGCGC
AAGCCTGATGCAGCCATGCCGCGTGTGTGAAGAAGGCCTTCGGGTTGTAAAGCACTTTCAGC
GGGGAGGAAGGCGATAAGGTTAATAACCTCATCGATTGACGTTACCCGCAGAAGAAGCACC
GGCTAACTCCGTGCCAGCAGCCGCGGTAATACGGAGGGTGCAAGCGTTAATCGGAATTACT
GGGCGTAAAGCGCACGCAGGCGGTCTGTCAAGTCGGATGTGAAATCCCCGGGCTCAACCTG
GGAACTGCATTCGAAACTGGCAGGCTAGAGTCTTGTAGAGGGGGGTAGAATTCCAGGTGTA
GCGGTGAAATGCGTAGAGATCTGGAGGAATACCGGTGGCGAAGGCGGCCCCCTGGACAAA
GACTGACGCTCAGGTGCGAAAGCGTGGGGAGCAAACAGGATTAGATACCCTGGTAGTCCAC
GCTGTAAACGATGTCGATTTGGAGGTTGTGCCCTTGAGGCGTGGCTTCCGGAGCTAACGCGT
TAAATCGACCGC
>BSNB_0616_ Burkholderia_sp

TTCACGCGTTAGCTACGTTACTAAGGAAATGAATCCCCAACAACTAGTTGACATCGTTTAGG
GCGTGGACTACCAGGGTATCTAATCCTGTTTGCTCCCCACGCTTTCGTGCATGAGCGTCAGTA
TTGGCCCAGGGGGCTGCCTTCGCCATCGGTATTCCTCCACATCTCTACGCATTTCACTGCTAC
ACGTGGAATTCTACCCCCCTCTGCCATACTCAAAGCCTGCCAGTCACCAATGCAGTTCCCAG
GTTAAGCCCGGGGATTTCACATCGGTCTTAACAGACCGCCTGCGCACGCTTTACGCCCAGTA
ATTCCGATTAACGCTCGCACCCTACGTATTACCGCGGCTGCTGGCACGTAGTTAGCCGGTGC
TTATTCTTCCGGTACCGTCATCCCACCACCATATTAGGGCGATGGTTTTCTTTCCGGACAAAA
GTGCTTTACAACCCGAAGGCCTTCTTCACACACGCGGCATTGCTGGATCAGGGTTGCCCCCA
TTGTCCAAAATTCCCCACTGCTGCCTCCCGTAGGAGTCTGGGCCGTGTCTCAGTCCCAGTGTG
GCTGGTCGTCCTCTCAGACCAGCTACTGATCGTCGCCTTGGTAGGCCTTTACCCCACCAACTA
GCTAATCTGCCATCGGCCGCCCCTGCAGCGCGAGGTCCGAAGATCCCCCGCTTTCTTCCGTA
GATCGTATGCGGTATTAATCCGGCTTTCGCCGGGCTATCCCCCACTACAGGACAACGTTCCG
ATGTATTACTCACCCGTTC

>BSNB_0617_ Burkholderia_arvi

ACTTCACGCGTTAGCTACGTTACTAAGGAAATGAATCCCCAACAACTAGTTGACATCGTTTA
GGGCGTGGACTACCAGGGTATCTAATCCTGTTTGCTCCCCACGCTTTCGTGCATGAGCGTCA
GTGTTGGCCCAGGGGGCTGCCTTCGCCATCGGTATTCCTCCACATCTCTACGCATTTCACTGC
TACACGTGGAATTCTACCCCCCTCTGCCACACTCAAAGCCTGCCAGTCACCAATGCAGTTCC
CAGGTTAAGCCCGGGGATTTCACATCGGTCTTAACAGACCGCCTGCGCACGCTTTACGCCCA
GTAATTCCGATTAACGCTCGCACCCTACGTATTACCGCGGCTGCTGGCACGTAGTTAGCCGG
TGCTTATTCTTCCGGTACCGTCATCCCTCACGGATATTAGCCACGAGGTTTTCTTTCCGGACA
AAAGTGCTTTACAACCCGAAGGCCTTCTTCACACACGCGGCATTGCTGGATCAGGGTTGCCC
CCATTGTCCAAAATTCCCCACTGCTGCCTCCCGTAGGAGTCTGGGCCGTGTCTCAGTCCCAGT
GTGGCTGGTCGTCCTCTCAGACCAGCTACAGATCGTCGCCTTGGTAGGCCTTTACCCCACCA
ACTAGCTAATCTGCCATCGGCCGCCCCTATAGCGCGAGGTCCGAAGATCCCCCGCTTTCTCC
CTTAGGTCGTATGCGGTATTAATCCGGCTTTCGCCGGGCTATCCCCCACTACAGGACACGTTC
CGATGTATTACTCACCCGTTCGCCACTCGCCACCAGGGTTGCCCCCGTGCTGCCGTTCGACTT
GCATGTGTAAGGCAT

>BSNB_0618_ Bacillus_cereus

AAAGGCGGCTTCGGCTGTCACTTATGGATGGACCCGCGTCGCATTAGCTAGTTGGTGAGGTA
ACGGCTCACCAAGGCAACGATGCGTAGCCGACCTGAGAGGGTGATCGGCCACACTGGGACT
GAGACACGGCCCAGACTCCTACGGGAGGCAGCAGTAGGGAATCTTCCGCAATGGACGAAA
GTCTGACGGAGCAACGCCGCGTGAGTGATGAAGGCTTTCGGGTCGTAAAACTCTGTTGTTAG
GGAAGAACAAGTGCTAGTTGAATAAGCTGGCACCTTGACGGTACCTAACCAGAAAGCCACG
GCTAACTACGTGCCAGCAGCCGCGGTAATACGTAGGTGGCAAGCGTTATCCGGAATTATTG
GGCGTAAAGCGCGCGCAGGTGGTTTCTTAAGTCTGATGTGAAAGCCCACGGCTCAACCGTG
GAGGGTCATTGGAAACTGGGAGACTTGAGTGCAGAAGAGGAAAGTGGAATTCCATGTGTAG
CGGTGAAATGCGTAGAGATATGGAGGAACACCAGTGGCGAAGGCGACTTTCTGGTCTGTAA
CTGACACTGAGGCGCGAAAGCGTGGGGAGCAAACAGGATTAGATACCCTGGTAGTCCACGC
CGTAAACGA

>BSNB_0619_ Burkholderia_sp

CGTGGACTACCAGGGTATCTAATCCTGTTTGCTCCCCACGCTTTCGTGCATGAGCGTCAGTAT
TGGCCCAGGGGGCTGCCTTCGCCATCGGGATTCCTCCACATCTCTACGCATTTCACTGCTACA
CGTGGAATTCTACCCCCCTCTGCCATACTCAAAGCCTGCCAGTCACCAATGCAGTTCCCAGG
TTAGACCCGGGGATTTCACATCGGTCTTAACAAACCGCCTGCGCACGCTTTACGCCCAGTAA
TTCCGATTAACGCTCGCACCCTACGTATTACCGCGGCTGCTGGCACGTAATTAGCCGGTGCTT
ATTCTTCCGGTACCGTCATCCCACCACCATATTAGGGCGATGGTTTTCTTTCCGGACAAAAGT
GCTTTACAACCCGAAGGCCTTCTTCACACACGCGGCATTGCTGGATCAGGGTTGCCCCCATT
GTCCAAAATTCCCCACTGCTGCCTCCCGTAGGAGTCTGGGCCGTGTCTCAGTCCCAGTGTGG
CTGGTCGTCCTCTCAGACCAGCTACAGATCGTCGCCTTGGTAGGCCTTTACCCCACCAACTA
GCTAATCTGCCATCGGCCGCCCCTGCAGCGCGAGGTCCGAAGATCCCCCGCTTTCTTCCGTA
GATCGTATGCGGTATTAATCCGGCTTTCGCCGGGCTATCCCCCACTACAGGACACGTTCCGA
TGTATTACTCACCCGTTCGCCACTCGCCACCAGGGTTGCCCCCGTGCTGCCGTTCGACTTGCA
TGTGTAAGGCATGCCGCCAGCGTTCAATCTGAGCCATGAAT

>BSNB_0626_ Burkholderia_tropica

CCAGGCGGTCACTTCACGCGTTAGCTACGTTACCAAGTCAATGAAGACCCGACAACCAGTT
GACATCGTTTAGGGCGTGGACTACCAGGGTATCTAATCCTGTTTGCTCCCCACGCTTTCGTGC
ATGAGCGTCAGTATTGACCCAGGGGGCTGCCTTCGCCATCGGTATTCCTCCACATCTCTACG
CATTTCACTGCTACACGTGGAATTCTACCCCCCTCTGCCATACTCAAGCGATGCAGTCACCA
ATGCAGTTCCCAGGTTGAGCCCGGGGATTTCACATCGGTCTTACATCACCGCCTGCGCACGC
TTTACGCCCAGTAATTCCGATTAACGCTTGCACCCTACGTATTACCGCGGCTGCTGGCACGTA
GTTAGCCGGTGCTTATTCTTCCGGTACCGTCATCCCCCGACCATATTAGGATCAAGGATTTCT
TTCCGGACAAAAGTGCTTTACAACCCGAAGGCCTTCTTCACACACGCGGCATTGCTGGATCA
GGCTTTCGCCCATTGTCCAAAATTCCCCACTGCTGCCTCCCGTAGGAGTCTGGGCCGTGTCTC
AGTCCCAGTGTGGCTGGTCGTCCTCTCAGACCAGCTACGGATCGTCGCCTTGGTGAGCCTTTA
CCTCACCAACTAGCTAATCCGCCATCGGCCGCCCCTATAGCGCGAGGTCCGAAGATCCCCC
GCTTTCATCCGTAGATCGTATGCGGTATTAATCCGGCTTTCGCCGGGCTATCCCCCACTACAG
GACACGTTCCGATGTATTACTCACCCGTTCGCCACTCGCCACCAGGTGCAAGCACCCGTGCT
GCCGTTCGACTTGCATGTGTAAGGCATGCCGCCAGCGTTCAATCTGAGCCATGAATCA

>BSNB_0630_ Burkholderia_tropica

CCAGGCGGTCACTTCACGCGTTAGCTACGTTACCAAGTCAATGAAGACCCGACAACCAGTT
GACATCGTTTAGGGCGTGGACTACCAGGGTATCTAATCCTGTTTGCTCCCCACGCTTTCGTGC
ATGAGCGTCAGTATTGACCCAGGGGGCTGCCTTCGCCATCGGTATTCCTCCACATCTCTACG

CATTTCACTGCTACACGTGGAATTCTACCCCCCTCTGCCATACTCAAGCGATGCAGTCACCA
ATGCAGTTCCCAGGTTGAGCCCGGGGATTTCACATCGGTCTTACATCACCGCCTGCGCACGC
TTTACGCCCAGTAATTCCGATTAACGCTTGCACCCTACGTATTACCGCGGCTGCTGGCACGTA
GTTAGCCGGTGCTTATTCTTCCGGTACCGTCATCCCCCGACCATATTAGGATCAAGGATTTCT
TTCCGGACAAAAGTGCTTTACAACCCGAAGGCCTTCTTCACACACGCGGCATTGCTGGATCA
GGCTTTCGCCCATTGTCCAAAATTCCCCACTGCTGCCTCCCGTAGGAGTCTGGGCCGTGTCTC
AGTCCCAGTGTGGCTGGTCGTCCTCTCAGACCAGCTACGGATCGTCGCCTTGGTGAGCCTTTA
CCTCACCAACTAGCTAATCCGCCATCGGCCGCCCCTATAGCGCGAGGTCCGAAGATCCCCC
GCTTTCATCCGTAGATCGTATGCGGTATTAATCCGGCTTTCGCCGGGCTATCCCCCACTACAG
GACACGTTCCGATGTATTACTCACCCGTTCGCCACTCGCCACCAGGTGCAAGCACCCGTGCT
GCCGTTCGACTTGCATGTGTAAGGCATGCCGCCAGCGTTCAATCTGAGCCATGAATCA

>BSNB_0632_ Burkholderia_tropica

CCAGGCGGTCACTTCACGCGTTAGCTACGTTACCAAGTCAATGAAGACCCGACAACCAGTT
GACATCGTTTAGGGCGTGGACTACCAGGGTATCTAATCCTGTTTGCTCCCCACGCTTTCGTGC
ATGAGCGTCAGTATTGACCCAGGGGGCTGCCTTCGCCATCGGTATTCCTCCACATCTCTACG
CATTTCACTGCTACACGTGGAATTCTACCCCCCTCTGCCATACTCAAGCGATGCAGTCACCA
ATGCAGTTCCCAGGTTGAGCCCGGGGATTTCACATCGGTCTTACATCACCGCCTGCGCACGC
TTTACGCCCAGTAATTCCGATTAACGCTTGCACCCTACGTATTACCGCGGCTGCTGGCACGTA
GTTAGCCGGTGCTTATTCTTCCGGTACCGTCATCCCCCGACCATATTAGGATCAAGGATTTCT
TTCCGGACAAAAGTGCTTTACAACCCGAAGGCCTTCTTCACACACGCGGCATTGCTGGATCA
GGCTTTCGCCCATTGTCCAAAATTCCCCACTGCTGCCTCCCGTAGGAGTCTGGGCCGTGTCTC
AGTCCCAGTGTGGCTGGTCGTCCTCTCAGACCAGCTACGGATCGTCGCCTTGGTGAGCCTTTA
CCTCACCAACTAGCTAATCCGCCATCGGCCGCCCCTATAGCGCGAGGTCCGAAGATCCCCC
GCTTTCATCCGTAGATCGTATGCGGTATTAATCCGGCTTTCGCCGGGCTATCCCCCACTACAG
GACACGTTCCGATGTATTACTCACCCGTTCGCCACTCGCCACCAGGTGCAAGCACCCGTGCT
GCCGTTCGACTTGCATGTGTAAGGCATGCCGCCAGCGTTCAATCTGAGCCATGAATCA

>BSNB_0635_ Paraburkholderia_guartelaensis

AACCAGTTGACATCGTTTAGGGCGTGGACTACCAGGGTATCTAATCCTGTTTGCTCCCCACG
CTTTCGTGCATGAGCGTCAGTATTGGCCCAGGGGGCTGCCTTCGCCATCGGTATTCCTCCACA
TCTCTACGCATTTCACTGCTACACGTGGAATTCTACCCCCCTCTGCCATACTCCAGCGATGCA
GTCACCAATGCAGTTCCCAGGTTAAGCCCGGGGATTTCACATCGGTCTTACATCACCGCCTG
CGCACGCTTTACGCCCAGTAATTCCGATTAACGCTTGCACCCTACGTATTACCGCGGCTGCTG
GCACGTAGTTAGCCGGTGCTTATTCTTCCGGTACCGTCATCCCACCCGGATATTAGCCAGGC
GGTTTTCTTTCCGGACAAAAGTGCTTTACAACCCGAAGGCCTTCTTCACACACGCGGCATTG
CTGGATCAGGGTTGCCCCCATTGTCCAAAATTCCCCACTGCTGCCTCCCGTAGGAGTCTGGG
CCGTGTCTCAGTCCCAGTGTGGCTGGTCGTCCTCTCAGACCAGCTACGGATCGTCGCCTTGGT
GGGCCTTTACCCCACCAACTAGCTAATCCGCCATCGG
>BSNB_0636_ Burkholderia_sp

CGGTCACTTCACGCGTTAGCTACGTTACCAAGTCAATGAAGACCCGACAACCAGTTGACATC
GTTTAGGGCGTGGACTACCAGGGTATCTAATCCTGTTTGCTCCCCACGCTTTCGTGCATGAGC
GTCAGTATTGGCCCAGGGGGCTGCCTTCGCCATCGGTATTCCTCCACATCTCTACGCATTTCA
CTGCTACACGTGGAATTCTACCCCCCTCTGCCATACTCCAGCGATGCAGTCACCAATGCAGT
TCCCAGGTTAAGCCCGGGGATTTCACATCGGTCTTACATCACCGCCTGCGCACGCTTTACGC
CCAGTAATTCCGATTAACGCTTGCACCCTACGTATTACCGCGGCTGCTGGCACGTAGTTAGC
CGGTGCTTATTCTTCCGGTACCGTCATCCCACCCGGATATTAGCCAGGCGGTTTTCTTTCCGG
ACAAAAGTGCTTTACAACCCGAAGGCCTTCTTCACACACGCGGCATTGCTGGATCAGGGTTG
CCCCCATTGTCCAAAATTCCCCACTGCTGCCTCCCGTAGGAGTCTGGGCCGTGTCTCAGTCCC

AGTGTGGCTGGTCGTCCTCTCAGACCAGCTACGGATCGTCGCCTTGGTGGGCCTTTACCCCAC
CAACTAGCTAATCCGCCATCGGCCACCCCAATAGCGCGAGGTCTTTCGATCCCCCGCTTTCC
TCCACAGAGCGTATGCGGTATTAATCCGGCTTTCGCCGGGCTATCCCCCACTACTGGACATG
TTCCGATGTATTACTCACCCGTTCGCCACTCGCCACCAGGTGCAAGCACCCGTGCTGCCGTTC
GACTTGCATGTGTAAGGCATGCCGCCAGCGTTCAATCT

>BSNB_0639_ Burkholderia_tropica

CCAGGCGGTCACTTCACGCGTTAGCTACGTTACCAAGTCAATGAAGACCCGACAACCAGTT
GACATCGTTTAGGGCGTGGACTACCAGGGTATCTAATCCTGTTTGCTCCCCACGCTTTCGTGC
ATGAGCGTCAGTATTGACCCAGGGGGCTGCCTTCGCCATCGGTATTCCTCCACATCTCTACG
CATTTCACTGCTACACGTGGAATTCTACCCCCCTCTGCCATACTCAAGCGATGCAGTCACCA
ATGCAGTTCCCAGGTTGAGCCCGGGGATTTCACATCGGTCTTACATCACCGCCTGCGCACGC
TTTACGCCCAGTAATTCCGATTAACGCTTGCACCCTACGTATTACCGCGGCTGCTGGCACGTA
GTTAGCCGGTGCTTATTCTTCCGGTACCGTCATCCCCCGACCATATTAGGATCAAGGATTTCT
TTCCGGACAAAAGTGCTTTACAACCCGAAGGCCTTCTTCACACACGCGGCATTGCTGGATCA
GGCTTTCGCCCATTGTCCAAAATTCCCCACTGCTGCCTCCCGTAGGAGTCTGGGCCGTGTCTC
AGTCCCAGTGTGGCTGGTCGTCCTCTCAGACCAGCTACGGATCGTCGCCTTGGTGAGCCTTTA
CCTCACCAACTAGCTAATCCGCCATCGGCCGCCCCTATAGCGCGAGGTCCGAAGATCCCCC
GCTTTCATCCGTAGATCGTATGCGGTATTAATCCGGCTTTCGCCGGGCTATCCCCCACTACAG
GACACGTTCCGATGTATTACTCACCCGTTCGCCACTCGCCACCAGGTGCAAGCACCCGTGCT
GCCGTTCGACTTGCATGTGTAAGGCATGCCGCCAGCGTTCAATCTGAGCCATGAATCA

>BSNB_0640_ Burkholderia_oxyphila

CGGTCACTTCACGCGTTAGCTACGTTACCAAGTCAATGAAGACCCGACAACCAGTTGACATC
GTTTAGGGCGTGGACTACCAGGGTATCTAATCCTGTTTGCTCCCCACGCTTTCGTGCATGAGC
GTCAGTATTGGCCCAGGGGGCTGCCTTCGCCATCGGTATTCCTCCACATCTCTACGCATTTCA
CTGCTACACGTGGAATTCTACCCCCCTCTGCCATACTCCAGCGATGCAGTCACCAATGCAGT
TCCCAGGTTAAGCCCGGGGATTTCACATCGGTCTTACATCACCGCCTGCGCACGCTTTACGC
CCAGTAATTCCGATTAACGCTTGCACCCTACGTATTACCGCGGCTGCTGGCACGTAGTTAGC
CGGTGCTTATTCTTCCGGTACCGTCATCCCACCCGGATATTAGCCAGGCGGTTTTCTTTCCGG
ACAAAAGTGCTTTACAACCCGAAGGCCTTCTTCACACACGCGGCATTGCTGGATCAGGGTTG
CCCCCATTGTCCAAAATTCCCCACTGCTGCCTCCCGTAGGAGTCTGGGCCGTGTCTCAGTCCC
AGTGTGGCTGGTCGTCCTCTCAGACCAGCTACGGATCGTCGCCTTGGTGGGCCTTTACCCCAC
CAACTAGCTAATCCGCCATCGGCCACCCCAATAGCGCGAGGTCTTTCGATCCCCCGCTTTCC
TCCACAGAGCGTATGCGGTATTAATCCGGCTTTCGCCGGGCTATCCCCCACTACTGGACATG
TTCCGATGTATTACTCACCCGTTCGCCACTCGCCACCAGGTGCAAGCACCCGTGCTGCCGTTC
GACTTGCATGTGTAAGGCATGCCGCCAGCGTTCAATCT

>BSNB_0643_ Paenibacillus_glucanolyticus

GCGGATGCTTAATGTGTTAACTTCGGCACCAAGGGTATCGAAACCCCTAACACCTAGCATTC
ATCGTTTACGGCGTGGACTACCAGGGTATCTAATCCTGTTTGCTCCCCACGCTTTCGCGCCTC
AGCGTCAGTTACAGCCCAGAGAGTCGCCTTCGCCACTGGTGTTCCTCCACATATCTACGCAT
TTCACCGCTACACGTGGAATTCCACTCTCCTCTTCTGCACTCAAGTTCCCCAGTTTCCAGTGC
GACCTGAAGTTGAGCCCCAGGTTTAAACACCAGACTTAAAGAACCGCCTGCGCGCGCTTTA
CGCCCAATAATTCCGGACAACGCTTGCCCCCTACGTATTACCGCGGCTGCTGGCACGTAGTT
AGCCGGGGCTTTCTTCTCAAGTACCGTCACTCTCATAGCAGTTACTCTATGAGACGTTCTTCC
TTGGCAACAGAGCTTTACGATCCGAAAACCTTCATCACTCACGCGGCGTTGCTCCGTCAGGC
TTTCGCCCATTGCGGAAGATTCCCTACTGCTGCCTCCCGTAGGAGTCTGGGCCGTGTCTCAGT
CCCAGTGTGGCCGTTCACCCTCTCAGGTCGGCTACGCATCGTCGCCTTGGTGGGCCGTTACCC

CACCAACTAGCTAATGCGCCGCAGGCCCATCCCCAAGTGACAGATTGCTCCGTCTTTCATTA
TCACATAATGCTATGTAATAAATTATCCGGTATTAGCTACCGTTTCCGGTAGTTATCCCAGTC
TTGAGGGCAGGTTGCCTACGTGTTACTCACCCGTCCGCCGCTAACTCTCAGGAGTGCAAGCA
CTCCTTCAAGTCCGCTCGACTTGCATGTATTAGGCACGCCGCCAGCGTTCGT

>BSNB_0648_ Bacillus_sp

CGGTCACTTCACGCGTTAGCTACGTTACCAAGTCAATGAAGACCCGACAACCAGTTGACATC
GTTTAGGGCGTGGACTACCAGGGTATCTAATCCTGTTTGCTCCCCACGCTTTCGTGCATGAGC
GTCAGTATTGGCCCAGGGGGCTGCCTTCGCCATCGGTATTCCTCCACATCTCTACGCATTTCA
CTGCTACACGTGGAATTCTACCCCCCTCTGCCATACTCCAGCGATGCAGTCACCAATGCAGT
TCCCAGGTTAAGCCCGGGGATTTCACATCGGTCTTACATCACCGCCTGCGCACGCTTTACGC
CCAGTAATTCCGATTAACGCTTGCACCCTACGTATTACCGCGGCTGCTGGCACGTAGTTAGC
CGGTGCTTATTCTTCCGGTACCGTCATCCCACCCGGATATTAGCCAGGCGGTTTTCTTTCCGG
ACAAAAGTGCTTTACAACCCGAAGGCCTTCTTCACACACGCGGCATTGCTGGATCAGGGTTG
CCCCCATTGTCCAAAATTCCCCACTGCTGCCTCCCGTAGGAGTCTGGGCCGTGTCTCAGTCCC
AGTGTGGCTGGTCGTCCTCTCAGACCAGCTACGGATCGTCGCCTTGGTGGGCCTTTACCCCAC
CAACTAGCTAATCCGCCATCGGCCACCCCAATAGCGCGAGGTCTTTCGATCCCCCGCTTTCC
TCCACAGAGCGTATGCGGTATTAATCCGGCTTTCGCCGGGCTATCCCCCACTACTGGACATG
TTCCGATGTATTACTCACCCGTTCGCCACTCGCCACCAGGTGCAAGCACCCGTGCTGCCGTTC
GACTTGCATGTGTAAGGCATGCCGCCAGCGTTCAATCT

>BSNB_0656_ Burkholderia_tropica

CCAGGCGGTCACTTCACGCGTTAGCTACGTTACCAAGTCAATGAAGACCCGACAACCAGTT
GACATCGTTTAGGGCGTGGACTACCAGGGTATCTAATCCTGTTTGCTCCCCACGCTTTCGTGC
ATGAGCGTCAGTATTGACCCAGGGGGCTGCCTTCGCCATCGGTATTCCTCCACATCTCTACG
CATTTCACTGCTACACGTGGAATTCTACCCCCCTCTGCCATACTCAAGCGATGCAGTCACCA
ATGCAGTTCCCAGGTTGAGCCCGGGGATTTCACATCGGTCTTACATCACCGCCTGCGCACGC
TTTACGCCCAGTAATTCCGATTAACGCTTGCACCCTACGTATTACCGCGGCTGCTGGCACGTA
GTTAGCCGGTGCTTATTCTTCCGGTACCGTCATCCCCCGACCATATTAGGATCAAGGATTTCT
TTCCGGACAAAAGTGCTTTACAACCCGAAGGCCTTCTTCACACACGCGGCATTGCTGGATCA
GGCTTTCGCCCATTGTCCAAAATTCCCCACTGCTGCCTCCCGTAGGAGTCTGGGCCGTGTCTC
AGTCCCAGTGTGGCTGGTCGTCCTCTCAGACCAGCTACGGATCGTCGCCTTGGTGAGCCTTTA
CCTCACCAACTAGCTAATCCGCCATCGGCCGCCCCTATAGCGCGAGGTCCGAAGATCCCCC
GCTTTCATCCGTAGATCGTATGCGGTATTAATCCGGCTTTCGCCGGGCTATCCCCCACTACAG
GACACGTTCCGATGTATTACTCACCCGTTCGCCACTCGCCACCAGGTGCAAGCACCCGTGCT
GCCGTTCGACTTGCATGTGTAAGGCATGCCGCCAGCGTTCAATCTGAGCCATGAATCA

>BSNB_0658_ Burkholderia_tropica

CCAGGCGGTCACTTCACGCGTTAGCTACGTTACCAAGTCAATGAAGACCCGACAACCAGTT
GACATCGTTTAGGGCGTGGACTACCAGGGTATCTAATCCTGTTTGCTCCCCACGCTTTCGTGC
ATGAGCGTCAGTATTGACCCAGGGGGCTGCCTTCGCCATCGGTATTCCTCCACATCTCTACG
CATTTCACTGCTACACGTGGAATTCTACCCCCCTCTGCCATACTCAAGCGATGCAGTCACCA
ATGCAGTTCCCAGGTTGAGCCCGGGGATTTCACATCGGTCTTACATCACCGCCTGCGCACGC
TTTACGCCCAGTAATTCCGATTAACGCTTGCACCCTACGTATTACCGCGGCTGCTGGCACGTA
GTTAGCCGGTGCTTATTCTTCCGGTACCGTCATCCCCCGACCATATTAGGATCAAGGATTTCT
TTCCGGACAAAAGTGCTTTACAACCCGAAGGCCTTCTTCACACACGCGGCATTGCTGGATCA
GGCTTTCGCCCATTGTCCAAAATTCCCCACTGCTGCCTCCCGTAGGAGTCTGGGCCGTGTCTC
AGTCCCAGTGTGGCTGGTCGTCCTCTCAGACCAGCTACGGATCGTCGCCTTGGTGAGCCTTTA
CCTCACCAACTAGCTAATCCGCCATCGGCCGCCCCTATAGCGCGAGGTCCGAAGATCCCCC

GCTTTCATCCGTAGATCGTATGCGGTATTAATCCGGCTTTCGCCGGGCTATCCCCCACTACAG
GACACGTTCCGATGTATTACTCACCCGTTCGCCACTCGCCACCAGGTGCAAGCACCCGTGCT
GCCGTTCGACTTGCATGTGTAAGGCATGCCGCCAGCGTTCAATCTGAGCCATGAATCA

>BSNB_0659_ Bacillus_subtilis

GGCTCAGGACGAACGCTGGCGGCGTGCCTAATACATGCAAGTCGAGCGGACAGATGGGAG
CTTGCTCCCTGATGTTAGCGGCGGACGGGTGAGTAACACGTGGGTAACCTGCCTGTAAGACT
GGGATAACTCCGGGAAACCGGGGCTAATACCGGATGGTTGTTTGAACCGCATGGTTCAAAC
ATAAAAGGTGGCTTCGGCTACCACTTACAGATGGACCCGCGGCGCATTAGCTAGTTGGTGA
GGTAACGGCTCACCAAGGCAACGATGCGTAGCCGACCTGAGAGGGTGATCGGCCACACTGG
GACTGAGACACGGCCCAGACTCCTACGGGAGGCAGCAGTAGGGAATCTTCCGCAATGGAC
GAAAGTCTGACGGAGCAACGCCGCGTGAGTGATGAAGGTTTTCGGATCGTAAAGCTCTGTT
GTTAGGGAAGAACAAGTACCGTTCGAATAGGGCGGTACCTTGACGGTACCTAACCAGAAAG
CCACGGCTAACTACGTGCCAGCAGCCGCGGTAATACGTAGGTGGCAAGCGTTGTCCGGAAT
TATTGGGCGTAAAGGGCTCGCAGGCGGTTTCTTAAGTCTGATGTGAAAGCCCCCGGCTCAAC
CGGGGAGGGTCATTGGAAACTGGGGAACTTGAGTGCAGAAGAGGAGAGTGGAATTCCACG
TGTAGCGGTGAAATGCGTAGAGATGTGGAGGAACACCAGTGGCGAAGGCGACTCTCTGGTC
TGTAACTGACGCTGAGGAGCGAAAGCGTGGGGAGCGAACAGGATTAGATACCCTGGTAGTC
CACGCCGTAAACGATGAGTGCTAAGTGTTAGGGGGTTTCCGCCCCTTAGTGCTGCAGCTAAC
GCATTAAGCACTCCGCC
>BSNB_0661_ Burkholderia_tropica

CCAGGCGGTCACTTCACGCGTTAGCTACGTTACCAAGTCAATGAAGACCCGACAACCAGTT
GACATCGTTTAGGGCGTGGACTACCAGGGTATCTAATCCTGTTTGCTCCCCACGCTTTCGTGC
ATGAGCGTCAGTATTGACCCAGGGGGCTGCCTTCGCCATCGGTATTCCTCCACATCTCTACG
CATTTCACTGCTACACGTGGAATTCTACCCCCCTCTGCCATACTCAAGCGATGCAGTCACCA
ATGCAGTTCCCAGGTTGAGCCCGGGGATTTCACATCGGTCTTACATCACCGCCTGCGCACGC
TTTACGCCCAGTAATTCCGATTAACGCTTGCACCCTACGTATTACCGCGGCTGCTGGCACGTA
GTTAGCCGGTGCTTATTCTTCCGGTACCGTCATCCCCCGACCATATTAGGATCAAGGATTTCT
TTCCGGACAAAAGTGCTTTACAACCCGAAGGCCTTCTTCACACACGCGGCATTGCTGGATCA
GGCTTTCGCCCATTGTCCAAAATTCCCCACTGCTGCCTCCCGTAGGAGTCTGGGCCGTGTCTC
AGTCCCAGTGTGGCTGGTCGTCCTCTCAGACCAGCTACGGATCGTCGCCTTGGTGAGCCTTTA
CCTCACCAACTAGCTAATCCGCCATCGGCCGCCCCTATAGCGCGAGGTCCGAAGATCCCCC
GCTTTCATCCGTAGATCGTATGCGGTATTAATCCGGCTTTCGCCGGGCTATCCCCCACTACAG
GACACGTTCCGATGTATTACTCACCCGTTCGCCACTCGCCACCAGGTGCAAGCACCCGTGCT
GCCGTTCGACTTGCATGTGTAAGGCATGCCGCCAGCGTTCAATCTGAGCCATGAATCA

>BSNB_0663_ Burkholderia_tropica

CCAGGCGGTCACTTCACGCGTTAGCTACGTTACCAAGTCAATGAAGACCCGACAACCAGTT
GACATCGTTTAGGGCGTGGACTACCAGGGTATCTAATCCTGTTTGCTCCCCACGCTTTCGTGC
ATGAGCGTCAGTATTGACCCAGGGGGCTGCCTTCGCCATCGGTATTCCTCCACATCTCTACG
CATTTCACTGCTACACGTGGAATTCTACCCCCCTCTGCCATACTCAAGCGATGCAGTCACCA
ATGCAGTTCCCAGGTTGAGCCCGGGGATTTCACATCGGTCTTACATCACCGCCTGCGCACGC
TTTACGCCCAGTAATTCCGATTAACGCTTGCACCCTACGTATTACCGCGGCTGCTGGCACGTA
GTTAGCCGGTGCTTATTCTTCCGGTACCGTCATCCCCCGACCATATTAGGATCAAGGATTTCT
TTCCGGACAAAAGTGCTTTACAACCCGAAGGCCTTCTTCACACACGCGGCATTGCTGGATCA
GGCTTTCGCCCATTGTCCAAAATTCCCCACTGCTGCCTCCCGTAGGAGTCTGGGCCGTGTCTC
AGTCCCAGTGTGGCTGGTCGTCCTCTCAGACCAGCTACGGATCGTCGCCTTGGTGAGCCTTTA
CCTCACCAACTAGCTAATCCGCCATCGGCCGCCCCTATAGCGCGAGGTCCGAAGATCCCCC
GCTTTCATCCGTAGATCGTATGCGGTATTAATCCGGCTTTCGCCGGGCTATCCCCCACTACAG

GACACGTTCCGATGTATTACTCACCCGTTCGCCACTCGCCACCAGGTGCAAGCACCCGTGCT
GCCGTTCGACTTGCATGTGTAAGGCATGCCGCCAGCGTTCAATCTGAGCCATGAATCA

>BSNB_0669_ Enterobacter_amnigenus

GGCGGTCGACTTAACGCGTTAGCTCCGGAAGCCACGCCTCAAGGGCACAACCTCCAAGTCG
ACATCGTTTACGGCGTGGACTACCAGGGTATCTAATCCTGTTTGCTCCCCACGCTTTCGCACC
TGAGCGTCAGTCTTTGTCCAGGGGGCCGCCTTCGCCACCGGTATTCCTCCAGATCTCTACGCA
TTTCACCGCTACACCTGGAATTCTACCCCCCTCTACAAGACTCTAGCCTGCCAGTTTCGAATG
CAGTTCCCAGGTTGAGCCCGGGGATTTCACATCCGACTTGACAGACCGCCTGCGTGCGCTTT
ACGCCCAGTAATTCCGATTAACGCTTGCACCCTCCGTATTACCGCGGCTGCTGGCACGGAGT
TAGCCGGTGCTTCTTCTGCGGGTAACGTCAATTGCTGAGGTTATTAACCTCAACACCTTCCTC
CCCGCTGAAAGTACTTTACAACCCGAAGGCCTTCTTCATACACGCGGCATGGCTGCATCAGG
CTTGCGCCCATTGTGCAATATTCCCCACTGCTGCCTCCCGTAGGAGTCTGGACCGTGTCTCAG
TTCCAGTGTGGCTGGTCATCCTCTCAGACCAGCTAGGGATCGTCGCCTAGGTGAGCCGTTAC
CCCACCTACTAGCTAATCCCATCTGGGCACATCTGATGGCAAGAGGCCCGAAGGTCCCCCTC
TTTGGTCTTGCGACGTTATGCGGTATTAGCTACCGTTTCCAGTAGTTATCCCCCTCCATCAGG
CAGTTTCCCAGACATTACTCACCCGTCCGCCGCTCGCCGGCAAAGTAGCAAGCTACTTTCCG
CTGCCGCTCGACTTGCATGTGTTAGGCCTGCCGCCAGCG

>BSNB_0671_ Pantoea_stewartii

CTTAACGCGTTAGCTCCGGAAGCCACTCCTCAAGGGAACAACCTCCAAGTCGACATCGTTTA
CGGCGTGGACTACCAGGGTATCTAATCCTGTTTGCTCCCCACGCTTTCGCACCTGAGCGTCA
GTCTTCGTCCAGGGGGCCGCCTTCGCCACCGGTATTCCTCCAGATCTCTACGCATTTCACCGC
TACACCTGGAATTCTACCCCCCTCTACGAGACTCAAGCCTGCCAGTTTCAAATGCAGTTCCC
AGGTTAAGCCCGGGGATTTCACATCTGACTTAACAGACCGCCTGCGTGCGCTTTACGCCCAG
TAATTCCGATTAACGCTTGCACCCTCCGTATTACCGCGGCTGCTGGCACGGAGTTAGCCGGT
GCTTCTTCTGCGGGTAACGTCAATTGCTGAGGTTATTAACCTCAGCACCTTCCTCCCCGCTGA
AAGTACTTTACAACCCGAAGGCCTTCTTCATACACGCGGCATGGCTGCATCAGGCTTGCGCC
CATTGTGCAATATTCCCCACTGCTGCCTCCCGTAGGAGTCTGGACCGTGTCTCAGTTCCAGTG
TGGCTGGTCATCCTCTCAGACCAGCTAGGGATCGTCGCCTAGGTGGGCCGTTACCCCGCCTA
CCAGCTAATCCCATCTGGGCACATCCGATGGTGTGAGGCCCGAAGGTCCCCCACTTTGGTCT
TGCGACGTTATGCGGTATTAGCTACCGTTTCCAGT

>BSNB_0672_ Pantoea_stewartii

CTTAACGCGTTAGCTCCGGAAGCCACTCCTCAAGGGAACAACCTCCAAGTCGACATCGTTTA
CGGCGTGGACTACCAGGGTATCTAATCCTGTTTGCTCCCCACGCTTTCGCACCTGAGCGTCA
GTCTTCGTCCAGGGGGCCGCCTTCGCCACCGGTATTCCTCCAGATCTCTACGCATTTCACCGC
TACACCTGGAATTCTACCCCCCTCTACGAGACTCAAGCCTGCCAGTTTCAAATGCAGTTCCC
AGGTTAAGCCCGGGGATTTCACATCTGACTTAACAGACCGCCTGCGTGCGCTTTACGCCCAG
TAATTCCGATTAACGCTTGCACCCTCCGTATTACCGCGGCTGCTGGCACGGAGTTAGCCGGT
GCTTCTTCTGCGGGTAACGTCAATTGCTGAGGTTATTAACCTCAGCACCTTCCTCCCCGCTGA
AAGTACTTTACAACCCGAAGGCCTTCTTCATACACGCGGCATGGCTGCATCAGGCTTGCGCC
CATTGTGCAATATTCCCCACTGCTGCCTCCCGTAGGAGTCTGGACCGTGTCTCAGTTCCAGTG
TGGCTGGTCATCCTCTCAGACCAGCTAGGGATCGTCGCCTAGGTGGGCCGTTACCCCGCCTA
CCAGCTAATCCCATCTGGGCACATCCGATGGTGTGAGGCCCGAAGGTCCCCCACTTTGGTCT
TGCGACGTTATGCGGTATTAGCTACCGTTTCCAGT

>BSNB_0675_ Enterobacter_mori

GGCGGTCGACTTAACGCGTTAGCTCCGGAAGCCACGCCTCAAGGGCACAACCTCCAAGTCG
ACATCGTTTACGGCGTGGACTACCAGGGTATCTAATCCTGTTTGCTCCCCACGCTTTCGCACC
TGAGCGTCAGTCTTTGTCCAGGGGGCCGCCTTCGCCACCGGTATTCCTCCAGATCTCTACGCA
TTTCACCGCTACACCTGGAATTCTACCCCCCTCTACAAGACTCTAGCCTGCCAGTTTCGAATG
CAGTTCCCAGGTTGAGCCCGGGGATTTCACATCCGACTTGACAGACCGCCTGCGTGCGCTTT
ACGCCCAGTAATTCCGATTAACGCTTGCACCCTCCGTATTACCGCGGCTGCTGGCACGGAGT
TAGCCGGTGCTTCTTCTGCGGGTAACGTCAATTGCTGAGGTTATTAACCTCAACACCTTCCTC
CCCGCTGAAAGTACTTTACAACCCGAAGGCCTTCTTCATACACGCGGCATGGCTGCATCAGG
CTTGCGCCCATTGTGCAATATTCCCCACTGCTGCCTCCCGTAGGAGTCTGGACCGTGTCTCAG
TTCCAGTGTGGCTGGTCATCCTCTCAGACCAGCTAGGGATCGTCGCCTAGGTGAGCCGTTAC
CCCACCTACTAGCTAATCCCATCTGGGCACATCTGATGGCAAGAGGCCCGAAGGTCCCCCTC
TTTGGTCTTGCGACGTTATGCGGTATTAGCTACCGTTTCCAGTAGTTATCCCCCTCCATCAGG
CAGTTTCCCAGACATTACTCACCCGTCCGCCGCTCGCCGGCAAAGTAGCAAGCTACTTTCCG
CTGCCGCTCGACTTGCATGTGTTAGGCCTGCCGCCAGCG

>BSNB_0683_ Bacillus_subtilis

CGGAGTGCTTAATGCGTTAGCTGCAGCACTAAGGGGCGGAAACCCCCTAACACTTAGCACT
CATCGTTTACGGCGTGGACTACCAGGGTATCTAATCCTGTTCGCTCCCCACGCTTTCGCTCCT
CAGCGTCAGTTACAGACCAGAGAGTCGCCTTCGCCACTGGTGTTCCTCCACATCTCTACGCA
TTTCACCGCTACACGTGGAATTCCACTCTCCTCTTCTGCACTCAAGTTCCCCAGTTTCCAATG
ACCCTCCCCGGTTGAGCCGGGGGCTTTCACATCAGACTTAAGAAACCGCCTGCGAGCCCTTT
ACGCCCAATAATTCCGGACAACGCTTGCCACCTACGTATTACCGCGGCTGCTGGCACGTAGT
TAGCCGTGGCTTTCTGGTTAGGTACCGTCAAGGTACCGCCCTATTCGAACGGTACTTGTTCTT
CCCTAACAACAGAGCTTTACGATCCGAAAACCTTCATCACTCACGCGGCGTTGCTCCGTCAG
ACTTTCGTCCATTGCGGAAGATTCCCTACTGCTGCCTCCCGTAGGAGTCTGGGCCGTGTCTCA
GTCCCAGTGTGGCCGATCACCCTCTCAGGTCGGCTACGCATCGTTGCCTTGGTGAGCCGTTAC
CTCACCAACTAGCTAATGCGCCGCGGGTCCATCTGTAAGTGGTAGCCGAAGCCACCTTTTAT
GTTTGAACCATGCGGTTCAAACAACCATCCGGTATTAGCCCCGGTTTCCCGGAGTTATCCCA
GTCTTACAGGCAGGTTACCCACGTGTTACTCACCCGTCCGCCGCTAACATCAGGGAGCAAGC
TCCCATCTGTCCGCTCGACTTGCATGTATTAGGCACGCCGCCAGCGTTCGTC

>BSNB_0685_ Bacillus_subtilis

CGGAGTGCTTAATGCGTTAGCTGCAGCACTAAGGGGCGGAAACCCCCTAACACTTAGCACT
CATCGTTTACGGCGTGGACTACCAGGGTATCTAATCCTGTTCGCTCCCCACGCTTTCGCTCCT
CAGCGTCAGTTACAGACCAGAGAGTCGCCTTCGCCACTGGTGTTCCTCCACATCTCTACGCA
TTTCACCGCTACACGTGGAATTCCACTCTCCTCTTCTGCACTCAAGTTCCCCAGTTTCCAATG
ACCCTCCCCGGTTGAGCCGGGGGCTTTCACATCAGACTTAAGAAACCGCCTGCGAGCCCTTT
ACGCCCAATAATTCCGGACAACGCTTGCCACCTACGTATTACCGCGGCTGCTGGCACGTAGT
TAGCCGTGGCTTTCTGGTTAGGTACCGTCAAGGTACCGCCCTATTCGAACGGTACTTGTTCTT
CCCTAACAACAGAGCTTTACGATCCGAAAACCTTCATCACTCACGCGGCGTTGCTCCGTCAG
ACTTTCGTCCATTGCGGAAGATTCCCTACTGCTGCCTCCCGTAGGAGTCTGGGCCGTGTCTCA
GTCCCAGTGTGGCCGATCACCCTCTCAGGTCGGCTACGCATCGTTGCCTTGGTGAGCCGTTAC
CTCACCAACTAGCTAATGCGCCGCGGGTCCATCTGTAAGTGGTAGCCGAAGCCACCTTTTAT
GTTTGAACCATGCGGTTCAAACAACCATCCGGTATTAGCCCCGGTTTCCCGGAGTTATCCCA
GTCTTACAGGCAGGTTACCCACGTGTTACTCACCCGTCCGCCGCTAACATCAGGGAGCAAGC
TCCCATCTGTCCGCTCGACTTGCATGTATTAGGCACGCCGCCAGCGTTCGTC

>BSNB_0704_ Stenotrophomonas_maltophilia

CAGGCGGCGAACTTAACGCGTTAGCTTCGATACTGCGTGCCAAATTGCACCCAACATCCATG

TTCGCATCGTTTAGGGCGTGGACTACCAGGGTATCTAATCCTGTTTGCTCCCCACGCTTTCGT
GCCTCAGTGTCAATGTTGGTCCAGGTAGCTGCCTTCGCCATGGATGTTCCTCCTGATCTCTAC
GCATTTCACTGCTACACCAGGAATTCCGCTACCCTCTACCACATTCTAGTCATCCAGTATCCA
CTGCAGTTCCCAGGTTGAGCCCAGGGCTTTCACAACGGACTTAAATAACCACCTACGCACGC
TTTACGCCCAGTAATTCCGAGTAACGCTTGCACCCTTCGTATTACCGCGGCTGCTGGCACGA
AGTTAGCCGGTGCTTATTCTTTGGGTACCGTCATCCCAACCAGGTATTAACCGGCTGGATTTC
TTTCCCAACAAAAGGGCTTTACAACCCGAAGGCCTTCTTCACCCACGCGGTATGGCTGGATC
AGGCTTGCGCCCATTGTCCAATATTCCCCACTGCTGCCTCCCGTAGGAGTCTGGACCGTGTCT
CAGTTCCAGTGTGGCTGATCATCCTCTCAAACCAGCTACGGATCGTCGCCTTGGTGGGCCTTT
ACCCCGCCAACTAGCTAATCCGACATCGGCTCATTCAATCGCGCAAGGTCCGAAGATCCCCT
GCTTTCACCCGTAGGTCGTATGCG

>BSNB_0706_ Enterobacter_mori

GGCGGTCGACTTAACGCGTTAGCTCCGGAAGCCACGCCTCAAGGGCACAACCTCCAAGTCG
ACATCGTTTACGGCGTGGACTACCAGGGTATCTAATCCTGTTTGCTCCCCACGCTTTCGCACC
TGAGCGTCAGTCTTTGTCCAGGGGGCCGCCTTCGCCACCGGTATTCCTCCAGATCTCTACGCA
TTTCACCGCTACACCTGGAATTCTACCCCCCTCTACAAGACTCTAGCCTGCCAGTTTCGAATG
CAGTTCCCAGGTTGAGCCCGGGGATTTCACATCCGACTTGACAGACCGCCTGCGTGCGCTTT
ACGCCCAGTAATTCCGATTAACGCTTGCACCCTCCGTATTACCGCGGCTGCTGGCACGGAGT
TAGCCGGTGCTTCTTCTGCGGGTAACGTCAATTGCTGAGGTTATTAACCTCAACACCTTCCTC
CCCGCTGAAAGTACTTTACAACCCGAAGGCCTTCTTCATACACGCGGCATGGCTGCATCAGG
CTTGCGCCCATTGTGCAATATTCCCCACTGCTGCCTCCCGTAGGAGTCTGGACCGTGTCTCAG
TTCCAGTGTGGCTGGTCATCCTCTCAGACCAGCTAGGGATCGTCGCCTAGGTGAGCCGTTAC
CCCACCTACTAGCTAATCCCATCTGGGCACATCTGATGGCAAGAGGCCCGAAGGTCCCCCTC
TTTGGTCTTGCGACGTTATGCGGTATTAGCTACCGTTTCCAGTAGTTATCCCCCTCCATCAGG
CAGTTTCCCAGACATTACTCACCCGTCCGCCGCTCGCCGGCAAAGTAGCAAGCTACTTTCCG
CTGCCGCTCGACTTGCATGTGTTAGGCCTGCCGCCAGCG

>BSNB_0708_ Enterobacter_mori

GGCGGTCGACTTAACGCGTTAGCTCCGGAAGCCACGCCTCAAGGGCACAACCTCCAAGTCG
ACATCGTTTACGGCGTGGACTACCAGGGTATCTAATCCTGTTTGCTCCCCACGCTTTCGCACC
TGAGCGTCAGTCTTTGTCCAGGGGGCCGCCTTCGCCACCGGTATTCCTCCAGATCTCTACGCA
TTTCACCGCTACACCTGGAATTCTACCCCCCTCTACAAGACTCTAGCCTGCCAGTTTCGAATG
CAGTTCCCAGGTTGAGCCCGGGGATTTCACATCCGACTTGACAGACCGCCTGCGTGCGCTTT
ACGCCCAGTAATTCCGATTAACGCTTGCACCCTCCGTATTACCGCGGCTGCTGGCACGGAGT
TAGCCGGTGCTTCTTCTGCGGGTAACGTCAATTGCTGAGGTTATTAACCTCAACACCTTCCTC
CCCGCTGAAAGTACTTTACAACCCGAAGGCCTTCTTCATACACGCGGCATGGCTGCATCAGG
CTTGCGCCCATTGTGCAATATTCCCCACTGCTGCCTCCCGTAGGAGTCTGGACCGTGTCTCAG
TTCCAGTGTGGCTGGTCATCCTCTCAGACCAGCTAGGGATCGTCGCCTAGGTGAGCCGTTAC
CCCACCTACTAGCTAATCCCATCTGGGCACATCTGATGGCAAGAGGCCCGAAGGTCCCCCTC
TTTGGTCTTGCGACGTTATGCGGTATTAGCTACCGTTTCCAGTAGTTATCCCCCTCCATCAGG
CAGTTTCCCAGACATTACTCACCCGTCCGCCGCTCGCCGGCAAAGTAGCAAGCTACTTTCCG
CTGCCGCTCGACTTGCATGTGTTAGGCCTGCCGCCAGCG

>BSNB_0709_ Enterobacter_mori

GGCGGTCGACTTAACGCGTTAGCTCCGGAAGCCACGCCTCAAGGGCACAACCTCCAAGTCG
ACATCGTTTACGGCGTGGACTACCAGGGTATCTAATCCTGTTTGCTCCCCACGCTTTCGCACC
TGAGCGTCAGTCTTTGTCCAGGGGGCCGCCTTCGCCACCGGTATTCCTCCAGATCTCTACGCA
TTTCACCGCTACACCTGGAATTCTACCCCCCTCTACAAGACTCTAGCCTGCCAGTTTCGAATG

CAGTTCCCAGGTTGAGCCCGGGGATTTCACATCCGACTTGACAGACCGCCTGCGTGCGCTTT
ACGCCCAGTAATTCCGATTAACGCTTGCACCCTCCGTATTACCGCGGCTGCTGGCACGGAGT
TAGCCGGTGCTTCTTCTGCGGGTAACGTCAATTGCTGAGGTTATTAACCTCAACACCTTCCTC
CCCGCTGAAAGTACTTTACAACCCGAAGGCCTTCTTCATACACGCGGCATGGCTGCATCAGG
CTTGCGCCCATTGTGCAATATTCCCCACTGCTGCCTCCCGTAGGAGTCTGGACCGTGTCTCAG
TTCCAGTGTGGCTGGTCATCCTCTCAGACCAGCTAGGGATCGTCGCCTAGGTGAGCCGTTAC
CCCACCTACTAGCTAATCCCATCTGGGCACATCTGATGGCAAGAGGCCCGAAGGTCCCCCTC
TTTGGTCTTGCGACGTTATGCGGTATTAGCTACCGTTTCCAGTAGTTATCCCCCTCCATCAGG
CAGTTTCCCAGACATTACTCACCCGTCCGCCGCTCGCCGGCAAAGTAGCAAGCTACTTTCCG
CTGCCGCTCGACTTGCATGTGTTAGGCCTGCCGCCAGCG

>BSNB_0712_ Bacillus_subtilis

GGCTCAGGACGAACGCTGGCGGCGTGCCTAATACATGCAAGTCGAGCGGACAGATGGGAG
CTTGCTCCCTGATGTTAGCGGCGGACGGGTGAGTAACACGTGGGTAACCTGCCTGTAAGACT
GGGATAACTCCGGGAAACCGGGGCTAATACCGGATGGTTGTTTGAACCGCATGGTTCAAAC
ATAAAAGGTGGCTTCGGCTACCACTTACAGATGGACCCGCGGCGCATTAGCTAGTTGGTGA
GGTAACGGCTCACCAAGGCAACGATGCGTAGCCGACCTGAGAGGGTGATCGGCCACACTGG
GACTGAGACACGGCCCAGACTCCTACGGGAGGCAGCAGTAGGGAATCTTCCGCAATGGAC
GAAAGTCTGACGGAGCAACGCCGCGTGAGTGATGAAGGTTTTCGGATCGTAAAGCTCTGTT
GTTAGGGAAGAACAAGTACCGTTCGAATAGGGCGGTACCTTGACGGTACCTAACCAGAAAG
CCACGGCTAACTACGTGCCAGCAGCCGCGGTAATACGTAGGTGGCAAGCGTTGTCCGGAAT
TATTGGGCGTAAAGGGCTCGCAGGCGGTTTCTTAAGTCTGATGTGAAAGCCCCCGGCTCAAC
CGGGGAGGGTCATTGGAAACTGGGGAACTTGAGTGCAGAAGAGGAGAGTGGAATTCCACG
TGTAGCGGTGAAATGCGTAGAGATGTGGAGGAACACCAGTGGCGAAGGCGACTCTCTGGTC
TGTAACTGACGCTGAGGAGCGAAAGCGTGGGGAGCGAACAGGATTAGATACCCTGGTAGTC
CACGCCGTAAACGATGAGTGCTAAGTGTTAGGGGGTTTCCGCCCCTTAGTGCTGCAGCTAAC
GCATTAAGCACTCCGCC
>BSNB_0716_ Bacillus_subtilis

CGGAGTGCTTAATGCGTTAGCTGCAGCACTAAGGGGCGGAAACCCCCTAACACTTAGCACT
CATCGTTTACGGCGTGGACTACCAGGGTATCTAATCCTGTTCGCTCCCCACGCTTTCGCTCCT
CAGCGTCAGTTACAGACCAGAGAGTCGCCTTCGCCACTGGTGTTCCTCCACATCTCTACGCA
TTTCACCGCTACACGTGGAATTCCACTCTCCTCTTCTGCACTCAAGTTCCCCAGTTTCCAATG
ACCCTCCCCGGTTGAGCCGGGGGCTTTCACATCAGACTTAAGAAACCGCCTGCGAGCCCTTT
ACGCCCAATAATTCCGGACAACGCTTGCCACCTACGTATTACCGCGGCTGCTGGCACGTAGT
TAGCCGTGGCTTTCTGGTTAGGTACCGTCAAGGTACCGCCCTATTCGAACGGTACTTGTTCTT
CCCTAACAACAGAGCTTTACGATCCGAAAACCTTCATCACTCACGCGGCGTTGCTCCGTCAG
ACTTTCGTCCATTGCGGAAGATTCCCTACTGCTGCCTCCCGTAGGAGTCTGGGCCGTGTCTCA
GTCCCAGTGTGGCCGATCACCCTCTCAGGTCGGCTACGCATCGTTGCCTTGGTGAGCCGTTAC
CTCACCAACTAGCTAATGCGCCGCGGGTCCATCTGTAAGTGGTAGCCGAAGCCACCTTTTAT
GTTTGAACCATGCGGTTCAAACAACCATCCGGTATTAGCCCCGGTTTCCCGGAGTTATCCCA
GTCTTACAGGCAGGTTACCCACGTGTTACTCACCCGTCCGCCGCTAACATCAGGGAGCAAGC
TCCCATCTGTCCGCTCGACTTGCATGTATTAGGCACGCCGCCAGCGTTCGTC

>BSNB_0719_ Luteibacter_yeojuensis

GGCGAACTTAACGCGTTAGCTTCGACACTGATCTCCGAGTTGAGACCAACATCCAGTTCGCA
TCGTTTAGGGCGTGGACTACCAGGGTATCTAATCCTGTTTGCTCCCCACGCTTTCGTGCCTCA
GCGTCAGTGTTGATCCAGATGGCCGCCTTCGCCACTGATGTTCCTCCCGATCTCTACGCATTT
CACCGCTACACCGGGAATTCCACCATCCTCTATCACACTCTAGCTCGCCAGTATCCACTGCC
ATTCCCAGGTTGAGCCCGGGGCTTTCACAGCAGACTTAACGAACCGCCTACGCACGCTTTAC

GCCCAGTAATTCCGATTAACGCTTGCACCCTCCGTATTACCGCGGCTGCTGGCACGGAGTTA
GCCGGTGCTTATTCCTCAGGTACCGTCAGACTGCACGGGTATTAACCTTGCAGATTTCGTTCC
TGATAAAAGTGCTTTACAACCCGAGGGCCTTCTTCACACACGCGGTATTGCTGGATCAGGCT
TGCGCCCATTGTCCAATATTCCCCACTGCTGCCTCCCGTAGGAGTCTGGGCCGTGTCTCAGTC
CCAGTGTGGCTGATCATCCTCTCAGACCAGCTAGCGATCGTCGCCTTGGTAAGCCATTACCTT
ACCAACTAGCTAATCGCACATCGGTCCATCCAACCGCGCGAGGTCTTGCGATCCCCCGCTTT
CTCCCGTAGGACGTATGCGGTATTAGCGTAAGTTTCCCTACGTTATCCCCCACGTCTGGGTAG
GTCCCGATGCATTACTCACCCGTCCGCCACTCGCCACCCATAGAGCAAGCTCTACCGTGCTG
CCGTTCGACTTGCATGTGTTAAGCATACCGCCAGCGTTCAATC

>BSNB_0721_ Luteibacter_yeojuensis

GGCGAACTTAACGCGTTAGCTTCGACACTGATCTCCGAGTTGAGACCAACATCCAGTTCGCA
TCGTTTAGGGCGTGGACTACCAGGGTATCTAATCCTGTTTGCTCCCCACGCTTTCGTGCCTCA
GCGTCAGTGTTGATCCAGATGGCCGCCTTCGCCACTGATGTTCCTCCCGATCTCTACGCATTT
CACCGCTACACCGGGAATTCCACCATCCTCTATCACACTCTAGCTCGCCAGTATCCACTGCC
ATTCCCAGGTTGAGCCCGGGGCTTTCACAGCAGACTTAACGAACCGCCTACGCACGCTTTAC
GCCCAGTAATTCCGATTAACGCTTGCACCCTCCGTATTACCGCGGCTGCTGGCACGGAGTTA
GCCGGTGCTTATTCCTCAGGTACCGTCAGACTGCACGGGTATTAACCTTGCAGATTTCGTTCC
TGATAAAAGTGCTTTACAACCCGAGGGCCTTCTTCACACACGCGGTATTGCTGGATCAGGCT
TGCGCCCATTGTCCAATATTCCCCACTGCTGCCTCCCGTAGGAGTCTGGGCCGTGTCTCAGTC
CCAGTGTGGCTGATCATCCTCTCAGACCAGCTAGCGATCGTCGCCTTGGTAAGCCATTACCTT
ACCAACTAGCTAATCGCACATCGGTCCATCCAACCGCGCGAGGTCTTGCGATCCCCCGCTTT
CTCCCGTAGGACGTATGCGGTATTAGCGTAAGTTTCCCTACGTTATCCCCCACGTCTGGGTAG
GTCCCGATGCATTACTCACCCGTCCGCCACTCGCCACCCATAGAGCAAGCTCTACCGTGCTG
CCGTTCGACTTGCATGTGTTAAGCATACCGCCAGCGTTCAATC

>BSNB_0722_ Bacillus_subtilis

GGCTCAGGACGAACGCTGGCGGCGTGCCTAATACATGCAAGTCGAGCGGACAGATGGGAG
CTTGCTCCCTGATGTTAGCGGCGGACGGGTGAGTAACACGTGGGTAACCTGCCTGTAAGACT
GGGATAACTCCGGGAAACCGGGGCTAATACCGGATGGTTGTTTGAACCGCATGGTTCAAAC
ATAAAAGGTGGCTTCGGCTACCACTTACAGATGGACCCGCGGCGCATTAGCTAGTTGGTGA
GGTAACGGCTCACCAAGGCAACGATGCGTAGCCGACCTGAGAGGGTGATCGGCCACACTGG
GACTGAGACACGGCCCAGACTCCTACGGGAGGCAGCAGTAGGGAATCTTCCGCAATGGAC
GAAAGTCTGACGGAGCAACGCCGCGTGAGTGATGAAGGTTTTCGGATCGTAAAGCTCTGTT
GTTAGGGAAGAACAAGTACCGTTCGAATAGGGCGGTACCTTGACGGTACCTAACCAGAAAG
CCACGGCTAACTACGTGCCAGCAGCCGCGGTAATACGTAGGTGGCAAGCGTTGTCCGGAAT
TATTGGGCGTAAAGGGCTCGCAGGCGGTTTCTTAAGTCTGATGTGAAAGCCCCCGGCTCAAC
CGGGGAGGGTCATTGGAAACTGGGGAACTTGAGTGCAGAAGAGGAGAGTGGAATTCCACG
TGTAGCGGTGAAATGCGTAGAGATGTGGAGGAACACCAGTGGCGAAGGCGACTCTCTGGTC
TGTAACTGACGCTGAGGAGCGAAAGCGTGGGGAGCGAACAGGATTAGATACCCTGGTAGTC
CACGCCGTAAACGATGAGTGCTAAGTGTTAGGGGGTTTCCGCCCCTTAGTGCTGCAGCTAAC
GCATTAAGCACTCCGCC
>BSNB_0723_ Enterobacter_mori

GGCGGTCGACTTAACGCGTTAGCTCCGGAAGCCACGCCTCAAGGGCACAACCTCCAAGTCG
ACATCGTTTACGGCGTGGACTACCAGGGTATCTAATCCTGTTTGCTCCCCACGCTTTCGCACC
TGAGCGTCAGTCTTTGTCCAGGGGGCCGCCTTCGCCACCGGTATTCCTCCAGATCTCTACGCA
TTTCACCGCTACACCTGGAATTCTACCCCCCTCTACAAGACTCTAGCCTGCCAGTTTCGAATG
CAGTTCCCAGGTTGAGCCCGGGGATTTCACATCCGACTTGACAGACCGCCTGCGTGCGCTTT
ACGCCCAGTAATTCCGATTAACGCTTGCACCCTCCGTATTACCGCGGCTGCTGGCACGGAGT

TAGCCGGTGCTTCTTCTGCGGGTAACGTCAATTGCTGAGGTTATTAACCTCAACACCTTCCTC
CCCGCTGAAAGTACTTTACAACCCGAAGGCCTTCTTCATACACGCGGCATGGCTGCATCAGG
CTTGCGCCCATTGTGCAATATTCCCCACTGCTGCCTCCCGTAGGAGTCTGGACCGTGTCTCAG
TTCCAGTGTGGCTGGTCATCCTCTCAGACCAGCTAGGGATCGTCGCCTAGGTGAGCCGTTAC
CCCACCTACTAGCTAATCCCATCTGGGCACATCTGATGGCAAGAGGCCCGAAGGTCCCCCTC
TTTGGTCTTGCGACGTTATGCGGTATTAGCTACCGTTTCCAGTAGTTATCCCCCTCCATCAGG
CAGTTTCCCAGACATTACTCACCCGTCCGCCGCTCGCCGGCAAAGTAGCAAGCTACTTTCCG
CTGCCGCTCGACTTGCATGTGTTAGGCCTGCCGCCAGCG

>BSNB_0725_ Enterobacter_mori

GGCGGTCGACTTAACGCGTTAGCTCCGGAAGCCACGCCTCAAGGGCACAACCTCCAAGTCG
ACATCGTTTACGGCGTGGACTACCAGGGTATCTAATCCTGTTTGCTCCCCACGCTTTCGCACC
TGAGCGTCAGTCTTTGTCCAGGGGGCCGCCTTCGCCACCGGTATTCCTCCAGATCTCTACGCA
TTTCACCGCTACACCTGGAATTCTACCCCCCTCTACAAGACTCTAGCCTGCCAGTTTCGAATG
CAGTTCCCAGGTTGAGCCCGGGGATTTCACATCCGACTTGACAGACCGCCTGCGTGCGCTTT
ACGCCCAGTAATTCCGATTAACGCTTGCACCCTCCGTATTACCGCGGCTGCTGGCACGGAGT
TAGCCGGTGCTTCTTCTGCGGGTAACGTCAATTGCTGAGGTTATTAACCTCAACACCTTCCTC
CCCGCTGAAAGTACTTTACAACCCGAAGGCCTTCTTCATACACGCGGCATGGCTGCATCAGG
CTTGCGCCCATTGTGCAATATTCCCCACTGCTGCCTCCCGTAGGAGTCTGGACCGTGTCTCAG
TTCCAGTGTGGCTGGTCATCCTCTCAGACCAGCTAGGGATCGTCGCCTAGGTGAGCCGTTAC
CCCACCTACTAGCTAATCCCATCTGGGCACATCTGATGGCAAGAGGCCCGAAGGTCCCCCTC
TTTGGTCTTGCGACGTTATGCGGTATTAGCTACCGTTTCCAGTAGTTATCCCCCTCCATCAGG
CAGTTTCCCAGACATTACTCACCCGTCCGCCGCTCGCCGGCAAAGTAGCAAGCTACTTTCCG
CTGCCGCTCGACTTGCATGTGTTAGGCCTGCCGCCAGCG

>BSNB_0726_ Enterobacter_mori

GGCGGTCGACTTAACGCGTTAGCTCCGGAAGCCACGCCTCAAGGGCACAACCTCCAAGTCG
ACATCGTTTACGGCGTGGACTACCAGGGTATCTAATCCTGTTTGCTCCCCACGCTTTCGCACC
TGAGCGTCAGTCTTTGTCCAGGGGGCCGCCTTCGCCACCGGTATTCCTCCAGATCTCTACGCA
TTTCACCGCTACACCTGGAATTCTACCCCCCTCTACAAGACTCTAGCCTGCCAGTTTCGAATG
CAGTTCCCAGGTTGAGCCCGGGGATTTCACATCCGACTTGACAGACCGCCTGCGTGCGCTTT
ACGCCCAGTAATTCCGATTAACGCTTGCACCCTCCGTATTACCGCGGCTGCTGGCACGGAGT
TAGCCGGTGCTTCTTCTGCGGGTAACGTCAATTGCTGAGGTTATTAACCTCAACACCTTCCTC
CCCGCTGAAAGTACTTTACAACCCGAAGGCCTTCTTCATACACGCGGCATGGCTGCATCAGG
CTTGCGCCCATTGTGCAATATTCCCCACTGCTGCCTCCCGTAGGAGTCTGGACCGTGTCTCAG
TTCCAGTGTGGCTGGTCATCCTCTCAGACCAGCTAGGGATCGTCGCCTAGGTGAGCCGTTAC
CCCACCTACTAGCTAATCCCATCTGGGCACATCTGATGGCAAGAGGCCCGAAGGTCCCCCTC
TTTGGTCTTGCGACGTTATGCGGTATTAGCTACCGTTTCCAGTAGTTATCCCCCTCCATCAGG
CAGTTTCCCAGACATTACTCACCCGTCCGCCGCTCGCCGGCAAAGTAGCAAGCTACTTTCCG
CTGCCGCTCGACTTGCATGTGTTAGGCCTGCCGCCAGCG

>BSNB_0727_ Enterobacter_mori

GGCGGTCGACTTAACGCGTTAGCTCCGGAAGCCACGCCTCAAGGGCACAACCTCCAAGTCG
ACATCGTTTACGGCGTGGACTACCAGGGTATCTAATCCTGTTTGCTCCCCACGCTTTCGCACC
TGAGCGTCAGTCTTTGTCCAGGGGGCCGCCTTCGCCACCGGTATTCCTCCAGATCTCTACGCA
TTTCACCGCTACACCTGGAATTCTACCCCCCTCTACAAGACTCTAGCCTGCCAGTTTCGAATG
CAGTTCCCAGGTTGAGCCCGGGGATTTCACATCCGACTTGACAGACCGCCTGCGTGCGCTTT
ACGCCCAGTAATTCCGATTAACGCTTGCACCCTCCGTATTACCGCGGCTGCTGGCACGGAGT
TAGCCGGTGCTTCTTCTGCGGGTAACGTCAATTGCTGAGGTTATTAACCTCAACACCTTCCTC

CCCGCTGAAAGTACTTTACAACCCGAAGGCCTTCTTCATACACGCGGCATGGCTGCATCAGG
CTTGCGCCCATTGTGCAATATTCCCCACTGCTGCCTCCCGTAGGAGTCTGGACCGTGTCTCAG
TTCCAGTGTGGCTGGTCATCCTCTCAGACCAGCTAGGGATCGTCGCCTAGGTGAGCCGTTAC
CCCACCTACTAGCTAATCCCATCTGGGCACATCTGATGGCAAGAGGCCCGAAGGTCCCCCTC
TTTGGTCTTGCGACGTTATGCGGTATTAGCTACCGTTTCCAGTAGTTATCCCCCTCCATCAGG
CAGTTTCCCAGACATTACTCACCCGTCCGCCGCTCGCCGGCAAAGTAGCAAGCTACTTTCCG
CTGCCGCTCGACTTGCATGTGTTAGGCCTGCCGCCAGCG

>BSNB_0730_ Bacillus_subtilis

CGGAGTGCTTAATGCGTTAGCTGCAGCACTAAGGGGCGGAAACCCCCTAACACTTAGCACT
CATCGTTTACGGCGTGGACTACCAGGGTATCTAATCCTGTTCGCTCCCCACGCTTTCGCTCCT
CAGCGTCAGTTACAGACCAGAGAGTCGCCTTCGCCACTGGTGTTCCTCCACATCTCTACGCA
TTTCACCGCTACACGTGGAATTCCACTCTCCTCTTCTGCACTCAAGTTCCCCAGTTTCCAATG
ACCCTCCCCGGTTGAGCCGGGGGCTTTCACATCAGACTTAAGAAACCGCCTGCGAGCCCTTT
ACGCCCAATAATTCCGGACAACGCTTGCCACCTACGTATTACCGCGGCTGCTGGCACGTAGT
TAGCCGTGGCTTTCTGGTTAGGTACCGTCAAGGTACCGCCCTATTCGAACGGTACTTGTTCTT
CCCTAACAACAGAGCTTTACGATCCGAAAACCTTCATCACTCACGCGGCGTTGCTCCGTCAG
ACTTTCGTCCATTGCGGAAGATTCCCTACTGCTGCCTCCCGTAGGAGTCTGGGCCGTGTCTCA
GTCCCAGTGTGGCCGATCACCCTCTCAGGTCGGCTACGCATCGTTGCCTTGGTGAGCCGTTAC
CTCACCAACTAGCTAATGCGCCGCGGGTCCATCTGTAAGTGGTAGCCGAAGCCACCTTTTAT
GTTTGAACCATGCGGTTCAAACAACCATCCGGTATTAGCCCCGGTTTCCCGGAGTTATCCCA
GTCTTACAGGCAGGTTACCCACGTGTTACTCACCCGTCCGCCGCTAACATCAGGGAGCAAGC
TCCCATCTGTCCGCTCGACTTGCATGTATTAGGCACGCCGCCAGCGTTCGTC

>BSNB_0762_ Bacillus_subtilis

GGCTCAGGACGAACGCTGGCGGCGTGCCTAATACATGCAAGTCGAGCGGACAGATGGGAG
CTTGCTCCCTGATGTTAGCGGCGGACGGGTGAGTAACACGTGGGTAACCTGCCTGTAAGACT
GGGATAACTCCGGGAAACCGGGGCTAATACCGGATGGTTGTTTGAACCGCATGGTTCAAAC
ATAAAAGGTGGCTTCGGCTACCACTTACAGATGGACCCGCGGCGCATTAGCTAGTTGGTGA
GGTAACGGCTCACCAAGGCAACGATGCGTAGCCGACCTGAGAGGGTGATCGGCCACACTGG
GACTGAGACACGGCCCAGACTCCTACGGGAGGCAGCAGTAGGGAATCTTCCGCAATGGAC
GAAAGTCTGACGGAGCAACGCCGCGTGAGTGATGAAGGTTTTCGGATCGTAAAGCTCTGTT
GTTAGGGAAGAACAAGTACCGTTCGAATAGGGCGGTACCTTGACGGTACCTAACCAGAAAG
CCACGGCTAACTACGTGCCAGCAGCCGCGGTAATACGTAGGTGGCAAGCGTTGTCCGGAAT
TATTGGGCGTAAAGGGCTCGCAGGCGGTTTCTTAAGTCTGATGTGAAAGCCCCCGGCTCAAC
CGGGGAGGGTCATTGGAAACTGGGGAACTTGAGTGCAGAAGAGGAGAGTGGAATTCCACG
TGTAGCGGTGAAATGCGTAGAGATGTGGAGGAACACCAGTGGCGAAGGCGACTCTCTGGTC
TGTAACTGACGCTGAGGAGCGAAAGCGTGGGGAGCGAACAGGATTAGATACCCTGGTAGTC
CACGCCGTAAACGATGAGTGCTAAGTGTTAGGGGGTTTCCGCCCCTTAGTGCTGCAGCTAAC
GCATTAAGCACTCCGCC
>BSNB_0803_ Bacillus_subtilis

GGCTCAGGACGAACGCTGGCGGCGTGCCTAATACATGCAAGTCGAGCGGACAGATGGGAG
CTTGCTCCCTGATGTTAGCGGCGGACGGGTGAGTAACACGTGGGTAACCTGCCTGTAAGACT
GGGATAACTCCGGGAAACCGGGGCTAATACCGGATGGTTGTTTGAACCGCATGGTTCAAAC
ATAAAAGGTGGCTTCGGCTACCACTTACAGATGGACCCGCGGCGCATTAGCTAGTTGGTGA
GGTAACGGCTCACCAAGGCAACGATGCGTAGCCGACCTGAGAGGGTGATCGGCCACACTGG
GACTGAGACACGGCCCAGACTCCTACGGGAGGCAGCAGTAGGGAATCTTCCGCAATGGAC
GAAAGTCTGACGGAGCAACGCCGCGTGAGTGATGAAGGTTTTCGGATCGTAAAGCTCTGTT
GTTAGGGAAGAACAAGTACCGTTCGAATAGGGCGGTACCTTGACGGTACCTAACCAGAAAG

CCACGGCTAACTACGTGCCAGCAGCCGCGGTAATACGTAGGTGGCAAGCGTTGTCCGGAAT
TATTGGGCGTAAAGGGCTCGCAGGCGGTTTCTTAAGTCTGATGTGAAAGCCCCCGGCTCAAC
CGGGGAGGGTCATTGGAAACTGGGGAACTTGAGTGCAGAAGAGGAGAGTGGAATTCCACG
TGTAGCGGTGAAATGCGTAGAGATGTGGAGGAACACCAGTGGCGAAGGCGACTCTCTGGTC
TGTAACTGACGCTGAGGAGCGAAAGCGTGGGGAGCGAACAGGATTAGATACCCTGGTAGTC
CACGCCGTAAACGATGAGTGCTAAGTGTTAGGGGGTTTCCGCCCCTTAGTGCTGCAGCTAAC
GCATTAAGCACTCCGCC
>BSNB_0804_ Bacillus_subtilis

GGCTCAGGACGAACGCTGGCGGCGTGCCTAATACATGCAAGTCGAGCGGACAGATGGGAG
CTTGCTCCCTGATGTTAGCGGCGGACGGGTGAGTAACACGTGGGTAACCTGCCTGTAAGACT
GGGATAACTCCGGGAAACCGGGGCTAATACCGGATGGTTGTTTGAACCGCATGGTTCAAAC
ATAAAAGGTGGCTTCGGCTACCACTTACAGATGGACCCGCGGCGCATTAGCTAGTTGGTGA
GGTAACGGCTCACCAAGGCAACGATGCGTAGCCGACCTGAGAGGGTGATCGGCCACACTGG
GACTGAGACACGGCCCAGACTCCTACGGGAGGCAGCAGTAGGGAATCTTCCGCAATGGAC
GAAAGTCTGACGGAGCAACGCCGCGTGAGTGATGAAGGTTTTCGGATCGTAAAGCTCTGTT
GTTAGGGAAGAACAAGTACCGTTCGAATAGGGCGGTACCTTGACGGTACCTAACCAGAAAG
CCACGGCTAACTACGTGCCAGCAGCCGCGGTAATACGTAGGTGGCAAGCGTTGTCCGGAAT
TATTGGGCGTAAAGGGCTCGCAGGCGGTTTCTTAAGTCTGATGTGAAAGCCCCCGGCTCAAC
CGGGGAGGGTCATTGGAAACTGGGGAACTTGAGTGCAGAAGAGGAGAGTGGAATTCCACG
TGTAGCGGTGAAATGCGTAGAGATGTGGAGGAACACCAGTGGCGAAGGCGACTCTCTGGTC
TGTAACTGACGCTGAGGAGCGAAAGCGTGGGGAGCGAACAGGATTAGATACCCTGGTAGTC
CACGCCGTAAACGATGAGTGCTAAGTGTTAGGGGGTTTCCGCCCCTTAGTGCTGCAGCTAAC
GCATTAAGCACTCCGCC
>BSNB_0805_ Methylobacterium_mesophilicum

CCGGATACGCCCTTTTGGGGAAAGGTTTACTGCCGGAAGATCGGCCCGCGTCTGATTAGCTA
GTTGGTGGGGTAACGGCCTACCAAGGCGACGATCAGTAGCTGGTCTGAGAGGATGATCAGC
CACACTGGGACTGAGACACGGCCCAGACTCCTACGGGAGGCAGCAGTGGGGAATATTGGA
CAATGGGCGCAAGCCTGATCCAGCCATGCCGCGTGAGTGATGAAGGCCTTAGGGTTGTAAA
GCTCTTTTATCCGGGACGATAATGACGGTACCGGAGGAATAAGCCCCGGCTAACTTCGTGCC
AGCAGCCGCGGTAATACGAAGGGGGCTAGCGTTGCTCGGAATCACTGGGCGTAAAGGGCGC
GTAGGCGGCGTTTTAAGTCGGGGGTGAAAGCCTGTGGCTCAACCACAGAATGGCCTTCGAT
ACTGGGACGCTTGAGTATGGTAGAGGTTGGTGGAACTGCGAGTGTAGAGGTGAAATTCGTA
GATATTCGCAAGAACACCGGTGGCGAAGGCGGCCAACTGGACCATTACTGACGCTGAGGCG
CGAAAGCGTGGGGAGCAAACAGGATTAGATACCCTGGTAGTCCACGCCGTAAACGATGAAT
GCCAGCTGTTGGGGTGCTTGCACCGCAGTAGCGCAGCTAACGCTTTGAGCATCCGCC
>BSNB_0816_ Bacillus_subtilis

GGCTCAGGACGAACGCTGGCGGCGTGCCTAATACATGCAAGTCGAGCGGACAGATGGGAG
CTTGCTCCCTGATGTTAGCGGCGGACGGGTGAGTAACACGTGGGTAACCTGCCTGTAAGACT
GGGATAACTCCGGGAAACCGGGGCTAATACCGGATGGTTGTTTGAACCGCATGGTTCAAAC
ATAAAAGGTGGCTTCGGCTACCACTTACAGATGGACCCGCGGCGCATTAGCTAGTTGGTGA
GGTAACGGCTCACCAAGGCAACGATGCGTAGCCGACCTGAGAGGGTGATCGGCCACACTGG
GACTGAGACACGGCCCAGACTCCTACGGGAGGCAGCAGTAGGGAATCTTCCGCAATGGAC
GAAAGTCTGACGGAGCAACGCCGCGTGAGTGATGAAGGTTTTCGGATCGTAAAGCTCTGTT
GTTAGGGAAGAACAAGTACCGTTCGAATAGGGCGGTACCTTGACGGTACCTAACCAGAAAG
CCACGGCTAACTACGTGCCAGCAGCCGCGGTAATACGTAGGTGGCAAGCGTTGTCCGGAAT
TATTGGGCGTAAAGGGCTCGCAGGCGGTTTCTTAAGTCTGATGTGAAAGCCCCCGGCTCAAC
CGGGGAGGGTCATTGGAAACTGGGGAACTTGAGTGCAGAAGAGGAGAGTGGAATTCCACG
TGTAGCGGTGAAATGCGTAGAGATGTGGAGGAACACCAGTGGCGAAGGCGACTCTCTGGTC

TGTAACTGACGCTGAGGAGCGAAAGCGTGGGGAGCGAACAGGATTAGATACCCTGGTAGTC
CACGCCGTAAACGATGAGTGCTAAGTGTTAGGGGGTTTCCGCCCCTTAGTGCTGCAGCTAAC
GCATTAAGCACTCCGCC
>BSNB_0817_ Bacillus_subtilis

GGCTCAGGACGAACGCTGGCGGCGTGCCTAATACATGCAAGTCGAGCGGACAGATGGGAG
CTTGCTCCCTGATGTTAGCGGCGGACGGGTGAGTAACACGTGGGTAACCTGCCTGTAAGACT
GGGATAACTCCGGGAAACCGGGGCTAATACCGGATGGTTGTTTGAACCGCATGGTTCAAAC
ATAAAAGGTGGCTTCGGCTACCACTTACAGATGGACCCGCGGCGCATTAGCTAGTTGGTGA
GGTAACGGCTCACCAAGGCAACGATGCGTAGCCGACCTGAGAGGGTGATCGGCCACACTGG
GACTGAGACACGGCCCAGACTCCTACGGGAGGCAGCAGTAGGGAATCTTCCGCAATGGAC
GAAAGTCTGACGGAGCAACGCCGCGTGAGTGATGAAGGTTTTCGGATCGTAAAGCTCTGTT
GTTAGGGAAGAACAAGTACCGTTCGAATAGGGCGGTACCTTGACGGTACCTAACCAGAAAG
CCACGGCTAACTACGTGCCAGCAGCCGCGGTAATACGTAGGTGGCAAGCGTTGTCCGGAAT
TATTGGGCGTAAAGGGCTCGCAGGCGGTTTCTTAAGTCTGATGTGAAAGCCCCCGGCTCAAC
CGGGGAGGGTCATTGGAAACTGGGGAACTTGAGTGCAGAAGAGGAGAGTGGAATTCCACG
TGTAGCGGTGAAATGCGTAGAGATGTGGAGGAACACCAGTGGCGAAGGCGACTCTCTGGTC
TGTAACTGACGCTGAGGAGCGAAAGCGTGGGGAGCGAACAGGATTAGATACCCTGGTAGTC
CACGCCGTAAACGATGAGTGCTAAGTGTTAGGGGGTTTCCGCCCCTTAGTGCTGCAGCTAAC
GCATTAAGCACTCCGCC
>BSNB_0955_ Bacillus_subtilis

GGCTCAGGACGAACGCTGGCGGCGTGCCTAATACATGCAAGTCGAGCGGACAGATGGGAG
CTTGCTCCCTGATGTTAGCGGCGGACGGGTGAGTAACACGTGGGTAACCTGCCTGTAAGACT
GGGATAACTCCGGGAAACCGGGGCTAATACCGGATGGTTGTTTGAACCGCATGGTTCAAAC
ATAAAAGGTGGCTTCGGCTACCACTTACAGATGGACCCGCGGCGCATTAGCTAGTTGGTGA
GGTAACGGCTCACCAAGGCAACGATGCGTAGCCGACCTGAGAGGGTGATCGGCCACACTGG
GACTGAGACACGGCCCAGACTCCTACGGGAGGCAGCAGTAGGGAATCTTCCGCAATGGAC
GAAAGTCTGACGGAGCAACGCCGCGTGAGTGATGAAGGTTTTCGGATCGTAAAGCTCTGTT
GTTAGGGAAGAACAAGTACCGTTCGAATAGGGCGGTACCTTGACGGTACCTAACCAGAAAG
CCACGGCTAACTACGTGCCAGCAGCCGCGGTAATACGTAGGTGGCAAGCGTTGTCCGGAAT
TATTGGGCGTAAAGGGCTCGCAGGCGGTTTCTTAAGTCTGATGTGAAAGCCCCCGGCTCAAC
CGGGGAGGGTCATTGGAAACTGGGGAACTTGAGTGCAGAAGAGGAGAGTGGAATTCCACG
TGTAGCGGTGAAATGCGTAGAGATGTGGAGGAACACCAGTGGCGAAGGCGACTCTCTGGTC
TGTAACTGACGCTGAGGAGCGAAAGCGTGGGGAGCGAACAGGATTAGATACCCTGGTAGTC
CACGCCGTAAACGATGAGTGCTAAGTGTTAGGGGGTTTCCGCCCCTTAGTGCTGCAGCTAAC
GCATTAAGCACTCCGCC
>BSNB_0956_ Bacillus_subtilis

GGCTCAGGACGAACGCTGGCGGCGTGCCTAATACATGCAAGTCGAGCGGACAGATGGGAG
CTTGCTCCCTGATGTTAGCGGCGGACGGGTGAGTAACACGTGGGTAACCTGCCTGTAAGACT
GGGATAACTCCGGGAAACCGGGGCTAATACCGGATGGTTGTTTGAACCGCATGGTTCAAAC
ATAAAAGGTGGCTTCGGCTACCACTTACAGATGGACCCGCGGCGCATTAGCTAGTTGGTGA
GGTAACGGCTCACCAAGGCAACGATGCGTAGCCGACCTGAGAGGGTGATCGGCCACACTGG
GACTGAGACACGGCCCAGACTCCTACGGGAGGCAGCAGTAGGGAATCTTCCGCAATGGAC
GAAAGTCTGACGGAGCAACGCCGCGTGAGTGATGAAGGTTTTCGGATCGTAAAGCTCTGTT
GTTAGGGAAGAACAAGTACCGTTCGAATAGGGCGGTACCTTGACGGTACCTAACCAGAAAG
CCACGGCTAACTACGTGCCAGCAGCCGCGGTAATACGTAGGTGGCAAGCGTTGTCCGGAAT
TATTGGGCGTAAAGGGCTCGCAGGCGGTTTCTTAAGTCTGATGTGAAAGCCCCCGGCTCAAC
CGGGGAGGGTCATTGGAAACTGGGGAACTTGAGTGCAGAAGAGGAGAGTGGAATTCCACG
TGTAGCGGTGAAATGCGTAGAGATGTGGAGGAACACCAGTGGCGAAGGCGACTCTCTGGTC

TGTAACTGACGCTGAGGAGCGAAAGCGTGGGGAGCGAACAGGATTAGATACCCTGGTAGTC
CACGCCGTAAACGATGAGTGCTAAGTGTTAGGGGGTTTCCGCCCCTTAGTGCTGCAGCTAAC
GCATTAAGCACTCCGCC
>BSNB_0959_ Bacillus_subtilis

GGCTCAGGACGAACGCTGGCGGCGTGCCTAATACATGCAAGTCGAGCGGACAGATGGGAG
CTTGCTCCCTGATGTTAGCGGCGGACGGGTGAGTAACACGTGGGTAACCTGCCTGTAAGACT
GGGATAACTCCGGGAAACCGGGGCTAATACCGGATGGTTGTTTGAACCGCATGGTTCAAAC
ATAAAAGGTGGCTTCGGCTACCACTTACAGATGGACCCGCGGCGCATTAGCTAGTTGGTGA
GGTAACGGCTCACCAAGGCAACGATGCGTAGCCGACCTGAGAGGGTGATCGGCCACACTGG
GACTGAGACACGGCCCAGACTCCTACGGGAGGCAGCAGTAGGGAATCTTCCGCAATGGAC
GAAAGTCTGACGGAGCAACGCCGCGTGAGTGATGAAGGTTTTCGGATCGTAAAGCTCTGTT
GTTAGGGAAGAACAAGTACCGTTCGAATAGGGCGGTACCTTGACGGTACCTAACCAGAAAG
CCACGGCTAACTACGTGCCAGCAGCCGCGGTAATACGTAGGTGGCAAGCGTTGTCCGGAAT
TATTGGGCGTAAAGGGCTCGCAGGCGGTTTCTTAAGTCTGATGTGAAAGCCCCCGGCTCAAC
CGGGGAGGGTCATTGGAAACTGGGGAACTTGAGTGCAGAAGAGGAGAGTGGAATTCCACG
TGTAGCGGTGAAATGCGTAGAGATGTGGAGGAACACCAGTGGCGAAGGCGACTCTCTGGTC
TGTAACTGACGCTGAGGAGCGAAAGCGTGGGGAGCGAACAGGATTAGATACCCTGGTAGTC
CACGCCGTAAACGATGAGTGCTAAGTGTTAGGGGGTTTCCGCCCCTTAGTGCTGCAGCTAAC
GCATTAAGCACTCCGCC
>BSNB_0978_ Acidisoma_sp

AGAATAAGCCCCGGATAACTTTGTGCCAGCAGCCGCGGTAATACGAAGGGGGCTAGCGTTG
CTCGGAATGATTGGGTGTAAAGGGCGCGTAGGCGGCGGCCAAAGTCAGGCGTGAAATTCCT
GGGCTCAACCTGGGGGCTGCGCTTGATACTTGGTTGCTTGAGTGGGGAAGAGGGTCGTGGA
ATTCCCAGTGTAGAGGTGAAATTCGTAGATATTGGGAAGAACACCGGTGGCGAAGGCGGCG
ACCTGGTCCTTGACTGACGCTGAGGCGCGAAAGCGTGGGGAGCAAACAGGATTAGATACCC
TGGTAGTCCACGCTGTAAACGATGTGTGCTGGATGTTGGGTGACTTAGTCACTCAGTGTCGTA
GCTAACGCGATAAGCATCA

>BSNB_1020_ Burkholderia_tropica

CCAGGCGGTCACTTCACGCGTTAGCTACGTTACCAAGTCAATGAAGACCCGACAACCAGTT
GACATCGTTTAGGGCGTGGACTACCAGGGTATCTAATCCTGTTTGCTCCCCACGCTTTCGTGC
ATGAGCGTCAGTATTGACCCAGGGGGCTGCCTTCGCCATCGGTATTCCTCCACATCTCTACG
CATTTCACTGCTACACGTGGAATTCTACCCCCCTCTGCCATACTCAAGCGATGCAGTCACCA
ATGCAGTTCCCAGGTTGAGCCCGGGGATTTCACATCGGTCTTACATCACCGCCTGCGCACGC
TTTACGCCCAGTAATTCCGATTAACGCTTGCACCCTACGTATTACCGCGGCTGCTGGCACGTA
GTTAGCCGGTGCTTATTCTTCCGGTACCGTCATCCCCCGACCATATTAGGATCAAGGATTTCT
TTCCGGACAAAAGTGCTTTACAACCCGAAGGCCTTCTTCACACACGCGGCATTGCTGGATCA
GGCTTTCGCCCATTGTCCAAAATTCCCCACTGCTGCCTCCCGTAGGAGTCTGGGCCGTGTCTC
AGTCCCAGTGTGGCTGGTCGTCCTCTCAGACCAGCTACGGATCGTCGCCTTGGTGAGCCTTTA
CCTCACCAACTAGCTAATCCGCCATCGGCCGCCCCTATAGCGCGAGGTCCGAAGATCCCCC
GCTTTCATCCGTAGATCGTATGCGGTATTAATCCGGCTTTCGCCGGGCTATCCCCCACTACAG
GACACGTTCCGATGTATTACTCACCCGTTCGCCACTCGCCACCAGGTGCAAGCACCCGTGCT
GCCGTTCGACTTGCATGTGTAAGGCATGCCGCCAGCGTTCAATCTGAGCCATGAATCA

>BSNB_1022_ Methylobacterium_populi

CCCCTATCTTCCGGTMCGTCATTATCGTCCCGGACAAAAGAGCTTTACAACCCTAAGGCCTT
CATCACTCACGCGGCATGGCTGGATCAGGCTTGCGCCCATTGTCCAATATTCCCCACTGCTG

CCTCCCGTAGGAGTCTGGGCCGTGTCTCAGTCCCAGTGTGGCTGATCATCCTCTCAGACCAG
CTACTGATCGTCGCCTTGGTAGGCCGTTACCCCACCAACAAGCTAATCAGACGCGGGCCGAT
CCTTCGGCAGTAAACCTTTCCCCATAAGGGCGTATCCGGTATTAGCTCAAGTTTCCCTGAGTT
ATTCCGAACCGAAGGGCACGTTCCCACGTGTTACTCACCCGTCTGCCACTGACTTCCGAAGA
AGCCCGTTCGACTTGCATGTGTTAAGCCTGCCGCCAGCGTTCGCTCTGAGCCAGGATCAAAC
TCTA

>BSNB_1023_ Curtobacterium_sp

CTTCTTTCTGCAGGTACGTCACTTTCGCTTCTTCCCTACTAAAAGAGGTTTACAACCCGAAGG
CCGTCATCCCTCACGCGGCGTTGCTGCATCAGGCTTTCGCCCATTGTGCAATATTCCCCACTG
CTGCCTCCCGTAGGAGTCTGGGCCGTGTCTCAGTCCCAGTGTGGCCGGTCACCCTCTCAGGC
CGGCTACCCGTCGTCGCCTTGGTGAGCCATTACCTCACCAACAAGCTGATAGGCCGCGAGTC
CATCCCCAACCAAAAAATCTTTCCACCACCAGACCATGCGGCCGGCAGTCCTATCCAGTATT
AGACGTCGTTTCCAACGCTTATCCCAGAGTCAAGGGCAGGTTACTCACGTGTTACTCACCCG
TTCGCCACTAATCCACCCAGCAAGCTGGGCATCATCGTTCGACTTGCATGTGTTAAGCACGC
CGCCAGCGTTCGTCCTGAGCCAGGTYAAAACTCTA
>BSNB_1024_ Pantoea_dispersa

GGTCGGTCTCTCTGCGGGTAACGTCAATCGGCGAGGTTATTAACCTCACCGCCTTCCTCCCCG
CTGAAAGTACTTTACAACCCGAAGGCCTTCTTCATACACGCGGCATGGCTGCATCAGGCTTG
CGCCCATTGTGCAATATTCCCCACTGCTGCCTCCCGTAGGAGTCTGGACCGTGTCTCAGTTCC
AGTGTGGCTGGTCATCCTCTCAGACCAGCTAGGGATCGTCGCCTAGGTGAGCCATTACCCCA
CCTACTAGCTAATCCCATCTGGGCACATCCGATGGTGTGAGGCCCGAAGGTCCCCCACTTTG
GTCTTGCGACGTTATGCGGTATTAGCTACCGTTTCCAGTAGTTATCCCCCTCCATCGGGCAGT
TTCCCAGACATTACTCACCCGTCCGCCACTCGCCACCCAAAGAGCAAGCTCTTCTGTGCTGC
CGTTCGACTTGCATGTGTTAGGCCTGCCGCCAGCGTTCAATATGAGCCAGGTYCAAAAACTC
TA
>BSNB_1025_ Bacillus_megaterium

ATCGTGCTTCTGGTAGGTACCGTCAAGGTACGAGCAGTTACTCTCGTACTTGTTCTTCCCTAA
CAACAGAGTTTTACGACCCGAAAGCCTTCATCACTCACGCGGCGTTGCTCCGTCAGACTTTC
GTCCATTGCGGAAGATTCCCTACTGCTGCCTCCCGTAGGAGTCTGGGCCGTGTCTCAGTCCC
AGTGTGGCCGATCACCCTCTCAGGTCGGCTATGCATCGTTGCCTTGGTGAGCCGTTACCTCAC
CAACTAGCTAATGCACCGCGGGCCCATCTGTAAGTGATAGCCGAAACCATCTTTCAATCATC
TCCCATGAAGGAGAAGATCCTATCCGGTATTAGCTTCGGTTTCCCGAAGTTATCCCAGTCTTA
CAGGCAGGTTGCCCACGTGTTACTCACCCGTCCGCCGCTAACGTCATAGAAGCAAGCTTCTA
ATCAGTTCGCTCGACTTGCATGTATTAGGCACGCCGCCAGCGTTCATCCTGAGCCAGGTYMA
AAACTCTA
>BSNB_1026_ Methylobacterium_sp

CTCTCCTATCTCGGTACGTCATTATCGTCCCGGATAAAAGAGCTTTACAACCCTAAGGCCTTC
ATCACTCACGCGGCATGGCTGGATCAGGCTTGCGCCCATTGTCCAATATTCCCCACTGCTGC
CTCCCGTAGGAGTCTGGGCCGTGTCTCAGTCCCAGTGTGGCTGATCATCCTCTCAGACCAGC
TACTGATCGTCGCCTTGGTAGGCCGTTACCCCACCAACTAGCTAATCAGACGCGGGCCGATC
TTCCGGCAGTAAACCTTTCCCCATAAGGGCGTATCCGGTATTAGCCCTAGTTTCCCAGGGTTA
TTCCGAACCGGAAGGCACGTTCCCACGCGTTACTCACCCGTCCGCCGCTGACCCCGAAGGG
CCCGCTCGACTTGCATGTGTTAAGCCTGCCGCCAGCGTTCGCTCTGAGCCAGGATCAAACTC
TAA
>BSNB_1027_ Asaia_sp

GTCTGGCTCTTCTACGGGTACCGTCATCATCGTCCCCGTCGAAAGTGCTTTACAATCCGAAG
ACCTTCTTCACACACGCGGCATTGCTGGATCAGGCTTTCGCCCATTGTCCAATATTCCCCACT
GCTGCCTCCCGTAGGAGTCTGGGCCGTGTCTCAGTCCCAGTGTGGCTGATCATCCTCTCAGAC
CAGCTATCGATCATCGCCTTGGTAGGCCTTTACCCCACCAACAAGCTAATCGAACGCAGGCT
CCTCCACAGGCGACTTGCGCCTTTGACCCTCAGGTATCATGCGGTATTAGCACCAGTTTCCCG
ATGTTATCCCCCACCCGTGGATAGATCCCTACGCGATACTCACCCGTCCGCCACTCACCCCG
AAGGGTCCGTGCGACTTGCATGTGTTAAGCATGCCGCCAGCGTTCGCTCTGAGCCAGATCAA
AACTCTA
>BSNB_1030_ Pseudomonas_sp

CTTATTCTGTCGGTACGTCAAAACTCACAGGTATTCGCTGTAAGCCCTTCCTCCCAACTTAAA
GTGCTTTACGACCCGARGGCCTTCTTCACACACGCGGCATGGCTGGATCAGGCTTTCGCCCA
TTGTCCAATATTCCCCACTGCTGCCTCCCGTAGGAGTCTGGACCGTGTCTCAGTTCCAGTGTG
ACTGATCATCCTCTCAGACCAGTTACGGATCGTCGCCTTGGTAGGCCTTTACCCTACCAACTA
GCTAATCCGACCTAGGCTCATCTAATAGCGTGAGGTCCGAAGATCCCCCACTTTCTCCCGTA
GGACGTATGCGGTATTAGCGTTCCTTTCGAAACGTTGTCCCCCACTACTAGGCAGATTCCTAG
GCATTACTCACCCGTCCGCCGCTGAATCGARGAGCAAGCTCCTCTCATCCGCTCGACTTGCA
TGTGTTAGGCCTGCCGCCAGCGTTCAATCTRAGCCAGGATCAAAACTCTA
>BSNB_1032_ Arthrobacter_sp

CCGGCCTCTTCTGCAGGTACCGTCACTTTCGCTTCTTCCCTACTGAAAGAGGTTTACAACCCG
AAGGCCGTCATCCCTCACGCGGCGTCGCTGCATCAGGCTTGCGCCCATTGTGCAATATTCCC
CACTGCTGCCTCCCGTAGGAGTCTGGGCCGTGTCTCAGTCCCAGTGTGGCCGGTCACCCTCTC
AGGCCGGCTACCCGTCGTCGCCTTGGTAGGCCATTACCCCACCAACAAGCTGATAGGCCGC
GAGTCCATCCAAAACCACAAAAGCTTTCCACCACCATGACATGCGCCAGATGGTCGTATCC
GGTATTAGACCCAGTTTCCCAGGCTTATCCCAGAGTCAAGGGCAGGTTACTCACGTGTTACT
CACCCGTTCGCCACTAATCCCCCAGCAAGCTGGGATCATCGTTCGACTTGCATGTGTTAAGC
ACGCCGCCAGCGTTCATCCTGAGCCAGGATCAAACTCTA
>BSNB_1034_ Pantoea_agglomerans

GGGTGCGGGGCTCTTCTGCGGGTACGTCATCGACASGGTTATTAACCCCRTCGCCTTCCTCCC
CGCTGAAAGTACTTTACAACCCGAAGGCCTTCTTCATACACGCGGCATGGCTGCATCAGGCT
TGCGCCCATTGTGCAATATTCCCCACTGCTGCCTCCCGTAGGAGTCTGGACCGTGTCTCAGTT
CCAGTGTGGCTGGTCATCCTCTCAGACCAGCTAGGGATCGTCGCCTAGGTGGGCCATTACCC
CGCCTACTAGCTAATCCCATCTGGGTTCATCCGATAGTGAGAGGCCCGAAGGTCCCCCTCTT
TGGTCTTGCGACGTTATGCGGTATTAGCCACCGTTTCCAGTGGTTATCCCCCTCTATCGGGCA
GATCCCCAGACATTACTCACCCGTCCGCCACTCGTCACCCAAGRRCMAGCTYTCTSTGCKAC
CGYCCSACWTGCACGTGTKAGGCSTGCCSCCMSCGTTCAAWMTSAGMCMSGGCAAAAAMTC
TSAAAA
>BSNB_1116_ Methylobacterium_mesophilicum

CCGGATACGCCCTTTTGGGGAAAGGTTTACTGCCGGAAGATCGGCCCGCGTCTGATTAGCTA
GTTGGTGGGGTAACGGCCTACCAAGGCGACGATCAGTAGCTGGTCTGAGAGGATGATCAGC
CACACTGGGACTGAGACACGGCCCAGACTCCTACGGGAGGCAGCAGTGGGGAATATTGGA
CAATGGGCGCAAGCCTGATCCAGCCATGCCGCGTGAGTGATGAAGGCCTTAGGGTTGTAAA
GCTCTTTTATCCGGGACGATAATGACGGTACCGGAGGAATAAGCCCCGGCTAACTTCGTGCC
AGCAGCCGCGGTAATACGAAGGGGGCTAGCGTTGCTCGGAATCACTGGGCGTAAAGGGCGC
GTAGGCGGCGTTTTAAGTCGGGGGTGAAAGCCTGTGGCTCAACCACAGAATGGCCTTCGAT
ACTGGGACGCTTGAGTATGGTAGAGGTTGGTGGAACTGCGAGTGTAGAGGTGAAATTCGTA
GATATTCGCAAGAACACCGGTGGCGAAGGCGGCCAACTGGACCATTACTGACGCTGAGGCG

CGAAAGCGTGGGGAGCAAACAGGATTAGATACCCTGGTAGTCCACGCCGTAAACGATGAAT
GCCAGCTGTTGGGGTGCTTGCACCGCAGTAGCGCAGCTAACGCTTTGAGCATCCGCC
>BSNB_1123_ Methylobacterium_mesophilicum

CCGGATACGCCCTTTTGGGGAAAGGTTTACTGCCGGAAGATCGGCCCGCGTCTGATTAGCTA
GTTGGTGGGGTAACGGCCTACCAAGGCGACGATCAGTAGCTGGTCTGAGAGGATGATCAGC
CACACTGGGACTGAGACACGGCCCAGACTCCTACGGGAGGCAGCAGTGGGGAATATTGGA
CAATGGGCGCAAGCCTGATCCAGCCATGCCGCGTGAGTGATGAAGGCCTTAGGGTTGTAAA
GCTCTTTTATCCGGGACGATAATGACGGTACCGGAGGAATAAGCCCCGGCTAACTTCGTGCC
AGCAGCCGCGGTAATACGAAGGGGGCTAGCGTTGCTCGGAATCACTGGGCGTAAAGGGCGC
GTAGGCGGCGTTTTAAGTCGGGGGTGAAAGCCTGTGGCTCAACCACAGAATGGCCTTCGAT
ACTGGGACGCTTGAGTATGGTAGAGGTTGGTGGAACTGCGAGTGTAGAGGTGAAATTCGTA
GATATTCGCAAGAACACCGGTGGCGAAGGCGGCCAACTGGACCATTACTGACGCTGAGGCG
CGAAAGCGTGGGGAGCAAACAGGATTAGATACCCTGGTAGTCCACGCCGTAAACGATGAAT
GCCAGCTGTTGGGGTGCTTGCACCGCAGTAGCGCAGCTAACGCTTTGAGCATCCGCC
>BSNB_1136_ Methylobacterium_mesophilicum

CCGGATACGCCCTTTTGGGGAAAGGTTTACTGCCGGAAGATCGGCCCGCGTCTGATTAGCTA
GTTGGTGGGGTAACGGCCTACCAAGGCGACGATCAGTAGCTGGTCTGAGAGGATGATCAGC
CACACTGGGACTGAGACACGGCCCAGACTCCTACGGGAGGCAGCAGTGGGGAATATTGGA
CAATGGGCGCAAGCCTGATCCAGCCATGCCGCGTGAGTGATGAAGGCCTTAGGGTTGTAAA
GCTCTTTTATCCGGGACGATAATGACGGTACCGGAGGAATAAGCCCCGGCTAACTTCGTGCC
AGCAGCCGCGGTAATACGAAGGGGGCTAGCGTTGCTCGGAATCACTGGGCGTAAAGGGCGC
GTAGGCGGCGTTTTAAGTCGGGGGTGAAAGCCTGTGGCTCAACCACAGAATGGCCTTCGAT
ACTGGGACGCTTGAGTATGGTAGAGGTTGGTGGAACTGCGAGTGTAGAGGTGAAATTCGTA
GATATTCGCAAGAACACCGGTGGCGAAGGCGGCCAACTGGACCATTACTGACGCTGAGGCG
CGAAAGCGTGGGGAGCAAACAGGATTAGATACCCTGGTAGTCCACGCCGTAAACGATGAAT
GCCAGCTGTTGGGGTGCTTGCACCGCAGTAGCGCAGCTAACGCTTTGAGCATCCGCC
>BSNB_1137_ Methylobacterium_mesophilicum

CCGGATACGCCCTTTTGGGGAAAGGTTTACTGCCGGAAGATCGGCCCGCGTCTGATTAGCTA
GTTGGTGGGGTAACGGCCTACCAAGGCGACGATCAGTAGCTGGTCTGAGAGGATGATCAGC
CACACTGGGACTGAGACACGGCCCAGACTCCTACGGGAGGCAGCAGTGGGGAATATTGGA
CAATGGGCGCAAGCCTGATCCAGCCATGCCGCGTGAGTGATGAAGGCCTTAGGGTTGTAAA
GCTCTTTTATCCGGGACGATAATGACGGTACCGGAGGAATAAGCCCCGGCTAACTTCGTGCC
AGCAGCCGCGGTAATACGAAGGGGGCTAGCGTTGCTCGGAATCACTGGGCGTAAAGGGCGC
GTAGGCGGCGTTTTAAGTCGGGGGTGAAAGCCTGTGGCTCAACCACAGAATGGCCTTCGAT
ACTGGGACGCTTGAGTATGGTAGAGGTTGGTGGAACTGCGAGTGTAGAGGTGAAATTCGTA
GATATTCGCAAGAACACCGGTGGCGAAGGCGGCCAACTGGACCATTACTGACGCTGAGGCG
CGAAAGCGTGGGGAGCAAACAGGATTAGATACCCTGGTAGTCCACGCCGTAAACGATGAAT
GCCAGCTGTTGGGGTGCTTGCACCGCAGTAGCGCAGCTAACGCTTTGAGCATCCGCC
>BSNB_1141_ Methylobacterium_mesophilicum

CCGGATACGCCCTTTTGGGGAAAGGTTTACTGCCGGAAGATCGGCCCGCGTCTGATTAGCTA
GTTGGTGGGGTAACGGCCTACCAAGGCGACGATCAGTAGCTGGTCTGAGAGGATGATCAGC
CACACTGGGACTGAGACACGGCCCAGACTCCTACGGGAGGCAGCAGTGGGGAATATTGGA
CAATGGGCGCAAGCCTGATCCAGCCATGCCGCGTGAGTGATGAAGGCCTTAGGGTTGTAAA
GCTCTTTTATCCGGGACGATAATGACGGTACCGGAGGAATAAGCCCCGGCTAACTTCGTGCC
AGCAGCCGCGGTAATACGAAGGGGGCTAGCGTTGCTCGGAATCACTGGGCGTAAAGGGCGC
GTAGGCGGCGTTTTAAGTCGGGGGTGAAAGCCTGTGGCTCAACCACAGAATGGCCTTCGAT
ACTGGGACGCTTGAGTATGGTAGAGGTTGGTGGAACTGCGAGTGTAGAGGTGAAATTCGTA

GATATTCGCAAGAACACCGGTGGCGAAGGCGGCCAACTGGACCATTACTGACGCTGAGGCG
CGAAAGCGTGGGGAGCAAACAGGATTAGATACCCTGGTAGTCCACGCCGTAAACGATGAAT
GCCAGCTGTTGGGGTGCTTGCACCGCAGTAGCGCAGCTAACGCTTTGAGCATCCGCC
>BSNB_1179_ Rhodococcus_kroppenstedtii

CCGCTTAATGCGTTAGCTACGGCACGGATCCCGTGGAAGGAAACCCACACCTAGCGCCCAC
CGTTTACGGCGTGGACTACCAGGGTATCTAATCCTGTTCGCTACCCACGCTTTCGCTCCTCAG
CGTCAGTTATTTCCCAGAGACCCGCCTTCGCCACCGGTGTTCCTCCTGATATCTGCGCATTTC
ACCGCTACACCAGGAATTCCAGTCTCCCCTGAAATACTCAAGTCTGCCCGTATCGCCTGCAA
GCCAACAGTTGAGCTGCTGGTTTTCACAGACGACGCGACAAACCGCCTACGAGCTCTTTACG
CCCAGTAATTCCGGACAACGCTTGCACCCTACGTATTACCGCGGCTGCTGGCACGTAGTTGG
CCGGTGCTTCTTCTGCAGGTACCGTCACTCACGCTTCGTCCCTGCTGAAAGAGGTTTACAACC
CGAAGGCCGTCATCCCTCACGCGGCGTCGCTGCATCAGGCTTGCGCCCATTGTGCAATATTC
CCCACTGCTGCCTCCCGTAGGAGTCTGGGCCGTGTCTCAGTCCCAGTGTGGCCGGTCGCCCT
CTCAGGCCGGCTACCCGTCGTCGCCTTGGTAGGCCATTACCCCACCAACAAGCTGATAGGCC
GCGGGCCCATCCTGCACCGATAAATCTTTCCACCACACGGCATGCACCGCGCAGTCCTATCC
GGTATTAGACCCAGTTTCCCGGGCTTATCCCGAAGTGCAGGGCAGATCACCCACGTGTTACT
CACCCGTTCGCCACTCGTGTACCCCGAAGGGCCTTACCGTTCGACTTGCATGTGTTAAAGCA
CGCCGCCAGCGTTCGTCCTGA
>BSNB_1190_ Rhodococcus_kroppenstedtii

CCGCTTAATGCGTTAGCTACGGCACGGATCCCGTGGAAGGAAACCCACACCTAGCGCCCAC
CGTTTACGGCGTGGACTACCAGGGTATCTAATCCTGTTCGCTACCCACGCTTTCGCTCCTCAG
CGTCAGTTATTTCCCAGAGACCCGCCTTCGCCACCGGTGTTCCTCCTGATATCTGCGCATTTC
ACCGCTACACCAGGAATTCCAGTCTCCCCTGAAATACTCAAGTCTGCCCGTATCGCCTGCAA
GCCAACAGTTGAGCTGCTGGTTTTCACAGACGACGCGACAAACCGCCTACGAGCTCTTTACG
CCCAGTAATTCCGGACAACGCTTGCACCCTACGTATTACCGCGGCTGCTGGCACGTAGTTGG
CCGGTGCTTCTTCTGCAGGTACCGTCACTCACGCTTCGTCCCTGCTGAAAGAGGTTTACAACC
CGAAGGCCGTCATCCCTCACGCGGCGTCGCTGCATCAGGCTTGCGCCCATTGTGCAATATTC
CCCACTGCTGCCTCCCGTAGGAGTCTGGGCCGTGTCTCAGTCCCAGTGTGGCCGGTCGCCCT
CTCAGGCCGGCTACCCGTCGTCGCCTTGGTAGGCCATTACCCCACCAACAAGCTGATAGGCC
GCGGGCCCATCCTGCACCGATAAATCTTTCCACCACACGGCATGCACCGCGCAGTCCTATCC
GGTATTAGACCCAGTTTCCCGGGCTTATCCCGAAGTGCAGGGCAGATCACCCACGTGTTACT
CACCCGTTCGCCACTCGTGTACCCCGAAGGGCCTTACCGTTCGACTTGCATGTGTTAAAGCA
CGCCGCCAGCGTTCGTCCTGA
>BSNB_1191_ Microbacterium_testaceum

GGGATAAGCGCTGGAAACGGTGTCTAATACTGGATATGAGCCTCTTCCGCATGGTGGGGGTT
GGAAAGATTTTTCGGTCTGGGATGGGCTCGCGGCCTATCAGCTTGTTGGTGAGGTAATGGCT
CACCAAGGCGTCGACGGGTAGCCGGCCTGAGAGGGTGACCGGCCACACTGGGACTGAGAC
ACGGCCCAGACTCCTACGGGAGGCAGCAGTGGGGAATATTGCACAATGGGCGGAAGCCTG
ATGCAGCAACGCCGCGTGAGGGATGACGGCCTTCGGGTTGTAAACCTCTTTTAGCAGGGAA
GAAGCGGAAGTGACGGTACCTGCAGAAAAAGCGCCGGCTAACTACGTGCCAGCAGCCGCG
GTAATACGTAGGGCGCAAGCGTTATCCGGAATTATTGGGCGTAAAGAGCTCGTAGGCGGTTT
GTCGCGTCTGCTGTGAAATCCCGAGGCTCAACCTCGGGCCTGCAGTGGGTACGGGCAGACT
AGAGTGCGGTAGGGGAGATTGGAATTCCTGGTGTAGCGGTGGAATGCGCAGATATCAGGAG
GAACACCGATGGCGAAGGCAGATCTCTGGGCCGTAACTGACGCTGAGGAGCGAAAGGGTG
GGGAGCAAACAGGCTTAGATACCCTGGTAGTCCACCCCGTAAACGTTGGGAACTAGTTGTG
GGGACCATTCCACGGTTTCCGTGACGCAGCTAACGCATTAAGT
>BSNB_1192_ Microbacterium_testaceum

AGATTTTTCGGTCTGGGATGGGCTCGCGGCCTATCAGCTTGTTGGTGAGGTAATGGCTCACC
AAGGCGTCGACGGGTAGCCGGCCTGAGAGGGTGACCGGCCACACTGGGACTGAGACACGG
CCCAGACTCCTACGGGAGGCAGCAGTGGGGAATATTGCACAATGGGCGGAAGCCTGATGCA
GCAACGCCGCGTGAGGGATGACGGCCTTCGGGTTGTAAACCTCTTTTAGCAGGGAAGAAGC
GAAAGTGACGGTACCTGCAGAAAAAGCGCCGGCTAACTACGTGCCAGCAGCCGCGGTAAT
ACGTAGGGCGCAAGCGTTATCCGGAATTATTGGGCGTAAAGAGCTCGTAGGCGGTTTGTCGC
GTCTGCTGTGAAATCCCGAGGCTCAACCTCGGGCCTGCAGTGGGTACGGGCAGACTAGAGT
GCGGTAGGGGAGATTGGAATTCCTGGTGTAGCGGTGGAATGCGCAGATATCAGGAGGAACA
CCGATGGCGAAGGCAGATCTCTGGGCCGTAACTGACGCTGAGGAGCGAAAGGGTGGGGAG
CAAACAGGCTTAGATACCCTGGTAGTCCACCCCGTAAACGTTGGGAACTAGTTGTGGGGAC
C
>BSNB_1207_ Bacillus_subtilis

GGCTCAGGACGAACGCTGGCGGCGTGCCTAATACATGCAAGTCGAGCGGACAGATGGGAG
CTTGCTCCCTGATGTTAGCGGCGGACGGGTGAGTAACACGTGGGTAACCTGCCTGTAAGACT
GGGATAACTCCGGGAAACCGGGGCTAATACCGGATGGTTGTTTGAACCGCATGGTTCAAAC
ATAAAAGGTGGCTTCGGCTACCACTTACAGATGGACCCGCGGCGCATTAGCTAGTTGGTGA
GGTAACGGCTCACCAAGGCAACGATGCGTAGCCGACCTGAGAGGGTGATCGGCCACACTGG
GACTGAGACACGGCCCAGACTCCTACGGGAGGCAGCAGTAGGGAATCTTCCGCAATGGAC
GAAAGTCTGACGGAGCAACGCCGCGTGAGTGATGAAGGTTTTCGGATCGTAAAGCTCTGTT
GTTAGGGAAGAACAAGTACCGTTCGAATAGGGCGGTACCTTGACGGTACCTAACCAGAAAG
CCACGGCTAACTACGTGCCAGCAGCCGCGGTAATACGTAGGTGGCAAGCGTTGTCCGGAAT
TATTGGGCGTAAAGGGCTCGCAGGCGGTTTCTTAAGTCTGATGTGAAAGCCCCCGGCTCAAC
CGGGGAGGGTCATTGGAAACTGGGGAACTTGAGTGCAGAAGAGGAGAGTGGAATTCCACG
TGTAGCGGTGAAATGCGTAGAGATGTGGAGGAACACCAGTGGCGAAGGCGACTCTCTGGTC
TGTAACTGACGCTGAGGAGCGAAAGCGTGGGGAGCGAACAGGATTAGATACCCTGGTAGTC
CACGCCGTAAACGATGAGTGCTAAGTGTTAGGGGGTTTCCGCCCCTTAGTGCTGCAGCTAAC
GCATTAAGCACTCCGCC
>BSNB_1208_ Bacillus_subtilis

GGCTCAGGACGAACGCTGGCGGCGTGCCTAATACATGCAAGTCGAGCGGACAGATGGGAG
CTTGCTCCCTGATGTTAGCGGCGGACGGGTGAGTAACACGTGGGTAACCTGCCTGTAAGACT
GGGATAACTCCGGGAAACCGGGGCTAATACCGGATGGTTGTTTGAACCGCATGGTTCAAAC
ATAAAAGGTGGCTTCGGCTACCACTTACAGATGGACCCGCGGCGCATTAGCTAGTTGGTGA
GGTAACGGCTCACCAAGGCAACGATGCGTAGCCGACCTGAGAGGGTGATCGGCCACACTGG
GACTGAGACACGGCCCAGACTCCTACGGGAGGCAGCAGTAGGGAATCTTCCGCAATGGAC
GAAAGTCTGACGGAGCAACGCCGCGTGAGTGATGAAGGTTTTCGGATCGTAAAGCTCTGTT
GTTAGGGAAGAACAAGTACCGTTCGAATAGGGCGGTACCTTGACGGTACCTAACCAGAAAG
CCACGGCTAACTACGTGCCAGCAGCCGCGGTAATACGTAGGTGGCAAGCGTTGTCCGGAAT
TATTGGGCGTAAAGGGCTCGCAGGCGGTTTCTTAAGTCTGATGTGAAAGCCCCCGGCTCAAC
CGGGGAGGGTCATTGGAAACTGGGGAACTTGAGTGCAGAAGAGGAGAGTGGAATTCCACG
TGTAGCGGTGAAATGCGTAGAGATGTGGAGGAACACCAGTGGCGAAGGCGACTCTCTGGTC
TGTAACTGACGCTGAGGAGCGAAAGCGTGGGGAGCGAACAGGATTAGATACCCTGGTAGTC
CACGCCGTAAACGATGAGTGCTAAGTGTTAGGGGGTTTCCGCCCCTTAGTGCTGCAGCTAAC
GCATTAAGCACTCCGCC
>BSNB_1209_ Bacillus_subtilis

GGCTCAGGACGAACGCTGGCGGCGTGCCTAATACATGCAAGTCGAGCGGACAGATGGGAG
CTTGCTCCCTGATGTTAGCGGCGGACGGGTGAGTAACACGTGGGTAACCTGCCTGTAAGACT
GGGATAACTCCGGGAAACCGGGGCTAATACCGGATGGTTGTTTGAACCGCATGGTTCAAAC
ATAAAAGGTGGCTTCGGCTACCACTTACAGATGGACCCGCGGCGCATTAGCTAGTTGGTGA

GGTAACGGCTCACCAAGGCAACGATGCGTAGCCGACCTGAGAGGGTGATCGGCCACACTGG
GACTGAGACACGGCCCAGACTCCTACGGGAGGCAGCAGTAGGGAATCTTCCGCAATGGAC
GAAAGTCTGACGGAGCAACGCCGCGTGAGTGATGAAGGTTTTCGGATCGTAAAGCTCTGTT
GTTAGGGAAGAACAAGTACCGTTCGAATAGGGCGGTACCTTGACGGTACCTAACCAGAAAG
CCACGGCTAACTACGTGCCAGCAGCCGCGGTAATACGTAGGTGGCAAGCGTTGTCCGGAAT
TATTGGGCGTAAAGGGCTCGCAGGCGGTTTCTTAAGTCTGATGTGAAAGCCCCCGGCTCAAC
CGGGGAGGGTCATTGGAAACTGGGGAACTTGAGTGCAGAAGAGGAGAGTGGAATTCCACG
TGTAGCGGTGAAATGCGTAGAGATGTGGAGGAACACCAGTGGCGAAGGCGACTCTCTGGTC
TGTAACTGACGCTGAGGAGCGAAAGCGTGGGGAGCGAACAGGATTAGATACCCTGGTAGTC
CACGCCGTAAACGATGAGTGCTAAGTGTTAGGGGGTTTCCGCCCCTTAGTGCTGCAGCTAAC
GCATTAAGCACTCCGCC
>BSNB_1210_ Bacillus_subtilis

GGCTCAGGACGAACGCTGGCGGCGTGCCTAATACATGCAAGTCGAGCGGACAGATGGGAG
CTTGCTCCCTGATGTTAGCGGCGGACGGGTGAGTAACACGTGGGTAACCTGCCTGTAAGACT
GGGATAACTCCGGGAAACCGGGGCTAATACCGGATGGTTGTTTGAACCGCATGGTTCAAAC
ATAAAAGGTGGCTTCGGCTACCACTTACAGATGGACCCGCGGCGCATTAGCTAGTTGGTGA
GGTAACGGCTCACCAAGGCAACGATGCGTAGCCGACCTGAGAGGGTGATCGGCCACACTGG
GACTGAGACACGGCCCAGACTCCTACGGGAGGCAGCAGTAGGGAATCTTCCGCAATGGAC
GAAAGTCTGACGGAGCAACGCCGCGTGAGTGATGAAGGTTTTCGGATCGTAAAGCTCTGTT
GTTAGGGAAGAACAAGTACCGTTCGAATAGGGCGGTACCTTGACGGTACCTAACCAGAAAG
CCACGGCTAACTACGTGCCAGCAGCCGCGGTAATACGTAGGTGGCAAGCGTTGTCCGGAAT
TATTGGGCGTAAAGGGCTCGCAGGCGGTTTCTTAAGTCTGATGTGAAAGCCCCCGGCTCAAC
CGGGGAGGGTCATTGGAAACTGGGGAACTTGAGTGCAGAAGAGGAGAGTGGAATTCCACG
TGTAGCGGTGAAATGCGTAGAGATGTGGAGGAACACCAGTGGCGAAGGCGACTCTCTGGTC
TGTAACTGACGCTGAGGAGCGAAAGCGTGGGGAGCGAACAGGATTAGATACCCTGGTAGTC
CACGCCGTAAACGATGAGTGCTAAGTGTTAGGGGGTTTCCGCCCCTTAGTGCTGCAGCTAAC
GCATTAAGCACTCCGCC
>BSNB_1220_ Rhodococcus_cerastii

GGGGTACACGAGCGGCGAACCGGGGTGAGTAACACGTGGGTGGATCTCCCCTGCACTCTGG
GATAAGCTTGGGAAACTGGGTCTAATACCGGATATGACCACATCCCGCATGGTGTGTGGTGG
AAAGATTTATCGGTGCAGGATGGGCCCGCGGCCTATCAGCTTGTTGGTGGGGTAATGGCCTA
CCAAGGCGACGACGGGTAGCCGACCTGAGAGGGTGACCGGCCACACTGGGACTGAGACAC
GGCCCAGACTCCTACGGGAGGCAGCAGTGGGGAATATTGCACAATGGGCGGAAGCCTGAT
GCAGCGACGCCGCGTGAGGGATGAAGGCCTTCGGGTTGTAAACCTCTTTCAGCAGGGACGA
AGCGTGAGTGACGGTACCTGCAGAAGAAGCACCGGCTAACTACGTGCCAGCAGCCGCGGT
AATACGTAGGGTGCGAGCGTTGTCCGGAATTACTGGGCGTAAAGAGTTCGTAGGCGGTTTGT
CGCGTCGTTTGTGAAAACCCGGGGCTCAACTTCGGGCTTGCAGGCGATACGGGCAGACTTG
AGTGTTTCAGGGGAGACTGGAATTCCTGGTGTAGCGGTGAAATGCGCAGATATCAGGAGGA
ACACCGGTGGCGAAGGCGGGTCTCTGGGAAACAACTGACGCTGAGGAACGAAAGCGTGGG
TAGCAAACAGGATTAGATACCCTGGTAGTCCACGCCGTAAACGGTGGGCGCTAGGTGTGGG
TTCCTTCCACGGGATCTGTGCCGTAGCCTAACGCATTAAGCGCCC
>BSNB_1238_ Bacillus_subtilis

GGCTCAGGACGAACGCTGGCGGCGTGCCTAATACATGCAAGTCGAGCGGACAGATGGGAG
CTTGCTCCCTGATGTTAGCGGCGGACGGGTGAGTAACACGTGGGTAACCTGCCTGTAAGACT
GGGATAACTCCGGGAAACCGGGGCTAATACCGGATGGTTGTTTGAACCGCATGGTTCAAAC
ATAAAAGGTGGCTTCGGCTACCACTTACAGATGGACCCGCGGCGCATTAGCTAGTTGGTGA
GGTAACGGCTCACCAAGGCAACGATGCGTAGCCGACCTGAGAGGGTGATCGGCCACACTGG
GACTGAGACACGGCCCAGACTCCTACGGGAGGCAGCAGTAGGGAATCTTCCGCAATGGAC

GAAAGTCTGACGGAGCAACGCCGCGTGAGTGATGAAGGTTTTCGGATCGTAAAGCTCTGTT
GTTAGGGAAGAACAAGTACCGTTCGAATAGGGCGGTACCTTGACGGTACCTAACCAGAAAG
CCACGGCTAACTACGTGCCAGCAGCCGCGGTAATACGTAGGTGGCAAGCGTTGTCCGGAAT
TATTGGGCGTAAAGGGCTCGCAGGCGGTTTCTTAAGTCTGATGTGAAAGCCCCCGGCTCAAC
CGGGGAGGGTCATTGGAAACTGGGGAACTTGAGTGCAGAAGAGGAGAGTGGAATTCCACG
TGTAGCGGTGAAATGCGTAGAGATGTGGAGGAACACCAGTGGCGAAGGCGACTCTCTGGTC
TGTAACTGACGCTGAGGAGCGAAAGCGTGGGGAGCGAACAGGATTAGATACCCTGGTAGTC
CACGCCGTAAACGATGAGTGCTAAGTGTTAGGGGGTTTCCGCCCCTTAGTGCTGCAGCTAAC
GCATTAAGCACTCCGCC
>BSNB_1246_ Rhodococcus_cerastii

GGGGTACACGAGCGGCGAACCGGGGTGAGTAACACGTGGGTGGATCTCCCCTGCACTCTGG
GATAAGCTTGGGAAACTGGGTCTAATACCGGATATGACCACATCCCGCATGGTGTGTGGTGG
AAAGATTTATCGGTGCAGGATGGGCCCGCGGCCTATCAGCTTGTTGGTGGGGTAATGGCCTA
CCAAGGCGACGACGGGTAGCCGACCTGAGAGGGTGACCGGCCACACTGGGACTGAGACAC
GGCCCAGACTCCTACGGGAGGCAGCAGTGGGGAATATTGCACAATGGGCGGAAGCCTGAT
GCAGCGACGCCGCGTGAGGGATGAAGGCCTTCGGGTTGTAAACCTCTTTCAGCAGGGACGA
AGCGTGAGTGACGGTACCTGCAGAAGAAGCACCGGCTAACTACGTGCCAGCAGCCGCGGT
AATACGTAGGGTGCGAGCGTTGTCCGGAATTACTGGGCGTAAAGAGTTCGTAGGCGGTTTGT
CGCGTCGTTTGTGAAAACCCGGGGCTCAACTTCGGGCTTGCAGGCGATACGGGCAGACTTG
AGTGTTTCAGGGGAGACTGGAATTCCTGGTGTAGCGGTGAAATGCGCAGATATCAGGAGGA
ACACCGGTGGCGAAGGCGGGTCTCTGGGAAACAACTGACGCTGAGGAACGAAAGCGTGGG
TAGCAAACAGGATTAGATACCCTGGTAGTCCACGCCGTAAACGGTGGGCGCTAGGTGTGGG
TTCCTTCCACGGGATCTGTGCCGTAGCCTAACGCATTAAGCGCCC
>BSNB_A1F2A1_ Klebsiella_michiganensis
TTCATGGCTCAGATTGAACGCTGGCGGCAGGCCTAACACATGCAAGTCGAGCGGTAGCACG
GGGAGCTTGCTCCTGGGTGACGAGCGGCGGACGGGTGAGTAATGTCTGGGGATCTGCCTGA
TGGAGGGGGATAACTACTGGAAACGGTAGCTAATACCGCATAACGTCGCAAGACCAAAGA
GGGGGACCTTCGGGCCTCTTGCCATCAGATGAACCCAGATGGGATTAGCTAGTAGGTGGGG
TAATGGCTCACCTAGGCGACGATCCCTAGCTGGTCTGAGAGGATGACCAGCCACACTGGAA
CTGAGACACGGTCCAGACTCCTACGGGAGGCAGCAGTGGGGAATATTGCACAATGGGCGCA
AGCCTGATGCAGCCATGCCGCGTGTATGAAGAAGGCCTTCGGGTTGTAAAGTACTTTCAGCG
AGGAGGAAGGCGTTAAGGTTAATAACCTTGGCGATTGACGTTACTCGCAGAAGAAGCACCG
GCTAACTCCGTGCCAGCAGCCGCGGTAATACGGAGGGTGCAAGCGTTAATCGGAATTACTG
GGCGTAAAGCGCACGCAGGCGGTTTGTTAAGTCGGATGTGAAATCCCCGGGCTCAACCTGG
GAACTGCATTCGAAACTGGCAAGCTTGAGTCTTGTAGAGGGGGGTAGAATTCCAGGTGTAG
CGGTGAAATGCGTAGAGATCTGGAGGAATACCGGTGGCGAAGGCGGCCCCCTGGACAAAG
ACTGACGCTCAGGTGCGAAAGCGTGGGGAGCAAACAGGATTAGATACCCTGGTAGTCCACG
CCGTAAACGATGTCGACTTGGAGGTTGTGCCCTTGAGGCGTGGCTTCCGGAGCTAACGCGTT
AAGTCGACCGCCTG
>BSNB_A4F1_12_ Klebsiella_michiganensis

TTCATGGCTCAGATTGAACGCTGGCGGCAGGCCTAACACATGCAAGTCGAGCGGTAGCACG
GGGAGCTTGCTCCTGGGTGACGAGCGGCGGACGGGTGAGTAATGTCTGGGGATCTGCCTGA
TGGAGGGGGATAACTACTGGAAACGGTAGCTAATACCGCATAACGTCGCAAGACCAAAGA
GGGGGACCTTCGGGCCTCTTGCCATCAGATGAACCCAGATGGGATTAGCTAGTAGGTGGGG
TAATGGCTCACCTAGGCGACGATCCCTAGCTGGTCTGAGAGGATGACCAGCCACACTGGAA
CTGAGACACGGTCCAGACTCCTACGGGAGGCAGCAGTGGGGAATATTGCACAATGGGCGCA
AGCCTGATGCAGCCATGCCGCGTGTATGAAGAAGGCCTTCGGGTTGTAAAGTACTTTCAGCG
AGGAGGAAGGCGTTAAGGTTAATAACCTTGGCGATTGACGTTACTCGCAGAAGAAGCACCG
GCTAACTCCGTGCCAGCAGCCGCGGTAATACGGAGGGTGCAAGCGTTAATCGGAATTACTG

GGCGTAAAGCGCACGCAGGCGGTTTGTTAAGTCGGATGTGAAATCCCCGGGCTCAACCTGG
GAACTGCATTCGAAACTGGCAAGCTTGAGTCTTGTAGAGGGGGGTAGAATTCCAGGTGTAG
CGGTGAAATGCGTAGAGATCTGGAGGAATACCGGTGGCGAAGGCGGCCCCCTGGACAAAG
ACTGACGCTCAGGTGCGAAAGCGTGGGGAGCAAACAGGATTAGATACCCTGGTAGTCCACG
CCGTAAACGATGTCGACTTGGAGGTTGTGCCCTTGAGGCGTGGCTTCCGGAGCTAACGCGTT
AAGTCGACCGCCTG
>BSNB_LRFP_069_ Methylobacterium_mesophilicum

CCGGATACGCCCTTTTGGGGAAAGGTTTACTGCCGGAAGATCGGCCCGCGTCTGATTAGCTA
GTTGGTGGGGTAACGGCCTACCAAGGCGACGATCAGTAGCTGGTCTGAGAGGATGATCAGC
CACACTGGGACTGAGACACGGCCCAGACTCCTACGGGAGGCAGCAGTGGGGAATATTGGA
CAATGGGCGCAAGCCTGATCCAGCCATGCCGCGTGAGTGATGAAGGCCTTAGGGTTGTAAA
GCTCTTTTATCCGGGACGATAATGACGGTACCGGAGGAATAAGCCCCGGCTAACTTCGTGCC
AGCAGCCGCGGTAATACGAAGGGGGCTAGCGTTGCTCGGAATCACTGGGCGTAAAGGGCGC
GTAGGCGGCGTTTTAAGTCGGGGGTGAAAGCCTGTGGCTCAACCACAGAATGGCCTTCGAT
ACTGGGACGCTTGAGTATGGTAGAGGTTGGTGGAACTGCGAGTGTAGAGGTGAAATTCGTA
GATATTCGCAAGAACACCGGTGGCGAAGGCGGCCAACTGGACCATTACTGACGCTGAGGCG
CGAAAGCGTGGGGAGCAAACAGGATTAGATACCCTGGTAGTCCACGCCGTAAACGATGAAT
GCCAGCTGTTGGGGTGCTTGCACCGCAGTAGCGCAGCTAACGCTTTGAGCATCCGCC

Table S5. ITS of studied fungal strains (BSNB)

>BSNB_0001_ *Epicoccum_nigrum*

TGGGTATCCTACATGATCCGAGGTCAAGAGTGTAAAAATGTACTTTTGGACGTCGTCGTTAT
GAGTGCAAAGCGCGAGATGTACTGCGCTCCGAAATCAATACGCCGGCTGCCAATTGTTTTAA
GGCGAGTCTGCGCGCGGAGGCGAGACAAAACACCCAACACCAAGCAGAGCTTGAAGGTAC
AAATGACGCTCGAACAGGCATGCCCCATGGAATACCAAGGGGCGCAATGTGCGTTCAAAG
ATTCGATGATTCACTGAATTCTGCAATTCACACTACTTATCGCATTTCGCTGCGTTCTTCATCG
ATGCCAGAACCAAGAGATCCGTTGTTGAAAGTTGTAACTATTAAGTTTTTCAGACGCTGATT
GCAACTGCAAAGGGTTTGAATGTTGTCCAACCGGCGGGCGGACCCGCCGAGGAAACGAAG
GTACTCAAAAGACATGGGTAAGAGGTAGCAGACCGAAGTCTGCAAACTCTAGGTAATGATC
CTTCCGCAGGTTCACCCTACGGAAGGA

>BSNB_0002_Colletotrichum_gloeosporioides

TGGGTCTCCTACCTGATCCGAGGTCAACCTTTGGAAAATTGGGGGGTTTTACGGCAAGAGTC
CCTCCGGATCCCAGTGCGAGACGTAAAGTTACTACGCAAAGGAGGCTCCGGGAGGGTCCGC
CACTACCTTTGAGGGCCTACATCAGCTGTAGGGCCCCAACACCAAGCAGAGCTTGAGGGTT
GAAATGACGCTCGAACAGGCATGCCCGCCAGAATGCTGGCGGGCGCAATGTGCGTTCAAAG
ATTCGATGATTCACTGAATTCTGCAATTCACATTACTTATCGCATTTCGCTGCGTTCTTCATCG
ATGCCAGAACCAAGAGATCCGTTGTTAAAAGTTTTGATTATTTGCTTGTACCACTCAGAAGA
AACGTCGTTAAATCAGAGTTTGGTTATCCTCCGGCGGGCGCCGACCCGCCCGGGGGCGGGA
GGCCGGGAGGGTCACGGAGACCCTACCCGCCGAAGCAACAGTTATAGGTATGTTCACAAAG
GGTTATAGAGCGTAAACTCAGTAATGATCCCTCCGAAGGTTCACCCCTACGGAA

>BSNB_0006_Pestalotiopsis_sp

GTCGGGGATACTACCTGATCCGAGGTCAACCCAGTAAAAAAGTTGGGGGTTTAGCGGCGAG
GGCCACGGCACCTACAGAAGCGAGAGGTATATTACTGCGCTCAGAGGTAGAACCGTCACTC
CGCCGGTGAATTTGAGGAGCTACAGGGTAACAGCTACAGGGTAGCTCCCGGGTAGTCTCCC
AACGCCAAGACTAGGTCTTGAGTGGTACTAATGACGCTCGAACAGGCATGCCCTCCAGAAT
GCTGGGGGGCGCAATGTGCGTTCAAAGATTCGATGATTCACTGAATTCTGCAATTCACATTA
CTTATCGCATTTCGCTGCGTTCTTCATCGATGCCAGAACCAAGAGATCCGTTGTTAAAGGTTT
TGACTTATTTTCATAAGACTCTCAGATGATAATAATACAAATAGTTTGGTTTTCCACCGGCGG
TCCGCCCGCTACAGGGTAAGCTGCAGGGTAAGACCGCCGAGGCAACGTAAGGTATAGGTTC
ACAAATGGTTTATACAGGAGTGTTTTAATACTCTGTAATGATCCCTCCGCAGGTTCACCCTAC
AGAA

>BSNB_0008_Pestalotiopsis_sp

TGGGGTATCTACCTGATCCGAGGTCAACCCAGTAAAAAAGTTGGGGGTTTAGCGGCGAGGG
CCACGGCACCTACAGAAGCGAGAGGTATATTACTGCGCTCAGAGGTAGAACCGTCACTCCG
CCGGTGAATTTGAGGAGCTACAGGGTAACAGCTACAGGGTAGCTCCCGGGTAGTCTCCCAA
CGCCAAGACTAGGTCTTGAGTGGTACTAATGACGCTCGAACAGGCATGCCCTCCAGAATGC
TGGGGGGCGCAATGTGCGTTCAAAGATTCGATGATTCACTGAATTCTGCAATTCACATTACTT
ATCGCATTTCGCTGCGTTCTTCATCGATGCCAGAACCAAGAGATCCGTTGTTAAAGGTTTTGA
CTTATTTTCATAAGACTCTCAGATGATAATAATACAAATAGTTTGGTTTTCCACCGGCGGTCC
GCCCGCTACAGGGTAAGCTGCAGGGTAAGACCGCCGAGGCAACGTAAGGTATAGGTTCACA
AATGGTTTATACAGGAGTGTTTTAATACTCTGTAATGATCCCTCCGCAGGTTACCCCTACGGA
A

>BSNB_0018_Chaetomium_globosum

CTACCTGATCCGAGGTCAACCTTGGGTTAAAAGGTGGTTTAACGGCCGGAACCCGCAGCAC
GCCCAGAGCGAGATGTATGCTACTACGCTCGGTGTGACAGCGAGCCCGCCACTGCTTTTCAG
GGCCTGCGGCAGCCGCAGGTCCCCAACACAAGCCCGGGGGCTTGATGGTTGAAATGACGCT
CGAACAGGCATGCCCGCCAGAATACTGGCGGGCGCAATGTGCGTTCAAAGATTCGATGATT
CACTGAATTCTGCAATTCACATTACTTATCGCATTTCGCTGCGTTCTTCATCGATGCCAGAAC
CAAGAGATCCGTTGTTGAAAGTTTTGACTTATTCAGTACAGAAGACTCAGAGAGGCCATAA
ATTATCAAGAGTTTGGTGACCTCCGGCGGGCGCCCGCGGTGGGCCCAGGGGCGCCCGGGG
GGTAAACCCCGGGGCCGCCCGCCGAAGCAACGGTTTAGGTAACGTTCACAATGGTTTAGGG
AGTTTTGCAACTCTGTAATGATCCCTCCGCAGGTTCACCTACGGAA
>BSNB_0020_Diaporthe_phaseolorum

CGACTAGCTGCATGATCCGAGGTCAAATTTTACAGAAGTTGGGGGTTTAACGGCAGGGCAC
CGCCAGGGCCTTCCAGAACGAGATATAACTACTACGCTCGGGGTCCTAGCGAGCTCGCCAC
TAGATTTCAGGGCCTGCCCTCGTTAGAAGGCAGTGCCCCATCACCAAGCCAGGCTTGAGGGT
TGAAATGACGCTCGAACAGGCATGCCCTCCGGAATACCAGAGGGCGCAATGTGCGTTCAAA
GATTCGATGATTCACTGAATTCTGCAATTCACATTACTTATCGCATTTCGCTGCGTTCTTCATC
GATGCCAGAACCAAGAGATCCGTTGTTGAAAGTTTTGATTCATTTATGTTTTTACTCAGAGA
TTCACTAAAGAAACAAGAGTTTAGTTGGCCGCCGGCGGGCTGCTCCCTGTTTCCAGGGGGCC
TCAGTGAAGAGGCCGGCCTGCGCCGAGGCAACAAATAGGTATAAGTTCACAAAGGGTTTCT
GGGTGCGCCGAAGCGCGTTCCAGCAATGATCCCTCCGCAGGTTCCCCCTACGGAAG
>BSNB_0022_Xylaria_cubensis

NNNNNNNNNGGCAGAACCGATTMTGAGGTATGCCTTGCAGAAKTTGGGGTTTTACGGCAG
GGGTATCAGTCCAACTAATAGGCGAGATAAAAAATTACTACGTCTAGAGTGTGAACCAACT
CCGCCACTAACTTTGAGGAGCTACGTTACCGTAGGCTCCCAACACTAAGCAACTAAGGCTTA
AGGGTTGAAATGACGCTCGAACGGTCCTGCCCACTAGAATACTAATGGGCGCAATATGCGT
TCAAAGATTCGATGATTCACTGAATTCTGCAATTCACATTACTTATCGCATTTCGCTGCGTTCT
TCATCGATGCCAGAACCAAGAGATCCGTTGTTGAAAGTTTTAACTTATTTAGTTATAGGTTCA
GAATTCAATATCAAACAGAGTTTGGTGGGCCGCCGGCAGGCTTACCCGCACCTCCCGGGTA
AGTCTTACAGGGTAAGTCTACGAGGTAGGCGCGACCTGCCGAGGCAACGTTAGGTATGTTC
ACATGGGTTTGGGAGTTATAGAACTCTTTAATGATCCCTCCGCAGGTTCACCTACGGAGGGA
>BSNB_0024_Mycoleptodiscus_sp

TAATCAAACTGCATAATTCGAGGTCGACGTGAGAAAAAATGAAGTTTCGTTTGTCCGGCTGG
CTTCGGGCTGCTCAAAGCTGCATTGCTGCTGCGCTCCAGGCTGCACCGGCACCGCCGATGGA
TTCAAGGGCCGCCTCCGTATTTCAGGGGCGGTCCCCAACACTAAGCTTGCGCTTAAGGGTCG
AAACGACGCTCGAATAGGCATGCCTCGAGGAATACCAAGAGGCGCAATGTGCGTTCAAAG
ACTCGATGATTCACTGAAATCTGCAATTCACACTAGTTATCGCATTTCGCTGCGTTCTTCATC
GATGCTGGAGCCAAGAGATCCATTGTTGAAAGTTGTATTTATCGCATTCGCGAAGGTAAAAT
CAGACGTTCTTCGAACAGAATGGTTTTGTAGCGTCCTGCGGCGGGCTGCTCCTCCCGAGGGA
GGGGCGCCAGTCGAGCCAGTGCCGGCCCGCCGAGGCAACGAAACGTTTGGCAAACAGTGG
TTTGAGAATCGGGGCCACGAGGGCACCCTTTTCCTGTTAATGATCCTTCCGCAGGTTCACCTA
CGGAAG
>BSNB_0025_Colletotrichum_siamense

GGTGTATGCTACCTGATCCGAGGTCAACCTTTGGAAAATTGGGGGGTTTACGGCAAGAGTC
CCTCCGGATCCCAGTGCGAGACGTAAAGTTACTACGCAAAGGAGGCTCCGGGAGGGTCCGC
CACTACCTTTGAGGGCCTACATCAGCTGTAGGGCCCCAACACCAAGCAGAGCTTGAGGGTT
GAAATGACGCTCGAACAGGCATGCCCGCCAGAATGCTGGCGGGCGCAATGTGCGTTCAAAG
ATTCGATGATTCACTGAATTCTGCAATTCACATTACTTATCGCATTTCGCTGCGTTCTTCATCG
ATGCCAGAACCAAGAGATCCGTTGTTAAAAGTTTTGATTATTTGCTTGTACCACTCAGAAGA

AACGTCGTTAAATCAGAGTTTGGTTATCCTCCGGCGGGCGCCGACCCGCCCGGGGGCGGGA
GGCCGGGAGGGTCACGGAGACCCTACCCGCCGAAGCAACAGTTATAGGTATGTTCACAAAG
GGTTATAGAGCGTAAACTCAGTAGTGATCCCTCCAAAGGTTCACCTACGGAA
>BSNB_0026_Mycoleptodiscus_sp

TTAATAACGGATACTGCATAATTCGAGGTCGACGTGAGAAAAAATGAAGTTTCGTTTGTCCG
GCTGGCTTCGGGCTGCTCAAAGCTGCATTGCTGCTGCGCTCCAGGCTGCACCGGCACCGCCG
ATGGATTCAAGGGCCGCCTCCGTATTTCAGGGGCGGTCCCCAACACTAAGCTTGCGCTTAAG
GGTCGAAACGACGCTCGAATAGGCATGCCTCGAGGAATACCAAGAGGCGCAATGTGCGTTC
AAAGACTCGATGATTCACTGAAATCTGCAATTCACACTAGTTATCGCATTTCGCTGCGTTCTT
CATCGATGCTGGAGCCAAGAGATCCATTGTTGAAAGTTGTATTTATCGCATTCGCGAAGGTA
AAATCAGACGTTCTTCGAACAGAATGGTTTTGTAGCGTCCTGCGGCGGGCTGCTCCTCCCGA
GGGAGGGGCGCCAGTCGAGCCAGTGCCGGCCCGCCGAGGCAACGAAACGTTTGGTAAACA
GTGGTTTGAGAATCGGGGCCACGAGGGCACCCTTTTCCTGTTAATGATCCTTCCGCAGGTTC
ACCCTACGGAAG
>BSNB_0029_Colletotrichum_gloeosporioides

GGGTATCCTACCTGATCCGAGGTCAACCTTTGGAAAATTGGGGGGTTTTACGGCAAGAGTCC
CTCCGGATCCCAGTGCGAGACGTAAAGTTACTACGCAAAGGAGGCTCCGGGAGGGTCCGCC
ACTACCTTTGAGGGCCTACATCAGCTGTAGGGCCCCAACACCAAGCAGAGCTTGAGGGTTG
AAATGACGCTCGAACAGGCATGCCCGCCAGAATGCTGGCGGGCGCAATGTGCGTTCAAAGA
TTCGATGATTCACTGAATTCTGCAATTCACATTACTTATCGCATTTCGCTGCGTTCTTCATCGA
TGCCAGAACCAAGAGATCCGTTGTTAAAAGTTTTGATTATTTGCTTGTACCACTCAGAAGAA
ACGTCGTTAAATCAGAGTTTGGTTATCCTCCGGCGGGCGCCGACCCGCCCGGAGGCGGGAG
GCCGGGAGGGTCGCGGAGACCCTACCCGCCGAAGCAACAGTTATAGGTATGTTCACAAAGG
GTTATAGAGCGTAAACTCAGTAATGATCCCTCCGCAGGTTCCCCCTACGGAAG
>BSNB_0032_Colletotrichum_boninense

TGGGTATGCTACCTGATCCGAGGTCAACCTTTGGAAAATTGGGGGGTTTTACGGCAAGAGTC
CCTCCGGATCCCAGTGCGAGACGAAATGTTACTACGCAAAGGAGGCTCCGGGAGGGTCCGC
CACTACCTTTGAGGGCCTACGTCGACCGTAGAGCCCCAACACCAAGCAGAGCTTGAGGGTT
GAAATGACGCTCGAACAGGCATGCCCGCCAGAATGCTGGCGGGCGCAATGTGCGTTCAAAG
ATTCGATGATTCACTGAATTCTGCAATTCACATTACTTATCGCATTTCGCTGCGTTCTTCATCG
ATGCCAGAACCAAGAGATCCGTTGTTAAAAGTTTTGATTATTTTGCTTATGCCACTCAGAAG
AAACGTCGTTACAATAGAGTTTGGTTATCCTCCGGCGGGCGCCGGGTCCGGTCCCGCGGGGG
GTCCGGTCCGGGCCGGGAGGCGTCCTTTTCAGGGGACGGCCTACCCGCCGAAGCAACAGTT
GTAGGTATGTTCACAAAGGGTTATAGAGCGGTAACTCAGTAATGATCCCTCCACAGGTTACC
CCTACGGAAG
>BSNB_0033_Diaporthe_phaseolorum

CTACCTGATCCGAGGTCAAATTTTCAGAAGTTGGGGGTTTAACGGCAGGGCACCGCCAGGG
CCTTCCAGAACGAGATATAACTACTACGCTCGGGGTCCTAGCGAGCTCGCCACTAGATTTCA
GGGCCTGCCCTCGTTAGAAGGCAGTGCCCCATCACCAAGCCAGGCTTGAGGGTTGAAATGA
CGCTCGAACAGGCATGCCCTCCGGAATACCAGAGGGCGCAATGTGCGTTCAAAGATTCGAT
GATTCACTGAATTCTGCAATTCACATTACTTATCGCATTTCGCTGCGTTCTTCATCGATGCCA
GAACCAAGAGATCCGTTGTTGAAAGTTTTGATTCATTTATGTTTTTTACTCAGAGATTCACTA
AAGAAACAAGAGTTTAGTTGGCCGCCGGCGGGCTGCTCCCTGTTTCCAGGGGGCCTCAGTG
AAGAGGCCGGCCTGCGCCGAGGCAACAAATAGGTATAAGTTCACAAAGGGTTTCTGGGTGC
GCCGAAGCGCGTTCCAGCAATGATCCCTCCGCAGGTTCACCCTACGGAA
>BSNB_0035_Colletotrichum_boninense

TTAGCTAGCTTCGNTTATTGGTCTGCTCCTGGAAAATTGGGGGGTTTACGGCAAGAGTCCCT
CCGGATCCCAGTGCGAGACGAAATGTTACTACGCAAAGGAGGCTCCGGGAGGGTCCGCCAC
TACCTTTGAGGGCCTACGTCGACCGTAGAGCCCCAACACCAAGCAGAGCTTGAGGGTTGAA
ATGACGCTCGAACAGGCATGCCCGCCAGAATGCTGGCGGGCGCAATGTGCGTTCAAAGATT
CGATGATTCACTGAATTCTGCAATTCACATTACTTATCGCATTTCGCTGCGTTCTTCATCGATG
CCAGAACCAAGAGATCCGTTGTTAAAAGTTTTGATTATTTTGCTTATGCCACTCAGAAGAAA
CGTCGTTACAATAGAGTTTGGTTATCCTCCGGCGGGCGCCGGGTCCGGTCCCGCGGGGGGTC
CGGTCCGGGCCGGGAGGCGTCCTTTTCAGGGGACGGCCTACCCGCCGCAGCAACAGTTGTA
GGTATGTTCACAAAGGGTTATAGAGCGGTAACTCAGTAATGATCCCTCCGCAGGTTCACCCT
ACGGA
>BSNB_0038_Xylaria_cubensis

NNNNNNNNNNNGGAACCTTCGATTATTGTCTGCTCCTCCGCGTTGTGGTTTACCGCCCGGT
TATCCGACCCACCCCTAAGCGAGAGATACGTTACTACGYYTAGGRCGTGAAACCCCTCCGCC
ACTAACTTTGAGGAGCTACTTTGGCGTAAGCTCCCGCCACTAGAGGCTAAAGCTTAAGGGG
ACAAATGACCCTGGATGCGCCCTGCCCACTAAAGTACTAATGGGCGCAATATGCGATCAAA
GATTCGAYYATTCTCTGAATTCYGCATTTCTCATTACTTATCGCTTTTCTCTGCGTTCTTCATCG
ATCCCAGAACCCAGATATCCGTTGTTGAAAGTTTTAACTTATTTGTTATAGGTTCAAAATTC
AATATCAAACATARTTTGGTGGGCCGCCGGCCGGCTTACCCGCACCTCCCGGGTAAGTCTTA
CCGGGTAAGACTACCAGGTAGGCGCGACCTGCCGAGGCAACGTTAGGTATGTTCACGTGGG
TTTGGGAGTTATAGAACTCTTTAATGATCCCTCCGCAGGTTCACCTACGGAAGG
>BSNB_0039_Colletotrichum_gloeosporioides

TCTACCTGATCCGAGGTCAACCTTTGGAAAATTGGGGGGTTTACGGCAAGAGTCCCTCCGG
ATCCCAGTGCGAGACGAAATGTTACTACGCAAAGGAGGCTCCGGGAGGGTCCGCCACTACC
TTTGAGGGCCTACGTCGACCGTAGAGCCCCAACACCAAGCAGAGCTTGAGGGTTGAAATGA
CGCTCGAACAGGCATGCCCGCCAGAATGCTGGCGGGCGCAATGTGCGTTCAAAGATTCGAT
GATTCACTGAATTCTGCAATTCACATTACTTATCGCATTTCGCTGCGTTCTTCATCGATGCCA
GAACCAAGAGATCCGTTGTTAAAAGTTTTGATTATTTTGCTTATGCCACTCAGAAGAAACGT
CGTTACAATAGAGTTTGGTTATCCTCCGGCGGGCGCCGGGTCCGGTCCCGCGGGGGGTCCGG
TCCGGGCCGGGAGGCGTCCTTTTCAGGGGACGGCCTACCCGCCGAAGCAACAGTTGTAGGT
ATGTTCACAAAGGGTTATAGAGCGGTAACTCAGTAATGATCCCTCCGCAGGTTCACCTACGG
AA
>BSNB_0041_Xylaria_sp

GGGGTATCCTACCTAATCCGAGGTCAACCACTAGAAAATATAGGGGTTTTAACGGCTAGCA
GCCAGGGCCACCACACGAGCGAGAGAAATTACTACGCTGAGAGTGTACCCTAACTCCGCAC
TAACTTTGAGGAACTACGCCGTAGATTCCCAACGCTAAGCAACAGGGGCTTAGGGGTCGAA
ATGACGCTCGAATAGGCATGCCCACTAGAATACTAGTGGGCGCAATGTGCGTTCAAAGATT
CGATGATTCACTGAATTCTGCAATTCACATTACTTATCGCATTTCGCTGCGTTCTTCATCGATG
CCAGAACCAAGAGATCCGTTGTTGAAAGTTTTAACTTATTTTGGTTTAAAATCAGAGAAACA
GTGGTAAAAACAAGAGTTTAACGGTCCTTCGGCGGGCCGAAGCCGACTACAGGGTAGCTCC
AGGGTAGCTCCAGGGTAGCTATAGGGTAGCTGCAGGGTAAGTGCAGGGTAGCTATAGGGTA
GCTATAGGGTAACTCTAGGGTAGCTCCAGGGTAGTTACAGGGTAGCCGTAGCTCACGCCGA
GGCAACGATGGTAAGGTTCACAAAGGGTTTGGAGTTTTGATAACTCAGTAATGATCCCTCCG
CAGATTACCCCTTACGGGAA
>BSNB_0042_Guignardia_mangiferae

AAGGCCTGCTACATGATCCGAGGTCAACCTTGGAAAAATAGACCGAAGGTCGATTGTCCGG
CGGCCGTCGCCCAGCACTCCAAAGCGAGATATTTTACTACTACGCTCGAGGCTAGGACGCC
GTCGCCGAGGTCTTCAAGGCACGTCCGGCAGCGGACGTTGCCCAATACCAAGCAGAGCTTG

AGGGTTGAAATGACGCTCGAACAGGCATGCCCTCCGGAATACCAGAGGGCGCAATGTGCGT
TCAAAGATTCGATGATTCACTGAATTCTGCAATTCACATTACTTATCGCATTTCGCTGCGTTCT
TCATCGATGCCAGAACCAAGAGATCCGTTGTTGAAAGTTTTAATCAATTAAATGATATATCA
GGACTTCACAAAATGAATTCTTGAGTTTTGTATACTGGCGGGCACTTAGCCGGGCGTCCTGG
CCAGTTAAGGCTGGGGGCGCCGGCCGCCTGGGTCGGAACCAGGTCGACCCGCCAAAGCAA
CATAGTGAGTACACAAGGGTGAGAAGGTCATTTCGGCGTTGTAGCGCCTACTCTGGAACCTT
TCAATAGAAGTTATTACATTTCAGTAATGATCCTTCCGCAGGTTCACCCTACGGAAGG
>BSNB_0043_Pestalotiopsis_sp

GGGGTATCCTACCTGATCCGAGGTCAACCCAGTAAAAAAGTTGGGGGTTTAGCGGCGAGGG
CCACGGCACCTACAGAAGCGAGAGGTATATTACTGCGCTCAGAGGTAGAACCGTCACTCCG
CCGGTGAATTTGAGGAGCTACAGGGTAACAGCTACAGGGTAGCTCCCGGGTAGTCTCCCAA
CGCCAAGACTAGGTCTTGAGTGGTACTAATGACGCTCGAACAGGCATGCCCTCCAGAATGC
TGGGGGGCGCAATGTGCGTTCAAAGATTCGATGATTCACTGAATTCTGCAATTCACATTACTT
ATCGCATTTCGCTGCGTTCTTCATCGATGCCAGAACCAAGAGATCCGTTGTTAAAGGTTTTGA
CTTATTTTCATAAGACTCTCAGATGATAATAATACAAATAGTTTGGCTTTCCACCGGCGGTCC
GCCCGCTACAGGGTAAGCTGCAGGGTAAGACCGCCGAGGCAACGTAAGGTATAGGTTCACA
AATGGTTTATACAGGAGTGTTTTAATACTCTGTAATGATCCCTCCGCAGGTTCCCCCTACAGA
A
>BSNB_0044_Diaporthe_sp

GGGTATGCTACATGATCCGAGGTCAAAATTTCAGAAGTTGGGGGTTTAACGGCAGGGCACC
GCCAGGGCCTTCCAGAACGAGATATAACTACTACGCTCGGGGTCCTAGCGAGCTCGCCACT
AGATTTCAGGGCCTGCTTCTCTCGAAGCAGTGCCCCAACACCAAGCCAGGCTTGAGGGTTGA
AATGACGCTCGAACAGGCATGCCCTCCGGAATACCAGAGGGCGCAATGTGCGTTCAAAGAT
TCGATGATTCACTGAATTCTGCAATTCACATTACTTATCGCATTTCGCTGCGTTCTTCATCGAT
GCCAGAACCAAGAGATCCGTTGTTGAAAGTTTTGATTCATTTATGTTTTTTACTCAGAGATTC
ACTAAGAAACAAGAGTTTGGTTGGCCGCCGGCGGGCTGCTCCCCGTCTCCGGGGGGCCTCA
GAAGAGGCCGGCCTTCGCCGAGGCAACAATAGGTATAAGTTCACAAAGGGTTTCTGGGTGC
GCCGAGGCGCGTTCCAGCAATGATCCCTCCGCAGGTTCCCCCTACGGAA
>BSNB_0047_Diaporthe_sp

GGGTATGCTACCTGATCCGAGGTCAAAATTTCAGAAGTTGGGGGTTTAACGGCAGGGCACC
GCCAGGGCCTTCCAGAACGAGATATAACTACTACGCTCGGGGTCCTAGCGAGCTCGCCACT
AGATTTCAGGGCCTGCTTCTCTCGAAGCAGTGCCCCAACACCAAGCCAGGCTTGAGGGTTGA
AATGACGCTCGAACAGGCATGCCCTCCGGAATACCAGAGGGCGCAATGTGCGTTCAAAGAT
TCGATGATTCACTGAATTCTGCAATTCACATTACTTATCGCATTTCGCTGCGTTCTTCATCGAT
GCCAGAACCAAGAGATCCGTTGTTGAAAGTTTTGATTCATTTATGTTTTTTACTCAGAGATTC
ACTAAGAAACAAGAGTTTGGTTGGCCGCCGGCGGGCTGCTCCCCGTCTCCGGGGGGCCTCA
GAAGAGGCCGGCCTTCGCCGAGGCAACAATAGGTATAAGTTCACAAAGGGTTTCTGGGTGC
GCCGAGGCGCGTTCCAGCAATGATCCCTCCGCAGGTTCCCCCTACGGAA
>BSNB_0048_Xylariales_sp

CGCACTGCTACCTAATCCGAGGTCAACCACTAGAAAATATAGGGGTTTTAACGGCTAGCAG
CCAGGGCCACCACACGAGCGAGAGAAATTACTACGCTGAGAGTGTACCCTAACTCCGCCAC
TAACTTTGAGGAACTACGCCGTAGATTCCCAACGCTAAGCAACAGGGGCTTAGGGGTCGAA
ATGACGCTCGAATAGGCATGCCCACTAGAATACTAGTGGGCGCAATGTGCGTTCAAAGATT
CGATGATTCACTGAATTCTGCAATTCACATTACTTATCGCATTTCGCTGCGTTCTTCATCGATG
CCAGAACCAAGAGATCCGTTGTTGAAAGTTTTAACTTATTTTGGTTTAAAATCAGAGAAACA
GTGGTAAAAACAAGAGTTTAACGGTCCTTCGGCGGGCCGAAGCCGACTACAGGGTAGCTCC
AGGGTAGCTCCAGGGTAGCTATAGGGTAGCTGCAGGGTAAGTGCAGGGTAGCTATAGGGTA

GCTATAGGGTAACTCTAGGGTAGCTCCAGGGTAGTTACAGGGTAGCCGTAGCTCACGCCGA
GGCAACGATGGTAAGGTTCACAAAGGGTTTGGAGTTTTGATAACTCAGTAATGATCCCTCCG
CAGGTTACCCCTAACGGAA
>BSNB_0050_Xylariales_sp

TAACTCCTGCTGCCTAATCCGAGGTCAACCACTAGAAAATATAGGGGTTTTAACGGCTAGCA
GCCAGGGCCACCACACGAGCGAGAGAAATTACTACGCTGAGAGTGTACCCTAACTCCGCCA
CTAACTTTGAGGAACTACGCCGTAGATTCCCAACGCTAAGCAACAGGGGCTTAGGGGTCGA
AATGACGCTCGAATAGGCATGCCCACTAGAATACTAGTGGGCGCAATGTGCGTTCAAAGAT
TCGATGATTCACTGAATTCTGCAATTCACATTACTTATCGCATTTCGCTGCGTTCTTCATCGAT
GCCAGAACCAAGAGATCCGTTGTTGAAAGTTTTAACTTATTTGGTTTAAAATCAGAGAAAC
AGTGGTAAAAACAAGAGTTTAACGGTCCTTCGGCGGGCCGAAGCCGACTACAGGGTAGCTC
CAGGGTAGCTCCAGGGTAGCTATAGGGTAGCTGCAGGGTAAGTGCAGGGTAGCTATAGGGT
AGCTATAGGGTAACTCTAGGGTAGCTCCAGGGTAGTTACAGGGTAGCCGTAGCTCACGCCG
AGGCAACGATGGTAAGGTTCACAAAGGGTTTGGAGTTTTGATAACTCAGTAATGATCCCTCC
GCAGGTTCACCCCTACGGAAG
>BSNB_0051_Penicillium_sp

NNNNNNNNNACCTACCTGATCCGAGGTCAACCGAAAAGGACGACAGCCCCGAAGGACCG
CCGAGAAGGAAGACCAGCGCCGACCGAGTCCCTCCCGAGCGGGTGACAAAGCCCCATACG
CTCGAGGACCCGACGCGGCGCCGCCACTGCCTTTGGGGCGTGTTCCCGGGGGAACAGCGCC
CAACACCCAGCCGTGCTGGAGGGCAGAAATGACGCTCGGACAGGCATGCCCTCCGGAATGC
CAGAGGGCGCAATGTGCGTTCAAAGATTCGATGATTCACGGAATTCTGCAATTCACATTACT
TATCGCATTTCGCTGCGTTCTTCATCGATGCTGGAACCAAGAGATCCGTTGTTGAAAGTTTTA
ATGATTCAATTCCACTCAGACTCACTATTCAGACAGGGTTCTCGGGCGCTTCGGCGGGCACG
GGCCCGGGGGCAGATGCCCCCGGCGACCGGGGCCAGGCCCCAGTGGGCCCGCCGAGGCA
ACGCGGTAACAGTAAACACGGGTGGGAGGTTGGGCTCGTTGGAACCCGCACTCGGTAATGA
TCCTTCCGCAGGTTCACCTACGGAAGG
>BSNB_0052_Phomopsis_sp

CCCGCATGCTACCTGATCCGAGGTCAAATTTTCAAAAGTTGGGGGTTTAACGGCAGGGCACC
GCCAGGGCCTTCCAGAGCGAGGGTTTAACTACTGCGCTCGGGGTCCTGGCGAGCTCGCCACT
AGATTTCAGGGCCTGCTTCGTTAAAAGCAGTGCCCCAACACCAAGCAATGCTTGAGGGTTGA
AATGACGCTCGAACAGGCATGCCCTCCGGAATACCAGAGGGCGCAATGTGCGTTCAAAGAT
TCGATGATTCACTGAATTCTGCAATTCACATTACTTATCGCATTTCGCTGCGTTCTTCATCGAT
GCCAGAACCAAGAGATCCGTTGTTGAAAGTTTTGATTCATTTGTGTTTTTTCTCAGAGTTTCA
GTGTAAAAACAGAGTTGACTTGGCCGCCGGCGTGCCGTCTCCTCACCGAAGTGAGGGGCCT
ACTAGAGACCAGCATGCGCCGAGGCAACAAAAGGTATAAGTTCACAAAGGGTTTCTGGGTG
CGCCTGGGGCGCGTTCCAGCAATGATCCCACCGCAGGTTACCCCTACGGGAA
>BSNB_0055_Phomopsis_phyllanthicola

CTCGCGGCTGCTACCTGATCCGAGGTCAAATTTTCAAAAGTTGGGGGTTTAACGGCAGGGCA
CCGCCAGGGCCTTCCAGAGCGAGGGTTTAACTACTGCGCTCGGGGTCCTGGCGAGCTCGCCA
CTAGATTTCAGGGCCTGCTTCGTTAGAAGCAGTGCCCCAACACCAAGCAATGCTTGAGGGTT
GAAATGACGCTCGAACAGGCATGCCCTCCGGAATACCAGAGGGCGCAATGTGCGTTCAAAG
ATTCGATGATTCACTGAATTCTGCAATTCACATTACTTATCGCATTTCGCTGCGTTCTTCATCG
ATGCCAGAACCAAGAGATCCGTTGTTGAAAGTTTTGATTCATTTGTGTTTTTCTCAGAGTTTC
AGTGTAAAAACAAGAGTTAACTTGGCCGCCGGCGTGCCTGCTCCCTGTCTCCAGGGGACCCC
ATAGGGGCCAGCATGCGCCGAGGCAACAAAAGGTATAAGTTCACAAAGGGTTTCTGGGTGC
GCCTGGGGCGCGTTCCAGCAATGATCCCACCGCAGGTTCACCCTACGGAA

>BSNB_0056_Xylaria_cubensis

NNNNNGCGGAGGACTACCAATAAGCGGAGGAGCATATCTAATAAGCGGAGGAGCATGTCR
CGCCTMSGAGGAGCTTCTACCCTGGAGGAGGTACCCGGGAGGTGCGGATARRCCTGCCGGC
GGCCCACCAAACTCTGTTTGATATTGAATTCTGAACCTATAACTAAATAAGTTAAAACTTTC
AACAACGGATCTCTTGGTTCTGGCATCGATGAAGAACGCAGCGAAATGCGATAAGTAATGT
GAATTGCAGAATTCAGTGAATCATCGAATCTTTGAACGCATATTGCGCCCATTAGTATTCTA
GTGGGCAGGACCGTTCGAGCGTCATTTCGACCCTTAAGCCTTAGTTGCTTAGTGTTGGGAGA
CTACGGCAACGTAGCTCCTCAAAGTTAGTGGCGGAGTTGGTTCACACTCTAGACGTAGTAAT
TTTTTATCTCGCCTATTAGTTGGACTGATCCCCTGCCGTAAAACCCCAACTTCTCAAGGTTGA
CCTCGAATCGGTTCAGACAAACTCGCTAAATTGAAGCATATCAATAAGCGGAGGAA
>BSNB_0057_Phomopsis_sp

CGAGTGACGCATGCTAGCATCGATCCATAGGTCAAATTTTCAAAAGTTGGGGGTTTAACGGC
AGGGCACCGCCAGGGCCTTCCAAAGCGAGGGTTTAACTACTGCGCTCGGGGTCCTGGCGAG
CTCGCCACTAGATTTCAGGGCCTGCCCTTTTACAGGCAGTGCCCCAACACCAAGCAATGCTT
GAGGGTTGAAATGACGCTCGAACAGGCATGCCCTCCGGAATACCAGAGGGCGCAATGTGCG
TTCAAAGATTCGATGATTCACTGAATTCTGCAATTCACATTACTTATCGCATTTCGCTGCGTTC
TTCATCGATGCCAGAACCAAGAGATCCGTTGTTGAAAGTTTTGATTCATTTGTGTTTTTTCTCA
GAGTTTCAGTGTAAAAACAAGAGTTAACTTGGCCGCCGGCGTGCCTGCTCCTTGTCTCCAAG
GGACCCCGAGGGGGCCAGCATGCGCCGAGGCAACAGTAAGGTATAAGTTCACAAAGGGTT
TCTGGGTGCGCCTGGGGCGCGTTCCAGCAATGATCCCTCCGCAGGTTCACCCTACGGA
>BSNB_0059_Diaporthe_phaseolorum

NNNNNNCTGGGACGCGCCCCAGGCGCACCCAGAAACCCTTTGTGAACTTATACCTTTTGTT
GCCTCGGCGCATGCTGGCCTCTAGTAGGCCCCTCACCCCGGTGAGGAGAAGGCACGCCGGC
GGCCAAGTTAACTCTTGTTTTTACACTGAAACTCTGAGAAAAAACACAAATGAATCAAAACT
TTCAACAACGGATCTCTTGGTTCTGGCATCGATGAAGAACGCAGCGAAATGCGATAAGTAA
TGTGAATTGCAGAATTCAGTGAATCATCGAATCTTTGAACGCACATTGCGCCCTCTGGTATTC
CGGAGGGCATGCCTGTTCGAGCGTCATTTCAACCCTCAAGCATTGCTTGGTGTTGGGGCACT
GCTTCTAACGAAGCAGGCCCTGAAATCTAGTGGCGAGCTCGCCAGGACCCCGAGCGTAGTA
GTTAAACCCTCGCTCTGGAAGGCCCTGGCGGTGCCCTGCCGTTAAACCCCCAACTTTTGAAA
ATTTGACCTCGGATCAGGTAGGAATACCCGCTGAACTTAAGCATATCAATAAGCGGAGGAA
>BSNB_0060_Diaporthe_phaseolorum

GAATGCTACCTGATCCGAGGTCAAATTTTCAAAAGTTGGGGGTTTAACGGCAGGGCACCGC
CAGGGCCTTCCAGAGCGAGGGTTTAACTACTACGCTCGGGGTCCTGGCGAGCTCGCCACTAG
ATTTCAGGGCCTGCTTCGTTAGAAGCAGTGCCCCAACACCAAGCAATGCTTGAGGGTTGAAA
TGACGCTCGAACAGGCATGCCCTCCGGAATACCAGAGGGCGCAATGTGCGTTCAAAGATTC
GATGATTCACTGAATTCTGCAATTCACATTACTTATCGCATTTCGCTGCGTTCTTCATCGATGC
CAGAACCAAGAGATCCGTTGTTGAAAGTTTTGATTCATTTGTGTTTTTTCTCAGAGTTTCAGT
GTAAAAACAAGAGTTAACTTGGCCGCCGGCGTGCCTTCTCCTCACCGGGGTGAGGGGCCTA
CTAGAGGCCAGCATGCGCCGAGGCAACAAAAGGTATAAGTTCACAAAGGGTTTCTGGGTGC
GCCTGGGGCGCGTTCCAGCAATGATCCCTCCGCAGGTTCCCCCTACGGAA
>BSNB_0063_Phomopsis_sp

CTGTGCATGCTACCTGATCCGAGGTCAAATTTTCAAAAGTTGGGGGTTTAACGGCAGGGCAC
CGCCAGGGCCTTCCAAAGCGAGGGTTTAACTACTGCGCTCGGGGTCCTGGCGAGCTCGCCA
CTAGATTTCAGGGCCTGCCCTTTTACAGGCAGTGCCCCAACACCAAGCAATGCTTGAGGGTT
GAAATGACGCTCGAACAGGCATGCCCTCCGGAATACCAGAGGGCGCAATGTGCGTTCAAAG
ATTCGATGATTCACTGAATTCTGCAATTCACATTACTTATCGCATTTCGCTGCGTTCTTCATCG

ATGCCAGAACCAAGAGATCCGTTGTTGAAAGTTTTGATTCATTTGTGTTTTTTCTCAGAGTTTC
AGTGTAAAAACAAGAGTTAACTTGGCCGCCGGCGTGCCTGCTCCTTGTCTCCAAGGACCCC
GAGGGGGCCAGCATGCGCCGAGGCAACAGTAAGGTATAAGTTCACAAAGGGTTTCTGGGTG
CGCCTGGGGCGCGTTCCAGCAATGATCCCTCCGCAGGTTCACCCTACGGAA
>BSNB_0071_Phomopsis_sp

CTACCTGATCCGAGGTCAAATTTTCAAAAGTTGGGGGTTTAACGGCAGGGCACCGCCAGGG
CCTTCCAAAGCGAGGGTTTAACTACTGCGCTCGGGGTCCTGGCGAGCTCGCCACTAGATTTC
AGGGCCTGCCCTTTTACAGGCAGTGCCCCAACACCAAGCAATGCTTGAGGGTTGAAATGAC
GCTCGAACAGGCATGCCCTCCGGAATACCAGAGGGCGCAATGTGCGTTCAAAGATTCGATG
ATTCACTGAATTCTGCAATTCACATTACTTATCGCATTTCGCTGCGTTCTTCATCGATGCCAG
AACCAAGAGATCCGTTGTTGAAAGTTTTGATTCATTTGTGTTTTTTCTCAGAGTTTCAGTGTAA
AAACAAGAGTTAACTTGGCCGCCGGCGTGCCTGCTCCTTGTCTCCAAGGACCCCGAGGGG
GCCAGCATGCGCCGAGGCAACAGTAAGGTATAAGTTCACAAAGGGTTTCTGGGTGCGCCTG
GGGCGCGTTCCAGCAATGATCCCTCCGCAGGTTCACCTACGGAA
>BSNB_0074_Chaetomium_sp

TATGCTACATGATCCGAGGTCAACCTTAAGATAAAGGGGTTTTACGGCCGGCACGCGCCGG
GACGCCGCGAGCGAGGTGTATTACTGCGCTACGGGTCCAGGCGCGCCCGCCACTTCTTTTCA
GGGCCCGCGGCGGCCGCGGGGCCCCAACACCGAGCAGGGCTCGAGGGTTGAAATGACGCT
CGAACAGGCATGCCCGCCGGAATGCCGGCGGGCGCAATGTGCGTTCAAAGATTCGATGATT
CGCTGAGTTCTGCAATTCACATTACTTATCGCATTTCGCTGCGTTCTTCATCGATGCCAGAAC
CAAGAGATCCGTTGTTGAAAGTTTTGACTCATTTAGTGTCTTCTCAGAGGGGCCTAAATCGCA
AGAGTTGTGCTGCGCTGCCGCCGGCGGGCGCCCTTGCGGGCTGCGGCCCCTCCCCGGGGCCG
CCCGCCGAAGCAACGAACAGTTGAAGAAACGTTCACGGGTGTATTAAGGAGAATGGAACTC
GTTAATGATCCCTCCGCAGGTTCACCTACGGAAGCC
>BSNB_0075_Xylaria_sp

GTATCCTACCTGATCCGAGGTCAACCATAGAAAAAGTTTGGGGCTTTAATGGCGAGAGGGC
CGGGGCGGCGTCCGAAGCGAGAGGAGATTCTACTACGCTTAGAGCACACCCTAGCTCTGCC
AGCTAGCTTTGGGGAGCTGCGTCTTACGGCAGGCTCCCAACACCAAGCAACCAGGGCTTGA
GGGTTGAAATGACGCTCGAACAGGCATGCCTACTAGAATACTAATAGGCGCAATGTGCGTT
CAAAGATTCGATGATTCACTGAATTCTGCAATTCACATTACTTATCGCATTTCGCTGCGTTCTT
CATCGATGCCAGAACCAAGAGATCCGTTGTTGAAAGTTTTGATTATTATGTTTTGTTACTCAG
AGTTCCACAGAAAAACAAGAGTTTAGTAGTCCACCGGCAGGCCAGCAAGCCCCTCGCGGGG
TGCGCCCTGCCGAGGCAACAGAAGGTAAGTTCACATGGGTTGGGAGTTGTGATAACTCAGT
AATGATCCCTCCGCAGGTTCACCCTACGGAA
>BSNB_0076_Acremonium_sp

GGTATGCTACCTGATCTGAGGTCAACCTTGAGAAGTGGGGGGTTTAACGGCGTGGTTCAACC
GCTATCCTGCCGCGAGAGGTTTAATTACTGCACGGAGGAGTTCGCGAGGGAACCGCCACTG
GATTTCAGGGCCAGCCGCCGCCAGGGGCAGGCTGATCCCCAACGCCAGGTCCCGCGAACGG
GTCCTGAGGGTTGAAATGACGCTCAGACAGGCATGCCCGCCAGAATACTGGCGGGCGCAAT
GTGCGTTCAAAGATTCGATGATTCACTGAATTCTGCAATTCACATTACTTATCGCATTTTGCT
GCGTTCTTCATCGATGCCAGAACCAAGAGATCCGTTGCTGAAAGTTTTGATTTATTTGCTTAT
GCCACTCAGAAATACACTAAAAGACAAGAGTTTGGAGCCTCCGGCGGACGCCTGGGTCCGG
GCCGCGCGAACGCGCCCGGGGCGAGGCCGCCGAAGCAACAGTGGTAGGTTCACAATGGTTT
GGGAGTTTTTACACTCGGTAATGATCCCTCCGCAGGTTACCCCTACGGAGG
>BSNB_0077_Pestalotiopsis_microspora

ATGGGGTATCCTACCTGATCCGAGGTCAACCACAAAAAATTGGGGGTTTAGCGGCTGGGAG

TTATAGCACCTAACAAAAGCGAGAAAAAAATTACTACGCTCAGAGGATACTACAAATCCGC
CGTTGTATTTCAGGAACTACAACTCCTAAGAGAAGTAGATTCCCAACACTAAGCTAGGCTTA
AGGGTTGAAATGACGCTCGAACAGGCATGCCCACTAGAATACTAATGGGCGCAATGTGCGT
TCAAAGATTCGATGATTCACTGAATTCTGCAATTCACATTACTTATCGCATTTCGCTGCGTTCT
TCATCGATGCCAGAACCAAGAGATCCGTTGTTGAAAGTTTTGACTTATTAAAATAAGACGCT
CAGATTACATAAAATAACAAGAGTTTAATGGTCCATCGGCAGCAGCTATAAGAAGACCTAT
AACTTCTGCCGAGGCAACAAAAGGTAAGTTCACATGGGTTGGGAGTTTAGAAAACTCTATA
ATGATCCCTCCGCAGGTTCACCCTACGGAAG
>BSNB_0078_Chaetomium_sp

NNNNNNNNNNNTTATAGGTCTGCTTGTAGATAAAGGGGTTTTACGGCCGGCACGCGCCGG
GACGCCGCGAGCGAGGTGTATTACTGCGCTACGGGTCCAGGCGCGCCCGCCACTTCTTTTCA
GGGCCCGCGGCGGCCGCGGGGCCCCAACACCGAGCAGGGCTCGAGGGTTGAAATGACGCT
CGAACAGGCATGCCCGCCGGAATGCCGGCGGGCGCAATGTGCGTTCAAAGATTCGATGATT
CGCTGAGTTCTGCAATTCACATTACTTATCGCATTTCGCTGCGTTCTTCATCGATGCCAGAAC
CAAGAGATCCGTTGTTGAAAGTTTTGACTCATTTAGTGTCTTCTCAGAGGGGCCTAAATCGCA
AGAGTTGTGCTGCGCTGCCGCCGGCGGGCGCCCTTGCGGGCTGCGGCCCCTCCCCGGGGCCG
CCCGCCGAAGCAACGAACAGTTGAAGAAACGTTCACGGGTGTATTAAGGAGAATGGAACTC
GTTAATGATCCCTCCGCAGGTTCACCTACGGAA
>BSNB_0085_Colletotrichum_gloeosporioides

TCGGGGTATCTACCTGATCCGAGGTCAACCTTTGGAAAATTGGGGGGTTTTACGGCAAGAGT
CCCTCCGGATCCCAGTGCGAGACGTAAAGTTACTACGCAAAGGAGGCTCCGGGAGGGTCCG
CCACTACCTTTGAGGGCCTACATCAGCTGTAGGGCCCCAACACCAAGCAGAGCTTGAGGGT
TGAAATGACGCTCGAACAGGCATGCCCGCCAGAATGCTGGCGGGCGCAATGTGCGTTCAAA
GATTCGATGATTCACTGAATTCTGCAATTCACATTACTTATCGCATTTCGCTGCGTTCTTCATC
GATGCCAGAACCAAGAGATCCGTTGTTAAAAGTTTTGATTATTTGCTTGTACCACTCAGAAG
AAACGTCGTTAAATCAGAGTTTGGTTATCCTCCGGCGGGCGCCGACCCGCCCGGAGGCGGG
AGGCCGGGAGGGTCGCGGAGACCCTACCCGCCGAAGCAACAGTTATAGGTATGTTCACAAA
GGGTTATAGAGCGTAAACTCAGTAATGATCCCTCCGCAGGTTCACCCTACGGAA
>BSNB_0087_Guignardia_mangiferae

CTGCATGCTACATGATCCGAGGTCAACCTTGGAAAAATAGACCGAAGGTCGATTGTCCGGC
GGCCGTCGCCCAGCACTCCAAAGCGAGATATTTTACTACTACGCTCGAGGCTAGGACGCCGT
CGCCGAGGTCTTCAAGGCACGTCCGGCAGCGGACGTTGCCCAATACCAAGCAGAGCTTGAG
GGTTGAAATGACGCTCGAACAGGCATGCCCTCCGGAATACCAGAGGGCGCAATGTGCGTTC
AAAGATTCGATGATTCACTGAATTCTGCAATTCACATTACTTATCGCATTTCGCTGCGTTCTTC
ATCGATGCCAGAACCAAGAGATCCGTTGTTGAAAGTTTTAATCAATTAAATGATATATCAGG
ACTTCACAAAATGAATTCTTGAGTTTTGTATACTGGCGGGCACTTAGCCGGGCGTCCTGGCC
AGTTAAGGCTGGGGGCGCCGGCCGCCTGGGTCGGAACCAGGTCGACCCGCCAAAGCAACA
TAGTGAGTACACAAGGGTGAGAAGGTCATTTCGGCGTTGTAGCGCCTACTCTGGAACCTTTC
AATAGAAGTTATTACATTTCAGTAATGATCCTTCCGCAGGTCACCCTTACGGAAG
>BSNB_0088_Xylaria_cubensis

NNNNNNNNGCTGAGCAGATTCGAGGTCAACCTTGAGAAGTTGGGGTTTTACGGCAGGGGA
TTAGTCCAACTAATAGGCGAGATAAAAAATTACTACGTCTAGAGTGTGAACCAACTCCGCC
ACTAACTTTGAGGAGCTACGTTACCGTAGGCTCCCAACACTAAGCAACTAAGGCTTAAGGG
TTGAAATGACGCTCGAACGGTCCTGCCCACTAGAATACTAATGGGCGCAATATGCGTTCAA
AGATTCGATGATTCACTGAATTCTGCAATTCACATTACTTATCGCATTTCGCTGCGTTCTTCAT
CGATGCCAGAACCAAGAGATCCGTTGTTGAAAGTTTTAACTTATTTAGTTATAGGTTCAGAA
TTCAATATCAAACAGAGTTTGGTGGGCCGCCGGCAGGCTTACCCGCACCTCCCGGGTAAGTC

TTACAGGGTAAGACTACGAGGTAGGCGCGACCTGCCGAGGCAACGTTAGGTATGTTCACAT
GGGTTTGGGAGTTATAGAACTCTTTAATGATCCCTCCGCAGGTTCACCTACGGAGGGA
>BSNB_0089_Xylaria_cubensis

NNNNNNNNGCTGAACGATTCTTAGGTCGCCTTGAGAAGTTGGGGTTTTACGGCAGGGGATT
AGTCCAACTAATAGGCGAGATAAAAAATTACTACGTCTAGAGTGTGAACCAACTCCGCCAC
TAACTTTGAGGAGCTACGTTACCGTAGGCTCCCAACACTAAGCAACTAAGGCTTAAGGGTTG
AAATGACGCTCGAACGGTCCTGCCCACTAGAATACTAATGGGCGCAATATGCGTTCAAAGA
TTCGATGATTCACTGAATTCTGCAATTCACATTACTTATCGCATTTCGCTGCGTTCTTCATCGA
TGCCAGAACCAAGAGATCCGTTGTTGAAAGTTTTAACTTATTTAGTTATAGGTTCAGAATTCA
ATATCAAACAGAGTTTGGTGGCCGCCGGCAGGCTTACCCGCACCTCCCGGGTAAGTCTTAC
AGGGTAAGACTACGAGGTAGGCGCGACCTGCCGAGGCAACGTTAGGTATGTTCACATGGGT
TTGGGAGTTATAGAACTCTTTAATGATCCCTCCGCAGGTTCACCTACGGAGGGA
>BSNB_0090_Xylaria_cubensis

NNNNNNNNNNNNNNCGCTTATGAGATATGCTTCGCAGAATTTGTGGGTTTGTACCCGTAG
GGGTATTAGTCCAACTAATAGGCGAGATAAAAAATTACTACGTCTAGAGTGTGAACCAACT
CCGCCACTAACTTTGAGGAGCTACGTTACCGTAGGCTCCCAACACTAAGCAACTAAGGCTTA
AGGGTTGAAATGACGCTCGAACGGTCCTGCCCACTAGAATACTAATGGGCGCAATATGCGT
TCAAAGATTCGATGATTCACTGAATTCTGCAATTCACATTACTTATCGCATTTCGCTGCGTTCT
TCATCGATGCCAGAACCAAGAGATCCGTTGTTGAAAGTTTTAACTTATTTAGTTATAGGTTCA
GAATTCAATATCAAACAGAGTTTGGTGGCCGCCGGCAGGCTTACCCGCACCTCCCGGGTA
AGTCTTACAGGGTAAGACTACGAGGTAGGCGCGACCTGCCGAGGCAACGTTAGGTATGTTC
ACATGGGTTTGGGAGTTATAGAACTCTTTAATGATCCCTCCGCAGGTTCACCTACGGAGGGA
>BSNB_0091_Xylaria_cubensis

NNNNNNNNNGAACATCCGCTTATTGATATGCTCCTCCGCTTATTGATATGCTCCTCCGCTTA
TTGATATGCTCCTCCGCTTATAGATATGTTCCTCCGCTTATWGWGAGGAYCCTCTCCTYCTCT
AACTGTGAGGAGCTACGTTACCGTGGGCTCCCAACACTAAGCAACTAAGGCTTAAGGGTTG
AAATGACGCTCGAACGGTCCTGCCCACTAGAATACTAATGGGCGCAATATGCGTTCAAAGA
TTCGATGATTCACTGAATTCTGCAATTCACATTACTTATCGCATTTCGCTGCGTTCTTCATCGA
TGCCAGAACCAAGAGATCCGTTGTTGAAAGTTTTAACTTATTTAGTTATAGGTTCAGAATTCA
ATATCAAACAGAGTTTGGTGGCCGCCGGCAGGCTTACCCGCACCTCCCGGGTAAGTCTTAC
AGGGTAAGACTACGAGGTAGGCGCGACCTGCCGAGGCAACGTTAGGTATGTTCACATGGGT
TTGGGAGTTATAGAACTCTTTAATGATCCCTCCGCAGGTTCACCTACGGAGGGA
>BSNB_0093_Colletotrichum_sp

TTGAGTCTGCTACCTGATCCGAGGTCAACCTTTGGAAAATTGGGGGTTTAACGGCTAGAGTC
CCTCCGAATCCCAGTGCGAGACAAAAGTTACTACGCAAAGGAGGCTCCGAGAGGGTCCGCC
ACTACCTTTGAGGGCCTACGTCAACCGTAGAGCCCCAACGCCAAGCAGTGCTTGAGGGTTG
AAATGACGCTCGAACAGGCATGCCCGCCAGAATGCTGGCGGGCGCAATGTGCGTTCAAAGA
TTCGATGATTCACTGAATTCTGCAATTCACATTACTTATCGCATTTCGCTGCGTTCTTCATCGA
TGCCAGAACCAAGAGATCCGTTGTTAAAAGTTTTGATTATTTGCTTGTGCCACTCAGAAGAA
ACGTCGTTAAATCAGAGTTTGGTTATCCTCCGGCGGACACCCCGCAGAGCGGGGCCGGGGG
CCCGGGAGCCGTCCGCCGAAGCAACAGTTAGGTATGTTCACAAAGGGTTATAGAGCGGTAA
CTCAGTAATGATCCCTCAGCAGGTTACCCCTACGGAA
>BSNB_0094_Diaporthe_phaseolorum

CTCGCATAGCTACATCGATTCGTAGGTCAAATTTTCAAAAGTTGGGGGTTTAACGGCAGGGC
ACCGCCAGGGCCTTCCAGAGCGAGGGTTTAACTACTACGCTCGGGGTCCTGGCGAGCTCGC
CACTAGATTTCAGGGCCTGCTTCGTTAGAAGCAGTGCCCCAACACCAAGCAATGCTTGAGG

GTTGAAATGACGCTCGAACAGGCATGCCCTCCGGAATACCAGAGGGCGCAATGTGCGTTCA
AAGATTCGATGATTCACTGAATTCTGCAATTCACATTACTTATCGCATTTCGCTGCGTTCTTCA
TCGATGCCAGAACCAAGAGATCCGTTGTTGAAAGTTTTGATTCATTTGTGTTTTTTCTCAGAG
TTTCAGTGTAAAAACAAGAGTTAACTTGGCCGCCGGCGTGCCTTCTCCTCACCGGGGTGAGG
GGCCTACTAGAGGCCAGCATGCGCCGAGGCAACAAAAGGTATAAGTTCACAAAGGGTTTCT
GGGTGCGCCTGGGGCGCGTTCCAGCAATGATCCCTCCGCAGGTTCACCCTACGGAA
>BSNB_0096_Diaporthe_sp

GCGCATAGCTGCATGATCCGTAGGTCAAAATTTACAGAAGTTGGGGGTTTAACGGCAGGGC
GCCGCCAGGGCCTTCCAGAACGAGATATAACTACTACGCTCGGGGTCCTAGCGAGCTCGCC
ACTAGATTTCAGGGCCTGCCCTTTTACAGGCAGTGCCCCAACACCAAGCCAGGCTTGAGGGT
TGAAATGACGCTCGAACAGGCATGCCCTCCGGAATACCAGAGGGCGCAATGTGCGTTCAAA
GATTCGATGATTCACTGAATTCTGCAATTCACATTACTTATCGCATTTCGCTGCGTTCTTCATC
GATGCCAGAACCAAGAGATCCGTTGTTGAAAGTTTTGATTCATTTATGTTTTTACTCAGAGA
TTCACTAAGAAACAAGAGTTTGGTTGGCCGCCGGCGGGCTGCTCCCTGTTCCCAGGGGGCCT
CAGGAGAGAGGCCGGCCTTCGCCGAGGCAACAATAGGTATAAGTTCACAAAGGGTTTCTGG
GTGCGCCGAGGCGCGTTCCAGCAATGATCCCTCCGCAGGTTCACCCTACGGAAG
>BSNB_0098_Colletotrichum_sp

TGCAGATGCTACCTGATCCGAGGTCAACCTTTGGAAAATTGGGGGTTTAACGGCTAGAGTCC
CTCCGAATCCCAGTGCGAGACAAAAGTTACTACGCAAAGGAGGCTCCGAGAGGGTCCGCCA
CTACCTTTGAGGGCCTACGTCAACCGTAGAGCCCCAACGCCAAGCAGTGCTTGAGGGTTGA
AATGACGCTCGAACAGGCATGCCCGCCAGAATGCTGGCGGGCGCAATGTGCGTTCAAAGAT
TCGATGATTCACTGAATTCTGCAATTCACATTACTTATCGCATTTCGCTGCGTTCTTCATCGAT
GCCAGAACCAAGAGATCCGTTGTTAAAAGTTTTGATTATTTGCTTGTGCCACTCAGAAGAAA
CGTCGTTAAATCAGAGTTTGGTTATCCTCCGGCGGACACCCCGCAGAGCGGGGCCGGGGGC
CCGGGAGCCGTCCGCCGAAGCAACAGTTAGGTATGTTCACAAAGGGTTATAGAGCGGTAAC
TCAGTAATGATCCCTCCAGCAGAGTTCACCCCTACGGGAA
>BSNB_0100_Diaporthe_sp

CGAAGAAAGCCTGCTGCAATGATCCGAGGTCTAAATTTCAGAAGTTGGGGGTTTAACGGCA
GGGCGCCGCCAGGGCCTTCCAGAACGAGATATAACTACTACGCTCGGGGTCCTAGCGAGCT
CGCCACTAGATTTCAGGGCCTGCCCTTTTACAGGCAGTGCCCCAACACCAAGCCAGGCTTGA
GGGTTGAAATGACGCTCGAACAGGCATGCCCTCCGGAATACCAGAGGGCGCAATGTGCGTT
CAAAGATTCGATGATTCACTGAATTCTGCAATTCACATTACTTATCGCATTTCGCTGCGTTCTT
CATCGATGCCAGAACCAAGAGATCCGTTGTTGAAAGTTTTGATTCATTTATGTTTTTACTCA
GAGATTCACTAAGAAACAAGAGTTTGGTTGGCCGCCGGCGGGCTGCTCCCTGTTCCCAGGG
GGCCTCAGGAGAGAGGCCGGCCTTCGCCGAGGCAACAATAGGTATAAGTTCACAAAGGGTT
TCTGGGTGCGCCGAGGCGCGTTCCAGCAATGATCCCTCCGCAGGTTCCCCCTACGGAAG
>BSNB_0102_Pestalotiopsis_sp

ATTGGGTATCCTACCTGATCCGAGGTCAACCCAGTAAAAAAGTTGGGGGTTTAGCGGCGAG
GGCCACGGCACCTACAGAAGCGAGAGGTATATTACTGCGCTCAGAGGTAGAACCGTCACTC
CGCCGGTGAATTTGAGGAGCTACAGGGTAACAGCTACAGGGTAGCTCCCGGGTAGTCTCCC
AACGCCAAGACTAGGTCTTGAGTGGTACTAATGACGCTCGAACAGGCATGCCCTCCAGAAT
GCTGGGGGGCGCAATGTGCGTTCAAAGATTCGATGATTCACTGAATTCTGCAATTCACATTA
CTTATCGCATTTCGCTGCGTTCTTCATCGATGCCAGAACCAAGAGATCCGTTGTTAAAGGTTT
TGACTTATTTTCATAAGACTCTCAGATGATAATAATACAAATAGTTTGGCTTTCCACCGGCGG
TCCGCCCGCTACAGGGTAAGCTGCAGGGTAAGACCGCCGAGGCAACGTAAGGTATAGGTTC
ACAAATGGTTTATACAGGAGTGTTTTAATACTCTGTAATGATCCCTCCGCAGGTTCACCCTAC
GGAA

>BSNB_0103_Xylaria_cubensis

NNNNNNNGGAGGAGGAACCATCCTATGCGGAGGTACCTATACAGTTGGCGGAGGGGTCGY
GCCTACCTCGAAGTCTTACMCTGTAAGACTTACCCGGGAGGTGCGGGTAAGCCTGCCGGCG
GCCCACCAAACTCTGTTTGATATTGAATTCTGAACCTATAACTAAATAAGTTAAAACTTTCA
ACAACGGATCTCTTGGTTCTGGCATCGATGAAGAACGCAGCGAAATGCGATAAGTAATGTG
AATTGCAGAATTCAGTGAATCATCGAATCTTTGAACGCATATTGCGCCCATTAGTATTCTAGT
GGGCAGGACCGTTCGAGCGTCATTTCAACCCTTAAGCCTTAGTTGCTTAGTGTTGGGAGCCT
ACGGTAACGTAGCTCCTCAAAGTTAGTGGCGGAGTTGGTTCACACTCTAGACGTAGTAATTT
TTTATCTCGCCTATTAGTTGGACTAATCCCCTGCCGTAAAACCCCAACTTCTCAAGGTTGACC
TCGAATCGGTTCAGACAAACTCGCTAAATTGAAGCATATCAATAAGCGGAGGAA
>BSNB_0106_Xylaria_cubensis

CGCTGTGCTACTGCACGGMTTTAGTTGCTTTGAGAATTGGGGGTTTCCCKGGGGGACMGMC
CAAGTAATARGGAGAAAAAAAATACTMCRCCTAGAGGGGGAACCAGCTCCGYCCCTAACT
TCGAGGAGCTACCTTACCGWASGGTCCGGMCACTAARCAACTAAGGSTTAGGGGGTGAAAT
GACGCTCSAACGGGGCTGCCCCCTARAATACTAATGGGGGCAATAGGGGTTCMAAGAATCS
ATGATTCCCTGAATTCTGCMATTCACATTACTTATCGCATTTCSCTGCSTTCTTCCTCGATGCC
MRAACCMARAGATCCSTTGTTGAAAGTTTTAACTTATTTRSTTATAGGTTCAGAATTCAATAT
CAAACARAGTTWGGTGGGCCGCCGGCAGGCTTACCCGCACCTCCCGGGTARGTCTTACAGG
GKAAGTCTACGAGGTASGCGCSACCTGCCGAGGCAACGTTAGGTATGTTCACATGGGTTTGG
GAGTTATARAACTCTTTAWTGATCCCYCCGCAGGTTCWCCTACGGAGGK
>BSNB_0107_Muscodor_sp

CTACCTGATCCGAGGTCAACCTTTGTATAAGGGGTTTTACGGCAGGGGCCGGGACCACTACA
GAAGCGAGATATAATTACTACGCTTAGAGTGAGAACCAACTCCGCCAATCACTTTAAGGAG
CTACGGGCCGTGCCGTAGGCTCCCAACGCTAAGCAACAAGGCTTAAGTGGTGAAATGACGC
TCGAACAGGCATGCCCACTAGAATGCTAATGGGCGCAATGTGCGTTCAAAGATTCGATGATT
CACTGAATTCTGCAATTCACATTACTTATCGCATTTCGCTGCGTTCTTCATCGATGCCAGAAC
CAAGAGATCCGTTGTTGAAAGTTTTAACTTATTAAGTTTGTTATTCAGAATTCCAAAGAAAA
CAGAGTTTAGTTGGCCGCCGGCGGGGATGACTACCGGGTAGCTGATGTACCTGAAACTACA
GGGTAACCACTAAGTGGTATTCTCCCGCAGGGTAGCCTCCGCCGCCGAAGCAACAAAGGTA
AGTTCACATAGGGTTGGGAGTTTAGAAAACTCTGTAATGATCCCTCCGCAGGTTCACCCTAC
GGAAA
>BSNB_0115_Guignardia_mangiferae

CCTACCTGATCCGAGGTCAACCTTGGAAAAATAGACCGAAGGTCGATTGTCCGGCGGCCGT
CGCCCAGCACTCCAAAGCGAGATATTTTACTACTACGCTCGAGGCTAGGACGCCGTCGCCG
AGGTCTTCAAGGCACGTCCGGCAGCGGACGTTGCCCAATACCAAGCAGAGCTTGAGGGTTG
AAATGACGCTCGAACAGGCATGCCCTCCGGAATACCAGAGGGCGCAATGTGCGTTCAAAGA
TTCGATGATTCACTGAATTCTGCAATTCACATTACTTATCGCATTTCGCTGCGTTCTTCATCGA
TGCCAGAACCAAGAGATCCGTTGTTGAAAGTTTTAATCAATTAAATGATATATCAGGACTTC
ACAAAATGAATTCTTGAGTTTTGTATACTGGCGGGCACTTAGCCGGGCGTCCTGGCCAGTTA
AGGCTGGGGGCGCCGGCCGCCTGGGTCGGAACCAGGTCGACCCGCCAAAGCAACATAGTG
AGTACACAAGGGTGAGAAGGTCATTTCGGCGTTGTAGCGCCTACTCTGGAACCTTTCAATAG
AAGTTATTACATTTCAGTAATGATCCTTCCGCAGGTTCACCTACGGAAGG
>BSNB_0118_Colletotrichum_gloeosporioides

GGGGTATTCCTACCTGATCCGAGGTCAACCTTTAGAAAATTGGGGGTTTAACGGCTAGAATC
CCTCCGAATCCCAGTGCGAGACAAAAGTTACTACGCAAAGGAGGCTCCGAGAGGGTCCGCC
ACTAGTTTTGGGGGCCTACGTCAACCGTAGAGCCCCAACACCAAGCAGTGCTTGAGGGTTG

AAATGACGCTCGAACAGGCATGCCCGCCAGAATGCTGGCGGGCGCAATGTGCGTTCAAAGA
TTCGATGATTCACTGAATTCTGCAATTCACATTACTTATCGCATTTCGCTGCGTTCTTCATCGA
TGCCAGAACCAAGAGATCCGTTGTTAAAAGTTTTGATTATTTGCTTGTGCCACTCAGAAGAA
ACGTCGTTAAATCAGAGTTTGGTTATCCTCCGGCGGACGCCCCGCGTGAGCGGGCCGGGG
GCCCGGGGGGCCGACCGCCGAAGCAACATGTAGGTATGTTCACAAAGGGTTATAGAGTGGT
AACTCGATAATGATCCCTCCGCAGGTTCACCTACGGAAG
>BSNB_0121_Xylaria_cubensis

CNGNGCGATTCGAGGTCAACCTTGAGAAGTTGGGGTTTTACGGCAGGGGATCAGTCCAACT
AATAGGCGAGATAAAAAATTACTACGTCTAGAGTGTGAACCAACTCCGCCACTAACTTTGA
GGAGCTACGTTACCGTAGGCTCCCAACACTAAGCAACTAAGGCTTAAGGGTTGAAATGACG
CTCGAACGGTCCTGCCCACTAGAATACTAATGGGCGCAATATGCGTTCAAAGATTCGATGAT
TCACTGAATTCTGCAATTCACATTACTTATCGCATTTCGCTGCGTTCTTCATCGATGCCAGAA
CCAAGAGATCCGTTGTTGAAAGTTTTAACTTATTTAGTTATAGGTTCAGAATTCAATATCAAA
CAGAGTTTGGTGGGCCGCCGGCAGGCTTACCCGCACCTCCCGGGTAAGTCTTACAGGGTAA
GACTACGAGGTAGGCGCGACCTGCCGAGGCAACGTTAGGTATGTTCACATGGGTTTGGGAG
TTATAGAACTCTTTAATGATCCCTCCGCAGGTTCACCTACGGANG
>BSNB_0122_Cystobasidium_minutum

TCTGACTGATTTGAGATCTAAAGCTTAAAGTGCTATAAAAGCGCATTAGAAGCACCTCTTAT
ATTTGAAGAAGACGTCCTTAGCGAAATAATTATTACGCCAAGTCAAACCGTCTATTTCAATA
GGGTTGCTCGTGTATTTCAGTTGAGCCGGCAATTACGCCGACAGACAACCATAATCCAAGCC
CACGCCCATTTCATTACAAAATAGGGGGGTTGAGAGTTTCATGATACTCAAACAGACATACT
CTTCGGAATACCAAAGAGTGCAAGGTGCGTTCAAAGATTCGATGATTCACTGAATTCTGCAA
TTCACATTACTTATCGCAATTCGCTGCGTTCTTCATCGATGCGAGAGCCAAGAGATCCGTTGC
TGAAAGTTTTGTTTTTGTTTATGCTCAATTAAGAGACTATTACATTCTTATACTAATGTGTTAA
AAGTGTGTGTAAAAAGAAGTGTGTGCACAGTGTAAGAAAATGAAATGGTCGGACTTCTAAA
AAGAACGTCCTAAAATTCATTAATGATCCTTCCGCAGGTTCACCTACGGAAGG
>BSNB_0123_Xylaria_sp

TTCCTCTGCGCGATTMAAGTCAGCTTTGAAAKTTGGGGGTTTTCCGGACGKGAACRCTCCRM
GCTCCCGGNGAGAGAAAAGTTACTACKGCKARAGAGAGGCCCCTCCACACCGCCCTGKATT
TCCGGGCCGGCCTCCCGCTCCCAGGGCTCCCCAAGTCCCCMTTAMCGACCCCCCTGACGGG
TTCAACGGGCCTGCCCMCTCTAATACTAGGGGGCCAATATGAATACTGGSATTCGATGATTC
CCTGAATTCATTAATTCACATTACTTATTCTGTTTCGCTGCTACCTTCCTCAATGCCCTAACCA
CTARATCCATTGTTGAAACTTTTAATTTATTTATGAATAKGTTCATAATTCAATATCTWACAC
ASWTAGGTGGGCCACCKGCAGGCTTATCCGCRCCTCCCGAGWAAATCTTGCWKGGTACCAC
TACMRGGYACCTGCKACCTGCCSAGGCAACSTTAAGTATGTTCACATGGGTTTGGGAGTTAT
ATAACTCTTTAATGATCCCTCCGCAGGATCRCCTACSGAGG
>BSNB_0124_Colletotrichum_sp

CTACCTGATCCGAGGTCAACCTTTGGAAAATTGGGGGGTTTTACGGCAAGAGTCCCTCCGGA
TCCCAGTGCGAGACGTAAAGTTACTACGCAAAGGAGGCTCCGGGAGGGTCCGCCACTACCT
TTGAGGGCCTACATCAGCTGTAGGGCCCCAACACCAAGCAGAGCTTGAGGGTTGAAATGAC
GCTCGAACAGGCATGCCCGCCAGAATGCTGGCGGGCGCAATGTGCGTTCAAAGATTCGATG
ATTCACTGAATTCTGCAATTCACATTACTTATCGCATTTCGCTGCGTTCTTCATCGATGCCAG
AACCAAGAGATCCGTTGTTAAAAGTTTTGATTATTTGCTTGTACCACTCAGAAGAAACGTCG
TTAAATCAGAGTTTGGTTATCCTCCGGCGGGCGCCGACCCGCCCGGGGGGGCGGGAGGCCG
GGAGGGTCACGGGGACCCTACCCGCCGAAGCAACAGTTGTAGGTATGTTCACAAAGGGTTG
TAGAGCGTAAACTCAGTAATCATCCCTCCGCAGGTTCACCTACGGAA

>BSNB_0125_Candida_etchellsii

CTTGCTTGATTGGGGGCCATAAAAAATATTACTGCACAGAGTTATTAACGTGTGCTGTTCCAT
TTCTTTGACTCCAATAAGGAGCAACACCTCGTAATCCACAAGAAGTAGATTAGAGAGAAAG
TTCGGCGCTCCAACAAGCATGCTACTAGGAGATCCTAGAAGCGCAATGTGCGTTCAAAGAT
TCAATGACTCACGTCTGCAATTCGCATTACCTATCGCGCTTTGCTGCGTTCTTCATCGATGTG
AGAACCAAGAGATCCGTTGTTGAAAGTTTTAAATTTTCAAGTTTTCAGATAAATTGGTAAAA
GTTTAAAAGTTGGGTAGAGCTAGGCTCCACCCAAGCAGTGCCTTCCACGCTTGCGCGCTTCT
GGCGTTTCACAATGTCGGCGTGTAGCCTTCAAAAATGATCCTTCCGCAGGTTCACCTACGGA
AG
>BSNB_0132_Colletotrichum_gloeosporioides

CTACCTGATCCGAGGTCAACCTTTGGAAAATTGGGGGGTTTTACGGCAAGAGTCCCTCCGGA
TCCCAGTGCGAGACGTAAAGTTACTACGCAAAGGAGGCTCCGGGAGGGTCCGCCACTACCT
TTGAGGGCCTACATCAGCTGTAGGGCCCCAACACCAAGCAGAGCTTGAGGGTTGAAATGAC
GCTCGAACAGGCATGCCCGCCAGAATGCTGGCGGGCGCAATGTGCGTTCAAAGATTCGATG
ATTCACTGAATTCTGCAATTCACATTACTTATCGCATTTCGCTGCGTTCTTCATCGATGCCAG
AACCAAGAGATCCGTTGTTAAAAGTTTTGATTATTTGCTTGTACCACTCAGAAGAAACGTCG
TTAAATCAGAGTTTGGTTATCCTCCGGCGGGCGCCGACCCGCCCGGGGGCGGGAGGCCGGG
AGGGTCACGGAGACCCTACCCGCCGAAGCAACAGTTATAGGTATGTTCACAAAGGGTTGTA
GAGCGTAAACTCAGTAATGATCCCTACGCAGGTTCACCTACGGAA
>BSNB_0133_Colletotrichum_gloeosporioides

CTACCTGATCCGAGGTCAACCTTTGGAAAATTGGGGGGTTTTACGGCAAGAGTCCCTCCGGA
TCCCAGTGCGAGACGTAAAGTTACTACGCAAAGGAGGCTCCGGGAGGGTCCGCCACTACCT
TTGAGGGCCTACATCAGCTGTAGGGCCCCAACACCAAGCAGAGCTTGAGGGTTGAAATGAC
GCTCGAACAGGCATGCCCGCCAGAATGCTGGCGGGCGCAATGTGCGTTCAAAGATTCGATG
ATTCACTGAATTCTGCAATTCACATTACTTATCGCATTTCGCTGCGTTCTTCATCGATGCCAG
AACCAAGAGATCCGTTGTTAAAAGTTTTGATTATTTGCTTGTACCACTCAGAAGAAACGTCG
TTAAATCAGAGTTTGGTTATCCTCCGGCGGGCGCCGACCCGCCCGGGGGCGGGAGGCCGGG
AGGGTCACGGAGACCCTACCCGCCGAAGCAACAGTTATAGGTATGTTCACAAAGGGTTGTA
GAGCGTAAACTCAGTAATGATCCATCCGCAGGTTCACCTACGGAA
>BSNB_0135_Guignardia_mangiferae

GTATCCCTRCCTGATCCGAGGTCAACCTTGGAAAAATAGACCGAAGGTCGATTGTCCGGCGG
CCGTCGCCCAGCACTCCAAAGCGAGATATTTTACTACTACGCTCGAGGCTAGGACGCCGTCG
CCGAGGTCTTCAAGGCACGTCCGGCAGCGGACGTTGCCCAATACCAAGCAGAGCTTGAGGG
TTGAAATGACGCTCGAACAGGCATGCCCTCCGGAATACCAGAGGGCGCAATGTGCGTTCAA
AGATTCGATGATTCACTGAATTCTGCAATTCACATTACTTATCGCATTTCGCTGCGTTCTTCAT
CGATGCCAGAACCAAGAGATCCGTTGTTGAAAGTTTTAATCAATTAAATGATATATCAGGAC
TTCACAAAATGAATTCTTGAGTTTTGTATACTGGCGGGCACTTAGCCGGGCGTCCTGGCCAGT
TAAGGCTGGGGGCGCCGGCCGCCTGGGTCGGAACCAGGTCGACCCGCCAAAGCAACATAG
TGAGTACACAAGGGTGAGAAGGTCATTTCGGCGTTGTAGCGCCTACTCTGGAACCTTTCAAT
AGAAGTTATTACATTTCAGTAATGATCCTTCCGCAGGTTCACCTACGGAAGG
>BSNB_0154_Rhizomucor_variabilis

TTTGAGTATGCTTCTCAGCATATTTCTAATTTACTGTGAACTGTTTTACTGTTTAGCGTTTTGAG
GGATTGCCTAAAGATTATAGGGATAGGTCTTTAGGATGTTAACCTAGCTAAAGTCAGGCTTA
GGCCTGGTATCCTAATTCATTATTTACCAAAAGAATTCAGAATTAATTATTGTAACATAAGC
GTAAAAAACTTATAAAACAACTTTTAACAACGGATCTCTTGGTTCTCGCATCGATGAAGAAC
GTAGCAAAGTGCGATAACTAGTGTGAATTGCATATTCAGTGAATCATCGAGTCTTTGAACGC

ATCTTGCGCTCAATGGTATTCCATTGAGCACGCCTGTTTCAGTATCAACAACAACCCACATC
CACAATTTTGTTGTGAATGGAAATGAGAGTAATCGACGTTAAAATTGAACTCTTTAAAATTA
TTAGGCCTGAACTATTGTTCTTTTAGCCTGAACATTTTTTTAATATAAAGGAATGCTCTAGTT
ATTAAGACTGTCTTGGGGGCCTCCCAAATAAATCATTTTTTAAACTTGATCTGAAATCAGGTG
GGATTACCCGCTGAACTTAAGCATATCAATAAGCGGAGGAA

>BSNB_0155_Rhizomucor_variabilis

TTTAGAGTATGCTTCTCAGCATATTTCTAATTTACTGTGAACTGTTTTACTGTTTAGCGTTTTG
AGGGATTGCCTAAAGATTATAGGGATAGGTCTTTAGGATGTTAACCTAGCTAAAGTCAGGCT
TAGGCCTGGTATCCTAATTCATTATTTACCAAAAGAATTCAGAATTAATTATTGTAACATAAG
CGTAAAAAACTTATAAAACAACTTTTAACAACGGATCTCTTGGTTCTCGCATCGATGAAGAA
CGTAGCAAAGTGCGATAACTAGTGTGAATTGCATATTCAGTGAATCATCGAGTCTTTGAACG
CATCTTGCGCTCAATGGTATTCCATTGAGCACGCCTGTTTCAGTATCAACAACAACCCACAT
CCACAATTTTGTTGTGAATGGAAATGAGAGTAATCGACGTTAAAATTGAACTCTTTAAAATT
ATTAGGCCTGAACTATTGTTCTTTTAGCCTGAACATTTTTTTAATATAAAGGAATGCTCTAGT
TATTAAGACTGTCTTGGGGGCCTCCCAAATAAATCATTTTTTAAACTTGATCTGAAATCAGGT
GGGATTACCCGCTGAACTTAAGCATATCAATAAGGCGGAGGAA

>BSNB_0167_Penicillium_shearii

TCTGGGTCAACCTCCCACCCGTGTTTAACGAACCTTGTTGCTTCGGCGGGCCCGCCTCACGGC
CGCCGGGGGGCATMSGCGCCCGGGCCCGCGCCCGCCRAAGACACCTGTGAACTCTGTCTGA
AGTTGCASYCTGAGAAACTATTTAAATTAGTTMAAACTTTCAACSACGGATCTCTTGGTTCCG
GCMTCGATGAASAACMCASCSAAATGCSATAAATAATGTGAATTGCASAATTCAGTGAATCA
TCRAGTCTTTGAACSCACATTGCKCCCTCTGGTATTCCGGARGGCMTGCCTGTCCGAGCGTC
WTTGCTGCCCTCAMGCACGGCTTGTGTGTTGGGACCCGKCTTACCCCTCCCGGGGGGACAG
GGCCRAARAGSCACSGMKGCATCGCGTSCCGMWCTCCATCTTATGG

>BSNB_0168_Penicillium_simplicissimum

AGGTTTCCGTAGGTGAACCTGCGGAAGGATCATTACCGAGTGAGGGCCCTCTGGGTCCAAC
CTCCCACCCGTGTTTATCGTACCTTGTTGCTTCGGCGGGCCCGCCTCACGGCCGCCGGGGGG
CATCCGCTCCCGGGCCCGCGCCCGCCGAAGACACCAATGAACTCTGTCTGAAGATTGCAGT
CTGAGCAGATTAGCTAAATCAGTTAAAACTTTCAACAACGGATCTCTTGGTTCCGGCATCGA
TGAAGAACGCAGCGAAATGCGATACGTAATGTGAATTGCAGAATTCAGTGAATCATCGAGT
CTTTGAACGCACATTGCGCCCCCTGGTATTCCGGGGGGCATGCCTGTCCGAGCGTCATTGCT
GCCCTCAAGCACGGCTTGTGTGTTGGGCTCCGCCCCCCGGCTCCCGGGGGGCGGGCCCGAA
AGGCAGCGGCGGCACCGCGTCCGGTCCTCGAGCGTATGGGGCTTCGTCACCCGCTCTGTAGG
CCCGGCCGGCGCCCGCCGGCGACCCCAATCAATCTATCCAGGTTGACCTCGGATCCAGGGT
AGGGATCCCA
>BSNB_0174_Candida_tropicalis

CACCACATGTGTTTTTTATTGAACAAATTTCTTTGGTGGCGGGAGCAATCCTACCGCCAGAG
GTTATAACTAAACCAAACTTTTTATTTACAGTCAAACTTGATTTATTATTACAATAGTCAAAA
CTTTCAACAACGGATCTCTTGGTTCTCGCATCGATGAAGAACGCAGCGAAATGCGATACGTA
ATATGAATTGCAGATATTCGTGAATCATCGAATCTTTGAACGCACATTGCGCCCTTTGGTATT
CCAAAGGGCATGCCTGTTTGAGCGTCATTTCTCCCTCAAACCCCCGGGTTTGGTGTTGAGCA
ATACGCTAGGTTTGTTTGAAAGAATTTAACGTGGAAACTTATTTTAAGCGACTTAGGTTTATC
CAAAAMCSTTWATTTGGCTRKGGGCCCCCMCAATTTTTTTCKWACCTTGAACCTMAATTAGG
GWAGAACTCCCCSTTAAATTTARSCTAATCATSAAGCGAAGAA

>BSNB_0175_Rhizomucor_variabilis

TTTAGAGTATGCTTCTCAGCATATTTCTAATTTACTGTGAACTGTTTTACTGTTTAGCGTTTTG
AGGGATTGCCTAAAGATTATAGGGATAGGTCTTTAGGATGTTAACCTAGCTAAAGTCAGGCT
TAGGCCTGGTATCCTAATTCATTATTTACCAAAAGAATTCAGAATTAATTATTGTAACATAAG
CGTAAAAAACTTATAAAACAACTTTTAACAACGGATCTCTTGGTTCTCGCATCGATGAAGAA
CGTAGCAAAGTGCGATAACTAGTGTGAATTGCATATTCAGTGAATCATCGAGTCTTTGAACG
CATCTTGCGCTCAATGGTATTCCATTGAGCACGCCTGTTTCAGTATCAACAACAACCCACAT
CCACAATTTTGTTGTGAATGGAAATGAGAGTAATCGACGTTAAAATTGAACTCTTTAAAATT
ATTAGGCCTGAACTATTGTTCTTTTAGCCTGAACATTTTTTTTAATATAAAGGAATGCTCTAGT
TATTAAGACTGTCTTGGGGGCCTCCCAAATAAATCATTTTTTAAACTTGATCTGAAATCAGGT
GGGATTACCCGCTGAACTTAAGCATATCAATAAGCGGAGGAA

>BSNB_0177_Rhizomucor_variabilis

TTTGAGTATGCTTCTCAGCATATTTCTAATTTACTGTGAACTGTTTTACTGTTTAGCGTTTTGAG
GGATTGCCTAAAGATTATAGGGATAGGTCTTTAGGATGTTAACCTAGCTAAAGTCAGGCTTA
GGCCTGGTATCCTAATTCATTATTTACCAAAAGAATTCAGAATTAATTATTGTAACATAAGC
GTAAAAAACTTATAAAACAACTTTTAACAACGGATCTCTTGGTTCTCGCATCGATGAAGAAC
GTAGCAAAGTGCGATAACTAGTGTGAATTGCATATTCAGTGAATCATCGAGTCTTTGAACGC
ATCTTGCGCTCAATGGTATTCCATTGAGCACGCCTGTTTCAGTATCAACAACAACCCACATC
CACAATTTTGTTGTGAATGGAAATGAGAGTAATCGACGTTAAAATTGAACTCTTTAAAATTA
TTAGGCCTGAACTATTGTTCTTTTAGCCTGAACATTTTTTTTAATATAAAGGAATGCTCTAGTT
ATTAAGACTGTCTTGGGGGCCTCCCAAATAAATCATTTTTTAAACTTGATCTGAAATCAGGTG
GGATTACCCGCTGAACTTAAGCATATCAATTAAGCGGAGGAA

>BSNB_0196_Cladosporium_sp

TGACCCGGCTACGGCGGGAGTTCATAACCCTTTGTTGTCCGACTCTGTTGCCTCCGGGGCGA
CCCTGCCTTCGGGCGGGGGCTCCGGGTGGACACTTCAAACTCTTGCGTAACTTTGCAGTCTG
AGTAAACTTAATTAATAAATTAAAACTTTTAACAACGGATCTCTTGGTTCTGGCATCGATGA
AGAACGCAGCGAAATGCGATAAGTAATGTGAATTGCAGAATTCAGTGAATCATCGAATCTT
TGAACGCACATTGCGCCCCCTGGTATTCCGGGGGGCATGCCTGTTCGAGCGTCATTTCACCA
CTCAAGCCTCGCTTGGTATTGGGCAACGCGGTCCGCCGCGTGCCTCAAATCGTCCGGCTGGG
TCTTCTGTCCCCTAAGCGTTGTGGAAACTATTCGCTAAAGGGTGTTCGGGAGGCTACGCCGT
AAAACAACCCCATTTCTAAGGTTGACCTCGGATCAGGTAGGGATACCCGCTGAACTTAAGC
ATATCAATTAAGCGGAGGAA

>BSNB_0197_Penicillium_sanguifluum

GGAGCTCTGGGTCAACCTCCCACCCGTGTTTAACGAACCTTGTTGCTTCGGCGGGCCCGCCT
CACGGCCGCCGGGGGGCATCCGCCCCCGGGCCCGCGCCCGCCGAAGACACCTGTGAACACT
GTCTGAAGTTGCAGTCTGAGAAACTAGCTAAATTAGTTAAAACTTTCAACAACGGATCTCTT
GGTTCCGGCATCGATGAAGAACGCAGCGAAATGCGATAAATAATGTGAATTGCAGAATTCA
GTGAATCATCGAGTCTTTGAACGCACATTGCGCCCTCTGGTATTCCGGAGGGCATGCCTGTC
CGAGCGTCATTGCTGCCCTCAAGCACGGCTTGTGTGTTGGGCCCCCGTCCCCCCGCCAGGGG
GGACGGGCCCGAAAGGCAGCGGCGGCACCGCGTCCGGTCCTCGAGCGTATGGGGCTCTGTC
ACCCGCTCTTGTAGGCCCGGCCGGCGCCAGCCGACCCCCCTCAATCTATTTTTTCAGGTTGAC
CTCGGATCAGGTAGGGATACCCGCTGAACTTAAGCATATCAATAAGCGGAGGAA

>BSNB_0201_Exophiala_xenobiotica

GAGATNAGTATAGTACCTTGTAATCCGCTTGGTGTATACCGGGTGCCGCCAAACCCTAGATC
CGATACGTGCTCAGTTAAGAAGCTCAGTGTACCGGGGGTTCAACAGCCGCCGTCATTGTCTT
TAGGAGGGGTCTAGGGGTACCTAAACCAAACCGTCCAACACCAAGCCGGGGGCTTGAGGG
GTGAAAATGACGCTCGAACAGGCATGCCCTTCGGAATACCAAAGGGCGCAATGTGCGTTCA
AAGATTCGATGACTCACTGGAATTCTGCAATTCGCATTACTTATCGCATTTCGCTGCGTTCTT
CATCGATGCCAGAACCAAGAGATCCGTTGTTGAAAGTTTTGCTTTAATTTTTTGTTTACTCA
GACAATACATGTTTTGTTCAAGAGTTTTTGGTTTGGGCTATCGGTAGGCACTCTCCAGAGGAC
GTTTTACGGTCCTCCGGTAGGACGAGCCAGACCTACCGAAGCAACACAAGGTATAATAAGC
AAAGGGTTGGGAGATCGGGCCTTTGAGGACCCTAACTCGGTAATGATCCTTCCGCAGGTTCA
CCTACGGAAGGA
>BSNB_0212_Pseudallescheria_boydii

GTACCTACCTGATCCGAGGTCAACCATCTGGAGTTATAGGTGGTTTGACGGCAGGCCTCCGC
CGGGACCCAATGCGAGCTTGCAAAAGAGACTTACTACGCAGAAGGCAACCGCGGCGGGAC
CGCCACTGTATTTCAGGGCCTACGGAGGGTCGCGAAGACTCGCCGTAGCGCCCCAACACCG
ACCCTGAGCTTCCCTGAGGAAACGGAGGTTCGAGGGTTGAAATGACGCTCGGACAGGCATG
CCCGGCAGATTACTGCCGGGCGCAATGTGCGTTCAAAGATTCGATGATTCACTGAATTCTGC
AATTCACATTACTTATCGCATTTCGCTGCGTTCTTCATCGATGCCAGAACCAAGAGATCCGTT
GTTGAAAGTTTTAACTTGTTTTTTGTTTTCAAATCAGAACTGTAATCCGCTGTAAAATTCAAA
GAGTTTGGTGCTGCCGGCGGGAGGGCATCGTCCTCTTTTCAGAGGGGGCGCTGACCACGCCG
CCGAGGCAACAGAACATAGGTAAGGTTCACAATGGGTTTGGAGTAGTAACTCTGTAATGAT
CCCTCCGCAGGTTCACCTACGGAA
>BSNB_0216_Scedosporium_boydii

ACAAGGTCTCCGTTGGTGAACCAGCGGAGGGATCATTACAGAGTTACTACTCCAAACCCATT
GTGAACCTTACCTATGTTCTGTTGCCTCGGCGGCGTGGTCAGCGCCCCCTCTGAAAAGAGGA
CGATGCCCTCCCGCCGGCAGCACCAAACTCTTTGAATTTTACAGCGGATTACAGTTCTGATTT
GAAAACAAAAAACAAGTTAAAACTTTCAACAACGGATCTCTTGGTTCTGGCATCGATGAAG
AACGCAGCGAAATGCGATAAGTAATGTGAATTGCAGAATTCAGTGAATCATCGAATCTTTG
AACGCACATTGCGCCCGGCAGTAATCTGCCGGGCATGCCTGTCCGAGCGTCATTTCAACCCT
CGAACCTCCGTTTCCTCAGGGAAGCTCAGGGTCGGTGTTGGGGCGCTACGGCGAGTCTTCGC
GACCCTCCGTAGGCCCTGAAATACAGTGGCGGTCCCGCCGCGGTTGCCTTCTGCGTAGTAAG
TCTCTTTTGCAAGCTCGCATTGGGTCCCGGCGGAGGCCTGCCGTCAAACCACCTATAACTTCC
AGATGGTTTGACCTCGGA
>BSNB_0218_Penicillium_citrinum

GGATTGCTGCATGATTCGAGGTCAACCTGAGATAATTAAAGGTTGGGGGTCGGCTGGCGCCG
GCCGGGCCTACTAGAGCGGGTGACGAAGCCCCATACGCTCGAGGACCGGACGCGGTGCCG
CCGCTGCCTTTCGGGCCCGTCCCCCCGGCGGGGGGACGGGGCCCAACACACAAGCCGGGC
TTGAGGGCAGCAATGACGCTCGGACAGGCATGCCCTCCGGAATACCAGAGGGCGCAATGTG
CGTTCAAAGACTCGATGATTCACTGAATTCTGCAATTCACATTAGTTATCGCATTTCGCTGCG
TTCTTCATCGATGCCGGAACCAAGAGATCCGTTGTTGAAAGTTTTAACTAATTTCGTTATAGG
TCTCAGACTGCAACTTCAGACAGCGTTCAGGGGGGCCGTCGGCGGGCGCGGGGCCCGCCGA
GGCAACATAGGTTCGGGCAACACGGGTGGGAGGTTGGGCCCCC

>BSNB_0220_Paecilomyces_formosus

GTTTCCGTAGGTGAACCTGCGGAAGGATCATTACCGAGTGAGGGTCCCACGAGGCCCAACC
TCCCATCCGTGTTGAACTACACCTGTTGCTTCGGCGGGCCCGCCGTGGTTCACGCCCGGCCG
CCGGGGGGCCTTGTGCTCCCGGGCCCGCGCCCGCCGAAGACCCCTCGAACGCTGCCCTGAA
GGTTGCCGTCTGAGTATAAAATCAATCATTAAAACTTTCAACAACGGATCTCTTGGTTCCGG
CATCGATGAAGAACGCAGCGAAATGCGATAAGTAATGTGAATTGCAGAATTCCGTGAATCA

TCGAATCTTTGAACGCACATTGCGCCCCCTGGCATTCCGGGGGGCATGCCTGTCCGAGCGTC
ATTGCTAACCCTCCAGCCCGGCTGGTGTGTTGGGTCGACGTCCCCCCCCCGGGGGACGGGCC
CGAAAGGCAGCGGCGGCGCCGCGTCCGATCCTCGAGCGTATGGGGCTTTGTCACGCGCTCT
GGTAGGGTCGGCCGGCTGGCCAGCCAGCGACCTCACGGTCACCTATTTTTCTCTTAGGTTG
ACCTCGGATC
>BSNB_0222_Scedosporium_boydii

NNNNNNNNAGTACTACTCCAAACCCATTGTGACCTTACCTGTGTTCTGTTGCCTCGGCGGC
GTGGTCAGCGCCCCTCTGAAAAGAGGACGATGCCCTCCCGCCGGCAGCACCAAACTCTTTG
AATTTTACAGCGGATTACAGTTCTGATTTGAAAACAAAAAACAAGTTAAAACTTTCAACAAC
GGATCTCTTGGTTCTGGCATCGATGAAGAACGCAGCGAAATGCGATAAGTAATGTGAATTGC
AGAATTCAGTGAATCATCGAATCTTTGAACGCACATTGCGCCCGGCAGTAATCTGCCGGGCA
TGCCTGTCCGAGCGTCATTTCAACCCTCGAACCTCCGTTTCCTCAGGGAAGCTCAGGGTCGG
TGTTGGGGCGCTACGGCGAGTCTTCGCGACCCTCCGTAGGCCCTGAAATACAGTGGCGGTCC
CGCCGCGGTTGCCTTCTGCGTAGTAAGTCTCTTTTGCAAGCTCGCATTGGGTCCCGGCGGAGG
CCTGCCGTCAAACCACCTATAACTCCAGATGGTTTGACCTCGGATCAGGTAGGGTTACCCGC
TGAACTTAAGCATATCAATAAGCGGAGGAAA

>BSNB_0224_Paecilomyces_formosus

NNNNNNNNTCCTACCTGATCCGAGGTCACCTAAGAGAAAAAATAGGTGACCGTGAGGTCG
CTGGCTGGCCAGCCGGCCGACCCTACCAGAGCGCGTGACAAAGCCCCATACGCTCGAGGAT
CGGACGCGGCGCCGCCGCTGCCTTTCGGGCCCGTCCCCCGGGGGGGGGACGTCGACCCAAC
ACACCAGCCGGGCTGGAGGGTTAGCAATGACGCTCGGACAGGCATGCCCCCCGGAATGCCA
GGGGGCGCAATGTGCGTTCAAAGATTCGATGATTCACGGAATTCTGCAATTCACATTACTTA
TCGCATTTCGCTGCGTTCTTCATCGATGCCGGAACCAAGAGATCCGTTGTTGAAAGTTTTAAT
GATTGATTTTATACTCAGACGGCAACCTTCAGGGCAGCGTTCGAGGGGTCTTCGGCGGGCGC
GGGCCCGGGAGCACAAGGCCCCCCGGCGGCCGGGCGTGAACCACGGCGGGCCCGCCGAAG
CAACAGGTGTAGTTCAACACGGATGGGAGGTTGGGCCTCGTGGGACCCTCACTCGGTAATG
ATCCTTCCGCAGGTTCACCTACGGAAGGAT
>BSNB_0225_Pestalotiopsis_microspora

AGTTTTATCTCTGCTGCCTGATCCGAGGTCAACCATTAAAAATTGGGGGGTTTAGCGGCTAA
AGACGCTGCAACTCCAGTCAAAAGCGAGATAAAAATTACTACGCTCAGAGGATATCGCAGA
TCCGCCGTTGTATTTCAGGAGCTACAGCTAGCAAAAGCAGTAGGCTCCCAACACTAAGCTA
GGCTTAAGGGTTGAAATGACGCTCGAACAGGCATGCCCACTAGAATACTAATGGGCGCAAT
GTGCGTTCAAAGATTCGATGATTCACTGAATTCTGCAATTCACATTACTTATCGCATTTCGCT
GCGTTCTTCATCGATGCCAGAACCAAGAGATCCGTTGTTGAAAGTTTTGACTTATTAAAATA
AGACGCTCAGATAACCATAAAATAACAAGAGTTTGGTAGTCCACCGGCAGCCGTTGCAGGG
TAAGCCGTTCCAGGGTAAGGCGCTACAGGGTAGGCCGTTCCAAGGTAAGGTGCACCGAGCA
GCTTCTGCCGAGGCAACAATGGTAAGTTCACATGGGTTGGGAGTTTAGAAAACTCTATAATG
ATCCCTCCGCAGGTTCACCCTACGGA

>BSNB_0226_Pseudallescheria_boydii

NNNNNNNTACCTACCTGATCCGAGGTCAAACCATCTGGAGTTATAGGTGGTTTGACGGCAG
GCCTCCGCCGGGACCCAATGCGAGCTTGCAAAAGAGACTTACTACGCAGAAGGCAACCGC
GGCGGGACCGCCACTGTATTTCAGGGCCTACGGAGGGTCGCGAAGACTCGCCGTAGCGCCC
CAACACCGACCCTGAGCTTCCCTGAGGAAACGGAGGTTCGAGGGTTGAAATGACGCTCGGA
CAGGCATGCCCGGCAGATTACTGCCGGGCGCAATGTGCGTTCAAAGATTCGATGATTCACTG
AATTCTGCAATTCACATTACTTATCGCATTTCGCTGCGTTCTTCATCGATGCCAGAACCAAGA
GATCCGTTGTTGAAAGTTTTAACTTGTTTTTTGTTTTCAAATCAGAACTGTAATCCGCTGTAAA

ATTCAAAGAGTTTGGTGCTGCCGGCGGGAGGGCATCGTCCTCTTTTCAGAGGGGGCGCTGAC
CACGCCGCCGAGGCAACAGAACATAGGTAAGGTTCACAATGGGTTTGGAGTAGTAACTCTG
TAATGATCCCTCCGCAGGTTCACCCTACGGAA

>BSNB_0227_Aspergillus_aculeatus

TTAGACTAGCTAGCTCCGCTTGTTGATCTGCAACCAACGGAGTGATCGGTGGATTGACGGCA
GGCCTCCGCCGGGACCCAATGCGAGCTTGCAAAAGAGACTTACTACGCAGAAGGCAACCG
CGGCGGGACCGCCACTGTATTTCAGGGCCTACGGAGGGTCGCGAAGACTCGCCGTAGCGCC
CCAACACCGACCCTGAGCTTCCCTGAGGAAACGGAGGTTCGAGGGTTGAAATGACGCTCGG
ACAGGCATGCCCGGCAGATTACTGCCGGGCGCAATGTGCGTTCAAAGATTCGATGATTCACT
GAATTCTGCAATTCACATTACTTATCGCATTTCGCTGCGTTCTTCATCGATGCCAGAACCAAG
AGATCCGTTGTTGAAAGTTTTAACTTGTTTTTGTTTTCAAATCAGAACTGTAATCCGCTGTAA
AATTCAAAGAGTTTGGTGCTGCCGGCGGGAGGGCATCGTCCTCTTTTCAGAGGGGGCGCTGA
CCACGCCGCCGAGGCAACAGAACATAGGTAAGGTTCACAATGGGTTTGGAGTAGTAACTCT
GTAATGATCCCTCCGCAGGTTCACCTACGGA

>BSNB_0228_Penicillium_rubidurum

TAACAAGGTTTCCGTAGGTGAACCTGCGGAAGGATCATTACCGAGTGAGGACCCTCTGGGT
CCAACCTCCCACCCGTGTTTATCGTACCTTGTTGCTTCGGCGGGCCCGCCGCAAGGCCGCCG
GGGGGCTTCCGTCCCCGGGCCCGTGCCCGCCGAAGACACCTGTGAACGCTGTATGAAGATT
GCAGTCTGAGCGACAAGCTAAATTTGTTAAAACTTTCAACAACGGATCTCTTGGTTCCGGCA
TCGATGAAGAACGCAGCGAAATGCGATAAGTAATGTGAATTGCAGAATTCAGTGAATCATC
GAGTCTTTGAACGCACATTGCGCCCCCTGGTATTCCGGGGGGCATGCCTGTCCGAGCGTCAT
TGCTGCCCTCAAGCACGGCTTGTGTGTTGGGCCCTCGTCCCCCGGGACGGCCCGAAAGGCA
GCGGCGGCACCGCGTCCGGTCCTCGAGCGTATGGGGCTTCGTCACCCGCTCTGCAGGCCCGG
CCGGCGCCTGCCGACACCATCAATCTTTTTTTCCAGGTTGACCTCGGATCAGGTAGGATCCC
AT
>BSNB_0243_Penicillium_araracuarense

AGGAGAAGTTCGTAACAAGGTTTCCGTAGGTGAACCTGCGGAAGGATCATTACCGAGTGAG
GGCCCTCTGGGTCCAACCTCCCACCCGTGTTTATCGTACCTTGTTGCTTCGGCGGGCCCGCCT
CACGGCCGCCGGGGGGCATCTGCCCCCGGGCCCGCGCCCGCCGAAGACACCAATGAACTCT
TGTCTGAAGATTGCAGTCTGAGCAGATTAGCCTAAATCAGTTAAAACTTTCAACAACGGATC
TCTTGGTTCCGGCATCGATGAAGAACGCAGCGAAATGCGATACGTAATGTGAATTGCAGAA
TTCAGTGAATCATCGAGTCTTTGAACGCACATTGCGCCCCCTGGTATTCCGGGGGGCATGCC
TGTCCGAGCGTCATTGCTGCCCTCAAGCACGGCTTGTGTGTTGGGCTTCGCCCCCCGGCTCCC
GGGGGGCGGGCCCGAAAGGCAGCGGCGGCACCGCGTCCGGTCCTCGAGCGTATGGGGCTTC
GTCACCCGCTCTGTAGGCCCGGCCGGCGCCCGCCGGCGACCCCAATCAATCTTTCCAGGTTG
ACCTCGGATCAGGGTAAGGG
>BSNB_0244_Penicillium_araracuarense

AGCATCTGGGTCAACCTCCCACCCGTGTTTATCGTACCTTGTTGCTTCGGCGGGCCCGCCTCA
CGGCCGCCGGGGGGCATCTGCCCCCGGGCCCGCGCCCGCCGAAGACACCAATGAACTCTTG
TCTGAAGATTGCAGTCTGAGCAGATTAGCTAAATCAGTTAAAACTTTCAACAACGGATCTCT
TGGTTCCGGCATCGATGAAGAACGCAGCGAAATGCGATACGTAATGTGAATTGCAGAATTC
AGTGAATCATCGAGTCTTTGAACGCACATTGCGCCCCCTGGTATTCCGGGGGGCATGCCTGT
CCGAGCGTCATTGCTGCCCTCAAGCACGGCTTGTGTGTTGGGCTTCGCCCCCCGGCTCCCGG
GGGGCGGGCCCGAAAGGCAGCGGCGGCACCGCGTCCGGTCCTCGAGCGTATGGGGCTTCGT

CACCCGCTCTGTAGGCCCGGCCGGCGCCCGCCGGCGACCCCAATCAATCTTTCCAGGTTGAC
CTCGGATCAGGTAGGGATACCCGCTGAACTTAAGCATATCAATAACCCAGAGGAAA

>BSNB_0250_Aspergillus_sp

GGGTCTCGTACGAGTCTCTGGGTCACCTCCCACCCGTGTCTATTGTACCTTGTTGCTTCGGCG
GGCCCGCCGTCAGGCCGCCGGGGGGCATCTGCCCCCGGGCCCGCGCCCGCCGAAGACCCAA
CATGAACACTGTTCTGAAAGCTTGCAGTCTGAGTTGAATATCATAATCAGTTAAAACTTTCA
ACAACGGATCTCTTGGTTCCGGCATCGATGAAGAACGCAGCGAAATGCGATAACTAATGTG
AATTGCAGAATTCAGTGAATCATCGAGTCTTTGAACGCACATTGCGCCCCCTGGTATTCCGG
GGGGCATGCCTGTCCGAGCGTCATTGCTGCCCTCAAGCACGGCTTGTGTGTTGGGCCGCCGT
CCCCGCCTCCCCGGGGACGGGCCCGAAAGGCAGCGGCGGCACCGTGTCCGGTCCTCGAGCG
TATGGGGCTTTGTCACCCGCTCTGTAGGCCTGGCCGGCGCCAGCCGACGTACCAACCCTTTTT
CTTTAGGTTGACCTCGGATCAGGTAGGGATACCCGCTGAACTTAAGCATATCAATAAGCGG
AGGAAAAGGGGAGGGGACAGAGAGGGGGAA

>BSNB_0253_Penicillium_sp

ATGAGGCCTCCGGGTCCAACCTCCCACCCGTGTTTATTCGTACCTTGTTGTTCGGCAGGCCCG
CCTCACGGCCGCCGGGGGGCTTCTGCCCCCGGGCCCGCGCCTGCCGGAGACAATTCTGAAC
GCTGTCTGAAGAATGCAGTCTGAGCGATTAGCAAAATTAGTTAAAACTTTCAACAACGGATC
TCTTGGTTCCGGCATCGATGAAGAACGCAGCGAAATGCGATAATTAATGTGAATTGCAGAA
TTCAGTGAATCATCGAGTCTTTGAACGCACATTGCGCCCCCTGGTATTCCGGGGGGCATGCC
TGTCCGAGCGTCATTGCTGCCCTCAAGCCCGGCTTGTGTGTTGGGCCCTGTTCCCCCGGGAAC
AGGCCCGAAAGGCAGTGGCGGCACCGCGTCCGATCCTCGAGCGTATGGGGCTTTGTCACCC
GCTCTGTAGGCCCGGCCGGCGCCCGTCGACCCCCCAACCTTTTTTTTTTTTKTCCANGNNAC
CCTCGAACAGGGGGGGGACCCCCCCTTAAATTTAGGCATTTCCTTAAGGGGGAAGAA

>BSNB_0263_Paecilomyces_formosus

GGAGGCCCACCTCCCATCCGTGTTTGAACTACACCTGTTGCTTCGGCGGGCCCGCCGTGGTT
CACGCCCGGCCCGCCGGGGGGCCTTGTGCCCCCGGGCCCGCGCCCGCCGAAGACCCCTCTA
ACGCTGCCCTGAAGGTTACCGTCTGAGTATAAAATCAATCGTTAAAACTTTCAACAACGGAT
CTCTTGGTTCCGGCATCGATGAAGAACGCATCGAAATGCGATAAGTAATGTGAATTGCAGA
ATTCCGTGAATCATCGAATCTTTGAACGCACATTGCGCCCCCTGGCATTCCGGGGGGCATGC
CTGTCCGAGCGTCATTGCTAACCCTCCAGCCCGGCTGGTGTGTTGGGTCGACGTCCCCCCCG
GGGGACGGGCCCGAAAGGCAGGGGCGGCGCCGCGTCCAATCCTCKAGCGTATGGGGCTTTG
TCACGCCCTCTGGTATGGTCGGCCGACTGGTCATTCAACTATCTCGCAGTCACCTATT

>BSNB_0266_Rhizomucor_variabilis

NNNNNNNNNNNNNNNNNNNNNNNNNNNNCTGTTTTACTGTTTAGCGTTTTTGAGGGATTG
CCTAAAGATTATAGGGATAGGTCTTTAGGATGTTAACCTAGCTAAAGTCAGGCTTAGGCCTG
GTATCCTAATTCATTATTTACCAAAAGAATTCAGAATTAATTATTGTAACATAAGCGTAAAA
AACTTATAAAACAACTTTTAACAACGGATCTCTTGGTTCTCGCATCGATGAAGAACGTAGCA
AAGTGCGATAACTAGTGTGAATTGCATATTCAGTGAATCATCGAGTCTTTGAACGCATCTTG
CGCTCAATGGTATTCCATTGAGCACGCCTGTTTCAGTATCAACAACAACCCACATCCACAAT
TTTGTTGTGAATGGAAATGAGAGTAATCGACGTTAAAATTGAACTCTTTAAAATTATTAGGC
CTGAACTATTGTTCTTTTAGCCTGAACATTTTTTTTAATATAAAGGAATGCTCTAGTTATTAAG
ACTGTCTTGGGGGCCTCCCAAATAAATCATTTTTTAAACTTGATCTGAAATCAGGTGGGATTA
CCCGCTGAACTTAAGCATATCAATAAGCGGAAGGAA

>BSNB_0283_Acrodictys_sp

GATCCGAGGTCAAACCACTATGCGTTTGGGGTGTTTAACGGCCAGCGCGCGCCGGGCGCCC
CTGAGCGAGAGGAGAACTACTGCGCTCGGAGGCTCGGCGAGCCCGCCACTGCATTTCGGGG
CCTACAGACGCTGTAGAGATGCCCCAACACCAAGCCGCGGGGGGCTTGAGGGTCGAAATG
ACGCTCGAACAGGCATGCCCGCCAGAATGCTGGCGGGCGCAATGTGCGTTCAAAGATTCGA
TGATTCACGGAATTCTGCAATTCACACTACGTATCGCATTTCGCTGCGTTCTTCATCGATGCT
GGAACCAAGAGATCCGTTGTTGAAAGTTTTGACTGATTTGTATGCTATACTCAGAAGACCAC
TGTACAGACGAGCTTCGGTTCTCCTCCGGCGGGCACCTCGCGGCCGCGGCCCCGCCCTGGGG
CCGCCCGCCGAAGCAACGGGACGGGTAGGTTCACAAAGGGTTTGGGAGTCTTGCAACTCGG
TAATGATCCCTCCGCTGGTTCACCAACGGAGACCTTGTTACGACTTCTCCT

>BSNB_0284_Ochroconis_sp

CCGTAGGTGAACCTGCGGAAGGATCATTACTGAGTGGCGTGGCGAGAATTGCGCTCGGAGA
CGAGACGTTCTCGGCCAGGTTACCCGACCTTTTGTCATTTGCTGGTTGGTGGAGTGGGGGTTG
GCTTCCGTTTCGGGACAGGTCTGAACGTCTTAGGGCGGGGAGGACCAGGTCGTCGGAGCGG
GATCCGCCCAACACGATGCCGACACTGTATTTCGGCGGAAGAGGTGGTCGTCGAGCTGCCC
GCCGCCGGTGGTATTTTCCAACTCCTTTTTTTTAATATTGTGAGAAGCTGAGAAGCGTCTGAG
GAAAAAACGATAAAATGAAAAAGCAAAACTTTCAACAACGGATCTCTTGGCTCTGGCAACG
ATGAAGAACGCAGCGAAACGCGAAAGGTAATGCGAATTGCAGAATCAGTGAGTCATCGAA
TCTTTGAACGCACATTGCGCCTCTTGGTATTCCAGGAGGCACGCCTGTTCGAGCGCCATCAC
AAACTCCAAGCGTGGCTTGATGTTGGACGGTGTCGGGAGGAGACTCCTGACACGTCTGGAA
CGCGTGGGCGCCGCCGCCAGACCACGAGCGTAGCAAGACGAAACAGTCGATCGTTCGGATT
GGGAGCTGGCGGTGGCCGTCTGGGGGGTGGCCTCTTGTTTTCCTTCCTGTGGGAGGGGGGAC
GGGAGGACCGCCGAACGTCAAAGTTTGGCCTCGGATCAGGC

>BSNB_0285_Ochroconis_sp

AGTTCAGAAATTTTGTCCGAAGACGTTAGAAGCGCGAACACTAGAATACCCTCCACAGCAA
CGCAGATAATTATCACGCTGAAGCGGCTGGTAACGTTCGCACTAATGCATTTCAGAGGAGCC
GACTACGAGAGCCGGCACGACCTCCAAGTCCAAGCCTTCGTCAATAAAGCCGAAGGTTGAG
AATTCCATGAGACTCAAACAGGCATGCTCCTCGGAATACCAAGGAGCGCAAGGTGCGTTCA
AAGATTCGATGATTCACTGAATTCTGCAATTCACATTACTTATCGCATTTCGCTGCGTTCTTCA
TCGATGCGAGAGCCAAGAGATCCGTTGCTGAAAGTTGTATATAAATTGCGTTATAGCAAAGT
ATGACATTCTAAAACTGAATCGTTTGTAGTAAAGCATAAGCCCGACACCTACAAGTGCGCG
AACGCACCCACAAGCCGGCCTATGAAAAGTGCACAGAAGTTGAGAGTGGATGAGACAGGC
GTGCACATGCCCTTGCGAGCCAGCAGACAACCCGTTCAAAACTCGATAATGATCCTTCCGCA
GGTTCACCTACGGAAACCTTGTTACGAACTTCTCCT
>BSNB_0287_Sordariomycetes_sp

TGATCCGAGGTCAACCTTGAGATAGAGGGGTTTTACGGGCGGAACGCCCCGGGACGCCGCG
AGCGAGGTGTATTACTGCGCTACGGGTCCAGGCGCGCCCGCCACTTCTTTTCAGGGCCCGCG
GCGGACGCGGGGCCCCAACACCGAGCAAGGCTCGAGGGTTGAAATGACGCTCGAACAGGC
ATGCCCGCCGGAATGCCGGCGGGCGCAATGTGCGTTCAAAGATTCGATGATTCGCTGAGTTC
TGCAATTCACATTACTTATCGCATTTCGCTGCGTTCTTCATCGATGCCAGAACCAAGAGATCC
GTTGTTGAAAGTTTTGACTCATTTAGTGTCTTCTCAGAGGGGCCTAAATCGCAAGAGTTGTGC
TGCGCTGCCGCCGGCGGGCGCCCTTGCGGGCTGCGGCCCCTCCCCGGGGCCGACCGCCAAA
ACAACGAAC

>BSNB_0289_Paramicrothyrium_sp

GATCCGAGGTCAACCTTAGGAGTTGATGGGGGTTTAACGGCCGGCATCCGCCGCGCGCGCC
AGGGCGAGGTATGTTACTGCGCCCGGTGCGACGGCGGGTCCGCCACTGCTTTTCGGGGCCCA
CGGCGGCCGTGGGGCCCCAACACCAAGCGGTGCTTGGGGGTCGAAATGACGCTCGGACAG
GCATGCCCGCCGGAGTGCCGGCGGGCGCAATGTGCGTTCAAAGATTCGATGATTCACTGAA
TTCTGCAATTCACATTACTTATCGCATTTCGCTGCGTTCTTCATCGATGCCAGAACCAAGAGA
TCCGTTGTTGAAAGTTTTGACGTTTGATAAACGCTCAGATGTGCCACTGTAAAGACAATAGTT
TTGGGGCCGCCGGCGGGCGCCCCGGGGGGCGCGCCGCCGAGGCAACGGGGTTCGTTCGCGA
CGGTTTTGGAGAGCAGAAAACTCTGTAATGATCCCTCCGCTGGTTCACCAACGGAGACCT

>BSNB_0290_Colletotrichium_gloeosporioides

CTGATCCGAGGTCAACCTTTGGAAAATTGGGGGTTTTACGGCAAGAGTCCCTCCGGATCCC
AGTGCGAGACGTAAAGTTACTACGCAAAGGAGGCTCCGGGAGGGTCCGCCACTACCTTTGA
GGGCCTACATCAGCTGTAGGGCCCCAACACCAAGCAGAGCTTGAGGGTTGAAATGACGCTC
GAACAGGCATGCCCGCCAGAATGCTGGCGGGCGCAATGTGCGTTCAAAGATTCGATGATTC
ACTGAATTCTGCAATTCACATTACTTATCGCATTTCGCTGCGTTCTTCATCGATGCCAGAACC
AAGAGATCCGTTGTTAAAAGTTTTGATTATTTGCTTGTACCACTCAGAAGAAACGTCGTTAA
ATCAGAGTTTGGTTATCCTCCGGCGGGCGCCGACCCGCCCGGGGGGGCGGGAGGCCGGGAG
GGTCACGGGGACCCTACCCGCCGAAGCAACAGTTGTAGGTATGTTCACAAAGGGTTGTAGA
GCGTAAACTCAGTAATGATCCCTCCGCTGGTTCACCAACGGAGACCTTGTTA

>BSNB_0292_Apioclypea_sp

CCTGATCCGAGGTCAACCCAGTAAAAAAGTTGGGGGTTTAGCGGCGAGGGCCACGGCACCT
ACAGAAGCGAGAGGTATATTACTGCGCTCAGAGGTAGAACCGTCACTCCGCCGGTGAATTT
GAGGAGCTACAGGGTAACAGCTACAGGGTAGCTCCCGGGTAGTCTCCCAACGCCAAGACTA
GGTCTTGAGTGGTACTAATGACGCTCGAACAGGCATGCCCTCCAGAATGCTGGGGGGCGCA
ATGTGCGTTCAAAGATTCGATGATTCACTGAATTCTGCAATTCACATTACTTATCGCATTTCG
CTGCGTTCTTCATCGATGCCAGAACCAAGAGATCCGTTGTTAAAGGTTTTGACTTATTTTCAT
AAGACTCTCAGATGATAATAATACAAATAGTTTGGTTTTCCACCGGCGGTCCGCCCGCTACA
GGGTAAGCTGCAGGGTAAGACCGCCGAGGCAACGTAAGGTATAGGTTCACAAATGGTTTAT
ACAGGAGTGTTTTAATACTCTGTAATGATCCCTCCGCTGGTTCACCAACGGAGACCTTGTTAC
GAACTTCTCCT

>BSNB_0293_Penicillium_sp

AAGCCCCATACGCTTGAGGACCGGACGCGGTGCCGCCGCTGCCTTTCGGGCCCGTCCCCCGG
AAGGAGGACGGAGCCCAACACACAAGCCGTGCTTGAGGGCAGCAATGACGCTCGGACAGG
CATGCCCTCCGGAATACCAGAGGGCGCAATGTGCGTTCAAAGACTCGATGATTCACTGAATT
CTGCAATTCACATTACGTATCGCATTTCGCTGCGTTCTTCATCGATGCCGGAACCAAGAGATC
CGTTGTTGAAAGTTTTAAATAATTTATATTTAATCTCAGACTACAATCTTCAGACAGAGTTCT
AAGGTGTCTTCGGCGAGCGCGGACCCGGGGACAGACGTCCCCCGGCAGCCAAAAGGCAGG
CTCGCCGAAGCAACAAGGTAAAATAAACACGGGTGGGAGGTTGGACCCAGAGGGCCCTCA
CTCGGTAATGATCCTTCCGCAGGTTCACCTACGGAAACCTTGTTACGACTTCTCCT

>BSNB_0295_Chaetomium_sp

CCTGATCCGAGGTCAACCTTGTAAGTTAGGGGGTTTTACGGCCGGTACGCACCGGGACGCCG
CGAGCGAGGTTAAGCTACTGCGCTACGGGTCCAGGCGCGCCCGCCACTTCTTTTCAGGGCCC
GCGGCAGCCGCGGGGCCCCAACACCGAGCAGGGCTCGAGGGTTGAAATGACGCTCGAACA
GGCATGCCCGCCGGAATGCCGGCGGGCGCAATGTGCGTTCAAAGATTCGATGATTCGCTGA
GTTCTGCAATTCACATTACTTATCGCATTTCGCTGCGTTCTTCATCGATGCCAGAACCAAGAG

ATCCGTTGTTGAAAGTTTTGACTCATTTAGTGTCTTCTCAGAGGGGCCTAAATTGCAAGAGTG
TAAAGTGCCGCCGGCGGGCGCCCTTGCGGGCTGCGGCCCCTCCCCGGGGCCGCCCACCGAA
GCAACGTCTTCAGTACAAGTTCACGGGTGTGTATAGAGAGAATGGAACTCTATAATGATCCC
TCCGCTGGTTCACCAACGGAGACCTTGTTACA

>BSNB_0296_Chaetomium_sp

AGTTCAGAAATTTTGTCCGAAGACGTTAGAAGCGCGAACACTAGAATACCCTCCACAGCAA
CGCAGATAATTATCACGCTGAAGCGGCTGGTAACGTTCGCACTAATGCATTTCAGAGGAGCC
GACTACGAGAGCCGGCACGACCTCCAAGTCCAAGCCTTCGTCAATAAAGCCGAAGGTTGAG
AATTCCATGAGACTCAAACAGGCATGCTCCTCGGAATACCAAGGAGCGCAAGGTGCGTTCA
AAGATTCGATGATTCACTGAATTCTGCAATTCACATTACTTATCGCATTTCGCTGCGTTCTTCA
TCGATGCGAGAGCCAAGAGATCCGTTGCTGAAAGTTGTATATAAATTGCGTTATAGCAAAGT
ATGACATTCTAAAACTGAATCGTTTGTAGTAAAGCATAAGCCCGACACCTACAAGTGCGCG
AACGCACCCACAAGCCGGCCTATGAAAAGTGCACAGAAGTTGAGAGTGGATGAGACAGGC
GTGCACATGCCCTTGCGAGCCAGCAGACAACCCGTTCAAAACTCGATAATGATCCTTCCGCA
GGTTCACCTACGGAAACCTTGTTACGAACTTCTCCT

>BSNB_0298_Penicillium_sp

CCCCATACGCTCGAGGACCGGACGCGGTGCCGCCGCTGCCTTTCGGGCCCGTCCCCCGGAAT
CGGAGGACGGGGCCCAACACACAAGCCGGGCTTGAGGGCAGCAATGACGCTCGGACAGGC
ATGCCCCCCGGAATACCAGGGGGCGCAATGTGCGTTCAAAGACTCGATGATTCACTGAATTT
GCAATTCACATTACGTATCGCATTTCGCTGCGTTCTTCATCGATGCCGGAACCAAGAGATCC
GTTGTTGAAAGTTTTAAATAATTTATATTTTCACTCAGACGACAATCTTCAGGCAGAGTTCGG
GGGTGTCTTCGGCGGGCGCGGGCCCGGGGGCGTGAGCCCCCCGGCGGCCAGTTAAGGCGGG
CCCGCCGAAGCAACGAGGTAAATAAACACGGGTGGGAGGTTGGACCCAAAGGGCCCTCAC
TCGGTAATGATCCTTCCGCAGGTTCACCTACGTAAAACCTTGTTACGACTTCTCC

>BSNB_0299_Muscodor_sp

TTCTGCAATTCACATTACTTATCGCATTTCGCTGCGTTCTTCATCGATGCCAGAACCAACAGA
TCCGTTGTTGAAAGTTTTAACTTATTAAGTTTATGATTCGCAATTCCAAAGAAAACAGAGTTT
AGTTGGCCGCCGGCGGGGATGACTACCGGGTAACAGATGTACCTGAGACTGCAGGGAAACC
ACTAGGTGGTAATCCCCCGCAGGGTAGCCTCCGCCGCCGAAGCAACGAAGGTAAGTTCACA
TAGGGTTGGGAGTTTAGAAAACTCTGTAATGATCCCTCCGCTGGTTCACCAACGGAGACCTT
GTTACGACTTCTCCTTAATGTCCACCCAGCTGTGAGTACGACCGCCGAACCAGTCGCCCCAC
TGGCAAGCCAAGGCACGGAACGTTGAGGTTCACATTGTGGTTTTAAATAATTCAATGATCCC
TCCCGCAGGTTCACCTACGGAGACCTTGTTACGACTTCTCCC

>BSNB_0300_Xylaria_sp

TTAACTTATTTAGTTATATGTTCAGAATTCAATACTAAACAGAGTTTCGTGGGCCACCGGCAG
GCTTACCCGCGTCTCCCGGGTAGGCCCTACAGGGTAGGGCGCTACGAGGTAGGCGCGACCT
GCCGAGGCAACGTAAGGTATGTTCACATGGGTTTGGGAGTTATAGAACTCTTTAATGATCCC
TCCGCTGGTTCACCAACGGAGA
>BSNB_0301_Albonectria_rigidiuscula

GATCCGAGGTCAACATTCAGAAGTTGGGGGTTTAACGGCTTGGCCGCGCCGCGTTCCAGTTG
CGAGGTGTTAGCTACTACGCAATGGAGGCTACAGCGAGACCGCCACTAGATTTGGGGGACG
GCGACTATCGCCGATCCCCAACACCAAGCCCGGGGGCTTGAGGGTTGAAATGACGCTCGAA
CAGGCATGCCCGCCAGAATACTGGCGGGCGCAATGTGCGTTCAAAGATTCGATGATTCACT

GAATTCTGCAATTCACATTACTTATCGCATTTCGCTGCGTTCTTCATCGATGCCAGAACCAAG
AGATCCGTTGTTGAAAGTTTTGATTTATTTGTTTTGTTTTACTCAGAAGATCCACAAGAATAC
ATAGAGTTTGGGGTTCCTCTGGCAGCGAGCAGCGCCCGATTTCTCGGAGCACCGTCGTTGAG
TCTGCCGAGGCAAATTATAGGTATGTTCACAGGGGTTTGGGAGTTGTAAACTCGGTAATGAT
CCCTCCGCTGGTTCACCAACGGAGACCTTGTT

>BSNB_0302_Fusarium_sp

GATCCGAGGTCAACATTCAGAAGTTGGGGGTTTAACGGCTTGGCCGCGCCGCGTTCCAGTTG
CGAGGTGTTAGCTACTACGCAATGGAGGCTACAGCGAGACCGCCACTAGATTTGGGGGACG
GCGACTATCGCCGATCCCCAACACCAAGCCCGGGGGCTTGAGGGTTGAAATGACGCTCGAA
CAGGCATGCCCGCCAGAATACTGGCGGGCGCAATGTGCGTTCAAAGATTCGATGATTCACT
GAATTCTGCAATTCACATTACTTATCGCATTTCGCTGCGTTCTTCATCGATGCCAGAACCAAG
AGATCCGTTGTTGAAAGTTTTGATTTATTTGTTTTGTTTTACTCAGAAGATCCACAAGAATAC
ATAGAGTTTGGGGTTCCTCTGGCAGCGAGCAGCGCCCGATTTCTCGGAGCACCGTCGTTGAG
TCTGCCGAGGCAAATTATAGGTATGTTCACAGGGGTTTGGGAGTTGTAAACTCGGTAATGAT
CCCTCCGCTGGTTCACCAACGGAGACCTTGTT

>BSNB_0303_Fusarium_sp

GATCCGAGGTCAACATTCAGAAGTTGGGGGTTTAACGGCTTGGCCGCGCCGCGTTCCAGTTG
CGAGGTGTTAGCTACTACGCAATGGAGGCTACAGCGAGACCGCCACTAGATTTGGGGGACG
GCGACTATCGCCGATCCCCAACACCAAGCCCGGGGGCTTGAGGGTTGAAATGACGCTCGAA
CAGGCATGCCCGCCAGAATACTGGCGGGCGCAATGTGCGTTCAAAGATTCGATGATTCACT
GAATTCTGCAATTCACATTACTTATCGCATTTCGCTGCGTTCTTCATCGATGCCAGAACCAAG
AGATCCGTTGTTGAAAGTTTTGATTTATTTGTTTTGTTTTACTCAGAAGATCCACAAGAATAC
ATAGAGTTTGGGGTTCCTCTGGCAGCGAGCAGCGCCCGATTTCTCGGAGCACCGTCGTTGAG
TCTGCCGAGGCAAATTATAGGTATGTTCACAGGGGTTTGGGAGTTGTAAACTCGGTAATGAT
CCCTCCGCTGGTTCACCAACGGAGACCTTGTT

>BSNB_0304_Sordariomycetes_sp

CCGAGGTCAAACTCGTGGGGTGCGCGCCCGGGGGGGGGCGCGCGGTTTGCTGGCCGGCCGG
CCGCCGGGCCCCGGAGCGAGATCGCTACTGCGCTCGGGGTGCGGCGGGGCCGCCACTGCAT
CTGGGGGCCAGCGGCGGGCGCTGGGCCCCGACACCAGGGCGCGCCTGGGGGGGTTGACAT
GACGCTCGGACGGGCGTGCCCGCCGGAATGCCGGCGGGCGCAATGTGCGTTCAAAGATTCG
ATGACTCACTGGATCTGCAATTCGCATTACGTATCGCATTTCGCTGCGTTCTTCATCGATGCC
AGAGCCAAGAGATCCGTTGCTGAAGGTTGTAGAAAGTTTGGGGGTCTGACAGGCCACACGG
GACAGAGTACAGGGGCTCCCGGCAGGGCGACCGCCCTGCCGGAGCAGCACGTTGGGTAGG
TTCGGAAGGGTGTCCAGGGCACAGAGGCGCTGTAACTTTAATGATCCCTCCGCTGGTTCACC
AACGGAGACCTTGT

>BSNB_0305_Chaetomium_sp

CTGATCCGAGGTCAACCTTGTAAGTTAGGGGGTTTTACGGCCGGTACGCACCGGGACGCCGC
GAGCGAGGTTAAGCTACTGCGCTACGGGTCCAGGCGCGCCCGCCACTTCTTTTCAGGGCCCG
CGGCAGCCGCGGGGCCCCAACACCGAGCAGGGCTCGAGGGTTGAAATGACGCTCGAACAG
GCATGCCCGCCGGAATGCCGGCGGGCGCAATGTGCGTTCAAAGATTCGATGATTCGCTGAG
TTCTGCAATTCACATTACTTATCGCATTTCGCTGCGTTCTTCATCGATGCCAGAACCAAGAGA
TCCGTTGTTGAAAGTTTTGACTCATTTAGTGTCTTCTCAGAGGGGCCTAAATTGCAAGAGTAT
AGAGTGCCGCCGGCGGGCGCCCTTGCGGGCTGCGGCCCCTCCCCGGGGCCGCCCACCGAAG

CAACGTCTTCAGTACAAGTTCACAGGTGTGTATAGAGAGAATAGAACTCTATAATGATCCCT
CCGCTGGTTCACCAACGGAG

>BSNB_0516_Oxydothis_sp

TTAACTTATTAAACAAGACGCTCAGATTTCCATAAATAACAGAGTTTAATGGTCCTCTGGCG
GAGCCGCCGGGTAGTGGGGACCCCGCCGAGGCAACAAAGGTATGTTCACATGGGTTTAGGA
CATAATGTCTTGTAATGATCCCTCCGCTGGTTCACCAACGGAGA
>BSNB_0520_Diaporthe_sp

AATTTCAGAAGTTGGGGGTTTAACGGCAGGGCGCCGCCAGGGCCTTCCAGAACGAGATATA
ACTACTACGCTCGGGGTCCTAGCGAGCTCGCCACTAGATTTCAGGGCCTGCTTCTCTCGAAG
CAGTGCCCCAACACCAAGCCAGGCTTGAGGGTTGAAATGACGCTCGAACAGGCATGCCCTC
CGGAATACCAGAGGGCGCAATGTGCGTTCAAAGATTCGATGATTCACTGAATTCTGCAATTC
ACATTACTTATCGCATTTCGCTGCGTTCTTCATCGATGCCAGAACCAAGAGATCCGTTGTTGA
AAGTTTTGATTCATTTATGTTTTTTACTCAGAGATTCACTAAGAAACAAGAGTTTGGTTGGC
CGCCGGCGGGCTGCTCCCCGTCTCCGGGGGGCCTCAGAAGAGAGGCCGGCCTGCGCCGAGG
CAACAGTAAGGTATAAGTTCACAAAGGGTTTCTGGGTGCGCCGGGGCGCGTTCCAGCAATG
ATCCCTCCGCTGGTTCACCAACGGAGACCTTGTTACGACTTCTCCT

>BSNB_0523_Stereum_hirsutum

CTGATTTGAGGTCAAAGTTCGATGAAAGCTGTCCTTTCGGACGGTTAGAAAGCGCGTCTCCA
CAGAAGCAAGCACACCACAGCGTAGATAATTATCACACCGAGGCGCACGTCGCAACAAGA
CGCACTAATGCATTTGAGAGGAGCCGAGCGGTGAAGCCCGGCAAAGCCTCCAAGTCCAATC
CACTACGTTCACAAAAGTGAAGAGGGTTGAGAATTTCACGACACTCAAACAGGTGTGCCCT
TCGGAATACCAAAGGGCGCAAGGTGCGTTCAAAGATTCGATGATTCACTGAATTCTGCAATT
CACATTACTTATCGCATTTCGCTGCGTTCTTCATCGATGCGAGAGCCAAGAGATCCGTTGTTG
AAAGTTGTATTAGATGCGTTTTATTACATCGAGTAGACATTCTTAAGACATACAAAGGGTGT
GTAAAGGGACGCGAGCCTCTTCGAGTTAACGAAGAGACCCCCGCGAAGGTGCACAGGTGTG
TGTGGATTAGTGAAAGGAGCGTGCACATGCCGTTTTTAAAGGCCAGCTACAACTCCAGTCAT
AAATTTCATTAATGATCCTTCCGCAGGTTCACCTACGGAAACCTTGTTACGACTTCTCCT

>BSNB_0526_Pseudopestalotiopsis_sp

TGATCCGAGGTCAACCACAAAAAATTGGGGGTTTAGCGGCTGGGAGCTGCAGCACCTAACA
AAAGCGAGAAATAATTTACTACGCTCAGAGGATACCACAGATCCGCCGTTGTATTTCAGGA
ATTACATAACTGTAAATTCCCAACACTAAGCTAGGCTTAAGGGTTGAAATGACGCTCGAAC
AGGCATGCCCACTAGAATACTAATGGGCGCAATGTGCGTTCAAAGATTCGATGATTCACTGA
ATTCTGCAATTCACATTACTTATCGCATTTCGCTGCGTTCTTCATCGATGCCAGAACCAAGAG
ATCCGTTGTTGAAAGTTTTGACTTATTAAAATAAGACGCTCAGATTACATAAAATAACAAGA
GTTTAGTAGTCCACCGGCAGTTGCTACAGGGTAACCTGTCTCCAGGTACCAGGTAACCTCTG
CCGAGGCAACAAAAGGTAAGTTCACATGGGTTGGGAGTTTAGAAAACTCTATAATGATCCC
TCCGCTGGTTCACCAACGGAGACCTTGT

>BSNB_0528_Endomelanconiopsis_endophytica

GGAGAAGTTCGTAACAAGGTTTCCGTAGGTGAACCTGCGGAAGGATCATTACCGAGTTCTA
GGGGTCTCCGGACCTCTTCTCTCACACCCTATGTGTATCTACCTCTGTTGCTTTGGCGGGCCG
CGGTCCTCCGCGGCCGGCCCCCTAACCGGGGCTGGCCAGCGCCCGCCAGAGGACTACCAAA
CTCCAGTCAGTAAACGTAGCTGTCTGATCAAAAGTTTAATAAACTAAAACTTTCAACAACGG
ATCTCTTGGTTCTGGCATCGATGAAGAACGCAGCGAAATGCGATAAGTAATGTGAATTGCAG

AATTCAGTGAATCATCGAATCTTTGAACGCACATTGCGCCCCTTGGTATTCCGAGGGGCATG
CCTGTTCGAGCGTCATTTCACCACTCAAGCTCTGCTTGGTATTGGGCGCCGTCCTTCACCGGA
CGCGCCTCAAAGACCTCGGCGGTGGCGTCTTGCCTCAAGCGTAGTAGAAAACACCTCGCTTT
GGAGGACGGGACGTTCGCTCGCCGGACGAACCTTCTGAATTTTCTCAAGGTTGACCTCGGAT
CAGGTA

>BSNB_0529_Colletotrichium_gloeosporioides

CTACCTGATCCGAGGTCAACCTTTGGAAAATTGGGGGGTTTTACGGCAAGAGTCCCTCCGGA
TCCCAGTGCGAGACGTAAAGTTACTACGCAAAGGAGGCTCCGGGAGGGTCCGCCACTACCT
TTGAGGGCCTACATCAGCTGTAGGGCCCCAACACCAAGCAGAGCTTGAGGGTTGAAATGAC
GCTCGAACAGGCATGCCCGCCAGAATGCTGGCGGGCGCAATGTGCGTTCAAAGATTCGATG
ATTCACTGAATTCTGCAATTCACATTACTTATCGCATTTCGCTGCGTTCTTCATCGATGCCAG
AACCAAGAGATCCGTTGTTAAAAGTTTTGATTATTTGCTTGTACCACTCAGAAGAAACGTCG
TTAAATCAGAGTTTGGTTATCCTCCGGCGGGCGCCGACCCGCCCGGAGGCGGGAGGCCGGG
AGGGTCGCGGAGACCCTACCCGCCGAAGCAACAGTTATAGGTATGTTCACAAAGGGTTATA
GAGCGTAAACTCAGTAATGATCCCTCCGCTGGTTCACCAACGGAGACCTTGTTACGACTTCT
CC

>BSNB_0530_Colletotrichium_gloeosporioides

CTACCTGATCCGAGGTCAACCTTTGGAAAATTGGGGGGTTTTACGGCAAGAGTCCCTCCGGA
TCCCAGTGCGAGACGTAAAGTTACTACGCAAAGGAGGCTCCGGGAGGGTCCGCCACTACCT
TTGAGGGCCTACATCAGCTGTAGGGCCCCAACACCAAGCAGAGCTTGAGGGTTGAAATGAC
GCTCGAACAGGCATGCCCGCCAGAATGCTGGCGGGCGCAATGTGCGTTCAAAGATTCGATG
ATTCACTGAATTCTGCAATTCACATTACTTATCGCATTTCGCTGCGTTCTTCATCGATGCCAG
AACCAAGAGATCCGTTGTTAAAAGTTTTGATTATTTGCTTGTACCACTCAGAAGAAACGTCG
TTAAATCAGAGTTTGGTTATCCTCCGGCGGGCGCCGACCCGCCCGGAGGCGGGAGGCCGGG
AGGGTCGCGGAGACCCTACCCGCCGAAGCAACAGTTATAGGTATGTTCACAAAGGGTTATA
GAGCGTAAACTCAGTAATGATCCCTCCGCTGGTTCACCAACGGAGACCTTGTTACGACTTCT
CC

>BSNB_0531_Stereum_hirsutum

CTGATTTGAGGTCAAAGTTCGATGAAAGCTGTCCTTTCGGACGGTTAGAAAGCGCGTCTCCA
CAGAAGCAAGCACACCACAGCGTAGATAATTATCACACCGAGGCGCACGTCGCAACAAGA
CGCACTAATGCATTTGAGAGGAGCCGAGCGGTGAAGCCCGGCAAAGCCTCCAAGTCCAATC
CACTACGTTCACAAAAGTGAAGAGGGTTGAGAATTTCACGACACTCAAACAGGTGTGCCCT
TCGGAATACCAAAGGGCGCAAGGTGCGTTCAAAGATTCGATGATTCACTGAATTCTGCAATT
CACATTACTTATCGCATTTCGCTGCGTTCTTCATCGATGCGAGAGCCAAGAGATCCGTTGTTG
AAAGTTGTATTAGATGCGTTTTATTACATCGAGTAGACATTCTTAAGACATACAAAGGGTGT
GTAAAGGGACGCGAGCCTCTTCGAGTTAACGAAGAGACCCCCGCGAAGGTGCACAGGTGTG
TGTGGATTAGTGAAAGGAGCGTGCACATGCCGTTTTTAAAGGCCAGCTACAACTCCAGTCAT
AAATTTCATTAATGATCCTTCCGCAGGTTCACCTACGGAAACCTTGTTACGACTTCTCCT

>BSNB_0532_Stereum_hirsutum

CTGATTTGAGGTCAAAGTTCGATGAAAGCTGTCCTTTCGGACGGTTAGAAAGCGCGTCTCCA
CAGAAGCAAGCACACCACAGCGTAGATAATTATCACACCGAGGCGCACGTCGCAACAAGA
CGCACTAATGCATTTGAGAGGAGCCGAGCGGTGAAGCCCGGCAAAGCCTCCAAGTCCAATC
CACTACGTTCACAAAAGTGAAGAGGGTTGAGAATTTCACGACACTCAAACAGGTGTGCCCT
TCGGAATACCAAAGGGCGCAAGGTGCGTTCAAAGATTCGATGATTCACTGAATTCTGCAATT

CACATTACTTATCGCATTTCGCTGCGTTCTTCATCGATGCGAGAGCCAAGAGATCCGTTGTTG
AAAGTTGTATTAGATGCGTTTTATTACATCGAGTAGACATTCTTAAGACATACAAAGGGTGT
GTAAAGGGACGCGAGCCTCTTCGAGTTAACGAAGAGACCCCCGCGAAGGTGCACAGGTGTG
TGTGGATTAGTGAAAGGAGCGTGCACATGCCGTTTTTAAAGGCCAGCTACAACTCCAGTCAT
AAATTTCATTAATGATCCTTCCGCAGGTTCACCTACGGAAACCTTGTTACGACTTCTCCT

>BSNB_0534_Nodulisporium_sp

CCTAATCCGAGGTCAACCACTAGAAAATAGGGGGTTTTACGGCTAGCAGCCAGGGCCACCA
CACAAGCGAGAGAGATTACTACGCTGAGAGTGTACCCTAACTCCGCCACTGGTTTTCAGGA
ACTACGCCGAAGCCGTAGACTCCCAACACTAAGCAACAGGGCTTAAGGGTTGAAATGACGC
TCGAATAGGCATGCCCACTAGAATACTAATGGGCGCAATGTGCGTTCAAAGATTCGATGATT
CACTGAATTCTGCAATTCACATTACTTATCGCATTTCGCTGCGTTCTTCATCGATGCCAGAAC
CAAGAGATCCGTTGTTGAAAGTTTTAACTTATTTCAGTTAAGTACTCAGAGATACAGCTGTA
AAAACAAGAGTTTAATGGTCCTGCGGCGGCCTTCACGCGGCTACAGGGTAGCTCCAGGGT
AGACACCTACAGGGTAGATGCCTCCAGGGCAGCTACAGGGTAACCGCAGCACGCGCCGAG
GAAACGACGGTAAGGTTCACAAAGGGTTTTGGAGTTTGGTAACTCATTAATGATCCCTCCGC
TGGTTCACCAACGGAGACCTTGTTACG

>BSNB_0535_Coriolopsis_rigida

AGAAGTCGTAACAAGGTCTCCGTTGGTGAACCAGCGGAGGGATCATTGCTGGAACGCGCCC
CTGGCGCACCCAGAAACCCTTTGTGAACTTATACCTTACTGTTGCCTCGGCGCAGGCCGGCC
CCTATGGGGTCCCTCGGAGACGAGGAGCAGCCGGCCGGTGGCCAAGTTAACTCTGTTTTTAC
ACTGAAACTCTGAGTATAAAACATAAATGAATCAAAACTTTCAACAACGGATCTCTTGGTTC
TGGCATCGATGAAGAACGCAGCGAAATGCGATAAGTAATGTGAATTGCAGAATTCAGTGAA
TCATCGAATCTTTGAACGCACATTGCGCCCTCTGGTATTCCGGAGGGCATGCCTGTTCGAGC
GTCATTTCAACCCTCAAGCCTGGCTTGGTGTTGGGGCACTGCTTTACCCACAAGCAGGCCCT
GAAATATAGTGGCGAGCTCGCCAGGACTCCGAGCGTAGTAGTTAAACCCTCGCTTTGGAAG
GCCTGGCGGTGCCCTGCCGTTAAACCCCAACTTTTGAAAATTGACCTCGGATCAG

>BSNB_0536_Colletotrichum_gloeosporioides

CTACCTGATCCGAGGTCAACCTTTGGAAAATTGGGGGGTTTTACGGCAAGAGTCCCTCCGGA
TCCCAGTGCGAGACGTAAAGTTACTACGCAAAGGAGGCTCCGGGAGGGTCCGCCACTACCT
TTGAGGGCCTACATCAGCTGTAGGGCCCCAACACCAAGCAGAGCTTGAGGGTTGAAATGAC
GCTCGAACAGGCATGCCCGCCAGAATGCTGGCGGGCGCAATGTGCGTTCAAAGATTCGATG
ATTCACTGAATTCTGCAATTCACATTACTTATCGCATTTCGCTGCGTTCTTCATCGATGCCAG
AACCAAGAGATCCGTTGTTAAAAGTTTTGATTATTTGCTTGTACCACTCAGAAGAAACGTCG
TTAAATCAGAGTTTGGTTATCCTCCGGCGGGCGCCGACCCGCCCGGAGGCGGGAGGCCGGG
AGGGTCGCGGAGACCCTACCCGCCGAAGCAACAGTTATAGGTATGTTCACAAAGGGTTATA
GAGCGTAAACTCAGTAATGATCCCTCCGCTGGTTCACCAACGGAGACCTTGTTACGACTTCT
CC

>BSNB_0537_Colletotrichum_gloeosporioides

CTACCTGATCCGAGGTCAACCTTTGGAAAATTGGGGGGTTTTACGGCAAGAGTCCCTCCGGA
TCCCAGTGCGAGACGTAAAGTTACTACGCAAAGGAGGCTCCGGGAGGGTCCGCCACTACCT
TTGAGGGCCTACATCAGCTGTAGGGCCCCAACACCAAGCAGAGCTTGAGGGTTGAAATGAC
GCTCGAACAGGCATGCCCGCCAGAATGCTGGCGGGCGCAATGTGCGTTCAAAGATTCGATG
ATTCACTGAATTCTGCAATTCACATTACTTATCGCATTTCGCTGCGTTCTTCATCGATGCCAG
AACCAAGAGATCCGTTGTTAAAAGTTTTGATTATTTGCTTGTACCACTCAGAAGAAACGTCG

TTAAATCAGAGTTTGGTTATCCTCCGGCGGGCGCCGACCCGCCCGGAGGCGGGAGGCCGGG
AGGGTCGCGGAGACCCTACCCGCCGAAGCAACAGTTATAGGTATGTTCACAAAGGGTTATA
GAGCGTAAACTCAGTAATGATCCCTCCGCTGGTTCACCAACGGAGACCTTGTTACGACTTCT
CC

>BSNB_0538_Colletotrichum_gloeosporioides

CTGATCCGAGGTCAACCTTTGGAAAATTGGGGGTTTTACGGCAAGAGTCCCTCCGGATCCC
AGTGCGAGACGTAAAGTTACTACGCAAAGGAGGCTCCGGGAGGGTCCGCCACTACCTTTGA
GGGCCTACATCAGCTGTAGGGCCCCAACACCAAGCAGAGCTTGAGGGTTGAAATGACGCTC
GAACAGGCATGCCCGCCAGAATGCTGGCGGGCGCAATGTGCGTTCAAAGATTCGATGATTC
ACTGAATTCTGCAATTCACATTACTTATCGCATTTCGCTGCGTTCTTCATCGATGCCAGAACC
AAGAGATCCGTTGTTAAAAGTTTTGATTATTTGCTTGTACCACTCAGAAGAAACGTCGTTAA
ATCAGAGTTTGGTTATCCTCCGGCGGGCGCCGACCCGCCCGGGGGGGCGGGAGGCCGGGAG
GGTCACGGGGACCCTACCCGCCGAAGCAACAGTTGTAGGTATGTTCACAAAGGGTTGTAGA
GCGTAAACTCAGTAATGATCCCTCCGCTGGTTCACCAACGGAGACCTTGTTA

>BSNB_0539_Nemania_sp

TGATCCGAGGTCAACCTTTAAAAATTGGGGTGTTTTACGGCAGGGGACCGGTCCAGCTACAG
GCGAGGTGAAAATCTACTACGTCTGGAGTGTGAACCGACCACGCCACTAACTTTAGGGAGC
TACGCTTCCGTAGGCTCCCAACGCTAAGCAACAGGGGCTTAAGGGTTGAAATGACGCTCGA
ACAGGCATGCCCACTAGAATACTAATGGGCGCAATGTGCGTTCAAAGATTCGATGATTCACT
GAATTCTGCAATTCACATTACTTATCGCATTTCGCTGCGTTCTTCATCGATGCCAGAACCAAG
AGATCCGTTGTTGAAAGTTTTAACTTATTTAGTTATACGTTCAGAATTCAATGCTAAACAGAG
TTTTGCGAGCCGCCGGCAGGTTCCCCCAGCGCCCCCCTAAAGCAAGCCCTACAATGTAGGG
GCGCTACAGGGTAGGCCAGGGCCTGCCGAGGCAACAGAAGGTATGTTCACAGGGGTTTGGG
AGTTGTAAAAACTCTTTAATGATCCCTCCGCTGGTTCACCAACGGAGACCT

>BSNB_0540_Colletotrichum_gloeosporioides

CTACCTGATCCGAGGTCAACCTTTGGAAAATTGGGGGTTTTACGGCAAGAGTCCCTCCGGA
TCCCAGTGCGAGACGTAAAGTTACTACGCAAAGGAGGCTCCGGGAGGGTCCGCCACTACCT
TTGAGGGCCTACATCAGCTGTAGGGCCCCAACACCAAGCAGAGCTTGAGGGTTGAAATGAC
GCTCGAACAGGCATGCCCGCCAGAATGCTGGCGGGCGCAATGTGCGTTCAAAGATTCGATG
ATTCACTGAATTCTGCAATTCACATTACTTATCGCATTTCGCTGCGTTCTTCATCGATGCCAG
AACCAAGAGATCCGTTGTTAAAAGTTTTGATTATTTGCTTGTACCACTCAGAAGAAACGTCG
TTAAATCAGAGTTTGGTTATCCTCCGGCGGGCGCCGACCCGCCCGGAGGCGGGAGGCCGGG
AGGGTCGCGGAGACCCTACCCGCCGAAGCAACAGTTATAGGTATGTTCACAAAGGGTTATA
GAGCGTAAACTCAGTAATGATCCCTCCGCTGGTTCACCAACGGAGACCTTGTTACGACTTCT
CC

>BSNB_0541_Diaporthe_sp

TACCTGATCCGAGGTCAAAATTTCAGAAGTTGGGGGTTTAACGGCAGGGCGCCGCCAGGGC
CTTCCAGAACGAGATATAACTACTACGCTCGGGGTCCTAGCGAGCTCGCCACTAGATTTCAA
GGCCTGCCCTTTTACAGGCAGTGCCCCAACACCAAGCCAGGCTTGAGGGTTGAAATGACGC
TCGAACAGGCATGCCCTCCGGAATACCAGAGGGCGCAATGTGCGTTCAAAGATTCGATGAT
TCACTGAATTCTGCAATTCACATTACTTATCGCATTTCGCTGCGTTCTTCATCGATGCCAGAA
CCAAGAGATCCGTTGTTGAAAGTTTTGATTCATTTATGTTTTTTACTCAGAGATTCACTAAG
AAACAAGAGTTTGGTTGGCCGCCGGCGGGCTGCTCCCCGTTTCCGGGGGGCCTCGAAGAGA
GGCCGGCCTTCGCCGAGGCAACAATAGGTATAAGTTCACAAAGGGTTTCTGGGTGCGCCGA

AGCGCGTTCCAGCAATGATCCCTCCGCTGGTTCACCAACGGAGACCTTGTTACGACCTTCTC
C

>BSNB_0546_Clonostachys_sp

ACTTCGCAGAGGAGGCCACGACGGGTCCGCCACTAGATTTAGGGGCCGGCCGTCCCTCGCG
GGCTTTGGCCGATCCCCAACACCACGCCCTAGGGGCATGAGGGTTGAAATGACGCTCAGAC
AGGCATGCCCGCCAGAATACTGGCGGGCGCAATGTGCGTTCAAAGATTCGATGATTCACTG
AATTCTGCAATTCACATTACTTATCGCATTTCGCTGCGTTCTTCATCGATGCCAGAACCAAGA
GATCCGTTGTTGAAAGTTTTTATTTATTTGTAAAAACTACTCAGAAGATTCCAAAATAAAAC
AAGAATTAAGTTTCCTAGGCGGGCGCCTGATCCGGGGCACACGAGGCGCCCGGGGCAATCC
CGCCGAAGCAACAGTAGGTATGTTCACATGGGTTTGGGAGTTGTAAACTCGGTAATGATCCC
TCCGCTGGTTCACCAACGGAGACCTTGTTACGACTTCTCCT

>BSNB_0548_Stereum_hirsutum

AGGAGAAGTCGTAACAAGGTTTCCGTAGGTGAACCTGCGGAAGGATCATTAATGAAATTTA
TGACTGGAGTTGTAGCTGGCCTTTAAAAACGGCATGTGCACGCTCCTTTCACTAATCCACAC
ACACCTGTGCACCTTCGCGGGGGTCTCTTCGTTAACTCGAAGAGGCTCGCGTCCCTTTACAC
ACCCTTTGTATGTCTTAAGAATGTCTACTCGATGTAATAAAACGCATCTAATACAACTTTCAA
CAACGGATCTCTTGGCTCTCGCATCGATGAAGAACGCAGCGAAATGCGATAAGTAATGTGA
ATTGCAGAATTCAGTGAATCATCGAATCTTTGAACGCACCTTGCGCCCTTTGGTATTCCGAAG
GGCACACCTGTTTGAGTGTCGTGAAATTCTCAACCCTCTTCACTTTTGTGAACGTAGTGGATT
GGACTTGGAGGCTTTGCCGGGCTTCACCGCTCGGCTCCTCTCAAATGCATTAGTGCGTCTTGT
TGCGACGTGCGCCTCGGTGTGATAATTATCTACGCTGTGGTGTGCTTGCTTCTGTGGAGACGC
GCTTTCTAACCGTCCGAAAGGACAGCTTTCATCGAACTTTGACCTCAAATCAG

>BSNB_0549_Colletotrichum_sp

CTACCTGATCCGAGGTCAACCTTTGGAAAATTGGGGGGTTTTACGGCAAGAGTCCCTCCGGA
TCCCAGTGCGAGACGTAAAGTTACTACGCAAAGGAGGCTCCGGGAGGGTCCGCCACTACCT
TTGAGGGCCTACATCAGCTGTAGGGCCCCAACACCAAGCAGAGCTTGAGGGTTGAAATGAC
GCTCGAACAGGCATGCCCGCCAGAATGCTGGCGGGCGCAATGTGCGTTCAAAGATTCGATG
ATTCACTGAATTCTGCAATTCACATTACTTATCGCATTTCGCTGCGTTCTTCATCGATGCCAG
AACCAAGAGATCCGTTGTTAAAAGTTTTGATTATTTGCTTGTACCACTCAGAAGAAACGTCG
TTAAATCAGAGTTTGGTTATCCTCCGGCGGGCGCCGACCCGCCCGGAGGCGGGAGGCCGGG
AGGGTCGCGGAGACCCTACCCGCCGAAGCAACAGTTATAGGTATGTTCACAAAGGGTTATA
GAGCGTAAACTCAGTAATGATCCCTCCGCTGGTTCACCAACGGAGACCTTGTTACGACTTCT
CC

>BSNB_0550_Stereum_sp

CTGATTTGAGGTCAAAGTTCGATGAAAGCTGTCCTTTCGGACGGTTAGAAAGCGCGTCTCCA
CAGAAGCAAGCACACCACAGCGTAGATAATTATCACACCGAGGCGCACGTCGCAACAAGA
CGCACTAATGCATTTGAGAGGAGCCGAGCGGTGAAGCCCGGCAAAGCCTCCAAGTCCAATC
CACTACGTTCACAAAAGTGAAGAGGGTTGAGAATTTCACGACACTCAAACAGGTGTGCCCT
TCGGAATACCAAAGGGCGCAAGGTGCGTTCAAAGATTCGATGATTCACTGAATTCTGCAATT
CACATTACTTATCGCATTTCGCTGCGTTCTTCATCGATGCGAGAGCCAAGAGATCCGTTGTTG
AAAGTTGTATTAGATGCGTTTTATTACATCGAGTAGACATTCTTAAGACATACAAAGGGTGT
GTAAAGGGACGCGAGCCTCTTCGAGTTAACGAAGAGACCCCCGCGAAGGTGCACAGGTGTG

TGTGGATTAGTGAAAGGAGCGTGCACATGCCGTTTTTAAAGGCCAGCTACAACTCCAGTCAT
AAATTTCATTAATGATCCTTCCGCAGGTTCACCTACGGAAACCTTGTTACGACTTCTCCT

>BSNB_0551_Colletotrichum_gloeosporioides

CCTGATCCGAGGTCAACCTTTGGAGATTTGGGGGTTTTACGGCAAGAGTCCCTCCGGATCCC
AGTGCGAGACGTAAAGTTACTACGCAAAGGAGGCTCCGGGAGGGTCCGCCACTACCTTTGA
GGGCCTACATCGGCTGTAGGGCCCCAACACCAAGCAGAGCTTGAGGGTTGAAATGACGCTC
GAACAGGCATGCCCGCCAGAATGCTGGCGGGCGCAATGTGCGTTCAAAGATTCGATGATTC
ACTGAATTCTGCAATTCACATTACTTATCGCATTTCGCTGCGTTCTTCATCGATGCCAGAACC
AAGAGATCCGTTGTTAAAAGTTTTGATTATTTGCTTGTACCACTCAGAAGAAACGTCGTTAA
ATCAGAGTTTGGTTATCCTCCGGCGGGCGCCGACCCGCCCGGGGGCGGGAGGCCGGGAGGG
TCACGGAGACCCTGCCCGCCGAAGCAACAGTTATAGGTATGTTCACAAAGGGTTGTAGAGC
GTAAACTCAGTAATGATCCCTCCGCTGGTTCACCAACGGAGACCTTGTT

>BSNB_0556_Neopestalotiopsis_sp

CTGATCCGAGGTCAACCACAAAAAATTGGGGGTTTAGCGGCTGGGAGTTATAGCACCTAAC
AAAAGCGAGAAAAAAATTACTACGCTCAGAGGATACTACAAATCCGCCGTTGTATTTCAGG
AACTACAACTCCTAAGAGAAGTAGATTCCCAACACTAAGCTAGGCTTAAGGGTTGAAATGA
CGCTCGAACAGGCATGCCCACTAGAATACTAATGGGCGCAATGTGCGTTCAAAGATTCGAT
GATTCACTGAATTCTGCAATTCACATTACTTATCGCATTTCGCTGCGTTCTTCATCGATGCCA
GAACCAAGAGATCCGTTGTTGAAAGTTTTGACTTATTAAAATAAGACGCTCAGATTACATAA
AATAACAAGAGTTTAATGGTCCACCGGCAGCAGCTATAAGAAGACCTATAACTTCTGCCGA
GGCAACAAAAGGTAAGTTCACATGGGTTGGGAGTTTAGAAAACTCTATAATGATCCCTCCG
CTGGTTCACCAACGGAGACCTTG

>BSNB_0557_Colletotrichum_vietnamense

TGATCCGAGGTCAACCTTTGGAAAATTGGGGGTTTTACGGCTAGAGTCCCTCCGAATCCCA
GTGCGAGACAAAAGTTACTACGCAAAGGAGGCTCCGAGAGGGTCCGCCACTACCTTTGAGG
GCCTACGTCAACCGTAGAGCCCCAACGCCAAGCAGTGCTTGAGGGTTGAAATGACGCTCGA
ACAGGCATGCCCGCCAGAATGCTGGCGGGCGCAATGTGCGTTCAAAGATTCGATGATTCAC
TGAATTCTGCAATTCACATTACTTATCGCATTTCGCTGCGTTCTTCATCGATGCCAGAACCAA
GAGATCCGTTGTTAAAAGTTTTGATTATTTGCTTGTGCCACTCAGAAGAAACGTCGTTAAATC
AGAGTTTGGTTATCCTCCGGCGGGCACCCCGACGAGCGGGGCCGGGAGCGGGCCGCAGTGG
CCGCGCTGCCCGCCGAAGCAACAGTTGTAGGTATGTTCACAAAGGGTTATAGAGCGGTAAC
TCAGTAATGATCCCTCCGCTGGTTCACCAACGGAGACCTTGTTACGAACTTCTC

>BSNB_0558_Stereum_hirsutum

CTGATTTGAGGTCAAAGTTCGATGAAAGCTGTCCTTTCGGACGGTTAGAAAGCGCGTCTCCA
CAGAAGCAAGCACACCACAGCGTAGATAATTATCACACCGAGGCGCACGTCGCAACAAGA
CGCACTAATGCATTTGAGAGGAGCCGAGCGGTGAAGCCCGGCAAAGCCTCCAAGTCCAATC
CACTACGTTCACAAAAGTGAAGAGGGTTGAGAATTTCACGACACTCAAACAGGTGTGCCCT
TCGGAATACCAAAGGGCGCAAGGTGCGTTCAAAGATTCGATGATTCACTGAATTCTGCAATT
CACATTACTTATCGCATTTCGCTGCGTTCTTCATCGATGCGAGAGCCAAGAGATCCGTTGTTG
AAAGTTGTATTAGATGCGTTTTATTACATCGAGTAGACATTCTTAAGACATACAAAGGGTGT
GTAAAGGGACGCGAGCCTCTTCGAGTTAACGAAGAGACCCCCGCGAAGGTGCACAGGTGTG
TGTGGATTAGTGAAAGGAGCGTGCACATGCCGTTTTTAAAGGCCAGCTACAACTCCAGTCAT
AAATTTCATTAATGATCCTTCCGCAGGTTCACCTACGGAAACCTTGTTACGACTTCTCCT

>BSNB_0559_Colletotrichum_gleoesporioides

CCTGATCCGAGGTCAACCTTTGGAGATTTGGGGGTTTTACGGCAAGAGTCCCTCCGGATCCC
AGTGCGAGACGTAAAGTTACTACGCAAAGGAGGCTCCGGGAGGGTCCGCCACTACCTTTGA
GGGCCTACATCGGCTGTAGGGCCCCAACACCAAGCAGAGCTTGAGGGTTGAAATGACGCTC
GAACAGGCATGCCCGCCAGAATGCTGGCGGGCGCAATGTGCGTTCAAAGATTCGATGATTC
ACTGAATTCTGCAATTCACATTACTTATCGCATTTCGCTGCGTTCTTCATCGATGCCAGAACC
AAGAGATCCGTTGTTAAAAGTTTTGATTATTTGCTTGTACCACTCAGAAGAAACGTCGTTAA
ATCAGAGTTTGGTTATCCTCCGGCGGGCGCCGACCCGCCCGGGGGCGGAGGCCGGGAGGG
TCACGGAGACCCTGCCCGCCGAAGCAACAGTTATAGGTATGTTCACAAAGGGTTGTAGAGC
GTAAACTCAGTAATGATCCCTCCGCTGGTTCACCAACGGAGACCTTGTT

>BSNB_0574_Colletotrichum_gleoesporioides

CCTGATCCGAGGTCAACCTTTGGAGATTTGGGGGTTTTACGGCAAGAGTCCCTCCGGATCCC
AGTGCGAGACGTAAAGTTACTACGCAAAGGAGGCTCCGGGAGGGTCCGCCACTACCTTTGA
GGGCCTACATCGGCTGTAGGGCCCCAACACCAAGCAGAGCTTGAGGGTTGAAATGACGCTC
GAACAGGCATGCCCGCCAGAATGCTGGCGGGCGCAATGTGCGTTCAAAGATTCGATGATTC
ACTGAATTCTGCAATTCACATTACTTATCGCATTTCGCTGCGTTCTTCATCGATGCCAGAACC
AAGAGATCCGTTGTTAAAAGTTTTGATTATTTGCTTGTACCACTCAGAAGAAACGTCGTTAA
ATCAGAGTTTGGTTATCCTCCGGCGGGCGCCGACCCGCCCGGGGGCGGAGGCCGGGAGGG
TCACGGAGACCCTGCCCGCCGAAGCAACAGTTATAGGTATGTTCACAAAGGGTTGTAGAGC
GTAAACTCAGTAATGATCCCTCCGCTGGTTCACCAACGGAGACCTTGTT

>BSNB_0575_Fusarium_concolor

GGTCAACATTCAGAAGTTGGGGTTTAACGGCGTGGCCGCGACGATTACCAGTAACGAGGTG
TAATTACTACGCTATGGAAGCTCGACGTGACCGCCAATAGATTTGGGGACCGCGGATTGCTC
CACGAATCCCAACACCAAGCTGTGCTTGAGGGTTGAAATGACGCTCGAACAGGCATGCCCG
CCAGAATACTGGCGGGCGCAATGTGCGTTCAAAGATTCGATGATTCACTGAATTCTGCAATT
CACATTACTTATCGCATTTTGCTGCGTTCTTCATCGATGCCAGAACCAAGAGATCCGTTGTTG
AAAGTTTTGATTTATTTGTTTGTTTTACTCAGAAGTTACACTAGAAACAGAGTTTAGGGTCCT
CTGGCGGGCCGTCCCGTTTTACCGGGGCCGGGCTGATCCGCCGAGGCAACAATAGGTATGTT
CACAGGGGTTTGGGAGTTGTAAACTCGGTAATGATCCCTCCGCTGGTTCACCAACGGAGACC
TTGTT

>BSNB_0578_Diaporthe_sp

GATCCGAGGTCAAAATTTCAGAAGTTGGGGGTTTAACGGCAGGGCGCCGCCAGGGCCTTCC
AGAACGAGATATAACTACTACGCTCGGGGTCCTAGCGAGCTCGCCACTAGATTTCAGGGCC
TGCTTTGTCTCCAAAGCAGTGCCCCAACACCAAGCCAGGCTTGAGGGTTGAAATGACGCTCG
AACAGGCATGCCCTCCGGAATACCAGAGGGCGCAATGTGCGTTCAAAGATTCGATGATTCA
CTGAATTCTGCAATTCACATTACTTATCGCATTTCGCTGCGTTCTTCATCGATGCCAGAACCA
AGAGATCCGTTGTTGAAAGTTTTGATTCATTTATGTTTTTTACTCAGAGATTCACCAAGAAAC
AAGAGTTTGGTTGGCCGCCGGCGGGCTGCTCCCCGTCTCCGGGGGGCCTCAGAAGAGGCCG
GCCTTCGCCGAGGCAACAATAAGGTATAAGTTCACAAAGGGTTTCTGGGTGCGCCGGGGCG
CGTTCCAGCAATGATCCCTCCGCTGGTTCACCAACGGAGACC

>BSNB_0579_Cladosporium_cladosporioides

CTGATCCGAGGTCACCTTAGAAATGGGGTTGTTTTACGGCGTAGCCTCCCGAACACCCTTTA

GCGAATAGTTTCCACAACGCTTAGGGGACAGAAGACCCAGCCGGTCGATTTGAGGCACGCG
GCGGACCGCGTTGCCCAATACCAAGCGAGGCTTGAGTGGTGAAATGACGCTCGAACAGGCA
TGCCCCCCGGAATACCAGGGGGCGCAATGTGCGTTCAAAGATTCGATGATTCACTGAATTCT
GCAATTCACATTACTTATCGCATTTCGCTGCGTTCTTCATCGATGCCAGAACCAAGAGATCCG
TTGTTAAAAGTTTTAATTTATTAATTAAGTTTACTCAGACTGCAAAGTTACGCAAGAGTTTGA
AGTGTCCACCCGGAGCCCCCGCCCGAAGGCAGGGTCGCCCCGGAGGCAACAGAGTCGGAC
AACAAAGGGTTATGAACATCCCGGTGGTTAGACCGGGGTCACTTGTAATGATCCCTCCGCAG
GTTCACCTACGGAGACCTTGTTACG

>BSNB_0580_Colletotrichum_gloeosporioides

CTACCTGATCCGAGGTCAACCTTTGGAAAATTGGGGGGTTTTACGGCAAGAGTCCCTCCGGA
TCCCAGTGCGAGACGTAAAGTTACTACGCAAAGGAGGCTCCGGGAGGGTCCGCCACTACCT
TTGAGGGCCTACATCAGCTGTAGGGCCCCAACACCAAGCAGAGCTTGAGGGTTGAAATGAC
GCTCGAACAGGCATGCCCGCCAGAATGCTGGCGGGCGCAATGTGCGTTCAAAGATTCGATG
ATTCACTGAATTCTGCAATTCACATTACTTATCGCATTTCGCTGCGTTCTTCATCGATGCCAG
AACCAAGAGATCCGTTGTTAAAAGTTTTGATTATTTGCTTGTACCACTCAGAAGAAACGTCG
TTAAATCAGAGTTTGGTTATCCTCCGGCGGGCGCCGACCCGCCCGGAGGCGGGAGGCCGGG
AGGGTCGCGGAGACCCTACCCGCCGAAGCAACAGTTATAGGTATGTTCACAAAGGGTTATA
GAGCGTAAACTCAGTAATGATCCCTCCGCTGGTTCACCAACGGAGACCTTGTTACGACTTCT
CC

>BSNB_0581_Fusarium_sp

TAGGAGAAGTCGTAACAAGGTCTCCGTTGGTGAACCAGCGGAGGGATCATTACCGAGTTTA
CAACTCCCAAACCCCTGTGAACATACCTATTGTTGCCTCGGCGGATCAGCCCGGCCCCGGTA
AAACGGGACGGCCCGCCAGAGGACCCTAAACTCTGTTTCTAGTGTAACTTCTGAGTAAAAC
AAACAAATAAATCAAAACTTTCAACAACGGATCTCTTGGTTCTGGCATCGATGAAGAACGC
AGCAAAATGCGATAAGTAATGTGAATTGCAGAATTCAGTGAATCATCGAATCTTTGAACGC
ACATTGCGCCCGCCAGTATTCTGGCGGGCATGCCTGTTCGAGCGTCATTTCAACCCTCAAGC
ACAGCTTGGTGTTGGGATTCGTGGAGCAATCCGCGGTCCCCAAATCTATTGGCGGTCACGTC
GAGCTTCCATAGCGTAGTAATTACACCTCGTTACTGGTAATCGTCGCGGCCACGCCGTTAAA
CCCCAACTTCTGAATGTTGACCTCGGATCAGGTA

>BSNB_0582_Endomelanconiopsis_endophytica

ACCTGATCCGAGGTCAACCTTGAGAAAATTCAGAAGGTTCGTCCGGCGAGCGAACGTCCCG
TCCTCCAAAGCGAGGTGTTTTCTACTACGCTTGAGGCAAGACGCCACCGCCGAGGTCTTTGA
GGCGCGTCCGGTGAAGGACGGCGCCCAATACCAAGCAGAGCTTGAGTGGTGAAATGACGCT
CGAACAGGCATGCCCCTCGGAATACCAAGGGGCGCAATGTGCGTTCAAAGATTCGATGATT
CACTGAATTCTGCAATTCACATTACTTATCGCATTTCGCTGCGTTCTTCATCGATGCCAGAAC
CAAGAGATCCGTTGTTGAAAGTTTTAGTTTATTAAACTTTTGATCAGACAGCTACGTTTACTG
ACTGGAGTTTGGTAGTCCTCTGGCGGGCGCTGGCCAGCCCCGGTTAGGGGGCCGGCCGCGG
AGGACCGCGGCCCGCCAAAGCAACAGAGGTAGGTACACATAGGGTGTGAGAGAAGAGGTC
CGAAGACCCCTAGAACTCGGTAATGATCCTTCCGCAGGTTCACCTACGGAAACCTTGTTACG
AACTTCTCCT

>BSNB_0583_Colletotrichum_sp

ATCCGAGGTCAACCTTTGGAAAATTGGGGGTTTTACGGCTAGAGTCCCTCCGAATCCCAGTG
CGAGACGAAAAGTTACTACGCAAAGGAGGCTCCGAGAGGGTCCGCCACTACCTTTGAGGGC
CTACGTCGACCGTAGAGCCCCAACGCCAAGCGGTGCTTGAGGGTTGAAATGACGCTCGAAC

AGGCATGCCCGCCAGAATGCTGGCGGGCGCAATGTGCGTTCAAAGATTCGATGATTCACTG
AATTCTGCAATTCACATTACTTATCGCATTTCGCTGCGTTCTTCATCGATGCCAGAACCAAGA
GATCCGTTGTTAAAAGTTTTGATTATTTGCTTGTGCCACTCAGAAGAAACGTCGCTAAATCAG
AGTTTGGTTATCCTCCGGCGGGCACCCCGACGAGCGGGGCCGGGAGCGGGCCGCAGTGGCC
GCGCTGCCCGCCGAAGCAACGGTTATAGGTATGTTCACAAAGGGTTATAGAGCGGTAACTC
AGTAATGATCCCTCCGCTGGTTCACCAACGGAGACCTTGTTAC

>BSNB_0584_Endomelanconiopsis_endophytica

AGGAGAAGTTCGTAACAAGGTTTCCGTAGGTGAACCTGCGGAAGGATCATTACCGAGTTCT
AGGGGTCTTCGGACCTCTTCTCTCACACCCTATGTGTACCTACCTCTGTTGCTTTGGCGGGCC
GCGGTCCTCCGCGGCCGGCCCCCTAACCGGGGCTGGCCAGCGCCCGCCAGAGGACTACCAA
ACTCCAGTCAGTAAACGTAGCTGTCTGATCAAAAGTTTAATAAACTAAAACTTTCAACAACG
GATCTCTTGGTTCTGGCATCGATGAAGAACGCAGCGAAATGCGATAAGTAATGTGAATTGCA
GAATTCAGTGAATCATCGAATCTTTGAACGCACATTGCGCCCCTTGGTATTCCGAGGGGCAT
GCCTGTTCGAGCGTCATTTCACCACTCAAGCTCTGCTTGGTATTGGGCGCCGTCCTTCACCGG
ACGCGCCTCAAAGACCTCGGCGGTGGCGTCTTGCCTCAAGCGTAGTAGAAAACACCTCGCTT
TGGAGGACGGGACGTTCGCTCGCCGGACGAACCTTCTGAATTTTCTCAAGGTTGACCTCGGA
TCAGGT

>BSNB_0586_Xylaria_sp

GGATCCGAGGTCAACCTTTAAAAATTGGGGTGTTTTACGGCAGGGGACCGGTCCAGCTACA
GGCGAGGTGAAAATCTACTACGTCTGGAGTGTGAACCGACCACGCCACTAACTTTAGGGAG
CTACGCTTCCGTAGGCTCCCAACGCTAAGCAACAGGGGCTTAAGGGTTGAAATGACGCTCG
AACAGGCATGCCCACTAGAATACTAATGGGCGCAATGTGCGTTCAAAGATTCGATGATTCA
CTGAATTCTGCAATTCACATTACTTATCGCATTTCGCTGCGTTCTTCATCGATGCCAGAACCA
AGAGATCCGTTGTTGAAAGTTTTAACTTATTTAGTTATACGTTCAGAATTCAATGCTAAACAG
AGTTTTGCGGGCCGCCGGCAGGTTCCCCCAGCGCCCCCCCTAAAGCAGGCCCTACAATGTA
GGGGCGCTACAGGGTAGGCCAGGGCCTGCCGAGGCAACAGAAGGTATGTTCACAGGGGTTT
GGGAGTTGTAAAAACTCTTTAATGATCCCTCCGCTGGTTCACCAACGGAGACC

>BSNB_0587_Endomelanconiopsis_sp

GGAGAAGTTCGTAACAAGGTTTCCGTAGGTGAACCTGCGGAAGGATCATTACCGAGTTCTA
GGGGTCTCCGGACCTCTTCTCTCACACCCTATGTGTATCTACCTCTGTTGCTTTGGCGGGCCG
CGGTCCTCCGCGGCCGGCCCCCTAACCGGGGCTGGCCAGCGCCCGCCAGAGGACTACCAAA
CTCCAGTCAGTAAACGTAGCTGTCTGATCAAAAGTTTAATAAACTAAAACTTTCAACAACGG
ATCTCTTGGTTCTGGCATCGATGAAGAACGCAGCGAAATGCGATAAGTAATGTGAATTGCAG
AATTCAGTGAATCATCGAATCTTTGAACGCACATTGCGCCCCTTGGTATTCCGAGGGGCATG
CCTGTTCGAGCGTCATTTCACCACTCAAGCTCTGCTTGGTATTGGGCGCCGTCCTTCACCGGA
CGCGCCTCAAAGACCTCGGCGGTGGCGTCTTGCCTCAAGCGTAGTAGAAAACACCTCGCTTT
GGAGGACGGGACGTTCGCTCGCCGGACGAACCTTCTGAATTTTCTCAAGGTTGACCTCGGAT
CAGGTA

>BSNB_0588_Endomelanconiopsis_endophytica

AGGAGAAGTTCGTAACAAGGTTTCCGTAGGTGAACCTGCGGAAGGATCATTACCGAGTTCT
AGGGGTCTTCGGACCTCTTCTCTCACACCCTATGTGTACCTACCTCTGTTGCTTTGGCGGGCC
GCGGTCCTCCGCGGCCGGCCCCCTAACCGGGGCTGGCCAGCGCCCGCCAGAGGACTACCAA
ACTCCAGTCAGTAAACGTAGCTGTCTGATCAAAAGTTTAATAAACTAAAACTTTCAACAACG
GATCTCTTGGTTCTGGCATCGATGAAGAACGCAGCGAAATGCGATAAGTAATGTGAATTGCA

GAATTCAGTGAATCATCGAATCTTTGAACGCACATTGCGCCCCTTGGTATTCCGAGGGGCAT
GCCTGTTCGAGCGTCATTTCACCACTCAAGCTCTGCTTGGTATTGGGCGCCGTCCTTCACCGG
ACGCGCCTCAAAGACCTCGGCGGTGGCGTCTTGCCTCAAGCGTAGTAGAAAACACCTCGCTT
TGGAGGACGGGACGTTCGCTCGCCGGACGAACCTTCTGAATTTTCTCAAGGTTGACCTCGGA
TCAGGT

>BSNB_0589_Cladosporium_sp

CGTAACAAGGTCTCCGTAGGTGAACCTGCGGAGGGATCATTACAAGTGACCCCGGTCTAAC
CACCGGGATGTTCATAACCCTTTGTTGTCCGACTCTGTTGCCTCCGGGGCGACCCTGCCTTCG
GGCGGGGGCTCCGGGTGGACACTTCAAACTCTTGCGTAACTTTGCAGTCTGAGTAAACTTAA
TTAATAAATTAAAACTTTTAACAACGGATCTCTTGGTTCTGGCATCGATGAAGAACGCAGCG
AAATGCGATAAGTAATGTGAATTGCAGAATTCAGTGAATCATCGAATCTTTGAACGCACATT
GCGCCCCCTGGTATTCCGGGGGGCATGCCTGTTCGAGCGTCATTTCACCACTCAAGCCTCGC
TTGGTATTGGGCAACGCGGTCCGCCGCGTGCCTCAAATCGACCGGCTGGGTCTTCTGTCCCCT
AAGCGTTGTGGAAACTATTCGCTAAAGGGTGTTCGGGAGGCTACGCCGTAAAACAACCCCA
TTTCTAAGGTGACCTCGGATCAG

>BSNB_0590_Colletotrichum_gloeosporioides

CTACCTGATCCGAGGTCAACCTTTGGAAAATTGGGGGGTTTTACGGCAAGAGTCCCTCCGGA
TCCCAGTGCGAGACGTAAAGTTACTACGCAAAGGAGGCTCCGGGAGGGTCCGCCACTACCT
TTGAGGGCCTACATCAGCTGTAGGGCCCCAACACCAAGCAGAGCTTGAGGGTTGAAATGAC
GCTCGAACAGGCATGCCCGCCAGAATGCTGGCGGGCGCAATGTGCGTTCAAAGATTCGATG
ATTCACTGAATTCTGCAATTCACATTACTTATCGCATTTCGCTGCGTTCTTCATCGATGCCAG
AACCAAGAGATCCGTTGTTAAAAGTTTTGATTATTTGCTTGTACCACTCAGAAGAAACGTCG
TTAAATCAGAGTTTGGTTATCCTCCGGCGGGCGCCGACCCGCCCGGAGGCGGGAGGCCGGG
AGGGTCGCGGAGACCCTACCCGCCGAAGCAACAGTTATAGGTATGTTCACAAAGGGTTATA
GAGCGTAAACTCAGTAATGATCCCTCCGCTGGTTCACCAACGGAGACCTTGTTACGACTTCT
CC

>BSNB_0615_Colletotrichum_fructicola

CCTTGATCCGAGGTCCACCTTTGGAAAATTGGGGGGTTTTACGGCAAGAGTCCTCCGGATCC
AGTGCGAGACGTAAAGTTACTACGCAAAGGAGGCTCCGGGAGGGTCCGCCACTACCTTTGA
GGGCCTACATCAGCTGTAGGGCCCCAACACCAAGCAGAGCTCGAGGGTTGAAATGACGCTC
GAACAGGCATGCCCGCCAGAATGCTGGCGGGCGCAATGTGCGTTCAAAGATTCGATGATTC
ACTGAATTCTGCAATTCACATTACTTATCGCATTTCGCTGCGTTCTTCATCGATGCCAGAACC
AAGAGATCCGTTGTTAAAAGTTTTGATTATTTGCTTGTACCACTCAGAAGAAACGTCGTTAA
ATCAGAGTTTGGTTATCCTCCGGCGGGCGCCGACCCGCCCGGAGGCGGGAGGCCGGGAGGG
TCGCGGAGACCCTACCCGCCGAAGCAACAGTTATAGGTATGTTCACAAAGGGTTATAGAGC
GTAAACTCAGTAATGATCCCTCCGCTGGTTCACCAACGG

>BSNB_0622_Colletotrichum_gloeosporioides

CTACCTGATCCGAGGTCAACCTTTGGAAAATTGGGGGGTTTTACGGCAAGAGTCCCTCCGGA
TCCCAGTGCGAGACGTAAAGTTACTACGCAAAGGAGGCTCCGGGAGGGTCCGCCACTACCT
TTGAGGGCCTACATCAGCTGTAGGGCCCCAACACCAAGCAGAGCTTGAGGGTTGAAATGAC
GCTCGAACAGGCATGCCCGCCAGAATGCTGGCGGGCGCAATGTGCGTTCAAAGATTCGATG
ATTCACTGAATTCTGCAATTCACATTACTTATCGCATTTCGCTGCGTTCTTCATCGATGCCAG
AACCAAGAGATCCGTTGTTAAAAGTTTTGATTATTTGCTTGTACCACTCAGAAGAAACGTCG
TTAAATCAGAGTTTGGTTATCCTCCGGCGGGCGCCGACCCGCCCGGAGGCGGGAGGCCGGG

AGGGTCGCGGAGACCCTACCCGCCGAAGCAACAGTTATAGGTATGTTCACAAAGGGTTATA
GAGCGTAAACTCAGTAATGATCCCTCCGCTGGTTCACCAACGGAGACCTTGTTACGACTTCT
CC

>BSNB_0623_Colletotrichum_gloeosporioides

GTCAACCTTTGGAAAATTGGGGGTTTTACGGCTAGAGTCCCTCCGGATCCCAGTGCGAGACG
TAAAGTTACTACGCAAAGGAGGCTCCGGGAGGGTCCGCCACTACCTTTGAGGGCCTACATC
AGCTGTAGGGCCCCAACACCAAGCAGAGCTTGAGGGTTGAAATGACGCTCGAACAGGCATG
CCCGCCAGAATGCTGGCGGGCGCAATGTGCGTTCAAAGATTCGATGATTCACTGAATTCTGC
AATTCACATTACTTATCGCATTTCGCTGCGTTCTTCATCGATGCCAGAACCAAGAGATCCGTT
GTTAAAAGTTTTGATTATTTGCTTGTACCACTCAGAAGAAACGTCGTTAAATCAGAGTTTGGT
TATCCTCCGGCGGGCGCCGACCCGCCCGGGGGCGGGAGGCCGGGAGGGTCGCGGAGACCC
TACCCGCCGAAGCAACAGTTATAGGTATGTTCACAAAGGGTTATAGAGCGTAAACTCAGTA
ATGATCCCTCCGCTGGTTAACCAACGGAGACCTTGTTA

>BSNB_0624_Thanatephorus_cucumeris

AGTTCAGAAATTTTGTCCGAAGACGTTAGAAGCGCGAACACTAGAATACCCTCCACAGCAA
CGCAGATAATTATCACGCTGAAGCGGCTGGTAACGTTCGCACTAATGCATTTCAGAGGAGCC
GACTACGAGAGCCGGCACGACCTCCAAGTCCAAGCCTTCGTCAATAAAGCCGAAGGTTGAG
AATTCCATGAGACTCAAACAGGCATGCTCCTCGGAATACCAAGGAGCGCAAGGTGCGTTCA
AAGATTCGATGATTCACTGAATTCTGCAATTCACATTACTTATCGCATTTCGCTGCGTTCTTCA
TCGATGCGAGAGCCAAGAGATCCGTTGCTGAAAGTTGTATATAAATTGCGTTATAGCAAAGT
ATGACATTCTAAAACTGAATCGTTTGTAGTAAAGCATAAGCCCGACACCTACAAGTGCGCG
AACGCACCCACAAGCCGGCCTATGAAAAGTGCACAGAAGTTGAGAGTGGATGAGACAGGC
GTGCACATGCCCTTGCGAGCCAGCAGACAACCCGTTCAAAACTCGATAATGATCCTTCCGCA
GGTTCACCTACGGAAACCTTGTTACGAACTTCTCCT
>BSNB_0625_Colletotrichium_gloeosporioides

CCTGATCCGAGGTCAACCTTTGGAGATTTGGGGGTTTTACGGCAAGAGTCCCTCCGGATCCC
AGTGCGAGACGTAAAGTTACTACGCAAAGGAGGCTCCGGGAGGGTCCGCCACTACCTTTGA
GGGCCTACATCGGCTGTAGGGCCCCAACACCAAGCAGAGCTTGAGGGTTGAAATGACGCTC
GAACAGGCATGCCCGCCAGAATGCTGGCGGGCGCAATGTGCGTTCAAAGATTCGATGATTC
ACTGAATTCTGCAATTCACATTACTTATCGCATTTCGCTGCGTTCTTCATCGATGCCAGAACC
AAGAGATCCGTTGTTAAAAGTTTTGATTATTTGCTTGTACCACTCAGAAGAAACGTCGTTAA
ATCAGAGTTTGGTTATCCTCCGGCGGGCGCCGACCCGCCCGGGGGCGGGAGGCCGGGAGGG
TCACGGAGACCCTGCCCGCCGAAGCAACAGTTATAGGTATGTTCACAAAGGGTTGTAGAGC
GTAAACTCAGTAATGATCCCTCCGCTGGTTCACCAACGGAGACCTTGTT

>BSNB_0627_Colletotrichum_sp

GTCAACCTTTGGAAAATTGGGGGTTTTACGGCTAGAGTCCCTCCGGATCCCAGTGCGAGACG
TAAAGTTACTACGCAAAGGAGGCTCCGGGAGGGTCCGCCACTACCTTTGAGGGCCTACATC
AGCTGTAGGGCCCCAACACCAAGCAGAGCTTGAGGGTTGAAATGACGCTCGAACAGGCATG
CCCGCCAGAATGCTGGCGGGCGCAATGTGCGTTCAAAGATTCGATGATTCACTGAATTCTGC
AATTCACATTACTTATCGCATTTCGCTGCGTTCTTCATCGATGCCAGAACCAAGAGATCCGTT
GTTAAAAGTTTTGATTATTTGCTTGTACCACTCAGAAGAAACGTCGTTAAATCAGAGTTTGGT
TATCCTCCGGCGGGCGCCGACCCGCCCGGGGGCGGGAGGCCGGGAGGGTCGCGGAGACCC
TACCCGCCGAAGCAACAGTTATAGGTATGTTCACAAAGGGTTATAGAGCGTAAACTCAGTA
ATGATCCCTCCGCTGGTTAACCAACGGAGACCTTGTTA

>BSNB_0628_Colletotrichum_theobromicola

CTGATCCGAGGTCAACCTTTGGAAAATTGGGGGGTTTACGGCAAGAGTCCCTCCGGATCCC
AGTGCGAGACGTAAAGTTACTACGCAAAGGAGGCTCCGGGAGGGTCCGCCACTACCTTTGA
GGGCCTACATCAGCTGTAGGGCCCCAACACCAAGCAGAGCTTGAGGGTTGAAATGACGCTC
GAACAGGCATGCCCGCCAGAATGCTGGCGGGCGCAATGTGCGTTCAAAGATTCGATGATTC
ACTGAATTCTGCAATTCACATTACTTATCGCATTTCGCTGCGTTCTTCATCGATGCCAGAACC
AAGAGATCCGTTGTTAAAAGTTTTGATTATTTGCTTGTACCACTCAGAAGAAACGTCGTTAA
ATCAGAGTTTGGTTATCCTCCGGCGGGCGCCGACCCGCCCGGGGGGGCGGGAGGCCGGGAG
GGTCACGGGGACCCTACCCGCCGAAGCAACAGTTGTAGGTATGTTCACAAAGGGTTGTAGA
GCGTAAACTCAGTAATGATCCCTCCGCTGGTTCACCAACGGAGACCTTGTTA

>BSNB_0637_Colletotrichum_theobromicola

CTGATCCGAGGTCAACCTTTGGAAAATTGGGGGGTTTACGGCAAGAGTCCCTCCGGATCCC
AGTGCGAGACGTAAAGTTACTACGCAAAGGAGGCTCCGGGAGGGTCCGCCACTACCTTTGA
GGGCCTACATCAGCTGTAGGGCCCCAACACCAAGCAGAGCTTGAGGGTTGAAATGACGCTC
GAACAGGCATGCCCGCCAGAATGCTGGCGGGCGCAATGTGCGTTCAAAGATTCGATGATTC
ACTGAATTCTGCAATTCACATTACTTATCGCATTTCGCTGCGTTCTTCATCGATGCCAGAACC
AAGAGATCCGTTGTTAAAAGTTTTGATTATTTGCTTGTACCACTCAGAAGAAACGTCGTTAA
ATCAGAGTTTGGTTATCCTCCGGCGGGCGCCGACCCGCCCGGGGGGGCGGGAGGCCGGGAG
GGTCACGGGGACCCTACCCGCCGAAGCAACAGTTGTAGGTATGTTCACAAAGGGTTGTAGA
GCGTAAACTCAGTAATGATCCCTCCGCTGGTTCACCAACGGAGACCTTGTTA

>BSNB_0638_Cladosporium_cladosporioides

CTGATCCGAGGTCACCTTAGAAATGGGGTTGTTTTACGGCGTAGCCTCCCGAACACCCTTTA
GCGAATAGTTTCCACAACGCTTAGGGGACAGAAGACCCAGCCGGTCGATTTGAGGCACGCG
GCGGACCGCGTTGCCCAATACCAAGCGAGGCTTGAGTGGTGAAATGACGCTCGAACAGGCA
TGCCCCCCGGAATACCAGGGGGCGCAATGTGCGTTCAAAGATTCGATGATTCACTGAATTCT
GCAATTCACATTACTTATCGCATTTCGCTGCGTTCTTCATCGATGCCAGAACCAAGAGATCCG
TTGTTAAAAGTTTTAATTTATTAATTAAGTTTACTCAGACTGCAAAGTTACGCAAGAGTTTGA
AGTGTCCACCCGGAGCCCCCGCCCGAAGGCAGGGTCGCCCCGGAGGCAACAGAGTCGGAC
AACAAAGGGTTATGAACATCCCGGTGGTTAGACCGGGGTCACTTGTAATGATCCCTCCGCAG
GTTCACCTACGGAGACCTTGTTACG

>BSNB_0641_Cladosporium_cladosporioides

CTGATCCGAGGTCAACCTTTGGAAAATTGGGGGGTTTACGGCAAGAGTCCCTCCGGATCCC
AGTGCGAGACGAAATGTTACTACGCAAAGGAGGCTCCGGGAGGGTCCGCCACTACCTTTGA
GGGCCTACGTCGACCGTAGAGCCCCAACACCAAGCAGAGCTTGAGGGTTGAAATGACGCTC
GAACAGGCATGCCCGCCAGAATGCTGGCGGGCGCAATGTGCGTTCAAAGATTCGATGATTC
ACTGAATTCTGCAATTCACATTACTTATCGCATTTCGCTGCGTTCTTCATCGATGCCAGAACC
AAGAGATCCGTTGTTAAAAGTTTTGATTATTTTGCTTATGCCACTCAGAAGAAACGTCGTTAC
AATAGAGTTTGGTTATCCTCCGGCGGGCGCCGGGTCCGGTCCCGCGGGGGGTCCGGTCCGGG
CCGGGAGGCGTCCTTTTCAGGGGACGGCCTACCCGCCGAAGCAACAGTTGTAGGTATGTTCA
CAAAGGGTTATAGAGCGGTAACTCAGTAATGATCCCTCCGCTGGTTCACCAACGGAGACCTT
GTTACGACTTCTCC

>BSNB_0642_Hypoxylon_investiens

CTAATCCGAGGTCAACCACTAGAAAATATAGGGGTTTTAACGGCCAGCAGCCAGGGCCACC

ACACGAGCGAGAGAAATTACTACGCTGAGAGTGTACCCTAACTCCGCCACTAACTTTGAGG
AACTACGCCGTAGATTCCCAACGCTAAGCAACAGGGGCTTAGGGGTCGAAATGACGCTCGA
ATAGGCATGCCCACTAGAATACTAGTGGGCGCAATGTGCGTTCAAAGATTCGATGATTCACT
GAATTCTGCAATTCACATTACTTATCGCATTTCGCTGCGTTCTTCATCGATGCCAGAACCAAG
AGATCCGTTGTTGAAAGTTTTAACTTATTTCAGTTTAGAATTCAGAGAAACAGTGGTAAAAA
CAAGAGTTTAACGGTCCTTCGGCGGGCCGAAGCCGACTACAGGGTAGCTCCAGGGTAGCTC
TAGGGTAGCTATAGGGTAGCTGCAGGGTAAGTGCAGGGTAGCTATAGGGTAACTCTAGGGT
AGCTCCAGGGTAGTTACAGGGTAGCCGTAGCTCACGCCGAGGCAACGACGGTAAGGTTCAC
AAAGGGTTTGGAGTTTTGATAACTCAGTAATGATCCCTCCGCTGGTTCACCAACGGAGACCT
TGTTACAACCTTCTCCT

>BSNB_0644_Diaporthe_sp

ACCTGATCCGAGGTCAAAATTTCAGAAGTTGGGGGTTTAACGGCAGGGCACCGCCAGGGCC
TTCCAGAACGAGATATAACTACTACGCTCGGGGTCCTAGCGAGCTCGCCACTAGATTTCAGG
GCCTGCCTTGTCTCCAAGGCAGTGCCCCAACACCAAGCCAGGCTTGAGGGTTGAAATGACG
CTCGAACAGGCATGCCCTCCGGAATACCAGAGGGCGCAATGTGCGTTCAAAGATTCGATGA
TTCACTGAATTCTGCAATTCACATTACTTATCGCATTTCGCTGCGTTCTTCATCGATGCCAGA
ACCAAGAGATCCGTTGTTGAAAGTTTTGATTCATTTATGTTTTTTACTCAGAGATTCACTAA
GAAACAAGAGTTTGGTTGGCCGCCGGCGGGCTGCTCCCCGTCTCCGGGGGGCCTCAGAAGA
GAGGCCGGCCTTCGCCGAGGCAACAATAAGGTATAAGTTCACAAAGGGTTTCTGGGTGCGC
CGGGGCGCGTTCCAGCAATGATCCCTCCGCTGGTTCACCAACGGAGACCTTGTT

>BSNB_0645_Colletotrichum_theobromicola

TGATCCGAGGTCAACCTTTGGAAAATTGGGGGGTTTTACGGCAAGAGTCCCTCCGGATCCCA
GTGCGAGACGTAAAGTTACTACGCAAAGGAGGCTCCGGGAGGGTCCGCCACTACCTTTGAG
GGCCTACATCAGCTGTAGGGCCCCAACACCAAGCAGAGCTTGAGGGTTGAAATGACGCTCG
AACAGGCATGCCCGCCAGAATGCTGGCGGGCGCAATGTGCGTTCAAAGATTCGATGATTCA
CTGAATTCTGCAATTCACATTACTTATCGCATTTCGCTGCGTTCTTCATCGATGCCAGAACCA
AGAGATCCGTTGTTAAAAGTTTTAATTATTTGCTTGTACCACTCAGAAGAAACGTCGTTAAAT
CAGAGTTTGGTTATCCTCCGGCGGGCGCCGACCCGCCCGGGGGGGCGGGAGGCCGGGAGGG
TCACGGGGACCCTACCCGCCGAAGCAACAGTTGTAGGTATGTTCACAAAGGGTTGTAGAGC
GTAAACTCAGTAATGATCCCTCCGCTGGTTCACCAACGGAGACC

>BSNB_0646_Colletotrichum_gloeosporioides

CTACCTGATCCGAGGTCAACCTTTGGAAAATTGGGGGGTTTTACGGCAAGAGTCCCTCCGGA
TCCCAGTGCGAGACGTAAAGTTACTACGCAAAGGAGGCTCCGGGAGGGTCCGCCACTACCT
TTGAGGGCCTACATCAGCTGTAGGGCCCCAACACCAAGCAGAGCTTGAGGGTTGAAATGAC
GCTCGAACAGGCATGCCCGCCAGAATGCTGGCGGGCGCAATGTGCGTTCAAAGATTCGATG
ATTCACTGAATTCTGCAATTCACATTACTTATCGCATTTCGCTGCGTTCTTCATCGATGCCAG
AACCAAGAGATCCGTTGTTAAAAGTTTTGATTATTTGCTTGTACCACTCAGAAGAAACGTCG
TTAAATCAGAGTTTGGTTATCCTCCGGCGGGCGCCGACCCGCCCGGAGGCGGGAGGCCGGG
AGGGTCGCGGAGACCCTACCCGCCGAAGCAACAGTTATAGGTATGTTCACAAAGGGTTATA
GAGCGTAAACTCAGTAATGATCCCTCCGCTGGTTCACCAACGGAGACCTTGTTACGACTTCT
CC

>BSNB_0647_Fusarium_sp

TGATCCGAGGTCAACATTCAGAAGTTGGGGTTTAACGGCGTGGCCGCGACGATTACCAGTA
ACGAGGTGTAATTACTACGCTATGGAAGCTCGACGTGACCGCCAATAGATTTGGGGACCGC

GGATTGCTCCACGAATCCCAACACCAAGCTGTGCTTGAGGGTTGAAATGACGCTCGAACAG
GCATGCCCGCCAGAATACTGGCGGGCGCAATGTGCGTTCAAAGATTCGATGATTCACTGAAT
TCTGCAATTCACATTACTTATCGCATTTTGCTGCGTTCTTCATCGATGCCAGAACCAAGAGAT
CCGTTGTTGAAAGTTTTGATTTATTTGTTTGTTTTACTCAGAAGTTACACTAGAAACAGAGTTT
AGGGTCCTCTGGCGGGCCGTCCCGTTTTACCGGGGCCGGGCTGATCCGCCGAGGCAACAAT
AGGTATGTTCACAGGGGTTTGGGAGTTGTAAACTCGGTAATGATCCCTCCGCTGGTTCACCA
ACGGAGACCTTGTTACGACTTCTC

>BSNB_0649_Colletotrichum_gloeosporioides

CTACCTGATCCGAGGTCAACCTTTGGAAAATTGGGGGGTTTTACGGCAAGAGTCCCTCCGGA
TCCCAGTGCGAGACGTAAAGTTACTACGCAAAGGAGGCTCCGGGAGGGTCCGCCACTACCT
TTGAGGGCCTACATCAGCTGTAGGGCCCCAACACCAAGCAGAGCTTGAGGGTTGAAATGAC
GCTCGAACAGGCATGCCCGCCAGAATGCTGGCGGGCGCAATGTGCGTTCAAAGATTCGATG
ATTCACTGAATTCTGCAATTCACATTACTTATCGCATTTCGCTGCGTTCTTCATCGATGCCAG
AACCAAGAGATCCGTTGTTAAAAGTTTTGATTATTTGCTTGTACCACTCAGAAGAAACGTCG
TTAAATCAGAGTTTGGTTATCCTCCGGCGGGCGCCGACCCGCCCGGAGGCGGGAGGCCGGG
AGGGTCGCGGAGACCCTACCCGCCGAAGCAACAGTTATAGGTATGTTCACAAAGGGTTATA
GAGCGTAAACTCAGTAATGATCCCTCCGCTGGTTCACCAACGGAGACCTTGTTACGACTTCT
CC

>BSNB_0650_Colletotrichum_gloeosporioides

CTGATCCGAGGTCAACCTTTGGAAAATTGGGGGGTTTTACGGCAAGAGTCCCTCCGGATCCC
AGTGCGAGACGAAATGTTACTACGCAAAGGAGGCTCCGGGAGGGTCCGCCACTACCTTTGA
GGGCCTACGTCGACCGTAGAGCCCCAACACCAAGCAGAGCTTGAGGGTTGAAATGACGCTC
GAACAGGCATGCCCGCCAGAATGCTGGCGGGCGCAATGTGCGTTCAAAGATTCGATGATTC
ACTGAATTCTGCAATTCACATTACTTATCGCATTTCGCTGCGTTCTTCATCGATGCCAGAACC
AAGAGATCCGTTGTTAAAAGTTTTGATTATTTTGCTTATGCCACTCAGAAGAAACGTCGTTAC
AATAGAGTTTGGTTATCCTCCGGCGGGCGCCGGGTCCGGTCCCGCGGGGGGTCCGGTCCGGG
CCGGGAGGCGTCCTTTTCAGGGGACGGCCTACCCGCCGAAGCAACAGTTGTAGGTATGTTCA
CAAAGGGTTATAGAGCGGTAACTCAGTAATGATCCCTCCGCTGGTTCACCAACGGAGACCTT
GTTACGACTTCTCC

>BSNB_0651_Fusarium_sp

TGATCCGAGGTCAACATTCAGAAGTTGGGGTTTAACGGCGTGGCCGCGACGATTACCAGTA
ACGAGGTGTAATTACTACGCTATGGAAGCTCGACGTGACCGCCAATAGATTTGGGGACCGC
GGATTGCTCCACGAATCCCAACACCAAGCTGTGCTTGAGGGTTGAAATGACGCTCGAACAG
GCATGCCCGCCAGAATACTGGCGGGCGCAATGTGCGTTCAAAGATTCGATGATTCACTGAAT
TCTGCAATTCACATTACTTATCGCATTTTGCTGCGTTCTTCATCGATGCCAGAACCAAGAGAT
CCGTTGTTGAAAGTTTTGATTTATTTGTTTGTTTTACTCAGAAGTTACACTAGAAACAGAGTTT
AGGGTCCTCTGGCGGGCCGTCCCGTTTTACCGGGGCCGGGCTGATCCGCCGAGGCAACAAT
AGGTATGTTCACAGGGGTTTGGGAGTTGTAAACTCGGTAATGATCCCTCCGCTGGTTCACCA
ACGGAGACCTTGTTACGACTTCTC

>BSNB_0652_Colletotrichum_theobromicola

CTGATCCGAGGTCAACCTTTGGAAAATTGGGGGGTTTTACGGCAAGAGTCCCTCCGGATCCC
AGTGCGAGACGTAAAGTTACTACGCAAAGGAGGCTCCGGGAGGGTCCGCCACTACCTTTGA
GGGCCTACATCAGCTGTAGGGCCCCAACACCAAGCAGAGCTTGAGGGTTGAAATGACGCTC
GAACAGGCATGCCCGCCAGAATGCTGGCGGGCGCAATGTGCGTTCAAAGATTCGATGATTC

ACTGAATTCTGCAATTCACATTACTTATCGCATTTCGCTGCGTTCTTCATCGATGCCAGAACC
AAGAGATCCGTTGTTAAAAGTTTTGATTATTTGCTTGTACCACTCAGAAGAAACGTCGTTAA
ATCAGAGTTTGGTTATCCTCCGGCGGGCGCCGACCCGCCCGGGGGGGCGGGAGGCCGGGAG
GGTCACGGGGACCCTACCCGCCGAAGCAACAGTTGTAGGTATGTTCACAAAGGGTTGTAGA
GCGTAAACTCAGTAATGATCCCTCCGCTGGTTCACCAACGGAGACCTTGTTA

>BSNB_0653_Colletotrichum_gloeosporioides

CTACCTGATCCGAGGTCAACCTTTGGAAAATTGGGGGGTTTTACGGCAAGAGTCCCTCCGGA
TCCCAGTGCGAGACGTAAAGTTACTACGCAAAGGAGGCTCCGGGAGGGTCCGCCACTACCT
TTGAGGGCCTACATCAGCTGTAGGGCCCCAACACCAAGCAGAGCTTGAGGGTTGAAATGAC
GCTCGAACAGGCATGCCCGCCAGAATGCTGGCGGGCGCAATGTGCGTTCAAAGATTCGATG
ATTCACTGAATTCTGCAATTCACATTACTTATCGCATTTCGCTGCGTTCTTCATCGATGCCAG
AACCAAGAGATCCGTTGTTAAAAGTTTTGATTATTTGCTTGTACCACTCAGAAGAAACGTCG
TTAAATCAGAGTTTGGTTATCCTCCGGCGGGCGCCGACCCGCCCGGAGGCGGGAGGCCGGG
AGGGTCGCGGAGACCCTACCCGCCGAAGCAACAGTTATAGGTATGTTCACAAAGGGTTATA
GAGCGTAAACTCAGTAATGATCCCTCCGCTGGTTCACCAACGGAGACCTTGTTACGACTTCT
CC

>BSNB_0654_Colletotrichum_gloeosporioides

CTGATCCGAGGTCAACCTTTGGAAAATTGGGGGGTTTTACGGCAAGAGTCCCTCCGGATCCC
AGTGCGAGACGAAATGTTACTACGCAAAGGAGGCTCCGGGAGGGTCCGCCACTACCTTTGA
GGGCCTACGTCGACCGTAGAGCCCCAACACCAAGCAGAGCTTGAGGGTTGAAATGACGCTC
GAACAGGCATGCCCGCCAGAATGCTGGCGGGCGCAATGTGCGTTCAAAGATTCGATGATTC
ACTGAATTCTGCAATTCACATTACTTATCGCATTTCGCTGCGTTCTTCATCGATGCCAGAACC
AAGAGATCCGTTGTTAAAAGTTTTGATTATTTTGCTTATGCCACTCAGAAGAAACGTCGTTAC
AATAGAGTTTGGTTATCCTCCGGCGGGCGCCGGGTCCGGTCCCGCGGGGGGTCCGGTCCGGG
CCGGGAGGCGTCCTTTTCAGGGGACGGCCTACCCGCCGAAGCAACAGTTGTAGGTATGTTCA
CAAAGGGTTATAGAGCGGTAACTCAGTAATGATCCCTCCGCTGGTTCACCAACGGAGACCTT
GTTACGACTTCTCC

>BSNB_0655_Colletotrichum_gloeosporioides

CTACCTGATCCGAGGTCAACCTTTGGAAAATTGGGGGGTTTTACGGCAAGAGTCCCTCCGGA
TCCCAGTGCGAGACGTAAAGTTACTACGCAAAGGAGGCTCCGGGAGGGTCCGCCACTACCT
TTGAGGGCCTACATCAGCTGTAGGGCCCCAACACCAAGCAGAGCTTGAGGGTTGAAATGAC
GCTCGAACAGGCATGCCCGCCAGAATGCTGGCGGGCGCAATGTGCGTTCAAAGATTCGATG
ATTCACTGAATTCTGCAATTCACATTACTTATCGCATTTCGCTGCGTTCTTCATCGATGCCAG
AACCAAGAGATCCGTTGTTAAAAGTTTTGATTATTTGCTTGTACCACTCAGAAGAAACGTCG
TTAAATCAGAGTTTGGTTATCCTCCGGCGGGCGCCGACCCGCCCGGAGGCGGGAGGCCGGG
AGGGTCGCGGAGACCCTACCCGCCGAAGCAACAGTTATAGGTATGTTCACAAAGGGTTATA
GAGCGTAAACTCAGTAATGATCCCTCCGCTGGTTCACCAACGGAGACCTTGTTACGACTTCT
CC

>BSNB_0660_Thanatephorus_cucumeris

AGTTCAGAAATTTTGTCCGAAGACGTTAGAAGCGCGAACACTAGAATACCCTCCACAGCAA
CGCAGATAATTATCACGCTGAAGCGGCTGGTAACGTTCGCACTAATGCATTTCAGAGGAGCC
GACTACGAGAGCCGGCACGACCTCCAAGTCCAAGCCTTCGTCAATAAAGCCGAAGGTTGAG
AATTCCATGAGACTCAAACAGGCATGCTCCTCGGAATACCAAGGAGCGCAAGGTGCGTTCA
AAGATTCGATGATTCACTGAATTCTGCAATTCACATTACTTATCGCATTTCGCTGCGTTCTTCA

TCGATGCGAGAGCCAAGAGATCCGTTGCTGAAAGTTGTATATAAATTGCGTTATAGCAAAGT
ATGACATTCTAAAACTGAATCGTTTGTAGTAAAGCATAAGCCCGACACCTACAAGTGCGCG
AACGCACCCACAAGCCGGCCTATGAAAAGTGCACAGAAGTTGAGAGTGGATGAGACAGGC
GTGCACATGCCCTTGCGAGCCAGCAGACAACCCGTTCAAAACTCGATAATGATCCTTCCGCA
GGTTCACCTACGGAAACCTTGTTACGAACTTCTCCT

>BSNB_0662_Colletotrichum_gloeosporioides

CTGATCCGAGGTCAACCTTTGGAAAATTGGGGGTTTTACGGCAAGAGTCCCTCCGGATCCC
AGTGCGAGACGTAAAGTTACTACGCAAAGGAGGCTCCGGGAGGGTCCGCCACTACCTTTGA
GGGCCTACATCAGCTGTAGGGCCCCAACACCAAGCAGAGCTTGAGGGTTGAAATGACGCTC
GAACAGGCATGCCCGCCAGAATGCTGGCGGGCGCAATGTGCGTTCAAAGATTCGATGATTC
ACTGAATTCTGCAATTCACATTACTTATCGCATTTCGCTGCGTTCTTCATCGATGCCAGAACC
AAGAGATCCGTTGTTAAAAGTTTTGATTATTTGCTTGTACCACTCAGAAGAAACGTCGTTAA
ATCAGAGTTTGGTTATCCTCCGGCGGGCGCCGACCCGCCCGGGGGGGCGGGAGGCCGGGAG
GGTCACGGGGACCCTACCCGCCGAAGCAACAGTTGTAGGTATGTTCACAAAGGGTTGTAGA
GCGTAAACTCAGTAATGATCCCTCCGCTGGTTCACCAACGGAGACCTTGTTA

>BSNB_0664_Acutodesmus_obliquus

CCTGAGCTCAGGTCGAAAGTTTAAGACATGCAAAGCATGTTTCCTGCTTGGCCTCTAGCAAA
GTCCACAAGCCACAACTTCGTGTAGTCGGCAGAAGCCGGTGCTACCTATCCAGTTGAAGCCC
ATATCGGGTCCTTGCTTAAGCCTCTAAGCTTCAGCCAACCCAATCGGAGTGAACCAATTGGG
AAAGCCAGATCCACCCCTAAGGCCAACTAGAAGCTGACCAGCCCTCCAAAAGGAGAGAGG
GGTGAGGGTATAAACCGACGCTGAGGCAGACATGCTCTTGCCCGAGGGCTCGAGCGCAATA
TGCGTTCAAAGATTCGATGGTTCACGGAATTCTGCAATTCACACTACGTATCGCATTTCGCTG
CGTTCTTCATCGTTGCGAGAGCCAAGATATCCGTTGTTGAGAGTTGTCTTTGGTTAAGATTGC
CAGTTAATAGCAATCAAAGCTTCAGAGTTTGGTTTTGACAGTGGTTAGCACTGGTGTATAAG
CATGCCAAAGCGCCACTGATGCAAGTAATCAAATTACTCGCACCAGCTTGCAAGTACAATT
GGGCAAGCCAATTGCCTTGCTGGCAGCTAAGGCACGGAACAGATAGGTTCGCATTGTGGTTT
TAATAATTCAATGATCCTTCCGCAGGTTCACCTACGGAAACCTTGTTACG

>BSNB_0666_Nodulisporium_sp

ATCCGAGGTCAACCACTAGAAAATAGGGGGTTTTACGGCTAGCAGCCAGGGCCACCACACA
AGCGAGAGAGATTACTACGCTGAGAGTGTACCCTAACTCCGCCACTGATTTTCAGGAACTAC
GCCGAAGCCGTAGACTCCCAACACTAAGCAACAGGGCTTAAGGGTTGAAATGACGCTCGAA
TAGGCATGCCCACTAGAATACTAATGGGCGCAATGTGCGTTCAAAGATTCGATGATTCACTG
AATTCTGCAATTCACATTACTTATCGCATTTCGCTGCGTTCTTCATCGATGCCAGAACCAAGA
GATCCGTTGTTGAAAGTTTTAACTTATTTCAGTTAAGTACTCAGAGATACAGCTGTAAAAAC
AAGAGTTTAATGGTCCTGCGGCGGGCCTTCACGCGGCTACAGGGTAGCTCCAGGGTAGACA
CCTACAGGGTAGGTGTCTCCAGGGTAGCTACAGGGTAACCGCAGCACGCGCCGAGGAAACG
ACGGTAAGGTTCACAAAGGGTTTTGGAGTTTGGAAACTCATTAATGATCCCTCCGCTGGTTC
ACCAACGGAGACC

>BSNB_0668_Cladosporium_herbarum

AGGAGAAGTCGTAACAAGGTCTCCGTAGGTGAACCTGCGGAGGGATCATTATAAGTTCACC
CAGGCTTGTACAGCTGGGGACTGACAACCCTTTGATTTCCGACTCTGTTGCCTCCGGGGCGA
CCCTGCCTTCGGGCGGGGGCTCCGGGTGGACACTTCAAACTCTTGCGTAACTTTGCAGTCTG
AGTAAACTTAATTAATAAATTAAAACTTTTAACAACGGATCTCTTGGTTCTGGCATCGATGA
AGAACGCAGCGAAATGCGATAAGTAATGTGAATTGCAGAATTCAGTGAATCATCGAATCTT

TGAACGCACATTGCGCCCCCTGGTATTCCGGGGGGCATGCCTGTTCGAGCGTCATTTCACCA
CTCAAGCCTCGCTTGGTATTGGGCAACGCGGTCCGCCGCGTGCCTCAAATCGTCCGGCTGGG
TCTTCTGTCCCCTAAGCGTTGTGGAA

>BSNB_0678_Fusarium_lateritium

AGGAGAAGTCGTAACAAGGTCTCCGTTGGTGAACCAGCGGAGGGATCATTACCGAGTTTAC
AACTCCCAAACCCCTGTGAACATACCTTAATGTTGCCTCGGCGGATCAGCCCGCGCCCCGTA
AAACGGGACGGCCCGCCAGAGGACCCAAACTCTAATGTTTCTTATTGTAACTTCTGAGTAAA
ACAAACAAATAAATCAAAACTTTCAACAACGGATCTCTTGGTTCTGGCATCGATGAAGAAC
GCAGCAAAATGCGATAAGTAATGTGAATTGCAGAATTCAGTGAATCATCGAATCTTTGAAC
GCACATTGCGCCCGCTGGTATTCCGGCGGGCATGCCTGTTCGAGCGTCATTTCAACCCTCAA
GCCCTCGGGTTTGGTGTTGGGGATCGGCTCTGCCTTCTGGCGGTGCCGCCCCCGAAATACATT
GGCGGTCTCGCTGCAGCCTCCATTGCGTAGTAGCTAACACCTCGCAACTGGAACGCGGCGC
GGCCATGCCGTAAAACCCCAACTTCTGAATGTTGACCTCGGATCAGGTAG

>BSNB_0679_Diaporthe_sp

AGGAGAAGTCGTAACAAGGTCTCCGTTGGTGAACCAGCGGAGGGATCATTGCTGGAACGCG
CTTCGGCGCACCCAGAAACCCTTTGTGAACTTATACCTCACTGTTGCCTCGGCGCAGGCCGG
CCCCTCCCACGGGGCCCCTCCGGAAGGAGGAGCAGCCCGCCGGCGGCCAACTAAACTCTTG
TTTCTTAGTGGATCTCTGAGTAAAAAACATAAATGAATCAAAACTTTCAACAACGGATCTCT
TGGTTCTGGCATCGATGAAGAACGCAGCGAAATGCGATAAGTAATGTGAATTGCAGAATTC
AGTGAATCATCGAATCTTTGAACGCACATTGCGCCCTCTGGTATTCCGGAGGGCATGCCTGT
TCGAGCGTCATTTCAACCCTCAAGCACTGCTTGGTGTTGGGGCACCGCCTGTGAAAGGGCGG
GCCCTGAAATCTAGTGGCGAGCTCGCCAGGACCCCGAGCGTAGTAGTTTATATCTCGTTCTG
GAAGGCCCTGGCGGTGCACTGCCGTTAAACCCCCAACTTCTGAAATTTGACCTCGGA

>BSNB_0680_Endomelanconiopsis_endophytica

TACCTGATCCGAGGTCAACCTTGAGAAAATTCAGAAGGTTCGTCCGGCGAGCGAACGTCCC
GTCCTCCAAAGCGAGGTGTTTTCTACTACGCTTGAGGCAAGACGCCACCGCCGAGGTCTTTG
AGGCGCGTCCGGTGAAGGACGGCGCCCAATACCAAGCAGAGCTTGAGTGGTGAAATGACG
CTCGAACAGGCATGCCCCTCGGAATACCAAGGGGCGCAATGTGCGTTCAAAGATTCGATGA
TTCACTGAATTCTGCAATTCACATTACTTATCGCATTTCGCTGCGTTCTTCATCGATGCCAGA
ACCAAGAGATCCGTTGTTGAAAGTTTTAGTTTATTAAACTTTTGATCAGACAGCTACGTTTAC
TGACTGGAGTTTGGTAGTCCTCTGGCGGGCGCTGGCCAGCCCCGGTTAGGGGGCCGGCCGCG
GAGGACCGCGGCCCGCCAAAGCAACAGAGGTAGATACACATAGGGTGTGAGAGAAGAGGT
CCGGAGACCCCTAGAACTCGGTAATGATCCTTCCGCAGGTTCACCTACGGAAACCTTGTTAC
GAACTTCTCC

>BSNB_0681_Endomelanconiopsis_endophytica

TACCTGATCCGAGGTCAACCTTGAGAAAATTCAGAAGGTTCGTCCGGCGAGCGAACGTCCC
GTCCTCCAAAGCGAGGTGTTTTCTACTACGCTTGAGGCAAGACGCCACCGCCGAGGTCTTTG
AGGCGCGTCCGGTGAAGGACGGCGCCCAATACCAAGCAGAGCTTGAGTGGTGAAATGACG
CTCGAACAGGCATGCCCCTCGGAATACCAAGGGGCGCAATGTGCGTTCAAAGATTCGATGA
TTCACTGAATTCTGCAATTCACATTACTTATCGCATTTCGCTGCGTTCTTCATCGATGCCAGA
ACCAAGAGATCCGTTGTTGAAAGTTTTAGTTTATTAAACTTTTGATCAGACAGCTACGTTTAC
TGACTGGAGTTTGGTAGTCCTCTGGCGGGCGCTGGCCAGCCCCGGTTAGGGGGCCGGCCGCG
GAGGACCGCGGCCCGCCAAAGCAACAGAGGTAGATACACATAGGGTGTGAGAGAAGAGGT

CCGGAGACCCCTAGAACTCGGTAATGATCCTTCCGCAGGTTCACCTAGGGAAACCTTGTTAC
GAACTTCTCC

>BSNB_0682_Colletotrichum_siamense

GTCAACCTTTGGAAAATTGGGGGTTTTACGGCTAGAGTCCCTCCGGATCCCAGTGCGAGACG
TAAAGTTACTACGCAAAGGAGGCTCCGGGAGGGTCCGCCACTACCTTTGAGGGCCTACATC
AGCTGTAGGGCCCCAACACCAAGCAGAGCTTGAGGGTTGAAATGACGCTCGAACAGGCATG
CCCGCCAGAATGCTGGCGGGCGCAATGTGCGTTCAAAGATTCGATGATTCACTGAATTCTGC
AATTCACATTACTTATCGCATTTCGCTGCGTTCTTCATCGATGCCAGAACCAAGAGATCCGTT
GTTAAAAGTTTTGATTATTTGCTTGTACCACTCAGAAGAAACGTCGTTAAATCAGAGTTTGGT
TATCCTCCGGCGGGCGCCGACCCGCCCGGGGGCGGGAGGCCGGGAGGGTCGCGGAGACCC
TACCCGCCGAAGCAACAGTTATAGGTATGTTCACAAAGGGTTATAGAGCGTAAACTCAGTA
ATGATCCCTCCGCTGGTTAACCAACGGAGACCTTGTTA

>BSNB_0684_Endomelanconiopsis_endophytica

ACCTGATCCGAGGTCAACCTTGAGAAAATTCAGAAGGTTCGTCCGGCGAGCGAACGTCCCG
TCCTCCAAAGCGAGGTGTTTTCTACTACGCTTGAGGCAAGACGCCACCGCCGAGGTCTTTGA
GGCGCGTCCGGTGAAGGACGGCGCCCAATACCAAGCAGAGCTTGAGTGGTGAAATGACGCT
CGAACAGGCATGCCCCTCGGAATACCAAGGGGCGCAATGTGCGTTCAAAGATTCGATGATT
CACTGAATTCTGCAATTCACATTACTTATCGCATTTCGCTGCGTTCTTCATCGATGCCAGAAC
CAAGAGATCCGTTGTTGAAAGTTTTAGTTTATTAAACTTTTGATCAGACAGCTACGTTTACTG
ACTGGAGTTTGGTAGTCCTCTGGCGGGCGCTGGCCAGCCCCGGTTAGGGGGCCGGCCGCGG
AGGACCGCGGCCCGCCAAAGCAACAGAGGTAGGTACACATAGGGTGTGAGAGAAGAGGTC
CGAAGACCCCTAGAACTCGGTAATGATCCTTCCGCAGGTTCACCTACGGAAACCTTGTTACG
AACTTCTCCT

>BSNB_0686_Fusarium_avenaceum

AGCTACTACGCAATGGAGGCTGCAGCGAGACCGCCAATGTATTTCGGGGGCGGCACCGCCA
GAAGGCAGAGCCGATCCCCAACACCAAACCCGAGGGCTTGAGGGTTGAAATGACGCTCGA
ACAGGCATGCCCGCCGGAATACCAGCGGGCGCAATGTGCGTTCAAAGATTCGATGATTCAC
TGAATTCTGCAATTCACATTACTTATCGCATTTTGCTGCGTTCTTCATCGATGCCAGAACCAA
GAGATCCGTTGTTGAAAGTTTTGATTTATTTGTTTGTTTTACTCAGAAGTTACAATAAGAAAC
ATTAGAGTTTGGGTCCTCTGGCGGGCCGTCCCGTTTTACGGGGCGCGGGCTGATCCGCCGAG
GCAACATTAAGGTATGTTCACAGGGGTTTGGGAGTTGTAAACTCGGTAATGATCCCTCCGCT
GGTTCACCAACGGAGACCTTGTTACGACTTCTCC

>BSNB_0687_Diaporthe_sp

TGATCCGAGGTCAAATTTCAGAAGTTGGGGGTTTAACGGCAGGGCGCCGCCAGGGCCTTCC
AGAACGAGATGTAACTACTACGCTCGGGGTCCTAGCGAGCTCGCCACTAGATTTCAGGGCC
TGCTTTGTCTCCAAAGCAGTGCCCCAACACCAAGCCAGGCTTGAGGGTTGAAATGACGCTCG
AACAGGCATGCCCTCCGGAATACCAGAGGGCGCAATGTGCGTTCAAAGATTCGATGATTCA
CTGAATTCTGCAATTCACATTACTTATCGCATTTCGCTGCGTTCTTCATCGATGCCAGAACCA
AGAGATCCGTTGTTGAAAGTTTTGATTCATTTATGTTTTTTACTCAGAGATTCACTAAGAAAC
AAGAGTTTGGTTGGCCGCCGGCGGGCTGCTCCCCGTTTCCGGGGGGCCTCAGAAGAGGCCG
GCCTTCGCCGAGGCAACAATAAGGTATAAGTTCACAAAGGGTTTCTGGGTGCGCCGGGGCG
CGTTCCAGCAATGATCCCTCCGCTGGTTCACCAACGGAGACCTTGTTACG

>BSNB_0688_Colletotrichum_gloeosporioides

CTGATCCGAGGTCAACCTTTGGAAAATTGGGGGGTTTACGGCAAGAGTCCCTCCGGATCCC
AGTGCGAGACGTAAAGTTACTACGCAAAGGAGGCTCCGGGAGGGTCCGCCACTACCTTTGA
GGGCCTACATCAGCTGTAGGGCCCCAACACCAAGCAGAGCTTGAGGGTTGAAATGACGCTC
GAACAGGCATGCCCGCCAGAATGCTGGCGGGCGCAATGTGCGTTCAAAGATTCGATGATTC
ACTGAATTCTGCAATTCACATTACTTATCGCATTTCGCTGCGTTCTTCATCGATGCCAGAACC
AAGAGATCCGTTGTTAAAAGTTTTGATTATTTGCTTGTACCACTCAGAAGAAACGTCGTTAA
ATCAGAGTTTGGTTATCCTCCGGCGGGCGCCGACCCGCCCGGGGGGGCGGGAGGCCGGGAG
GGTCACGGGGACCCTACCCGCCGAAGCAACAGTTGTAGGTATGTTCACAAAGGGTTGTAGA
GCGTAAACTCAGTAATGATCCCTCCGCTGGTTCACCAACGGAGACCTTGTTA

>BSNB_0691_Penicillium_verrucosum

ACAAAGCCCCATACGCTCGAGGACCGGACGCGGTGCCGCCGCTGCCTTTCGGGCCCGTCCC
CCGGAATCGGAGGACGGGGCCCAACACACAAGCCGGGCTTGAGGGCAGCAATGACGCTCG
GACAGGCATGCCCCCCGGAATACCAGGGGGCGCAATGTGCGTTCAAAGACTCGATGATTCA
CTGAATTTGCAATTCACATTACGTATCGCATTTCGCTGCGTTCTTCATCGATGCCGGAACCAA
GAGATCCGTTGTTGAAAGTTTTAAATAATTTATATTTTCACTCAGACTTCAATCTTCAGACAG
AGTTCGAGGGTGTCTTCGGCGGGCGCGGGCCCGGGGGCGTGAGCCCCCCGGCGGCCAGTAA
AGGCGGGCCCGCCGAAGCAACAAGGTAAAATAAACACGGGTGGGAGGTTGGACCCAGAGG
GCCCTCACTCGGTAATGATCCTTCCGCAGGTTCACCTACGGAAACCTTGTTACGACTTCTCC

>BSNB_0692_Endomelanconiopsis_endophytica

TACCTGATCCGAGGTCAACCTTGAGAAAATTCAGAAGGTTCGTCCGGCGAGCGAACGTCCC
GTCCTCCAAAGCGAGGTGTTTTCTACTACGCTTGAGGCAAGACGCCACCGCCGAGGTCTTTG
AGGCGCGTCCGGTGAAGGACGGCGCCCAATACCAAGCAGAGCTTGAGTGGTGAAATGACG
CTCGAACAGGCATGCCCCTCGGAATACCAAGGGGCGCAATGTGCGTTCAAAGATTCGATGA
TTCACTGAATTCTGCAATTCACATTACTTATCGCATTTCGCTGCGTTCTTCATCGATGCCAGA
ACCAAGAGATCCGTTGTTGAAAGTTTTAGTTTATTAAACTTTTGATCAGACAGCTACGTTTAC
TGACTGGAGTTTGGTAGTCCTCTGGCGGGCGCTGGCCAGCCCCGGTTAGGGGGCCGGCCGCG
GAGGACCGCGGCCCGCCAAAGCAACAGAGGTAGATACACATAGGGTGTGAGAGAAGAGGT
CCGGAGACCCCTAGAACTCGGTAATGATCCTTCCGCAGGTTCACCTACGGAAACCTTGTTAC
GAACTTCTCC

>BSNB_0693_Endomelanconiopsis_endophytica

TACCTGATCCGAGGTCAACCTTGAGAAAATTCAGAAGGTTCGTCCGGCGAGCGAACGTCCC
GTCCTCCAAAGCGAGGTGTTTTCTACTACGCTTGAGGCAAGACGCCACCGCCGAGGTCTTTG
AGGCGCGTCCGGTGAAGGACGGCGCCCAATACCAAGCAGAGCTTGAGTGGTGAAATGACG
CTCGAACAGGCATGCCCCTCGGAATACCAAGGGGCGCAATGTGCGTTCAAAGATTCGATGA
TTCACTGAATTCTGCAATTCACATTACTTATCGCATTTCGCTGCGTTCTTCATCGATGCCAGA
ACCAAGAGATCCGTTGTTGAAAGTTTTAGTTTATTAAACTTTTGATCAGACAGCTACGTTTAC
TGACTGGAGTTTGGTAGTCCTCTGGCGGGCGCTGGCCAGCCCCGGTTAGGGGGCCGGCCGCG
GAGGACCGCGGCCCGCCAAAGCAACAGAGGTAGATACACATAGGGTGTGAGAGAAGAGGT
CCGGAGACCCCTAGAACTCGGTAATGATCCTTCCGCAGGTTCACCTACGGAAACCTTGTTAC
GAACTTCTCC

>BSNB_0694_Colletotrichum_theobromicola

CTGATCCGAGGTCAACCTTTGGAAAATTGGGGGGTTTACGGCAAGAGTCCCTCCGGATCCC

AGTGCGAGACGTAAAGTTACTACGCAAAGGAGGCTCCGGGAGGGTCCGCCACTACCTTTGA
GGGCCTACATCAGCTGTAGGGCCCCAACACCAAGCAGAGCTTGAGGGTTGAAATGACGCTC
GAACAGGCATGCCCGCCAGAATGCTGGCGGGCGCAATGTGCGTTCAAAGATTCGATGATTC
ACTGAATTCTGCAATTCACATTACTTATCGCATTTCGCTGCGTTCTTCATCGATGCCAGAACC
AAGAGATCCGTTGTTAAAAGTTTTGATTATTTGCTTGTACCACTCAGAAGAAACGTCGTTAA
ATCAGAGTTTGGTTATCCTCCGGCGGGCGCCGACCCGCCCGGGGGGGCGGGAGGCCGGGAG
GGTCACGGGGACCCTACCCGCCGAAGCAACAGTTGTAGGTATGTTCACAAAGGGTTGTAGA
GCGTAAACTCAGTAATGATCCCTCCGCTGGTTCACCAACGGAGACCTTGTTA

>BSNB_0695_Colletotrichum_theobromicola

TGATCCGAGGTCAACCTTTGGAAAATTGGGGGGTTTTACGGCAAGAGTCCCTCCGGATCCCA
GTGCGAGACGTAAAGTTACTACGCAAAGGAGGCTCCGGGAGGGTCCGCCACTACCTTTGAG
GGCCTACATCAGCTGTAGGGCCCCAACACCAAGCAGAGCTTGAGGGTTGAAATGACGCTCG
AACAGGCATGCCCGCCAGAATGCTGGCGGGCGCAATGTGCGTTCAAAGATTCGATGATTCA
CTGAATTCTGCAATTCACATTACTTATCGCATTTCGCTGCGTTCTTCATCGATGCCAGAACCA
AGAGATCCGTTGTTAAAAGTTTTAATTATTTGCTTGTACCACTCAGAAGAAACGTCGTTAAAT
CAGAGTTTGGTTATCCTCCGGCGGGCGCCGACCCGCCCGGGGGGGCGGGAGGCCGGGAGGG
TCACGGGGACCCTACCCGCCGAAGCAACAGTTGTAGGTATGTTCACAAAGGGTTGTAGAGC
GTAAACTCAGTAATGATCCCTCCGCTGGTTCACCAACGGAGACC

>BSNB_0696_Colletotrichum_gloeosporioides

GGAGAAGTCGTAACAAGGTCTCCGTTGGTGAACCAGCGGAGGGATCATTACTGAGTTTACG
CTCTATAACCCTTTGTGAACATACCTATAACTGTTGCTTCGGCGGGTAGGGTCTCCGCGACCC
TCCCGGCCTCCCGCCTCCGGGCGGGTCGGCGCCCGCCGGAGGATAACCAAACTCTGATTTAA
CGACGTTTCTTCTGAGTGGTACAAGCAAATAATCAAAACTTTTAACAACGGATCTCTTGGTTC
TGGCATCGATGAAGAACGCAGCGAAATGCGATAAGTAATGTGAATTGCAGAATTCAGTGAA
TCATCGAATCTTTGAACGCACATTGCGCCCGCCAGCATTCTGGCGGGCATGCCTGTTCGAGC
GTCATTTCAACCCTCAAGCTCTGCTTGGTGTTGGGGCCCTACAGCTGATGTAGGCCCTCAAA
GGTAGTGGCGGACCCTCCCGGAGCCTCCTTTGCGTAGTAACTTTACGTCTCGCACTGGGATC
CGGAGGGACTCTTGCCGTAAAACCCCCCAATTTTCCAAAGGTTGACCTCGGATCAGGTAG

>BSNB_0699_Colletotrichum_theobromicola

CTGATCCGAGGTCAACCTTTGGAAAATTGGGGGGTTTTACGGCAAGAGTCCCTCCGGATCCC
AGTGCGAGACGTAAAGTTACTACGCAAAGGAGGCTCCGGGAGGGTCCGCCACTACCTTTGA
GGGCCTACATCAGCTGTAGGGCCCCAACACCAAGCAGAGCTTGAGGGTTGAAATGACGCTC
GAACAGGCATGCCCGCCAGAATGCTGGCGGGCGCAATGTGCGTTCAAAGATTCGATGATTC
ACTGAATTCTGCAATTCACATTACTTATCGCATTTCGCTGCGTTCTTCATCGATGCCAGAACC
AAGAGATCCGTTGTTAAAAGTTTTGATTATTTGCTTGTACCACTCAGAAGAAACGTCGTTAA
ATCAGAGTTTGGTTATCCTCCGGCGGGCGCCGACCCGCCCGGGGGGGCGGGAGGCCGGGAG
GGTCACGGGGACCCTACCCGCCGAAGCAACAGTTGTAGGTATGTTCACAAAGGGTTGTAGA
GCGTAAACTCAGTAATGATCCCTCCGCTGGTTCACCAACGGAGACCTTGTTA

>BSNB_0700_Apioclypea_sp

CCTGATCCGAGGTCAACCCAGTAAAAAAGTTGGGGGTTTAGCGGCGAGGGCCACGGCACCT
ACAGAAGCGAGAGGTATATTACTGCGCTCAGAGGTAGAACCGTCACTCCGCCGGTGAATTT
GAGGAGCTACAGGGTAACAGCTACAGGGTAGCTCCCGGGTAGTCTCCCAACGCCAAGACTA
GGTCTTGAGTGGTACTAATGACGCTCGAACAGGCATGCCCTCCAGAATGCTGGGGGGCGCA
ATGTGCGTTCAAAGATTCGATGATTCACTGAATTCTGCAATTCACATTACTTATCGCATTTCG

CTGCGTTCTTCATCGATGCCAGAACCAAGAGATCCGTTGTTAAAGGTTTTGACTTATTTTCAT
AAGACTCTCAGATGATAATAATACAAATAGTTTGGTTTTCCACCGGCGGTCCGCCCGCTACA
GGGTAAGCTGCAGGGTAAGACCGCCGAGGCAACGTAAGGTATAGGTTCACAAATGGTTTAT
ACAGGAGTGTTTTAATACTCTGTAATGATCCCTCCGCTGGTTCACCAACGGAGACCTTGTTAC
GAACTTCTCCT

>BSNB_0701_Colletotrichum_gloeosporioides

CTGATCCGAGGTCAACCTTTGGAAAATTGGGGGGTTTTACGGCAAGAGTCCCTCCGGATCCC
AGTGCGAGACGAAATGTTACTACGCAAAGGAGGCTCCGGGAGGGTCCGCCACTACCTTTGA
GGGCCTACGTCGACCGTAGAGCCCCAACACCAAGCAGAGCTTGAGGGTTGAAATGACGCTC
GAACAGGCATGCCCGCCAGAATGCTGGCGGGCGCAATGTGCGTTCAAAGATTCGATGATTC
ACTGAATTCTGCAATTCACATTACTTATCGCATTTCGCTGCGTTCTTCATCGATGCCAGAACC
AAGAGATCCGTTGTTAAAAGTTTTGATTATTTTGCTTATGCCACTCAGAAGAAACGTCGTTAC
AATAGAGTTTGGTTATCCTCCGGCGGGCGCCGGGTCCGGTCCCGCGGGGGGTCCGGTCCGGG
CCGGGAGGCGTCCTTTTCAGGGGACGGCCTACCCGCCGAAGCAACAGTTGTAGGTATGTTCA
CAAAGGGTTATAGAGCGGTAACTCAGTAATGATCCCTCCGCTGGTTCACCAACGGAGACCTT
GTTACGACTTCTCC

>BSNB_0702_Fusarium_lateritium

AGGAGAAGTCGTAACAAGGTCTCCGTTGGTGAACCAGCGGAGGGATCATTACCGAGTTTAC
AACTCCCAAACCCCTGTGAACATACCTTAATGTTGCCTCGGCGGATCAGCCCGCGCCCCGTA
AAACGGGACGGCCCGCCAGAGGACCCAAACTCTAATGTTTCTTATTGTAACTTCTGAGTAAA
ACAAACAAATAAATCAAAACTTTCAACAACGGATCTCTTGGTTCTGGCATCGATGAAGAAC
GCAGCAAAATGCGATAAGTAATGTGAATTGCAGAATTCAGTGAATCATCGAATCTTTGAAC
GCACATTGCGCCCGCTGGTATTCCGGCGGGCATGCCTGTTCGAGCGTCATTTCAACCCTCAA
GCCCTCGGGTTTGGTGTTGGGGATCGGCTCTGCCTTCTGGCGGTGCCGCCCCCGAAATACATT
GGCGGTCTCGCTGCAGCCTCCATTGCGTAGTAGCTAACACCTCGCAACTGGAACGCGGCGC
GGCCATGCCGTAAAACCCCAACTTCTGAATGTTGACCTCGGATCAGGTAG

>BSNB_0703_Colletotrichum_theobromicola

CTGATCCGAGGTCAACCTTTGGAAAATTGGGGGGTTTTACGGCAAGAGTCCCTCCGGATCCC
AGTGCGAGACGTAAAGTTACTACGCAAAGGAGGCTCCGGGAGGGTCCGCCACTACCTTTGA
GGGCCTACATCAGCTGTAGGGCCCCAACACCAAGCAGAGCTTGAGGGTTGAAATGACGCTC
GAACAGGCATGCCCGCCAGAATGCTGGCGGGCGCAATGTGCGTTCAAAGATTCGATGATTC
ACTGAATTCTGCAATTCACATTACTTATCGCATTTCGCTGCGTTCTTCATCGATGCCAGAACC
AAGAGATCCGTTGTTAAAAGTTTTGATTATTTGCTTGTACCACTCAGAAGAAACGTCGTTAA
ATCAGAGTTTGGTTATCCTCCGGCGGGCGCCGACCCGCCCGGGGGGGCGGGAGGCCGGGAG
GGTCACGGGGACCCTACCCGCCGAAGCAACAGTTGTAGGTATGTTCACAAAGGGTTGTAGA
GCGTAAACTCAGTAATGATCCCTCCGCTGGTTCACCAACGGAGACCTTGTTA

>BSNB_0707_Fusarium_lateritium

AGGAGAAGTCGTAACAAGGTCTCCGTTGGTGAACCAGCGGAGGGATCATTACCGAGTTTAC
AACTCCCAAACCCCTGTGAACATACCTTAATGTTGCCTCGGCGGATCAGCCCGCGCCCCGTA
AAACGGGACGGCCCGCCAGAGGACCCAAACTCTAATGTTTCTTATTGTAACTTCTGAGTAAA
ACAAACAAATAAATCAAAACTTTCAACAACGGATCTCTTGGTTCTGGCATCGATGAAGAAC
GCAGCAAAATGCGATAAGTAATGTGAATTGCAGAATTCAGTGAATCATCGAATCTTTGAAC
GCACATTGCGCCCGCTGGTATTCCGGCGGGCATGCCTGTTCGAGCGTCATTTCAACCCTCAA
GCCCTCGGGTTTGGTGTTGGGGATCGGCTCTGCCTTCTGGCGGTGCCGCCCCCGAAATACATT

GGCGGTCTCGCTGCAGCCTCCATTGCGTAGTAGCTAACACCTCGCAACTGGAACGCGGCGC
GGCCATGCCGTAAAACCCCAACTTCTGAATGTTGACCTCGGATCAGGTAG

>BSNB_0717_Harknessia_australiensis

CCTGATCCGAGGTCAATTTTCAGAAGTTGGGGGGTTTTACGGCCGGGACACCGCTAATCCTT
CCAAAGCGAGGTGAGAAAAAACTACTACGCTCAGAGTCTTAGCGAGCCCGCCACTGAATTT
CAGGGCCTACCGTGTAACGGGTAGTGCCCCAACACCAAGCCAGGCTTGAGGGTTGAAATGA
CGCTCGAACAGGCATGCCCGCTGGAATTCCAGCGGGCGCAATGTGCGTTCAAAGATTCGAT
GATTCACTGAATTCTGCAATTCACATTACTTATCGCATTTCGCTGCGTTCTTCATCGATGCCA
GAACCAAGAGATCCGTTGTTGAAAGTTTTGATTCATTTGTAAAAAATGAGACTCAGAAGAG
ATACGTTATAAAAACAAAGAGTTTAGTGGCCGCCGGCGGGCCTGCTCCGGACGCGACCGA
GAAAGGACACGACCGGCAGGGGGCGCGAGGGCCCCCCGAGCTCGACGCCGAGGCAACGAT
GTGTGGGTATAAGTTCACAAAGGGTGTCTGGGAGTGCGCCCGCGAGGGACGCAGTTCCAAC
AATGATCCCTCCGCTGGTTCACCAACGGAGACCTTGTTACGAACTTCTCC

>BSNB_0718_Xylaria_sp

GATCCGAGGTCAACCTTTAAAAAGTAGGGGTTTTACGGCAGGGGACCGGTCCAACTAATAG
GCGAGATAAAATCTACTACGTCTAGAGTGTGAACCGACTCCGCCACTAACTTTGAGGGGCT
ACAGTGCTGTAGGCCCCCAACACTAAGCAACAGAGGCTTAAGGGTTGAAATGACGCTCGAA
CAGGCATGCCCACTAGAATACTAATGGGCGCAATGTGCGTTCAAAGATTCGATGATTCACTG
AATTCTGCAATTCACATTACTTATCGCATTTCGCTGCGTTCTTCATCGATGCCAGAACCAAGA
GATCCGTTGTTGAAAGTTTTAACTTATTTAGTTATACTGTCAGAATTCAATAATAAACAGAGT
TTCGTGGGCCGCCGGCAGGTCATCGTTCCCTGCAAGGGTATTATTATGCAGTAAGGCTGTAA
CACTCGCATAGGAGGGTACGTTTATAAAGCTTTTATCCCCTATAGAAACCGATGCTGGTGTC
TATCGAGTAGCACTACTGAGCAGGCGTCCTCGGTCCCCGCAAGGGTATTATGCAGTAAAGTT
GTAACACTCGCATAGGAGGTACGGTCTTAAAGCTTTTCTCCTTACAAGGGGATTATGCAGTC
AGCTGTAACACTCGCATCGGAGGTACGTTCGTAAAGCATTATCCCCTATTGGAACCGAGTAG
CACTACTGAGCAGACGTCCTCGGTCCCCGCAAGGGTATTATGCAGTAAAGTTGTAACACTCG
CATAGGAGGTACGGTCTTAAAGCTTTTCTCCTCACAAGGGGATTATGCAGTCAGTTGTAACA
CTCGCATAGGAGGGTACGTTCGTAAAGCATTATCCCCTATTGGAACCGAAGGTCCTACAGGG
TAGGGCACTACTGCGCAGGCGTGACCTGCCGAGGC

>BSNB_0720_Oxydothis_sp

GGTGTTTTACGGCAGGGGCCGCGGCGCCTCACAGAGCGAAAAAGAGAACTTACTACGCTCA
GAGGACACACACGTTCCCGCCACTGGATTTGGGGAACCGCGGGGCAGCCCGCGGATTCCCA
ACACTAAGCTGTGCTTAAGGGTTGAAATGACGCTCGAACAGGCATGCCCGCCAGAATGCTG
ACGGGCGCAATGTGCGTTCAAAGATTCGATGATTCACTGAATTCTGCAATTCACATTACTTAT
CGCATTTCGCTGCGTTCTTCATCGATGCCAGAACCAAGAGATCCGTTGTTGAAAGTTTTGACT
TATTTAATAAGACGCTCAGATGTCCACTAAAATACAAGAGTTTAGGAGTCCACCGGCGGCG
CCTGCGGGGCGGGCCGCGCCGTCCGCTACAGGGTAGCTTCAGGGTAGGCCGCGCCCGACAT
GGCGTC

>BSNB_0731_Endomelanconiopsis_endophytica

TACCTGATCCGAGGTCAACCTTGAGAAAATTCAGAAGGTTCGTCCGGCGAGCGAACGTCCC
GTCCTCCAAAGCGAGGTGTTTTCTACTACGCTTGAGGCAAGACGCCACCGCCGAGGTCTTTG
AGGCGCGTCCGGTGAAGGACGGCGCCCAATACCAAGCAGAGCTTGAGTGGTGAAATGACG
CTCGAACAGGCATGCCCCTCGGAATACCAAGGGGCGCAATGTGCGTTCAAAGATTCGATGA
TTCACTGAATTCTGCAATTCACATTACTTATCGCATTTCGCTGCGTTCTTCATCGATGCCAGA

ACCAAGAGATCCGTTGTTGAAAGTTTTAGTTTATTAAACTTTTGATCAGACAGCTACGTTTAC
TGACTGGAGTTTGGTAGTCCTCTGGCGGGCGCTGGCCAGCCCCGGTTAGGGGGCCGGCCGCG
GAGGACCGCGGCCCGCCAAAGCAACAGAGGTAGATACACATAGGGTGTGAGAGAAGAGGT
CCGGAGACCCCTAGAACTCGGTAATGATCCTTCCGCAGGTTCACCTACGGAAACCTTGTTAC
GAACTTCTCC

>BSNB_0732_Akanthomyces_attenuatus

CTGATCCGAGGTCACGTTCAGAAGTTGGGTGTTTTACGGCGTGGCCACGTCGGGGTTCCGGT
GCGAGTTGGATTACTACGCAGAGGTCGCCGCGGACGGGCCGCCACTCCATTTCGGGGCCGG
CGGTATGCTGCCGTTCCCCAACGCCGATTTCCCCAAAGGGAAGTCGAGGGTTGAAATGACG
CTCGAACAGGCATGCCCGCCAGAATGCTGGCGGGCGCAATGTGCGTTCAAAGATTCGATGA
TTCACTGAATTCTGCAATTCACATTACTTATCGCATTTCGCTGCGTTCTTCATCGATGCCAGA
ACCAAGAGATCCGTTGTTGAAAGTTTTGATTCATTTGTTTTGCCTTGCGGCGGATTCAGAAAA
TGCTGATAATACAGAGTTTAGGGGTCTCCGGCGGCCGCCTGGGTCCGGGCCGCGGGCGGCG
CGAGGCCGTCCGGACGCCGGGGCGAGTCCGCCGAAGCAACAGTAGGTATGTTCACATAAGG
GTTTGGGAGTTGTAAACTCGATAATGATCCCTCCGCTGGTTCACCAACGGAGACCTTGTTAC
GACTTCTCCT

>BSNB_0733_ Endomelanconiopsis_endophytica

ACCTGATCCGAGGTCAACCTTGAGAAAATTCAGAAGGTTCGTCCGGCGAGCGAACGTCCCG
TCCTCCAAAGCGAGGTGTTTTCTACTACGCTTGAGGCAAGACGCCACCGCCGAGGTCTTTGA
GGCGCGTCCGGTGAAGGACGGCGCCCAATACCAAGCAGAGCTTGAGTGGTGAAATGACGCT
CGAACAGGCATGCCCCTCGGAATACCAAGGGGCGCAATGTGCGTTCAAAGATTCGATGATT
CACTGAATTCTGCAATTCACATTACTTATCGCATTTCGCTGCGTTCTTCATCGATGCCAGAAC
CAAGAGATCCGTTGTTGAAAGTTTTAGTTTATTAAACTTTTGATCAGACAGCTACGTTTACTG
ACTGGAGTTTGGTAGTCCTCTGGCGGGCGCTGGCCAGCCCCGGTTAGGGGGCCGGCCGCGG
AGGACCGCGGCCCGCCAAAGCAACAGAGGTAGGTACACATAGGGTGTGAGAGAAGAGGTC
CGAAGACCCCTAGAACTCGGTAATGATCCTTCCGCAGGTTCACCTACGGAAACCTTGTTACG
AACTTCTCCT

>BSNB_1019_Colletotrichum_gloeosporioides

CTGATCCGAGGTCAACCTTTGGAAAATTGGGGGGTTTTACGGCAAGAGTCCCTCCGGATCCC
AGTGCGAGACGTAAAGTTACTACGCAAAGGAGGCTCCGGGAGGGTCCGCCACTACCTTTGA
GGGCCTACATCAGCTGTAGGGCCCCAACACCAAGCAGAGCTTGAGGGTTGAAATGACGCTC
GAACAGGCATGCCCGCCAGAATGCTGGCGGGCGCAATGTGCGTTCAAAGATTCGATGATTC
ACTGAATTCTGCAATTCACATTACTTATCGCATTTCGCTGCGTTCTTCATCGATGCCAGAACC
AAGAGATCCGTTGTTAAAAGTTTTGATTATTTGCTTGTACCACTCAGAAGAAACGTCGTTAA
ATCAGAGTTTGGTTATCCTCCGGCGGGCGCCGACCCGCCCGGGGGGGCGGGAGGCCGGGAG
GGTCACGGGGACCCTACCCGCCGAAGCAACAGTTGTAGGTATGTTCACAAAGGGTTGTAGA
GCGTAAACTCAGTAATGATCCCTCCGCTGGTTCACCAACGGAGACCTTGTTA

>BSNB_1021_Colletotrichum_theobromicola

CTGATCCGAGGTCAACCTTTGGAAAATTGGGGGGTTTTACGGCAAGAGTCCCTCCGGATCCC
AGTGCGAGACGTAAAGTTACTACGCAAAGGAGGCTCCGGGAGGGTCCGCCACTACCTTTGA
GGGCCTACATCAGCTGTAGGGCCCCAACACCAAGCAGAGCTTGAGGGTTGAAATGACGCTC
GAACAGGCATGCCCGCCAGAATGCTGGCGGGCGCAATGTGCGTTCAAAGATTCGATGATTC
ACTGAATTCTGCAATTCACATTACTTATCGCATTTCGCTGCGTTCTTCATCGATGCCAGAACC
AAGAGATCCGTTGTTAAAAGTTTTGATTATTTGCTTGTACCACTCAGAAGAAACGTCGTTAA

ATCAGAGTTTGGTTATCCTCCGGCGGGCGCCGACCCGCCCGGGGGGGCGGGAGGCCGGGAG
GGTCACGGGGACCCTACCCGCCGAAGCAACAGTTGTAGGTATGTTCACAAAGGGTTGTAGA
GCGTAAACTCAGTAATGATCCCTCCGCTGGTTCACCAACGGAGACCTTGTTA

>BSNB_1036_Pestalotiopsis_sp

CCATGTGACTTACCTTTTGTTGCCTCGGCAGAAGTTATAGGTCTTCTTATAGCTGCTGCCGGT
GGACCATTAAACTCTTGTTATTTTATGTAATCTGAGCGTCTTATTTTAATAAGTCAAAACTTTC
AACAACGGATCTCTTGGTTCTGGCATCGATGAAGAACGCAGCGAAATGCGATAAGTAATGT
GAATTGCAGAATTCAGTGAATCATCGAATCTTTGAACGCACATTGCGCCCATTAGTATTCTA
GTGGGCATGCCTGTTCGAGCGTCATTTCAACCCTTAAGCCTAGCTTAGTGTTGGGAATCTACT
TCTCTTAGGAGTTGTAGTTCCTGAAATACAACGGCGGATTTGTAGTATCCTCTGAGCGTAGTA
ATTTTTTTCTCGCTTTTGTTAGGTGCTATAACTCCCAGCCGCTAAACCCCCAATTTTTTGTGGT
TGACCTCGGATCAGGTAGGAATACCCGCTGAACTTAAGCATATCARAA
>BSNB_1037_Pestalotiopsis_sp

AACCCATGTGAACTTACCTTTTGTTGCCTCGGCAGAAGTTATAGGTCTTCTTATAGCTGCTGC
CGGTGGACCATTAAACTCTTGTTATTTTATGTAATCTGAGCGTCTTATTTTAATAAGTCAAAA
CTTTCAACAACGGATCTCTTGGTTCTGGCATCGATGAAGAACGCAGCGAAATGCGATAAGTA
ATGTGAATTGCAGAATTCAGTGAATCATCGAATCTTTGAACGCACATTGCGCCCATTAGTAT
TCTAGTGGGCATGCCTGTTCGAGCGTCATTTCAACCCTTAAGCCTAGCTTAGTGTTGGGAATC
TACTTCTCTTAGGAGTTGTAGTTCCTGAAATACAACGGCGGATTTGTAGTATCCTCTGAGCGT
AGTAATTTTTTTCTCGCTTTTGTTAGGTGCTATAACTCCCAGCCGCTAAACCCCCAATTTTTTG
TGGTTGACCTCGGATCAGGTAGGAATACCCGCTGAACTTAAGCATATCAATAA
>BSNB_1038_Beauveria_bassiana

CTGTGAACCTACCTATCGTTGCTTCGGCGGACTCGCCCCAGCCCGGAGGCGGACTGGACCAG
CGGCCCGCCGGGGACCWCAAACTCTTGTATTCCAGCATCTTCTGAATACGCCGCAAGGCAA
AACAAATGAATCAAAACTTTCAACAACGGATCTCTTGGCTCTGGCATCGATGAAGAACGCA
GCGAAATGCGATAAGTAATGTGAATTGCAGAATCCAGTGAATCATCGAATCTTTGAACGCA
CATTGCGCCCGCCAGCATTCTGGCGGGCATGCCTGTTCGAGCGTCATTTCAACCCTCGACCTC
CCCTTGGGGAGGTCGGCGTTGGGGACCGGCAGCACACCGCCGGCCCTGAAATGGAGTGGCG
GCCCGTCCGCGGCGACCTCTGCGTAGTAATACAGCTCGCACCGGAACCCCGACGCGGCCAC
GCCGTAAAACACCCAACTTCTGAACGTTGACCTCGAATCAGGTAGGACTACCCGCTGAACTT
AAGCATATCAATAAGCGGAGGAA

>BSNB_1040_Trichoderma_sp

CTCCCAACCCAATGTGACCATACCAAACTGTTGCCTCGGCGGGGTCACGCCCCGGGTGCGTC
GCAGCCCCGGAACCAGGCGCCCGCCGGAGGGACCAACCAAACTCTTTCTGTAGTCCCCTCG
CGGACGTTATTTCTTACAGCTCTGAGCAAAAATTCAAAATGAATCAAAACTTTCAACAACGG
ATCTCTTGGTTCTGGCATCGATGAAGAACGCAGCGAAATGCGATAAGTAATGTGAATTGCAG
AATTCAGTGAATCATCGAATCTTTGAACGCACATTGCGCCCGCCAGTATTCTGGCGGGCATG
CCTGTCCGAGCGTCATTTCAACCCTCGAACCCCTCCGGGGGGTCGGCGTTGGGGATCGGGAA
CCCCTAAGACGGGATCCCGGCCCCGAAATACAGTGGCGGTCTCGCCGCAGCCTCTCCTGCG
CAGTAGTTTGCACAACTCGCACCGGGAGCGCGGCGCGTCCACGTCCGTAAAACACCCAACT
TCTGAAATGTTGACCTCGGATCAGGTAGGAATACCCGCTGAACTTAAGCATATCAA
>BSNB_1041_Beauveria_bassiana

CCCCTAAATCCCTTCTGTGACCTACCTATCGTTGCTTCGGCGGACTCGCCCCAGCCCGGACGC
GGACTGGACCAGCGGCCCGCCGGGGACCTCAAACTCTTGTATTCCAGCATCTTCTGAATACG
CCGCAAGGCAAAACAAATGAATCAAAACTTTCAACAACGGATCTCTTGGCTCTGGCATCGA

TGAAGAACGCAGCGAAATGCGATAAGTAATGTGAATTGCAGAATCCAGTGAATCATCGAAT
CTTTGAACGCACATTGCGCCCGCCAGCATTCTGGCGGGCATGCCTGTTCGAGCGTCATTTCA
ACCCTCGACCTCCCCTTGGGGAGGTCGGCGTTGGGGACCGGCAGCACACCGCCGGCCCTGA
AATGGAGTGGCGGCCCGTCCGCGGCGACCTCTGCGTAGTAATACAGCTCGCACCGGAACCC
CGACGCGGCCACGCCGTAAAACACCCAACTTCTGAACGTTGACCTCGAATCAGGTAGGACT
ACCCGCTGAACTTAAGCATATCAATAAGCGGAGGAA

>BSNB_1042_Beauveria_bassiana

TCTGTGAGCCTACCTATCGTTGCTTCGGCGGACTCGCCCCAGCCCGGACGCGGACTGGACCA
GCGGCCCGCCGGGGACCTCAAACTCTTGTATTCCAGCATCTTCTGAATACGCCGCAAGGCAA
AACAAATGAATCAAAACTTTCAACAACGGATCTCTTGGCTCTGGCATCGATGAAGAACGCA
GCGAAATGCGATAAGTAATGTGAATTGCAGAATCCAGTGAATCATCGAATCTTTGAACGCA
CATTGCGCCCGCCAGCATTCTGGCGGGCATGCCTGTTCGAGCGTCATTTCAACCCTCGACCTC
CCCTTGGGGAGGTCGGCGTTGGGGACCGGCAGCACACCGCCGGCCCTGAAA
TGGAGTGGCGGCCCGTCCGCGGCGACCTCTGCGTAGTAATACAGCTCGCACCGGAACCCCG
ACGCGGCCACGCCGTAAAACACCCAACTTCTGAACGTTGACCTCGAATCAGGTAGGACTAC
CCGCTGAACTTAAGCATATCAATAAGGCGGAGGAA
>BSNB_1043_Mucor_sp

CCGTATTTCTTATTTACTGTGAACTGTTTTATTGTTTGRCGCTTGAGGAATGTTGTTTAGCCAT
AGGGATAGGCTAGGCAAATGTTAACCGAGTCAAAGTCAGGCTTAGGCCTGGTATCCTATTC
ATTATTTACCAAAAGAATTCAGAATTATTATTGTAACATAAGCGTAAAAAACTTATAAAACA
ACTTTTAACAACGGATCTCTTGGTTCTCGCATCGATGAAGAACGTAGCAAAGTGCGATAACT
AGTGTGAATTGCATATTCAGTGAATCATCGAGTCTTTGAACGCATCTTGCGCTCAATGGTATT
CCATTGAGCACGCCTGTTTCAGTATCAAAAACACCCCACATTCACAACTTGTTTGTTGAATG
GAATTGAGAGTTTCGGCTTAACGCTGATCTCTTTAAAAWTATTAGGCCTGAACTTTTGTTCTT
TYYGCCTGAACATTTTTTTAATATAAAGGAATGCTCTAGTAAAAAGACTCTTTCTGGGGCCTC
CCAAATAAATCATTCTTAAACTTGATCTGAAATCAGGTGGGATTACCCGCTGAACTTAAGCA
TATCATAAGCCGGAGGA
>BSNB_1044_Penicillium_sp

AAAGCTGTACGGCGAGTGAGCGGCAAGAGCTCAAATTTGARRGCTGGCTCCTTCGGGGTCC
GCATTGTAATTTGCAGAGGATGCTTCGGGAGTGGCCCCCATCTAAGTGCCCTGGAACGGGCC
GTCATAGAGGGTGAGAATCCCGTCTGGGATGGGGTGTCCACGCCCGTGTGAAGCTCCTTCGA
CGAGTCGAGTTGTTTGGGAATGCAGCTCTAAATGGGTGGTAAATTTCATCTAAAGCTAAATA
CTGGCCGGAGACCGATAGCGCACAAGTAGAGTGATCGAAAGATGAAAAGCACTTTGAAAA
GAGAGTTAAACAGCACGTGAAATTGTTGAAAGGGAAGCGCTTGCGACCAGACTCGCCCACG
GGGTTCAGCCGGCATTCGTGCCGGTGTACTTCCCCGCGGGCGGGCCAGCGTCGGTTTGGGCG
GCCGGTCAAAGGCCCTCGGAATGTAACGCCCCCCGGGGCGTCTTATAGCCGAGGGTGCCAT
GCGGCCAGCCCGGACCGAGGAACGCGCTTCGGCTCGGACGCTGGCATAATGGTCGTAAGCG
ACCCGTCTTGAAACACGGACCAAA

>BSNB_1045_Penicillium_sp

CCACCTCCCACCCTTGTCTCCACACCTGTTGCTTCGGCGGGCCCACCGGGGCCACCCGGTCG
CCGGGGGACATCCGTCCCCGGGCCCGCGCCCGCCGAGGCGCTCTGTGAACCCTGATGAAGA
TGGGCTGTCTGAGTGATATGAAAATTGTCAAAACTTTCAACAATGGATCTCTKGGTTCCGGC
ATCGATSAAGAACGCAGCGAAWTGCGATAAGTAATGTGAATTGCAGAATTCCGTGAATCAT
CGAATCTTTGAACGCACATTGCGCCCCCTGGCATTCCGGGGGGCATGCCTGTCCGAGCGTCA
TTTCTGCCCTCAAGCACGGCTTGTGTGTTGGGTGTGGTCCCCCTGGGGACCTGCCCGAAAGG
CAGCGGCYACGTCCGTCTGGTCCTCGAGCGTATGGGGCTCTGTCACTCGCTCGGGAAGGACC

TGCGGGGGTTGGTCACCACCACATCTGTKTACAAGGTTGACCTCGGATCAGGTAGGAGTTAC
CCGCTTA
>BSNB_1046_Penicillium_simplicissimum

CGGGCCCTCACTCGGCAATGATCCTTCCGTAGGTGAACTTGCGGAAGGATCATTACCGAGTG
AGGGCCCTCTGGGTCCAACCTCCCACCCGTGTTTATCGTACCTTGTTGCTTCGGCGGGCCCGC
CTCACGGCCGCCGGGGGGCATCCGCTCCCGGGCCCGCGCCCGCGAAGACACCAATGAACTC
TGTCTGAAGATTGCAGTCTGAGCAGATTAGCTAAATCAGTTAAAACTTTCAACAACGGATCT
CTTGGTTCCGGCATCGATGAAGAACGCAGCGAAATGCGATACGTAATGTGAATTGCAGAAT
TCAGTGAATCATCGAGTCTTTGAACGCACATTGCGCCCCCTGGTATTCCGGGGGGCATGCCT
GTCCGAGCGTCATTGCTGCCCTCAAGCACGGCTTGTGTGTTGGGCTCCGCCCCCCGGCTCCC
GGGGGGCGGGCCCGAAAGGCAGCGGCGGCACCGCGTCCGGTCCTCGAGCGTATGGGGCTTG
TCACCCGCTCTGTAGGCCCGGCCGCGCCCGCCGGCGACCCCAATCAATCTCCAGGTTACCTC
GGAATCAGGT

>BSNB_1048_Neosartorya_sp

CACTCGGTAATGATCCTTCCGTAGGTGAACCTGCGGAAGGATCATTACCGAGTGAGGGCCCT
CTGGGTCCAACCTCCCACCCGTGTCTATTGTACCTTGTTGCTTCGGCGGGCCCGCCGTTTCGA
CGGCCGCCGGGGAGGCCTCGCGCCCCCGGGCCCGCGCCCGCCGAAGACCCCAACTGAACG
CTGTTCTGAAAGTATGCAGTCTGAGTTGATTATCATAATCAGTTAAAACTTTCAACAACGGA
TCTCTTGGTTCCGGCATCGATGAAGAACGCAGCGAAATGCGATAAGTAATGTGAATTGCAG
AATTCAGTGAATCATCGAGTCTTTGAACGCACATTGCGCCCCCTGGTATTCCGGGGGGCATG
CCTGTCCGAGCGTCATTGCTGCCCTCAAGCACGGCTTGTGTGTTGGGCCGCCGTCCCCGGTTT
CCCCCGGGGACGGGCCCGAAAGGCAGCGGCGGCACCGCGTCCGGTCCTCGAGCGTATGGG
GCTTTGTCACCCGCTCTGTAGGCCTGGCCGGCGCCAGCCGACCCCAACTTTATTTCAAGGTG
CACCTCGATTCATTC
>BSNB_1049_Penicillium_oxalicum

CGGTATGATCCTTCCGTAGGTGAACCTGCGGAAGGATCATACCGAGTGAGGGCCCTCTGGGT
CCAACCTCCCACCCGTGTTTATCGTACCTTGTTGCTTCGGCGAGCCCGCCTCACGGCCGCCGG
GGGGCATCTGCCCCCGGGCCTGCGCTCGCCGAAGACACACAAACGAACTCTTGTCTGAAGA
TTGCAGTCTGAGTACTTGACTAAATCAGTTAAAACTTTCAACAACGGATCTCTTGGTTCCGGC
ATCGATGAAGAACGCAGCGAAATGCGATAAGTAATGAATTGCAGAATTCAGTGAATCATCG
AGTCTTTGAACGCACATTGCGCCCCCTGGTATTCCGGGGGGCATGCCTGTCCGAGCGTCATT
GCTGCCCTCAAGCACGGCTTGTGTGTTGGGCTCTCGCCCCCCCCCTTGTGGGAGGGCGGGCC
CGAAAGGCAGCGGCGGCACCGTGTCCGGTCCTCGAGCGTATGGGGTTTGTCACCCGCTCTGT
AGGCCCGGCCGGCCCGCCGGCGAACCCAATCAACTTACCCATCTCACCTATCAAACCCCCC
G

>BSNB_1053_Beauveria_bassiana

GGCACCGCCAACAGGCGGCAAGGCTGGAGTATTTATTACCCTTGTCTTTTGCGCACTTGTTG
TTTCCTGGGCGGGTTCGCCCGCCTCCAGGACCACATGATAAACCTTTTTTATGCAGTTGCAAT
CAGCGTCAGTACAACAAATGTAAATCATTTACAACTTTCAACAACGGATCTCTTGGTTCTGG
CATCGATGAAGAACGCAGCGAAATGCGATACGTAGTGTGAATTGCAGAATTCAGTGAATCA
TCGAATCTTTGAACGCACATTGCGCCCTTTGGTATTCCAAAGGGCATGCCTGTTCGAGCGTCA
TTTGTACCCTCAAGCTTTGCTTGGTGTTGGGCGTTTTTGTCTTTGGTTTTGCCCAAAGACTCGC
CTTAAAACGATTGGCAGCCGGCCTACTGGTTTCGCAGCGCAGCACATTTTTGCGCTTGCAAT
CAGCAAAAGAGGACGGCACTCCATCAAGACTCTTTATCACTTTTGACCTCGGATCAGGTAGG
GATACCCGCTGAACTTAAGCATATCAATAAGCCGGAGGAA

>BSNB_1054_Pestalotiopsis_theae

AAGCTCCAACCCATGTGACTTACCTTTTTGTTGCCTCGGCAGAGGTTACCTGGTACCTGGAGA
CAGGTTACCCTGTAGCAACTGCCGGTGGACTACTAAACTCTTGTTATTTTATGTAATCTGAGC
GTCTTATTTTAATAAGTCAAAACTTTCAACAACGGATCTCTGGGTTCTGGCATCGATGAAGA
ACGCAGCGAAATGCGATAAGTAATGTGAATTGCAGAATTCAGTGAATCATCGAATCTTTGA
ACGCACATTGCGCCCATTAGTATTCTAGTGGGCATGCCTGTTCGAGCGTCATTTCAACCCTTA
AGCCTAGCTTAGTGTTGGGAATTTACAGTTATGTAATTCCTGAAATACAACGGCGGATCTGT
GGTATCCTCTGAGCGTAGTAAATTATTTCTCGCTTTTGTTAGGTGCTGCAGCTCCCAGCCGCT
AAACCCCCAATTTTTGTGGTTGACCTCGGATCAGGTAGGAATACCCGCTGAACTTAAGCAT
ATCAATAAGCGGAGGA
>BSNB_1055_Fusarium_sp

CTCCAAACCCCTGTGAACATACCTTATGTTGCCTCGGCGGATCAGCCCGCGCCCCGTAAAAA
GGGACGGCCCGCCGCAGGAACCCTAAACTCTGTTTTTAGTGGAACTTCTGAGTATAAAAAA
CAAATAAATCAAAACTTTCAACAACGGATCTCTTGGTTCTGGCATCGATGAAGAACGCAGC
AAAATGCGATAAGTAATGTGAATTGCAGAATTCAGTGAATCATCGAATCTTTGAACGCACAT
TGCGCCCGCCAGTATTCTGGCGGGCATGCCTGTTCGAGCGTCATTTCAACCCTCAAGCCCAG
CTTGGTGTTGGGAGCTGCAGTCCTGCTGCACTCCCCAAATACATTGGCGGTCACGTCGAGCT
TCCATAGCGTAGTAATTTACACATCGTTACTGGTAATCGTCGCGGCCACGCCGTTAAACCCC
AACTTCTGAATGTTGACCTCGGATCAGGTAGGAATACCCGCTGAACTTAAGCATATCATAAA
GCGGAGGA
>BSNB_1056_Lecanicillium_sp

ACCAAAACCCTTATGTGAACATACCACGATGTTGCTTCGGCGGACTCGCCCCGGCGTCCGGA
CGGCCTAGCGCCGCCCGCGGCCCGGATCCAGGCGGCCGCCGGAGACCACCAAAACTATTTT
GTATCAGCAGTTTTTTCTGAATCCGCCGCAAGGCAAAACAAATGAATCAAAACTTTCAACAA
CGGATCTCTTGGTTCTGGCATCGATGAAGAACGCAGCGAAATGCGATAAGTAATGTGAATTG
CAGAATTCAGTGAATCATCGAATCTTTGAACGCACATTGCGCCCGCCAGCATTCTGGCGGGC
ATGCCTGTTCGAGCGTCATTTCAACCCTCGACTTCCCTTTGGGGAAATCGGCGTTGGGGACTG
GCAGCATACCGCCGGCCCCGAAATGGAGTGGCGGCCCGTCCGCGGCGACCTCTGCGTAGTA
ATCCAACCTCGCACCGGAACCCCGACGTGGCCACGCCGTAAAACACCCCACTTTCTGAACG
TTGACCTCGGATCAGGTAGGAATACCCGCTGAACTTAAGCATATCAATAAGGCRGAGGA

>BSNB_1057_Fusarium_sp

TAGAACGTACGTAGAAAAAAAAAAAAAGTAGTAGCTCYYTYGCTCCYACCCCTCGACGTCCTT
ATGTTGCCTCGGCGGACACCCSCGCCCCGKAAAACGGGACGGCCCGCCAGAAAACCGAARC
TCTAATGTTTCTTATTGTAACTTCTGAGTWWWACAAACAAATAAATCAAAACTTTCAACAA
CGGATCTCTTGGTTCTGGCATCGATGAASAACGCAGCAAAATGCGATAAGTAATGTGAATTG
CAGAATTCWGTGAATCATCGAATCTTTGAACGCACATTGCGCCCGCTGGTATTCCGGCGGGC
ATGCCTGTTCGAGCGTCATTTCAACCCTCAAGCCCCCGGGTTTGGTGTTGGGGATCGGCTCTG
CCCTTCTGGGCGGTGCCGCCCCCGAAMTACATTGGCGGTCTCGCTGCASCCTCCATTGCGTA
GTAGCTAACACCTCGCAACTGGAACGCGGCGCGGCCATGCCGTAAAACCCCAACTTCTGAA
TGTTGACCTCGGATCAGGTAGGAATACCCGCTGAACTTAAGCATATCAATAAGCGGAGGAA
>BSNB_1058_Fusarium_sp

AAAGCCCCTGTGAACATACCTTAATGTTGCCTCGGCGGATCAGCCCGCGCCCCGTAAAACG
GGACGGCCCGCCAGAGGACCCAAACTCTAATGTTTCTTATTGTAACTTCTGAGTAAAACAAA
CAAATAAATCAAAACTTTCAACAACGGATCTCTTGGTTCTGGCATCGATGAAGAACGCAGC
AAAATGCGATAAGTAATGTGAATTGCAGAATTCAGTGAATCATCGAATCTTTGAACGCACAT
TGCGCCCGCTGGTATTCCGGCGGGCATGCCTGTTCGAGCGTCATTTCAACCCTCAAGCCCCC

GGGTTTGGTGTTGGGGATCGGCTCTGCCCTTCTGGGCGGTGCCGCCCCCGAAATACATTGGC
GGTCTCGCTGCAGCCTCCATTGCGTAGTAGCTAACACCTCGCAACTGGAACGCGGCGCGGCC
ATGCCGTAAAACCCCAACTTCTGAATGTTGACCTCGGATCAGGTAGGAATACCCGCTGAACT
TAAGCATATCAATAAGCCGGAGGAA
>BSNB_1059_Arthrinium_sp

CAACTCCATACCATCTGTTAACCTACCCAGTTATGCCTCGGCGTAAGCTCGGTTGGAGGCAC
CTGCAGCTACCCTGTAGTTGCGGACTGCCAACTCCAGCCGCGGCCCGCCGGCGGTACACTA
AACTCTGTTTTATTTTATATTCTGAGCGTCTTATTTTAATAAGTTAAAACTTTCAACAACGGAT
CTCTTGGTTCTGGCATCGATGAAGAACGCAGCGAAATGCGATAAGTAATGTGAATTGCAGA
ATTCAGTGAATCATCGAATCTTTGAACGCACATTGCGCCCATCAGTATTCTGGTGGGCATGC
CTGTTCGAGCGTCATTTCAACCCTTAAGCCTAGCTTAGTGTTGGGAATCTGCTGTACTGCAGT
TCCTTAAAGACAGTGGCGGAGCGGCGGTAGTCCTCTGAGCGTAGTAATTTATTTCTCGCTTTT
GTCAGGCTCTGTCCTCCCGCCATAAAACCCCCAATTTTTAGTGGTTGACCTCGGATCAGGTA
GGAATACCCGCTGAACTTAAGCATATCAATAARGCGGAGGA
>BSNB_1061_Penicillium_sp

GAACCCGCACTCGGTAATGATCCTTCCGTAGGTGAACCTGCGGAAGGATCATTACCGAGTG
CGGGTTCCAACGAGCCCAACCTCCCACCCGTGTCTACCGTTACCGCGTTGCCTCGGCGGGCC
CACTGGGGCCTGGCCCCGGTCGCCGGGGGGCTTCTGCCCCCGGGCCCGCGCCCGCCGAAGC
ACCCTAGAACCCTGTCTGAACAGTGAGTCTGAGTCGGATATTGAATCATTAAAACTTTCAAC
AACGGATCTCTTGGTTCCGGCATCGATGAAGAACGCAGCGAAATGCGATAAGTAATGTGAA
TTGCAGAATTCCGTGAATCATCGAATCTTTGAACGCACATTGCGCCCCCTGGCATTCCGGGG
GGCATGCCTGTCCGAGCGTCATTTCTGCCCTCCAGCACGGCTGGGTGTTGGGCGCTGTCCCCC
CGGGGACACGCCCCAAAAGCAGTGCGGCGCCGGTCGGGTCCTCGAGCGTATGGGGCTTTGT
CTCCGCTCGTTGGACTCGGTCGGCGCTGGTCTTAAAACAGGCGACCCCTCGGGGTCGCCTAT
CCGGTTCCCCTCGGAATTCCAAGG

>BSNB_1063_Mucor_hiemalis

CTTTGCTAGTTTTCTAGCGAATGGTTCATTCTTTTTACTGTGAACTGTTTTAATTTTTCAGCGT
CTGAGGAATGTCTTTTAGCCATAGGGATAGGCTACTAGAATGTTAACCGAGCTGAAAGTCAG
GCTTAGGCCTGGTATCCTATTAATTATTTACCAAAAGAATTCAGTATTATAATTGTAACATAA
GCGTAAAAAACTTATAAAACAACTTTTAACAACGGATCTCTTGGTTCTCGCATCGATGAAGA
ACGTAGCAAAGTGCGATAACTAGTGTGAATTGCATATTCAGTGAATCATCGAGTCTTTGAAC
GCAACTTGCGCTCAATGGTATTCCATTGAGCACGCCTGTTTCAGTATCAAAAACACCCCACA
TTCATAATTTTGTTGTGAATGGAAATGAGAGTTTCGGCTTTATTGCTGAATTCTTTAAAATTAT
TAGGCCTGAACTATTGTTCTTTCTGCCTGAACATTTTTTTAATATAAAGGAATGCTCTAGTAA
AAAGACTATCTCTGGGGCCTCCCAAATAAATCATTCTTAAATTTGATCTGAAATCAGGCGGG
ATTACCCGCTGAACTTAAGCATATCAATAA
>BSNB_1065_Fusarium_sp

AACTGCCAACCCCTGTGACATACCTTRATGTTGCCTCGGCGGATCAGCCCGCGCCCCGTAAA
ACGGGACGGCCCGCCAGAGGACCCAAACTCTAATGTTTCTTATTGTAACTTCTGAGTAAAAC
AAACAAATAAATCAAAACTTTCAACAACGGATCTCTTGGTTCTGGCATCGATGAAGAACGC
AGCAAAATGCGATAAGTAATGTGAATTGCAGAATTCAGTGAATCATCGAATCTTTGAACGC
ACATTGCGCCCGCTGGTATTCCGGCGGGCATGCCTGTTCGAGCGTCATTTCAACCCTCAAGC
CCCCGGGTTTGGTGTTGGGGATCGGCTCTGCCCTTCTGGGCGGTGCCGCCCCCGAAATACAT
TGGCGGTCTCGCTGCAGCCTCCATTGCGTAGTAGCTAACACCTCGCAACTGGAACGCGGCGC
GGCCATGCCGTAAAACCCCAACTTCTGAATGTTGACCTCGGATCAGGTAGGAATACCCGCTG
AACTTAAGCATATCAATAAGCGGAGGAA

>BSNB_1067_Penicillium_sp

CCTTCCGTAGGTGAACCTGCGGAAGGATCATTACCGAGTGCGGGCCCTCGCGGCCCAACCTC
CCACCCTTGTCTCTCTACACCTGTTGCTTTGGCGGGCCCACCGGGGCCACCCGGTCGCCGGG
GGACGCTCGTCCCCGGGCCCGCGCCCGCCGAAGCGCCCTGTGAACCCTGATGAAGATGGAC
TGTCTGAGTACCATGAAAATTGTCAAAACTTTCAACAATGGATCTCTTGGTTCCGGCATCGAT
GAAGAACGCAGCGAAATGCGATAAGTAATGTGAATTGCAGAATTCCGTGAATCATCGAATC
TTTGAACGCACATTGCGCCCCCTGGCATTCCGGGGGGCATGCCTGTCCGAGCGTCATTTCTGC
CCTCAAGCACGGCTTGTGTGTTGGGTGCGGTCCCCCCGGGGACCTGCCCGAAAGGCAGCGG
CGACGTCCGTCCGGTCCTCGAGCGTATGGGGCTTTGTCACTCGCTCGGGAAGGACCTGCGGG
GCGTTGGTCACCACCATATTTGACCTACGGCTGATTACCC

>BSNB_1069_Beauveria_sp

CTACCAACCCTTATGTGACCTACCTATCGTTGCTTCGGCGGACTCGCCCCAGCCGGACGCGG
ACTGGACCAGCGGCCGCCGGGGACCATCAAACTCTTGTATTATCAGCATCTTCTGAATACGC
CGCAWGGCAAAACAAATAAATTAAAACTTTCAACAACGGATCTCTTGGCTCTGGCATCGAT
GAAGAACGCAGCGAAATGCGATAAGTAATGTGAATTGCAGAATCCAGTGAATCATCGAATC
TTTGAACGCACATTGCGCCCGCCAGCATTCTGGCGGGCATGCCTGTTCGAGCGTCATTTCAA
CCCTCGACCTCCCTTTGGGGAAGTCGGCGTTGGGGACCGGCAGCACACCGCCGGCCCTGAA
ATGGAGTGGCGGCCCGTCCGCGGCGACCTCTGCGTAGTAAACCAACTCGCACCGGAACCCC
GACGTGGCCACGCCGTAAAACACCCAACTTCTGAACGTTGACCTCGAATCAGGTAGGACTA
CCCGCTGAACTTAAGCATATCAATAAGCCGGAGGA
>BSNB_1250_Isaria_farinosa

GCGGAGGGATCATTACCGAGTTTTCAACTCCCAAACCCTTTTGTGAACATACCTATCGTTGCT
TCGGCGGACTCGCCCCAGCGTCCGGCCGGCCCCGCGCCGGCCGCGGCCTGGATCCAGGCGG
CCGCCGGAGACCCCCAAACTCTGTATTCTCAGTATCTTCTGAATCCGCCGCAAGGCAAAACA
AATGAATCAAAACTTTCAACAACGGATCTCTTGGTTCTGGCATCGATGAAGAACGCAGCGA
AATGCGATAAGTAATGTGAATTGCAGAATTCAGTGAATCATCGAATCTTTGAACGCACATTG
CGCCCGCCAGCATTCTGGCGGGCATGCCTGTTCGAGCGTCATTTCAACCCTCGACTTCCCTTT
GGGGAAATCGGCGTTGGGGACCGGCCGTATACCGCCGGCCCCGAAATGAAGTGGCGGCCC
GTCCGCGGCGACCTCTGCGTAGTAATCCAACTCGCACCGGAACCCCGACGTGGCCACGCCG
TAAAACCCCCGACTTCTGAACGTTGACCTCGGATCAGGTAGGAATACCCGCTGAACTTAAGC
ATATCA
>BSNB_1252_Cordyceps_militaris

GCGGAGGGATCATTAACGAGTTTCCCAACTCCCAACCCTTTGTGAACATACCTATCGTTGCTT
CGGCGGACTCGCCCAGCGCCTGGACGCGGGCCTGGGCGGCGGCCGTCGGGGGCCCCAAAC
ACTGTATCTACCAGTTTTTCTGAATCCGCCGCAAGGCAAAACAAATGAATCAAAACTTTCAA
CAACGGATCTCTTGGCTCTGGCATCGATGAAGAACGCAGCGAAATGCGATAAGTAATGTGA
ATTGCAGAATTCAGTGAATCATCGAATCTTTGAACGCACATTGCGCCCGCCAGCATTCTGGC
GGGCATGCCTGTTCGAGCGTCATTTCAACCCTCGACGTCCCCTGGGGGATGTCGGCGTTGGG
GACCGGCAGCACACCGCCGCCCCCGAAATGAAGTGGCGGCCCGTCCGCGGCGACCTCTGCG
TAGTACCCCAACTCGCACCGGGAACCCGACGTGGCCACGCCGTAAAACGCCCAACTCTGAA
CGTTGACCTCGGATCAGGTAGGAATACCCGCTGAACTTAAGCATATC

>BSNB_1253_Ophiocordyceps_forquignonii

GCGGAGGGATCATTACTGAGTGTTAAAACTCTCTAACCCCCTATGTGATTACTACCATTTTTA
TTCCTCGGCAGTGTAAGGCGGGAAGAGTATCCCCCTCTATATTTCTCTATTATAGGGGATTCT
AGAGGGGCGCTGCTCCCCCCTCTCCCCCGCTGCCGGTGGAAACCACTATAATCTATATTTCA

TCTATTGAGTCTTCTGAGTTTTATAATACAAAACGCATAAAAACTTTCAGCAACGGATCTCTT
GGCTCTGGCATCGATGAAGAACGCAGCGAAATGCGATAAGTAATGCGAATTGTAGATTTCA
GTGAGTCATCGAATCTTTGAACGCACATTGCGCCTGCTGGCCATTCCAGCAGGCATGCCTGT
CCGAGCGTCATTATCAACCCCTCTCAGGCTTTCTTATAGGGTTTTAATAGGACCCTATATCGA
TTGACTGGCACCTGGAGATTGGCCGTGCCCCTCTTGTTTTTTTCTTAAAACCAAGGGGGAAGG
CCAGCTCTGAAATACAGTGGCGACTCGAGGTCTTACTTCTTCCCCTGGCAGTAAAACTCTTG
CTCGCAACGGGACTAGTGACCTTGTGTTAGCCATCTTAACACCCTAT

FIGURE S1. R SCRIPT USED: GENERATION OF A .MGF AND .CSV FILES

```
```{r setup, include=FALSE}

library(MALDIquant)

library(MALDIquantForeign)


path <- "C:/Users/Desktop/…" #enter directory path

setwd(path)


spectra <- import(path, verbose=FALSE, excludePattern = NULL)


liste_fichiers <- list.files(path)

NameListe = as.list(liste_fichiers)

condition = c(15,       ## HwS

              30,       ## baseline

              6,        ## SnR

              2000, 20000)     ## Trim

spectra2 = transformIntensity(spectra, method="sqrt")

spectra2 = trim(spectra2, range = c(condition[4], condition[5] ))

spectra2 = smoothIntensity(spectra2, method="SavitzkyGolay",

                           halfWindowSize=condition[1])

spectra2 <- removeBaseline(spectra2, method="SNIP",

                           iterations=condition[2])

spectra2 <- calibrateIntensity(spectra2, method="TIC")

peaks <- detectPeaks(spectra2, SNR=condition[3],
halfWindowSize=condition[1])

a = plot(peaks[[1]])


peaks <- binPeaks(peaks)


liste = list()
```
```

````
mgf <- paste(path, ".mgf", sep = "")

for (i in 1:length(peaks)) {

    test = matrix(data = NA, nrow = length(peaks[[i]]@mass), ncol= 2)

    colnames(test) = c("m/z", "intensity")

    test[,1] = peaks[[i]]@mass

    test[,2] = peaks[[i]]@intensity



    write.table("BEGIN IONS", file = mgf, append = TRUE, quote = FALSE, sep
= "\t ", row.names = FALSE, col.names = FALSE)

    write.table("PEPMASS=0", file = mgf,  append = TRUE, quote = FALSE, sep
= "\t ", row.names = FALSE, col.names = FALSE)



    write.table(list(test), file = mgf,  append = TRUE, quote = FALSE, sep
= "\t ", row.names = FALSE, col.names = FALSE)

    write.table("END IONS", file = mgf,  append = TRUE, quote = FALSE, sep
= "\t ", row.names = FALSE, col.names = FALSE)

    write.table("", file = mgf,  append = TRUE, quote = FALSE, sep = "\t ",
row.names = FALSE, col.names = FALSE)

}



csv <- paste(path, ".txt", sep = "")

for (i in 1:length(liste_fichiers)) {

    write.table(liste_fichiers[i], file = csv, append = TRUE, quote =
FALSE, sep = "\t ", eol = "\n", na = "NA", dec = ".", row.names = FALSE,
col.names = FALSE, qmethod = c("escape", "double"))

}

```
````
````

TABLE S6. PARAMETERS USED FOR CONSTRUCTING CHEMOTAXONOMIC NETWORKS ON METGEM

**Figures**	**Treat as MS^1 Data**	***m/z* Tolerance**	**Minimum Matched Peaks**	**Keep peaks above**	**Cosine score(s) above**	**Number of iterations**	**Learning Rate**	**Perplexity**
2	Used	2	2	1%	0.65	10 000	10	11
3	Used	2	2	1%	0.4	10 000	10	11
4	Used	0.1	4	1%	0.65	10 000	10	11
5	Used	4	2	1%	0.35	10 000	10	16
6	Used	0.1	4	1%	0.45	10 000	10	11
